# Quantitative proteomic analysis reveals a simple strategy of global resource allocation in bacteria

**DOI:** 10.15252/msb.20145697

**Published:** 2015-02-12

**Authors:** Sheng Hui, Josh M Silverman, Stephen S Chen, David W Erickson, Markus Basan, Jilong Wang, Terence Hwa, James R Williamson

**Affiliations:** 1 Department of Physics, University of California at San DiegoLa Jolla, CA, USA; 2 Department of Integrative Structural and Computational Biology, The Skaggs Institute for Chemical Biology, The Scripps Research InstituteLa Jolla, CA, USA; 3 Department of Chemistry, The Skaggs Institute for Chemical Biology, The Scripps Research InstituteLa Jolla, CA, USA; 4 Section of Molecular Biology, Division of Biological Sciences, University of California at San DiegoLa Jolla, CA, USA

**Keywords:** growth physiology, metabolic network, microbiology, quantitative proteomics, systems biology

## Abstract

A central aim of cell biology was to understand the strategy of gene expression in response to the environment. Here, we study gene expression response to metabolic challenges in exponentially growing *Escherichia coli* using mass spectrometry. Despite enormous complexity in the details of the underlying regulatory network, we find that the proteome partitions into several coarse-grained sectors, with each sector's total mass abundance exhibiting positive or negative linear relations with the growth rate. The growth rate-dependent components of the proteome fractions comprise about half of the proteome by mass, and their mutual dependencies can be characterized by a simple flux model involving only two effective parameters. The success and apparent generality of this model arises from tight coordination between proteome partition and metabolism, suggesting a principle for resource allocation in proteome economy of the cell. This strategy of global gene regulation should serve as a basis for future studies on gene expression and constructing synthetic biological circuits. Coarse graining may be an effective approach to derive predictive phenomenological models for other ‘omics’ studies.

## Introduction

One of the most extensively studied questions in biology is how cells alter gene expression to deal with changes in their environment. A widely held view, supported by a mountain of observations, is the idea that cells handle challenges to growth-limiting perturbations, for example, nutrient limitation, by increasing the amount of enzymes devoted to overcoming the limited process, in analogy with ‘supply and demand’ (Hofmeyr & Cornish-Bowden, 2000). This qualitative picture has been widely articulated in conceptual models from early on (Hinshelwood, 1944) to the present and is supported by analyses of 2D gel experiments (O'Farrell, 1975), microarrays (Brown & Botstein, 1999), deep sequencing (Ingolia *et al*, 2009), mass spectrometry (Aebersold & Mann, 2003), and other high-throughput measurements of gene expression (Ghaemmaghami *et al*, 2003; Taniguchi *et al*, 2010). For example, cells grown in minimal media increase the level of amino acid synthesis enzymes compared to rich media, and cells grown in the presence of translation inhibitors increase the synthesis of ribosomes (Dennis, 1976; Tao *et al*, 1999; Boer *et al*, 2003). Despite these results, there is little in the way of a quantitative understanding of resource allocation even in the simplest cells (Chubukov & Sauer, 2014).

Recently, it was shown that simple genetic circuits respond to changes in the physiological state of a cell in different ways, based upon the details of their defined regulation (Klumpp *et al*, 2009). At the molecular level, a cell's response to an applied limitation is the outcome of a complex interaction of metabolites, transcription factors, promoters, and other factors, conspiring to produce the observed pattern of gene expression. It is therefore unclear how the behavior of single genes under defined and specific regulation can be generalized to shifts in global gene expression arising from environmental changes. Many elementary questions remain unaddressed. In response to a growth-limiting perturbation, by how much does the cell adjust its composition to deal with the limiting process(es)? Does the cell handle limitation in the supply of a given nutrient by adjusting operons related to the specific shortage, or is gene expression organized according to some higher schema? Can the effect of different types of growth limitations be meaningfully compared? From the perspective of analysis, can cellular response, with changes in thousands of quantities as revealed by ‘omics’ experiments, be summarized by simple quantitative measures beyond statistical analysis? In characterizing the state of a gas, useful quantitative measures are macroscopic quantities such as pressure and temperature, not the statistical clustering of the trajectories of molecules in the gas. In systems biology, might similar measures exist to provide meaningful quantitative characterization of cellular responses?

Early studies of bacterial physiology identified a number of relations between the cell growth rate and quantities such as chromosome copy number, cell mass, and ribosome content (Schaechter *et al*, 1958; Bremer & Dennis, 2009). Despite the incredible complexity of ribosome biogenesis and its regulation, the proportion of translational machinery among all proteins can be captured by a simple linear relation with the cell growth rate (Bennett & Maaloe, 1974). These observations hint to a quantitative framework underlying the intuitive ‘supply and demand’ picture. The hint is the balance between the flux of amino acids synthesized into proteins by ribosomes and the flux of molecular building blocks from catabolic and biosynthetic reactions culminating in amino acids that are consumed by the ribosomes. This highlights an attractive possibility. If enzymes are regulated as subsets according to their shared purpose, as the hundred or so genes involved in translation are, it may be possible to capture their collective behavior quantitatively as is possible for the translational machinery. Rather than focusing upon the molecular details of hundreds of enzymes as they facilitate myriad reactions, the enzymes of a functional group might instead be profitably viewed as a single effective coarse-grained enzyme that catalyzes interconversion between major metabolic pools, such as carbon precursors to amino acids. In this view, proteome-wide response to nutrient limitations may be characterized quantitatively as adjustments to the concentrations of coarse-grained enzymes. This coarse-grained view of the proteome yields a simple picture that is amenable to mathematical analysis. Recently, the coarse-graining approach has been used to address the effects of protein overexpression (Scott *et al*, 2010), cAMP-mediated catabolite repression (You *et al*, 2013), growth bistability in response to antibiotics (Deris *et al*, 2013), and methionine biosynthesis (Li *et al*, 2014). But these studies focused on the expression of only a few genes, declared to be proxies for hundreds of proteins (Scott *et al*, 2010; You *et al*, 2013), or isolated in a backdrop of changing proteome (Deris *et al*, 2013; Li *et al*, 2014). There has been no study of its global applicability and, indeed, no work to predict quantitative proteome composition from physiological state.

Toward this end, it is our aim to quantitatively characterize global gene expression under various modes of growth limitation and to interrogate the intuitive ideas regarding resource allocation quantitatively. Samples were collected for *E. coli* cells growing exponentially in a variety of growth conditions: under titration of carbon import and nitrogen assimilation, and in the presence of varying amounts of translation inhibitor. Using quantitative mass spectrometry, the relative concentrations of ∽1,000 enzymes were measured across the set of growth-limiting conditions. Analysis of the enzyme concentrations reveals six groups of enzymes with distinct modes of gene expression in response to the applied limitations. An enrichment analysis of gene ontology terms appearing in these groups shows that each group consists of enzymes with uniform purpose, such as translation and catabolism. The cell up-regulates relevant groups to counteract the imposed limitation, confirming the qualitative expectations based on supply and demand. A key to this analysis is the concept of an ‘effective concentration’ for each coarse-grained enzyme, obtained as the fractional abundance of the sum of all its enzyme components among all expressed proteins in each condition. The concentration of the coarse-grained enzymes was estimated using coarse-grained spectral counts as a proxy for protein abundance (Malmström *et al*, 2009). Strikingly, the concentrations of these coarse-grained enzymes correlated linearly with the growth rate. These data, together with the intrinsic constraints of finite resource allocation, led to the construction of a self-consistent, flux-matching model of the proteome that not only quantitatively accounts for all the observed data but also predicts proteome composition in novel environments involving combinatorial modes of growth limitation.

## Results

### Growth limitations

**Figure 1:**
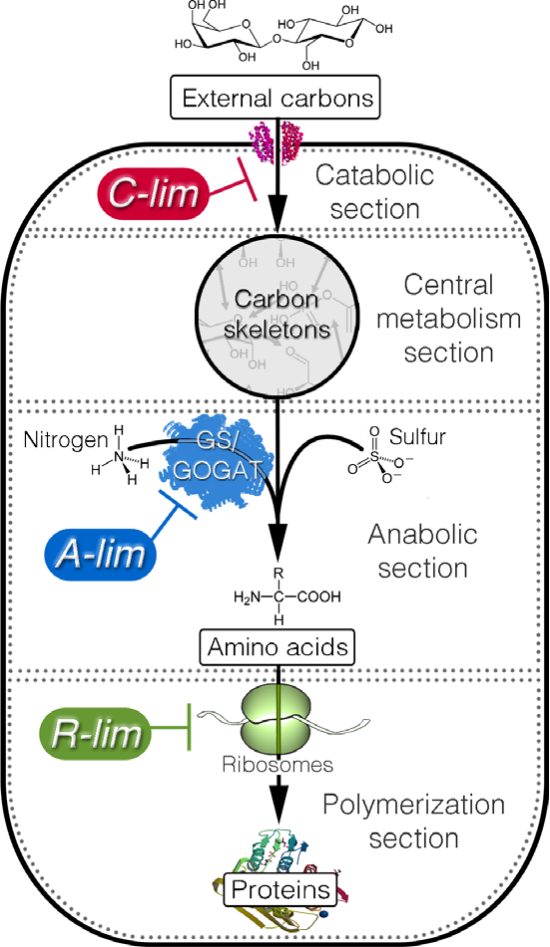
Coarse-grained metabolic flow of protein production and the three modes of growth limitation Through the (carbon) *catabolic section,* the cells take up external carbon sources and break them down into the set of standard carbon skeletons (pyruvate, oxaloacetate, etc.). The carbon skeletons are interconvertible through the *central metabolism section*. The *anabolic section* synthesizes amino acids from the carbon skeletons and other necessary elements such as ammonia and sulfur. The amino acids are then assembled into proteins by the *polymerization section*. The three modes of growth limitation were imposed on the metabolic sections as shown. The C-limitation (*C-lim*) and A-limitation (*A-lim*) were carried out with strains constructed for titrating the catabolic and anabolic flux, respectively; see Supplementary Figs S1 and S2, and Supplementary Table S1. The R-limitation (*R-lim*) was realized for the WT strain by supplying the growth medium with various levels of an antibiotic, chloramphenicol.

To probe gene expression, cell growth was perturbed by imposing three different modes of growth limitation at crucial bottlenecks in the metabolic network. A coarse-grained metabolic flow diagram for protein production by *E. coli* growing in minimal medium is shown in Fig1. Four *metabolic sections* act in concert to convert external carbon sources to proteins, incorporating nitrogen and sulfur elements during the process. Following the work of You *et al* (You *et al*, 2013), growth limitation was imposed on three of the four metabolic sections. The limitation imposed on the catabolic section (C-limitation or *C-lim*) was implemented by titrating the expression of lactose permease for cells growing on lactose (Supplementary Fig S1). The limitation on the anabolic section (A-limitation or *A-lim*) was realized by titrating a key enzyme (GOGAT) in the ammonia assimilation pathway (Supplementary Fig S2). Such ‘titratable uptake systems’ have been characterized in detail and found comparable to other modes of growth limitations such as those derived from continuous culture or microfluidic devices (You *et al*, 2013). To impose growth limitation on the polymerization sections, sublethal amounts of a translation inhibitor antibiotic, chloramphenicol, were supplied to the growth medium to inhibit translation by ribosomes (R-limitation or *R-lim*). The collective response of the *E. coli* proteome to these applied growth limitations was monitored using quantitative mass spectroscopy.

### Quantitative proteomic mass spectrometry

Proteomic mass spectrometry is a powerful tool for quantifying changes in global protein expression patterns (Aebersold & Mann, 2003; Ong & Mann, 2005; Bantscheff *et al*, 2007; Han *et al*, 2008). As shown below, mass spectrometry also has the advantage of reliably detecting small changes in protein levels, with precision comparable to that of enzymatic assays. Metabolic labeling with ^15^N (Oda *et al*, 1999) provides relative quantitation of unlabeled proteins with respect to labeled proteins across growth conditions of interest. Each experimental sample in a series is mixed in equal amount with a known labeled standard sample as reference, and the relative change of protein expression in the experimental sample is obtained for each protein.

#### Accuracy and precision

**Figure 2:**
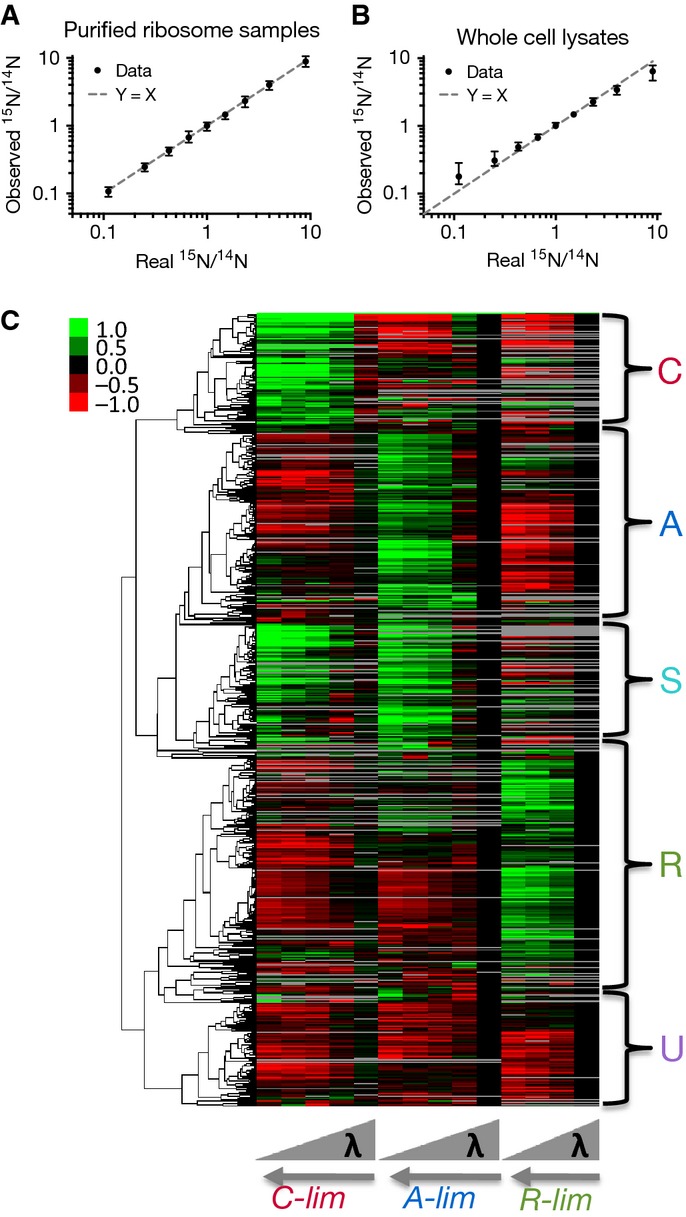
The quantitative protein mass spectrometry A Observed values versus real values for ratios of ^15^N ribosomal proteins to ^14^N ribosomal proteins. Black dots are the mean values, with error bars representing the range of the values for all ribosomal proteins. The dashed line represents perfect agreement between the observed values and real values.

The accuracy and precision of quantifying relative protein expression levels was determined from a standard curve using samples of unlabeled and ^15^N-labeled purified ribosomes. The observed relative levels, measured by ratios of the labeled to the unlabeled ribosomal proteins (or ^15^N/^14^N), agree extremely well with the expected values over a range of about two orders of magnitude (Fig2A). To assess the accuracy and precision for a whole-cell lysate with a much more complex proteome, labeled and unlabeled cells were mixed in fixed ratios and measured with quantitative mass spectrometry. The relative changes in protein levels can be precisely determined over the range of ratios from 0.1 to 10, as shown in Fig2B. The effective precision of relative protein quantification is ±18%, based on analysis of the 1:1 sample (Supplementary Fig S3). Thus, subtle changes in proteome composition that are much < 2-fold can be precisely determined. Furthermore, the relative quantitation using quantitative mass spectrometry agrees extremely well with a traditional biochemical measurement of ribosome content (Supplementary Fig S4A) and also with quantitation of LacZ using a β-galactosidase assay (Supplementary Fig S4B).

B Observed values versus real values for ratios of ^15^N proteins to ^14^N proteins from whole-cell lysates. Black dots are the median values for more than 600 proteins. The error bar for each median value indicates the quartiles. The dashed line represents perfect agreement between the observed values and real values. Additional characterizations are shown in Supplementary Figs S3 and S4.

C The expression matrix and clustering results. The matrix is composed of 1,053 proteins (rows) and 14 conditions (columns); see Supplementary Table S2. The first five columns are for C-limitation, the next five columns for A-limitation, and the last four columns for R-limitation. For each mode of growth limitation, the growth rate increases from left to right. The matrix is log2-transformed, with expression values at the standard condition as zero (see Materials and Methods), represented as black color. Red color indicates negative values, green color positive values, and gray color missing entries. A dendrogram generated by clustering analysis is shown on the left of the expression matrix (see Materials and Methods), with the five major clusters shown on the right side of the matrix. The data are estimated to cover ˜80% of the proteome; see Supplementary Fig S5.

#### Datasets and protein coverage

For the C-, A-, and R-limitations, a series of cultures were prepared with varying growth rates. For the C-limitation series, controlled inducible expression of the lacY gene gave doubling times from 40 to 92 min (five conditions), for the A-limitation series, controlled expression of GOGAT gave doubling times from 43 to 91 min (five conditions), and for the R-limitation series, inhibition of protein synthesis with chloramphenicol gave doubling times from 42 to 147 min (four conditions), as detailed in Supplementary Table S1. Samples from each of the fourteen cultures were collected, and the relative protein levels were determined using mass spectrometry, as described in the Materials and Methods. For C-, A-, and R-limitations, the numbers of proteins with reliable expression data are 856, 898, and 756, respectively. Most proteins present in one dataset are present in others, with 616 proteins shared in all three datasets and a total of 1,053 unique proteins in any dataset. Due to a highly non-uniform distribution of protein abundance, our experiments are estimated to cover ∽80% of the total proteome by mass and are validated using absolute abundance estimated by a recent experiment using ribosome profiling (Li *et al*, 2014); see Supplementary Fig S5. For data analysis, the combined datasets were represented as a matrix of 1,053 proteins across the 14 growth conditions (Supplementary Table S2), graphically shown in Fig2C.

#### Clustering analysis of protein expression trends

A qualitative global analysis of the data was performed with hierarchical clustering using the Pearson correlation as a distance metric (Materials and Methods), and the resulting dendrogram is shown on the expression matrix in Fig2C. Five major clusters are apparent, characterized by different trends in the three limitation series. The cluster where protein levels increase as growth rate is reduced under C-limitation, but decrease under A- and R-limitations, represents proteins that specifically respond to C-limitation and is designated as the C-cluster. The A-cluster is defined by increased protein levels under A-limitation, but decreased levels under C- and R-limitations, responding specifically to A-limitation. Similarly, the cluster where proteins levels increase in response to R-limitation, but decrease under C- and A-limitations, specifically respond to R-limitation and is designated as the R-cluster. The S-cluster is defined by protein levels that increase under both A- and C-limitations. Finally, the cluster for proteins that generally do not respond specifically to any of the three modes of growth limitation is designated as the U-cluster.

The clustering analysis is useful for providing an overview of the trends in the proteomic data, and revealing the qualitative responses of proteins to the different modes of growth limitation: Most proteins respond specifically to a single mode of growth limitation with the exception of the S-cluster. These clusters suggest that proteome levels are strongly coordinated based on the environmental stress and that the response of the proteome to the environment might be amenable to a quantitative coarse-graining analysis.

### Coarse-grained proteome sectors

Extensive analysis of a number of exemplary reporters of catabolic and biosynthetic gene expression revealed strikingly linear growth rate dependence in the expression of these genes (You *et al*, 2013). The prevalence of linear growth rate dependence has been described in omics studies of both proteins (Pedersen *et al*, 1978) and mRNAs (Brauer *et al*, 2008). Visual inspection of the expression data of individual proteins in Fig2C (see Supplementary Dataset S1 for individual plots) suggested that many exhibited a linear trend, and the coefficient of determination (*R*^2^) for the expression of each protein was calculated for each mode of growth limitations. The cumulative distribution of *R*^2^ for each mode of growth limitation shows that linear dependence on growth rate is widespread in our data (Supplementary Fig S6A), and is further supported by comparison with a quadratic fit of the data (Supplementary Fig S6B). Possible causes for the occurrence of low *R*^2^ values include limited method precision (Supplementary Fig S7A) and weak growth rate dependence for some genes (Supplementary Fig S7B and C). The approximate linear nature of the protein abundance data suggests that the results may be simplified using a coarse-grained analysis, by summing over the absolute abundance of individual proteins in a cluster (since the sum of linear functions is still linear).

For a protein exhibiting linear growth rate dependence, a negative slope corresponds to a higher expression level at slower growth rate, referred to as the ‘upward’ response (^↑^), while a positive slope corresponds to a lower expression level at slower growth rate, referred to as the ‘downward’ response (^↓^). Given that a protein has either upward or downward response under each of the three modes of growth limitation (C-, A-, and R-limitation), it has to belong to one of the 2^3^ = 8 groups: C^↑^A^↓^R^↓^, C^↑^A^↑^R^↓^, C^↓^A^↑^R^↓^, C^↓^A^↑^R^↑^, C^↓^A^↓^R^↑^, C^↑^A^↓^R^↑^, C^↑^A^↑^R^↑^, and C^↓^A^↓^R^↓^, where the group names are indicated by the upward or downward response under each of the three modes of growth limitation. For example, the C^↑^A^↓^R^↓^ group consists of proteins that have upward response under C-limitation and downward responses under both the A- and R-limitation. The membership of proteins in the resulting eight groups is given in Supplementary Table S2 and graphically shown in Supplementary Fig S8. Due to the precision limitations of the method, proteins exhibiting small change under a specific growth limitation are subject to misclassification. To examine the effect of this misclassification on our results, we carried out a probabilistic classification, by assigning a protein to one of the eight groups according to a probability (see Supplementary Text S2 for details). The analysis shows a very limited effect misclassification has on the binary classification.

**Figure 3:**
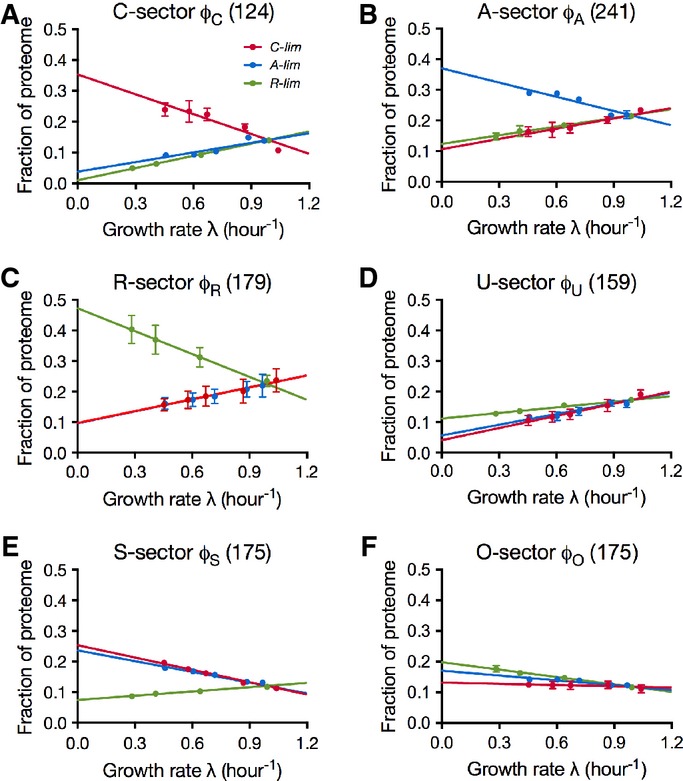
The coarse-grained proteome sectors A–F Coarse-grained responses of the C-, A-, R-, U-, S-, and O-sectors to the three modes of growth limitation. As indicated in (A), the red symbols in each panel are for C-limitation, the blue for A-limitation, and the green for R-limitation. The error bars indicate the standard deviation of triplicate mass spectrometry runs. Error bars smaller than the corresponding symbols are not shown (see Supplementary Fig S10 on the different degrees of variability associated with different sectors.) On each plot, the number in the title indicates the number of proteins in that sector, and colored lines are best linear fits of the data represented by symbols of the same colors; see Supplementary Table S3 for the data on proteome fraction and Supplementary Table S4 for parameters of the fitted lines.

The collective behavior of a protein group can be approximated by coarse graining, effectively summing the absolute protein abundance of proteins in the same group. Among the methods for quantifying absolute protein abundance from proteomic mass spectrometry data (Beynon *et al*, 2005; Ishihama *et al*, 2005, 2008; Lu *et al*, 2006; Silva *et al*, 2006; Vogel & Marcotte, 2008; Schmidt *et al*, 2011; Muntel *et al*, 2014), the method of spectral counting takes the number of peptides recorded for each protein as proxy for the absolute abundance of the protein (Malmström *et al*, 2009). While spectral counting provides a crude estimate of the absolute protein abundance for individual proteins (Bantscheff *et al*, 2007), it gives a much more reliable approximation for groups of proteins. For a protein group comprising more than ∽5% of the total proteome, spectral counting produces estimates with < 20% error (Supplementary Fig S9A). The comparison of spectral counting data for ribosomal proteins with estimates based on biochemical measurements and the ribosome profiling results (Li *et al*, 2014) is in good agreement (Supplementary Fig S9B). By applying the spectral counting method, the proteome fractions for the nine protein groups defined in Supplementary Table S2 were determined for each of the three series of growth limitations (Supplementary Fig S10). It is clear from Supplementary Fig S10 that some groups occupy significant fractions of the proteome while others are minor constituents. Ranked by the extent the fraction varies (indicated by the difference between the maximal and minimal intercepts on the *y*-axis), the top three groups are C^↑^A^↓^R^↓^, C^↓^A^↑^R^↓^, and C^↓^A^↓^R^↑^. These consist of proteins that only respond upward to the C-, A-, and R-limitation and are referred to as the C-, A-, and R-sector, respectively (FigA–C). The C^↓^A^↓^R^↓^ group includes proteins that are uninduced by any of the three applied limitations, and is referred to as the U-sector (Fig3D). Another significant protein sector is the C^↑^A^↑^R^↓^ group, which is composed of proteins that have upward response to both the A- and C-limitations, and referred to as the S-sector for general starvation; see Fig3E. The three remaining groups (i.e., C^↑^A^↑^R^↑^, C^↑^A^↓^R^↑^, and C^↓^A^↑^R^↑^ groups) are small, with most of the data at or below 5% of the proteome, below the accuracy of the spectral counting method (Supplementary Fig S9A). The three small groups were placed together into the O-sector (FigF). In summary, the proteome is coarse-grained into 6 ‘sectors’: C-, A-, R-, U-, S-, and O-sectors with distinct growth rate dependences as shown in Fig3, with complete data for all fractions shown in Supplementary Table S3. In contrast, the results obtained for randomly shuffled expression matrices do not show significant growth rate dependence (Supplementary Fig S11).

### Qualitative proteome responses to growth limitations

To elucidate the biological functions for each proteome sector, a Gene Ontology (GO) analysis was carried out using an *abundance-based* GO term enrichment to identify a small number of GO terms that best represent the abundant proteins in a sector. To reach such a list of GO terms, instead of calculating a single score of one measure (e.g., enrichment) for each GO term as in many GO analyses, we have taken a multi-step procedure to search for the best representing GO terms by examining a number of different measures such as coverage and overlap. The procedure leads to only a few GO terms accounting for more than 60% of the proteome in the sector; see Supplementary Text S3.

**Figure 4:**
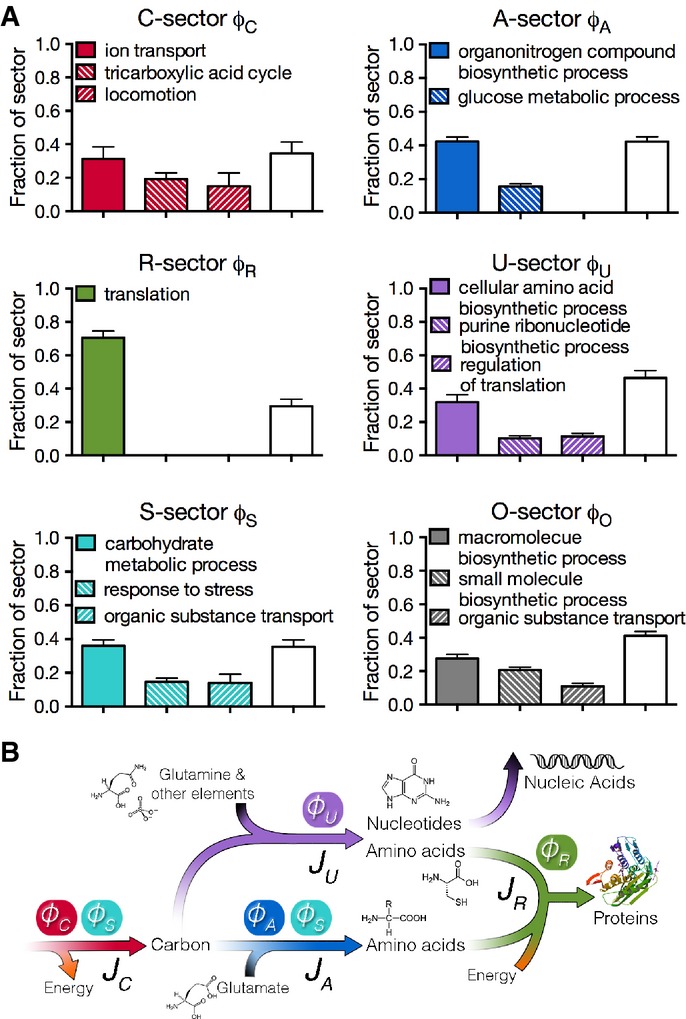
Abundance-based GO analysis A Composition of proteome sectors. Each bar graph shows the results of the abundance-based Gene Ontology analysis for each of the six sectors. Each bar indicates the mass fraction the corresponding GO term accounts for within a sector. The empty bar in each graph indicates the remaining fraction of the sector not accounted for by the GO terms listed. The results were calculated based on triplicate runs of all samples. Each bar height indicates the mean result and the standard deviation is shown as the error bar. See Supplementary Table S9 for the list of proteins represented by each bar in each sector, and Supplementary Text S3 for details of the method.

The results of the analysis are summarized in Fig4A, with each bar graph describing the major proteome composition for each sector. Sixty percent of the mass fraction of each sector could be accounted for by at most three terms, providing a simple interpretation of the functional significance of the sectors. For example, a single GO term, ‘translation’, describes more than 70% of the proteins in the R-sector. Since the R-limitation inhibits translation rate, the term suggests a strategy by which the cell specifically counteracts the applied growth limitation by increasing the abundance of ‘translational’ proteins (Scott *et al*, 2010).

B Association between metabolic fluxes and proteome sectors. There are four fluxes *J*_*C*_, *J*_*A*_, *J*_*U*_, and *J*_*R*_, represented by the arrows, replenishing the pools of carbon precursors, amino acids, other building blocks, and macromolecules, respectively. The *ϕ*_*s*_ on top of the fluxes represents the corresponding proteome fractions that carry the fluxes. Note that the S-sector proteins contribute to both *J*_*C*_ and *J*_*A*_.

The GO terms best describing the C-sector are ‘ion transport’, ‘tricarboxylic acid cycle’, and ‘locomotion’, pointing to a mode of carbon scavenging (by moving and increasing carbon uptake) and carbon saving (by increasing the efficiency of energy generation using the tricarboxylic acid cycle) to counteract the imposed carbon limitation. For the A-sector, the most abundant term is ‘organonitrogen compound biosynthetic process’. A closer look reveals that most of the terms are related to biosynthesis of amino acids (Supplementary Table S5). Again, similar to the responses to R- and C-limitations, the finding here suggests that the cell tries to counteract the imposed A-limitation, which specifically limits the biosynthesis of amino acids (Supplementary Fig S2). Interestingly, ‘glucose metabolic process’ proteins also constitute a significant fraction (∽15%) of the A-sector, possibly reflecting the important role of glycolysis in generating precursors for amino acid biosynthesis.

The U-sector consists of proteins that are not up-regulated by any of the growth limitations. More than one-third of the U-sector is accounted for by the term ‘cellular amino acid biosynthetic process’. The categories of proteins associated with this term are primarily related to cysteine, methionine, and tryptophan biosynthesis (Supplementary Table S5). This is not surprising for cysteine and methionine synthesis since sulfur is not limited in any of the growth limitations imposed here. The same logic applies to the case of tryptophan synthesis enzymes because tryptophan biosynthesis is not directly affected by the particular mode of A-limitation that was applied (trans-amination, see Supplementary Fig S2), nor by the other two limitations. The second abundant term of the U-sector is ‘purine ribonucleotide biosynthetic process’, which is again not targeted by our mode of A-limitation. The third term is ‘regulation of translation’, where a single ribosomal protein RpsA accounts for the majority of the mass fraction. It is surprising that unlike most of other ribosomal proteins, RpsA was not grouped into the R-sector. This is likely a misclassification due to statistical fluctuation. In summary, the U-sector is composed of enzymes that make up a diverse group of building blocks including some amino acids and purine ribonucleotides, with the common trait that they were not specifically limited by the three growth limitations tested.

The major term of the S-sector is ‘carbohydrate metabolic process’, revealing the sector's role in central metabolism- and energy-related activities. The other two terms are ‘response to stress’ and ‘organic substance transport’. These terms suggest the possible ‘multiple-purpose’ nature of the S-sector proteins that are mobilized in response to starvation conditions via either C- or A-limitation. This notion is best illustrated by the term ‘organic substance transport’, consisting mostly of transporters for peptides and amino acids which can clearly be used to counteract both C- and A-limitations (Supplementary Table S5). The interpretation of the O-sector is less obvious, with the top three terms as ‘macromolecule biosynthetic process’, ‘small molecule biosynthetic process’, and ‘organic substance transport’, reflecting diverse activities of proteins in this sector. Due to the way the O-sector is defined (it results from lumping together three small groups), it is likely that it also includes proteins with weak growth rate dependencies.

In summary, the GO analysis reveals that the R-sector consists mostly of the translational machinery, the C-sector engages in carbon scavenging and saving, the A-sector makes nitrogen-containing building blocks consisting mostly of amino acids, and the U-sector produces other building blocks including sulfur-containing amino acids and purine nucleotides. In exponentially growing cells, these coarse-grained enzymes carry steady fluxes of biomass. As illustrated in Fig4B, these four metabolic fluxes are denoted as *J*_*R*_, *J*_*C*_, *J*_*A*_, and *J*_*U*_, respectively, representing a coarse-grained metabolism. The S-sector shares functions with both the C- and A-sectors, thus carrying both *J*_*C*_ and *J*_*A*_ fluxes.

### Flux matching

The growth rate dependences of the proteome sectors shown in Fig3 are well described by linear relations (Supplementary Table S4). Closer scrutiny of the data and the fits in Fig3 suggests additional simplicity in the structure of the responses. In particular, the downward responses in Fig3 (positive slopes) are similar for each sector, and such responses are referred to as ‘general’ responses as they are not distinguishable between at least two different modes of limitations. On the other hand, the upward response of each of the C-, A-, and R-sectors is specific to only the C-, A-, and R-limitation, respectively, and such a response is referred to as a ‘specific’ response. The only exception is the S-sector, which has similar upward responses to both C- and A-limitations, and the O-sector, which is essentially growth rate-independent. These features suggest that there is a fundamental principle underlying the proteome response to environmental challenges.

As summarized in Fig4B, the GO analysis provides a strong motivation to construct a quantitative flux model for the growth rate dependence of the fluxes associated with each of the proteome sectors. Based on the analysis of the data in Fig3, the proteome is partitioned into six sectors, or ‘coarse-grained enzymes’, *φ*_*σ*_, each of which is comprised of a basal level, *φ*_*σ*,0_, and a growth rate-dependent component, *Δφ*_*σ*_(*λ*), that is, 

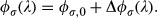
1

In our flux model, we make the central assumption that the flux processed by a proteome sector *σ*,*J*_*σ*_, is proportional to the growth rate-dependent component of the corresponding proteome fraction, Δ*φ*_*σ*_, that is, 

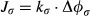
2where *k*_*σ*_ is a coarse-grained kinetic coefficient describing the efficiency of the metabolic sector *σ*. The model is an extension of a similar model proposed in a previous work based on the growth rate dependences of a few reporter genes (You *et al*, 2013). The flux of a sector can be defined as the sum of the metabolic products that flow out from the terminal enzymes per unit time, multiplied by a stoichiometric factor that reflects the composition of the material. For the collection of enzymes that we term the R-sector, the flux is clearly the proteins translated by ribosomes, while for the A-sector, it is largely amino acids. Some proteins, such as those involved in chemotaxis, do not directly handle flux in batch culture but are nonetheless coregulated as part of the C-sector, presumably reflecting their role in facilitating carbon flux in *E. coli*'s natural environment. As shown below, the model comprising of equations (1, 2) can quantitatively account for all of the observations summarized in Fig3.

The ‘downward’ general responses in Fig3 can be exemplified by the R-sector, where the total protein synthesis flux through the ribosomes is given by *J*_*R*_. The R-sector fraction of the proteome (*φ*_*R*_) is given by 

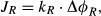
3where *k*_*R*_ is the corresponding enzyme kinetic parameter (given by the peptide elongation rate (Scott *et al*, 2010)). In combination with the stoichiometric requirement of the flux for cell growth, *c*_*R*_ · *J*_*R*_ = *λ*, where *c*_*R*_ is the stoichiometric coefficient (Varma & Palsson, 1994), the growth rate-dependent proteome fraction for the R-sector is given by 

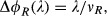
4where *ν*_*R*_ = *k*_*R*_*c*_*R*_ is an effective rate constant for the R-sector. Upon applying the C- or A-limitation, the peptide elongation is not affected, *ν*_*R*_ is constant and equation 4 describes a linear relation between *φ*_*R*_ and *λ*, which is the ‘general response’. Note that this model explicitly predicts identical general responses for the R-sector under C- and A- limitations (equation S1 of Supplementary Table S6), in good agreement with the data of Fig3.

Similarly, for the U-sector: 

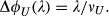
5

The downward lines of the U-sector in Fig3 are produced by equation 5 as long as none of the growth limitations affects the value of *ν*_*U*_, and none of the metabolic processes catalyzed by the U-sector is affected. Thus, the model predicts identical general responses for the U-sector under C-, A-, and R- limitations (equation S2 of Supplementary Table S6), which is consistent with the data in Fig3.

The responses of the C-, A-, and S- sectors are more complex since the S-sector is composed of proteins that provide both *J*_*C*_ and *J*_*A*_ fluxes (Fig4). This effect is modeled by considering two lists of proteins, called 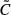 and 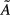, each responding specifically to C- and A-limitation, respectively. We apply the same linear relation between proteome fractions 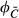, 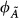 and with the fluxes, that is, 

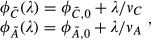
Then S-sector proteins are composed of those proteins that are common to both 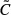 and 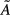, while C- and A-sector proteins are those unique in 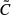 and 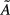, respectively (see Supplementary Fig S12). This is modeled as the follows, 

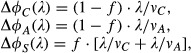
6with *f* being the fraction of 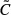
- and 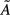-sector proteins that are in common (see Supplementary Fig S12). These relations describe the general responses of the C-, A-, and S-sectors (equations S3–S5 of Supplementary Table S6), for growth limitations that do not affect *v*_*C*_ or *ν*_*A*_. Note that in a previous proteome partition model (You *et al*, 2013) based on measurements of a few reporter genes, the hypothesized C- and A-sectors correspond respectively to the 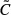- and 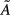-sectors here, whereas the possibility of the S-sector was not anticipated. Finally, we assume the existence of a growth rate-independent sector and identify it with the O-sector, that is, *φ*_*O*_(*λ*) = *φ*_*O*,0_ (equation S6 of Supplementary Table S6) with 

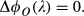
7

### Constraint of finite proteome resources

A striking result of this flux model is that the ‘specific’ upward responses of the C-, A-, R-, and S-sectors in Fig3 can also be produced by equations 4-7, without introducing any additional parameters. For example, under R-limitation, the value of *ν*_*R*_ changes in response to the limitation, and consequently, the growth rate dependence of *φ*_*R*_ can no longer be obtained from equation 4. However, *Δφ*_*R*_(*λ*) can be obtained from the important constraint 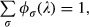 or equivalently 

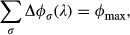
8where 
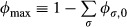
. Since under R-limitation only *ν*_*R*_ is reduced, all other sectors still follow the general responses. Using equations 4-8, the expression for the specific response of the R-sector becomes: 

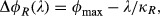
9with 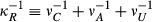 (equation S7 of Supplementary Table S6). Note that both parameters appearing in equation 10 are determined completely in terms of the parameters already introduced, and an important feature of the flux model is that there is no additional parameter for the specific responses once the general responses are established. In a similar manner, the specific responses of the C-, A-, and S-sectors are obtained in terms of *ϕ*_max_ and the *ν*s, with no additional parameters; see equations (S8–S10) of Supplementary Table S6 with derivation given in Supplementary Fig S12.

**Figure 5:**
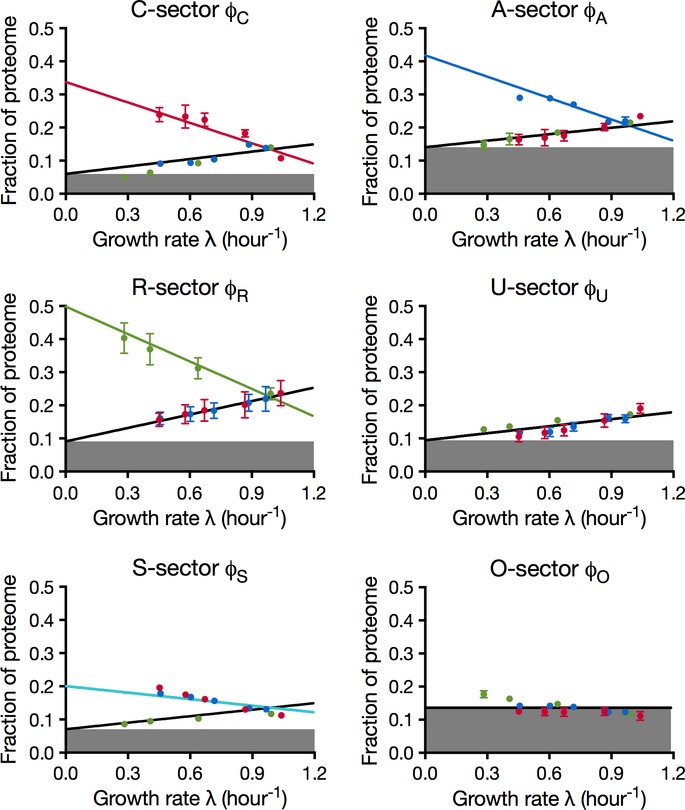
Performance of the proteome-based flux model The data points are identical to those in Fig3. The lines here are the result of a global fit to the predictions of the flux-based proteome model (Supplementary Table S6). The growth rate-independent component of each sector (*φ*_*σ*,0_) is represented as the height of the filled area in the corresponding plot. See Supplementary Table S7 for parameters of the fitted lines.

In summary, the linear equations in Supplementary Table S6 describe the prediction of the simple flux model (equations 1, 2) on the partitioning of the proteome as a function of growth rate under the three different modes of growth limitation. Although the model contains only 10 adjustable parameters, the quality of the fit of the model to the data (lines in Fig5; Supplementary Table S7) is comparable to the 24-parameter fit for each individual response (Fig3; Supplementary Table S4).

### Two global parameters

**Figure 6:**
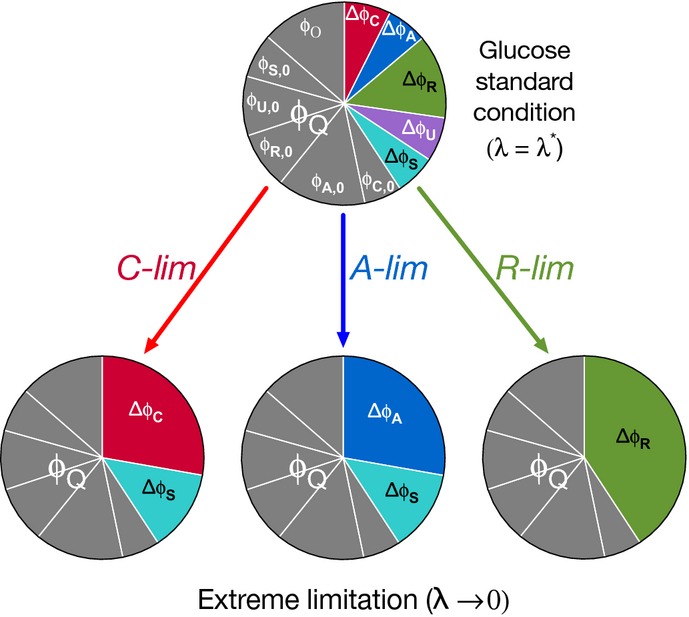
Representation of the proteome responses under extreme growth limitations and interpretation of the model parameters The growth rate-independent component of the protein is represented as *ϕ*_*Q*_ (the entire gray area), which is composed of the growth rate-independent components of the C-, A-, R-, U-, and S-sectors, and the O-sector. See Supplementary Table S7 for their values. The growth rate-dependent part of a sector *σ* is labeled as *Δφ*_*σ*_, distinguished by the different colors. The colored wedges in the top pie chart show the sizes of these sectors, *Δφ*_*σ*_(λ*), under the glucose standard condition (with growth rate λ*). Their values are as follows: ^2^*φ*_*C*_ = 0.07, ^2^*φ*_*A*_ = 0.06, ^2^*φ*_*R*_ = 0.13, ^2^*φ*_*U*_ = 0.07, and ^2^*φ*_*S*_ = 0.06. The pie charts at the bottom show the sizes of these sectors under the three modes of growth limitations in the extreme limit *λ* → 0. Theses sizes are governed by two parameters, *φ*_max_ = 1 − *φ*_*Q*_ and *f* ≈ 0.32.

The straightforward meanings of the remaining 10 parameters are illustrated by the cartoon in Fig6. The top pie chart in Fig6 represents the proteome fractions for the sectors under the glucose standard condition, with the growth rate-independent fraction of the proteome, *φ*_*Q*_ (gray area in the top pie chart) being 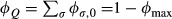. The growth rate-dependent component includes the remainder of every sector, shown as colored wedges, whose proteome fractions make up the rest of the pie, *φ*_max_.

The bottom three pie charts in Fig6 describe the responses of the proteome to each of the three modes of growth limitation in the extreme case *λ* → 0 according to the model. Under extreme R-limitation, the R-sector fraction Δ*φ*_*R*_ approaches *φ*_max_, while under C-limitation, *φ*_max_ is partitioned into Δ*φ*_*C*,max_ = (1 − *f*) · *φ*_max_ and Δ*φ*_*S*,max_ = *f* · *φ*_max_, and under A-limitation, *φ*_max_ is partitioned into Δ*φ*_*A*,max_ = (1 − *f*) · *φ*_max_ and Δ*φ*_*S*,max_ = *f* · *φ*_max_. Note that the growth rate-dependent responses Δ*φ*_*σ*_(*λ*) are described effectively by only two parameters, *φ*_max_ and *f*. *φ*_max_ provides a cap on the magnitude of the growth rate-dependent component of each sector. The best-fit value, *φ*_max_ ≈ 40%, is in quantitative agreement with previous estimates based on the ribosomal content (Scott *et al*, 2010) and a few reporters (You *et al*, 2013).

### Model prediction and testing

**Figure 7:**
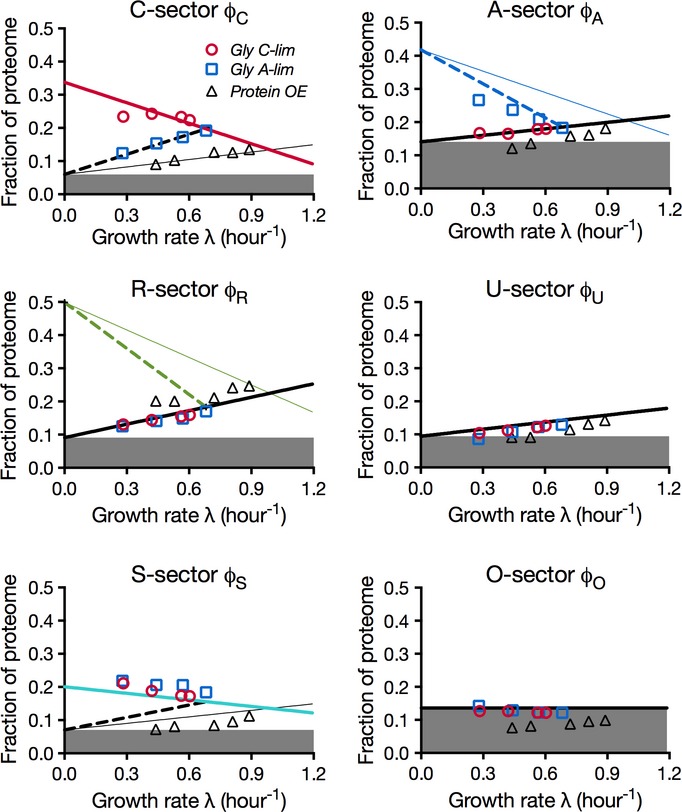
Proteome fractions under growth limitations with respect to the glycerol standard condition, and under growth limitation by expressing useless proteins Proteome fractions *φ*_*σ*_ for C- and A-limitation under glycerol standard condition are shown as the red and blue circles, respectively, for each of the six sectors; see Supplementary Table S9 for values. All thick lines are model predictions for responses under the glycerol standard condition. Thick solid lines describe responses which are predicted to be unchanged between the glucose and glycerol standard conditions, because these lines do not involve the parameter *ν*_*C*_, which has a new value for the new standard condition according to the model. Thick dashed lines describe responses which are predicted to be unique for the glycerol standard condition, due to their dependence on the value of parameter *ν*_*C*_. See Supplementary Fig S12 describing the dashed lines for the C-, A-, R-, and S-sectors. For comparison, the four respective proteome responses under glucose standard condition are also shown as thin solid lines. All solid lines are from Fig5. Note that the new value of *ν*_*C*_ is determined from the growth rate of cells in glycerol standard condition (Supplementary Table S7). Thus, all predictions for the glycerol standard condition were generated with no adjustable parameters. Proteome fractions under growth limitation by LacZ overexpression are shown as the black triangles for the six sectors; see Supplementary Table S9 for values.

Among the 10 parameters of the model, the four values of *ν*_*σ*_s are dependent on the growth medium, while the *φ*_*σ*,0_s as well as the constant *f* ≈ 0.32 are expected to be medium independent for a given strain. All of the data described so far (summarized in Fig3) were obtained using glucose minimal medium as the standard condition (with the *ν*_*σ*_s taking on the values 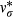), with each mode of growth limitation corresponding to varying one of the *ν*_*σ*_s away from 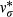. The proteome flux model also makes explicit predictions on the response of the proteome under combinatorial modes of growth limitation, corresponding to varying multiple *ν*_*σ*_s. The effect of varying multiple *ν*_*σ*_s can be treated as simply repeating the single mode of growth limitations for different standard conditions. This prediction was tested by repeating the proteomic experiments under C- and A-limitations using a different standard condition, for growth in the glycerol minimal medium (Supplementary Table S8). Compared to the standard condition with glucose minimal medium, the glycerol minimal medium should differ by only the value of *ν*_*C*_, which is fixed by the growth rate for the glycerol standard condition (Supplementary Table S7). Using this new value of *ν*_*C*_, together with the values of the other nine parameters obtained from the glucose data, the model describes the new data remarkably well (Fig7; Supplementary Table S9). Thus, the model can describe experiments in different standard conditions, an important benchmark for its ability to capture proteome responses to combinatorial limitations.

As a further test of the model, and specifically, the notion of general response, we studied another way of growth limitation by expressing useless proteins (Scott *et al*, 2010), which reduces the proteome fraction available for the six sectors, that is, it reduces *φ*_max_. This was achieved by using a LacZ-overexpressing strain (NQ1389) grown on glucose minimal medium (Supplementary Table S8). Since the applied growth limitation does not single out any metabolic sector, our model predicts that every proteome sector except the O-sector should exhibit general responses (equations 4–7), with the same slope *ν*_*σ*_s as those obtained from C-, A-, and R-limitations. In addition, the y-offset of every sector, *φ*_*σ*,0_ in equation 1, should remain unchanged. (Mathematically, we expect only *φ*_max_ to be reduced while all other nine model parameters to remain constant.)

The proteome fraction data (Supplementary Table S9) in response to LacZ overexpression are shown as the black triangles in Fig7. We see that there is generally a congruence of the black triangles and the black solid lines (general responses with glucose minimal medium as the standard condition) as predicted, except for the O-sector which also showed substantial reduction as growth rate is decreased. This suggests that perhaps a good share of the proteins that got classified into the O-sector actually exhibit growth rate dependence which were obscured by noise under C, A-, and R-limitations.

## Discussion

Understanding the principles of the global regulation of gene expression is a major goal of systems biology. However, the intricacy of genetic regulatory networks makes this goal difficult to realize through bottom-up analysis. Quantitative measurements of the concentrations of thousands of proteins, mRNA, and metabolites in cells have recently become possible. These techniques invite revealing measurements of the differential composition of the cell as a function of growth condition, as well as a framework capable of describing the resource allocation of the cell. Coarse-graining procedures offer a means to capture the allocation of proteome resources by the cell using such data, without detailed knowledge of thousands of enzyme rates, binding constants, and regulatory relationships.

Here, we measured the quantitative response of ∽1,000 proteins in *E. coli* as cells are progressively limited in three broad manners: limiting carbon uptake through the lactose importer, limiting nitrogen assimilation through the GS-GOGAT pathway, and limiting protein translation using sublethal amounts of chloramphenicol. Analysis of the individual protein concentrations in the three limitation series suggests six distinct sectors of the proteome, and abundance-based GO term enrichment reveals a functional coherence across the enzymes of each sector that is largely orthogonal to the functions of the other sectors. Note that the sectors are revealed by the nature of growth limitations applied and we expect other sectors to emerge under other growth limitations.

### Coarse-grained proteome sectors

During balanced exponential growth, a constant flux of matter from environmental nutrients cascades through the metabolic network of the cell to form biomass. In contrast to bottom-up descriptions of the metabolic network as an object of great complexity (e.g., the KEGG map), our results reveal an enzymatic network that is simply coarse-grained according to the functional grouping of the proteome sectors. Strikingly, the mass fractions of the various proteome sectors increase or decrease in approximately linear fashion with the change in cell growth rate, which serves as a quantitative measure of the degree of the applied limitation. For instance, the C-sector, consisting mostly of carbon catabolic proteins, increases linearly in response to the limitation of carbon influx.

The control of proteome partition is likely orchestrated by sophisticated regulatory networks that integrate information from multiple signaling molecules. Some of these signals are well known, for example, ppGpp directs ribosome synthesis in accordance with the level of amino acid depletion (Ross *et al*, 2013), cAMP-Crp coordinates catabolic protein expression in accordance with the availability of alpha-keto acids (You *et al*, 2013), and the PII/NtrBC system determines the degree of nitrogen assimilation in accordance with the availability of glutamine (Reitzer, 2003). However, many mysteries remain. The coherent response of the proteins in the anabolic sector is well beyond what is known to be controlled by nitrogen regulatory system, and the enzymes for amino acid synthesis and nucleic acid synthesis are clearly distinguished in their responses. Further, a substantial number of proteins are in the S-sector which responds to both C- and A-limitations; yet little similarity can be seen based on their promoter regions. It is possible that major pleiotropic regulators are yet to be discovered or that the roles of some existing pleiotropic regulators are to be reappraised (as has been done recently for cAMP-Crp (You *et al*, 2013), a well-characterized regulator whose function was long thought to be understood). The simple behaviors of the proteome sectors revealed in this work are molecular phenotypes that can be relied upon in future studies to identify the coordinators of such coherent responses. Importantly, a number of high abundance proteins of as yet unknown function reside within the coarse-grained functional sectors we identified.

In recent years, a number of studies have characterized the growth rate dependence of relative mRNA abundance in Baker's yeast under various nutrient-limiting conditions in chemostat (Regenberg *et al*, 2006; Castrillo *et al*, 2007; Levy *et al*, 2007; Brauer *et al*, 2008; Airoldi *et al*, 2009). Brauer *et al*, Castrillo *et al*, and Regenberg *et al* report a large group of mRNA that increase with carbon limitation, and are characterized by the enriched GO terms cellular carbohydrate metabolism, cellular macromolecule catabolism, transport, and response to stress, recalling our C- and S-sectors (Regenberg *et al* normalize their data such that 42 ORFs with negative correlations with the growth rate become growth rate independent. With this in mind, growth rate-independent groups should obtain negative λ dependence.). Notably, Levy *et al* report a decrease in ribosomal protein mRNA synthesis as the cell exits exponential growth, while Airoldi *et al* successfully predict growth rate in *S. cerevisaiae* from the behavior of a few reporter mRNA, which comports with our finding that the majority of proteins change with growth rate in a characteristic fashion (Supplementary Text S4).

### Proteome fraction as a quantitative measure

The similarities mentioned above suggest that the principles we uncover here may be fundamental to metabolism in a variety of cell types. However, we question the effectiveness of using mRNA measurements to infer protein concentrations which protein activities depend on (see Supplementary Text S5). A primary lesson drawn from the linear relation between the ribosome and growth rate is that cells operate at saturated translational capacity (Maaloe, 1979; Scott *et al*, 2010). As a result, mRNA and protein levels are not expected to couple tightly due to mRNA competition for the limited number of ribosomes, a phenomenon also known as ribosome queueing (Mather *et al*, 2013). Indeed, studies that compare mRNA and protein levels from *H. sapiens* to *E. coli* generally report poor correlations that fail to provide predictive power (Pearson correlation *r*_*p*_ ≈ 0.5) (Maier *et al*, 2009; Taniguchi *et al*, 2010; Vogel *et al*, 2010). Further, mRNA levels are typically reported as a fraction of total fluorescence intensity, that is, as a fraction of total mRNA, without keeping track of the change in total mRNA concentration across different growth conditions [e.g., cell volume can change many fold under nutrient limitation (Schaechter *et al*, 1958)], so that mRNA measurements do not necessarily correlate with mRNA concentration (Klumpp *et al*, 2009). Also, coarse graining requires knowledge of absolute concentrations, as can be provided by methods such as quantitative mass spectrometry and ribosome profiling (Li *et al*, 2014; Muntel *et al*, 2014), without which the true cost of gene expression is difficult to quantify. For example, a 50% increase in proteome mass fraction from 20% to 30% should not be compared to a 50% increase from 0.1% to 0.15%.

So far, the cost associated with making proteins has been quantified mostly for useless proteins (Andrews & Hegeman, 1976; Dong *et al*, 1995; Kurland & Dong, 1996; Scott *et al*, 2010; Shachrai *et al*, 2010) and, in some cases, proteins with specific functions (Dekel & Alon, 2005). Several recent studies have found that the protein cost plays a key role in understanding regulatory strategies in metabolism (Molenaar *et al*, 2009; Wessely *et al*, 2011; Flamholz *et al*, 2013). Genomescale computational models that integrate protein resource allocation to existing constraint-based models (e.g., stoichiometry constrained models) have been proposed as a step forward in unraveling intricate relations between growth, metabolism, and gene expression (Goelzer & Fromion, 2011; O'Brien *et al*, 2013). The quantitative data provided in this study will hopefully stimulate further quantitative studies along these directions.

### Open questions on regulatory mechanisms

Though mass spectrometry and ribosome profiling are capable of providing absolute abundances and are thus crucial to coarse-graining analysis of the proteome, there are deeper, regulatory layers to resource allocation (requiring other techniques) that remain unaddressed. As mentioned above, one urgent question raised by our results is the molecular basis for the coordination of genes in the A- and S-sectors, which could be addressed by transcription factor profiling and quantitative metabolomics. Significant action of a riboswitch or small RNA in translational regulation would be invisible to mass spectrometry and ribosome profiling; here, RNA-seq would be an obvious method to use. Finally, growth conditions with significant protein degradation (e.g., during growth transition; Kuroda, 2001) would lead to chronic overestimates of protein level by ribosome profiling and underestimates of translational cost by mass spectrometry. In these conditions, both methods could be rescued by an accurate determination of individual protein degradation rates (e.g., by pulse labeling).

### Principles of resource allocation

Despite the lack of detailed regulatory information, we showed that a phenomenological model that stipulates flux matching in the flow of material between the sectors of the coarse-grained reaction network, along with the constraint of a finite proteome, is sufficient to quantitatively capture the observed sector behavior over a range of growth rates, with only a few parameters. These governing principles imply that the flux through each sector σ is carried by a mass fraction *φ*_*σ*_ whose size is determined by the cost of supplying flux through the given sector under the given mode of growth limitation, which is given by 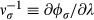. In this way, the cell's proteome management is analogous to the economic concept of ‘division of labor’ (Hayek, 1945), with finite capital allocated according to an effective pricing system given by the *ν*_*σ*_s (Lovell, 2004; Mankiw, 2011). When a sector is specifically challenged, such as the C-sector under carbon uptake limitation, the price to carry flux through the C-sector, 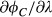, is increased while the price to carry flux through the other sectors remains the same. This requires an increased investment of capital, for example, proteome fraction, to carry the requisite flux *J*_*C*_. These results provide a quantitative framework to buttress the common depictions of cell metabolism, putting the conceptual device of supply and demand on rigorous footing.

### Possible origins of the growth rate-independent sectors

While the growth rate-dependent components (the ^2^*φ*_*σ*_s) closely follow economic principles, much of the growth rate-independent component (*ϕ*_*Q*_) comprises of offsets of the identified proteome sectors, that is, the *φ*_*σ*,0_s as shown in Fig6. A variety of possible mechanisms have been proposed for the R-sector offset: A favorite early model was the existence of a fraction of non-translating ribosomes; see Scott *et al* (2010) and references there. Zaslaver *et al* (2009) obtained an offset from *ad hoc* optimization scheme, while Klumpp *et al* (2013) proposed another mechanism based on the growth rate dependence of tRNA. For the offsets of the metabolic sectors, the simplest mechanism could be the biophysical difficulty to tightly repress gene expression, since a zero offset requires protein synthesis to be completely turned off at zero growth rate. It was shown that abundance in the growth rate-independent sector directly diminishes the maximal growth rate (Scott *et al*, 2010). However, this seemingly wasteful allocation of proteome resources may serve a purpose that transcends the simple economics of steady-state growth. For example, keeping substantial offsets on hand may help bacteria adapt more quickly to varying nutrient conditions (Kjeldgaard *et al*, 1958; Koch & Deppe, 1971; Dennis & Bremer, 1974). Competing considerations may well arise at very slow growth, or in starvation conditions, adding to the principles of proteome management revealed by this work.

This work specifically examined cells kept at moderate growth rates, and it is unclear when or whether the observed linear relations cease to hold at slower growth. We emphasize that our goal here is to describe the data by a minimal model with predictive power. We do not rule out nonlinear generalizations of the model presented here.

## Materials and Methods

Detailed bacterial growth protocol, procedures used for strain construction, total RNA and total protein measurements, and β-galactosidase assay are described in the Supplementary Text S1.

### Growth conditions

All growth media used in this study were based on the MOPS-buffered minimal medium used by Cayley *et al* (1989) with slight modifications. See Supplementary Text S1 for the composition of the base medium. The lactose minimal medium and the glucose minimal medium had 0.2% (w/v) lactose and 0.2% (w/v) glucose in addition to the base medium, respectively. For the C-limitation growth, 1 mM isopropyl β-d-1-thiogalactopyranoside (IPTG) and various concentrations (0–500 μM) of the inducer 3-methylbenzyl alcohol (3MBA) were added to the lactose minimal medium. For the A-limitation growth, various concentrations of IPTG (30–100 μM) were added to the glucose minimal medium. Various concentrations of chloramphenicol (0–8 μM) were used for the glucose minimal medium for the R-limitation growth. For the C-limitation growth with NQ399, 0.2% (w/v) glycerol was added to the MOPS base medium, in addition to 1 mM IPTG and various concentrations (0–500 μM) of 3MBA. The same glycerol minimal medium with no 3MBA and various amounts of IPTG was used for the A-limitation on glycerol.

### ^15^N-labeled proteomic mass spectrometry

#### Sample preparation

1.8 ml of cell culture at OD_600_ = 0.4∽0.5 during the exponential phase of the experimental culture (defined above) was collected by centrifugation. The cell pellet was re-suspended in 0.2 ml water and fast-frozen on dry ice.

Aliquot of the ^15^N reference cell sample (or labeled cell sample) was mixed with each of the ^14^N cell samples (or non-labeled cell samples), which contained the same amount of proteins. Each aliquot of the ^15^N samples contained about 100 μg of proteins. Each of the ^14^N cell samples also contained about 100 μg proteins. For each mode of growth limitation, a ^15^N reference cell sample was made in such a way that it contained cell samples from both the fastest and slowest growth conditions under that growth limitation. The mixed reference is used to avoid the composition of proteins in the reference cell sample be biased by a particular growth medium.

Proteins were precipitated by adding 100% (w/v) trichloroacetic acid (TCA) to 25% final concentration. Samples were let stand on ice for a minimum of 1 h. The protein precipitates were sped down by centrifugation at 16,000 *g* for 10 min at 4°C. The supernatant was removed and the pellets were washed with cold acetone. The pellets were dried in a Speed-Vac concentrator.

The pellets were dissolved in 80 μl 100 mM NH_4_HCO_3_ with 5% acetonitrile (ACN). Then, 8 μl of 50 mM dithiothreitol (DTT) was added to reduce the disulfide bonds before the samples were incubated at 65°C for 10 min. Cysteine residues were modified by the addition of 8 μl of 100 mM iodoacetamide (IAA) followed by incubation at 30°C for 30 min in the dark. The proteolytic digestion was carried out by the addition of 8 μl of 0.1 μg/μl trypsin (Sigma-Aldrich, St. Louis, MO) with incubation overnight at 37°C.

The peptide solutions were cleaned by using the PepClean® C-18 spin columns (Pierce, Rockford, IL). After drying in a Speed-Vac concentrator, the peptides were dissolved into 10 μl sample buffer (5% ACN and 0.1% formic acid).

#### Mass spectrometry

The peptide samples were analyzed on an AB SCIEX TripleTOF® 5600 system (AB SCIEX, Framingham, MA) coupled to an Eksigent NanoLC Ultra® system (Eksigent, Dublin, CA). The samples (2 μl) were injected using an autosampler. The samples were first loaded onto a Nano cHiPLC Trap column 200 μm × 0.5 mm ChromXP C18-CL 3 μm 120 Å (Eksigent) at a flow rate of 2 μl/min for 10 min. The peptides were then separated on a Nano cHiPLC column 75 μm × 15 cm ChromXP C18-CL 3 μm 120 Å (Eksigent) using a 120-min linear gradient of 5–35% ACN in 0.1% formic acid at a flow rate of 300 nl/min. MS1 settings: mass range of m/z 400–1,250 and accumulation time 0.5 s. MS2 settings: mass range of m/z 100–1,800, accumulation time 0.05 s, high sensitivity mode, charge state 2–5, selecting anything over 100 cps, maximal number of candidate/cycle 50, and excluding former targets for 12 s after each occurrence.

#### Protein identification

The raw mass spectrometry data files generated by the AB SCIEX TripleTOF® 5600 system were converted to Mascot generic format (mgf) files, which were submitted to the Mascot database searching engine (Matrix Sciences, London, UK) against the *E. coli* SwissProt database to identify proteins. The following parameters were used in the Mascot searches: maximum of two missed trypsin cleavage, fixed carbamidomethyl modification, variable oxidation modification, peptide tolerance ± 0.1 Da, MS/MS tolerance ± 0.1 Da, and 1+, 2+, and 3+ peptide charge. All peptides with scores less than the identity threshold (*P* = 0.05) were discarded.

#### Relative protein quantitation

The raw mass spectrometry data files were converted to the .mzML and .mgf formats using conversion tools provided by AB Sciex. The .mgf files were used to identify sequencing events against the Mascot database. Finally, spectra for peptides from the Mascot search were quantified using least-squares Fourier transform convolution implemented in house (Sperling *et al*, 2008). Briefly, data were extracted for each peak using a retention time and m/z window enclosing the envelope for both the light and heavy peaks. The data are summed over the retention time, and the light and heavy peaks amplitudes are obtained from a fit to the entire isotope distribution, yielding the relative intensity of the light and heavy species. The ratio of the non-labeled to labeled peaks was obtained for each peptide in each sample.

The relative protein quantitation data for each protein in each sample mixture was then obtained as a ratio by taking the median of the ratios of its peptides. No ratio (i.e., no data) was obtained if there was only one peptide for the protein. The uncertainty for each ratio was defined as the two quartiles associated with the median. To filter out data with poor quality, the ratio was removed for the protein in that sample if at least one of its quartiles lied outside of 50% range of its median; Furthermore, ratios were removed for a protein in all the sample mixtures in a growth limitation if at least one of the ratios has one of its quartiles lying outside of the 100% range of the median.

Since the ratios are all defined relative to the same reference sample, they represent the relative change of the expression of the protein across all the non-labeled cell samples and are referred as ‘relative expression data’.

#### Absolute protein quantitation

The spectral counting data used for absolute protein quantitation were extracted from Mascot search results. For our ^15^N and ^14^N mixture samples, only the ^14^N spectra were counted. The absolute abundance of a protein was calculated by dividing the total number of spectra of all peptides for that protein by the total number of ^14^N spectra in the sample.

#### Data availability

The mass spectrometry proteomics data have been deposited to the ProteomeXchange Consortium (Vizcaíno *et al*, 2014) via the PRIDE partner repository with the dataset identifier PXD001467. Plots of individual proteins under the three growth limitations are available as in Supplementary Dataset S1.

### Data analysis

#### Expression matrices

For each of the growth limitation, the relative expression data can be represented in the form of an expression matrix. For example, under C-limitation, the expression matrix is *N* × 5, where *N* is the number of proteins and 5 is the number of growth rates. To focus on proteins with high-quality data, a protein entry (i.e., a row in the matrix) is removed if the number of nonempty data elements for the protein is < 3. As described in the main text, the sizes of the three final expression matrices are 856 × 5, 898 × 5, and 756 × 4, respectively, for the C-, A-, and R-limitation.

#### Scaling of the expression matrices

Because different ^15^N reference samples were used for different modes of growth limitation, it is convenient to rescale the relative expression data, so that for each protein the value is set to 1 under a ‘glucose standard condition’, which was the condition of WT NCM3722 cells growing in glucose minimal medium. Note that for both the A-limitation and R-limitation, the unlimited condition (or the fastest growth condition) was exactly the standard condition. For the C-limitation, however, the standard condition was not one of the growth conditions. The growth rate of the standard condition was between the fastest growth condition (with a doubling time of 40 min) and the second fastest growth condition (with a doubling time of 48 min). Assuming protein expression follows a linear relation under C-limitation, the expression level for the standard condition was determined by extrapolating the expression levels for the two neighboring growth rates.

#### Clustering analysis of the expression data

After scaling, the three expression matrices were merged into a 1,053 × 14 expression matrix, with 14 ( = 5 + 5 + 4) for the total number of growth conditions and 1,053 for the total number of unique proteins. The pairwise distance (*d*) used for clustering was defined as *d* = 1 − *ρ*, where *ρ* is the Pearson correlation: 

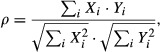
where *X*_*i*_ and *Y*_*i*_ are the log2-transformed relative expression data for two proteins at same growth condition *i*. The Matlab (The Mathworks, Natick, MA) function ‘linkage.m’ was used to carry out a hierarchical clustering with the option of ‘unweighted average distance’. The results were written in the format for the cluster viewing software Java TreeView (Saldanha, 2004).

#### Measure of the quality of a fit

We used the coefficient of determination, R^2^ as a measure of fit quality. Assuming a dataset has values *y*_*i*_, and the predicted values *f*_*i*_ based on the fit, *R*^2^ is defined as 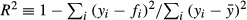, where 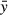 is the mean of the values *y*_*i*_. The value of *R*^2^ ranges from 0 to 1, with larger number meaning high quality of fit. For a linear fit, *R*^2^ indicates the degree of linearity of the data.

## Supporting Information

Supplementary Dataset S1

Supplementary Code

Review Process File
