# Supplementary material for: Quantitative proteomic analysis reveals a simple strategy of global resource allocation in bacteria
Source: Mol Syst Biol. 2015 Feb 12;11(2):784. doi: 10.15252/msb.20145697 (PMC4358657; doi:10.15252/msb.20145697)
Supplement: Supplementary file 1 [file msb0011-0784-sd1.doc]

**EquationSupplementary items**

Quantitative proteomic analysis reveals a simple strategy of global resource allocation in bacteria

Table of Contents

Supplementary Figures [2](#__RefHeading___Toc279923107)

Supplementary Tables [16](#__RefHeading___Toc279923108)

Supplementary Text 1 [25](#__RefHeading___Toc279923109)

Supplementary Text 2 [29](#__RefHeading___Toc279923110)

Supplementary Text 3 [32](#__RefHeading___Toc279923111)

Supplementary Text 4 [42](#__RefHeading___Toc279923112)

Supplementary Text 5 [44](#__RefHeading___Toc279923113)

Supplementary Dataset [47](#__RefHeading___Toc279923114)

Supplementary Code [48](#__RefHeading___Toc279923115)

References [49](#__RefHeading___Toc279923116)

**Equation**

# Supplementary Figures

**
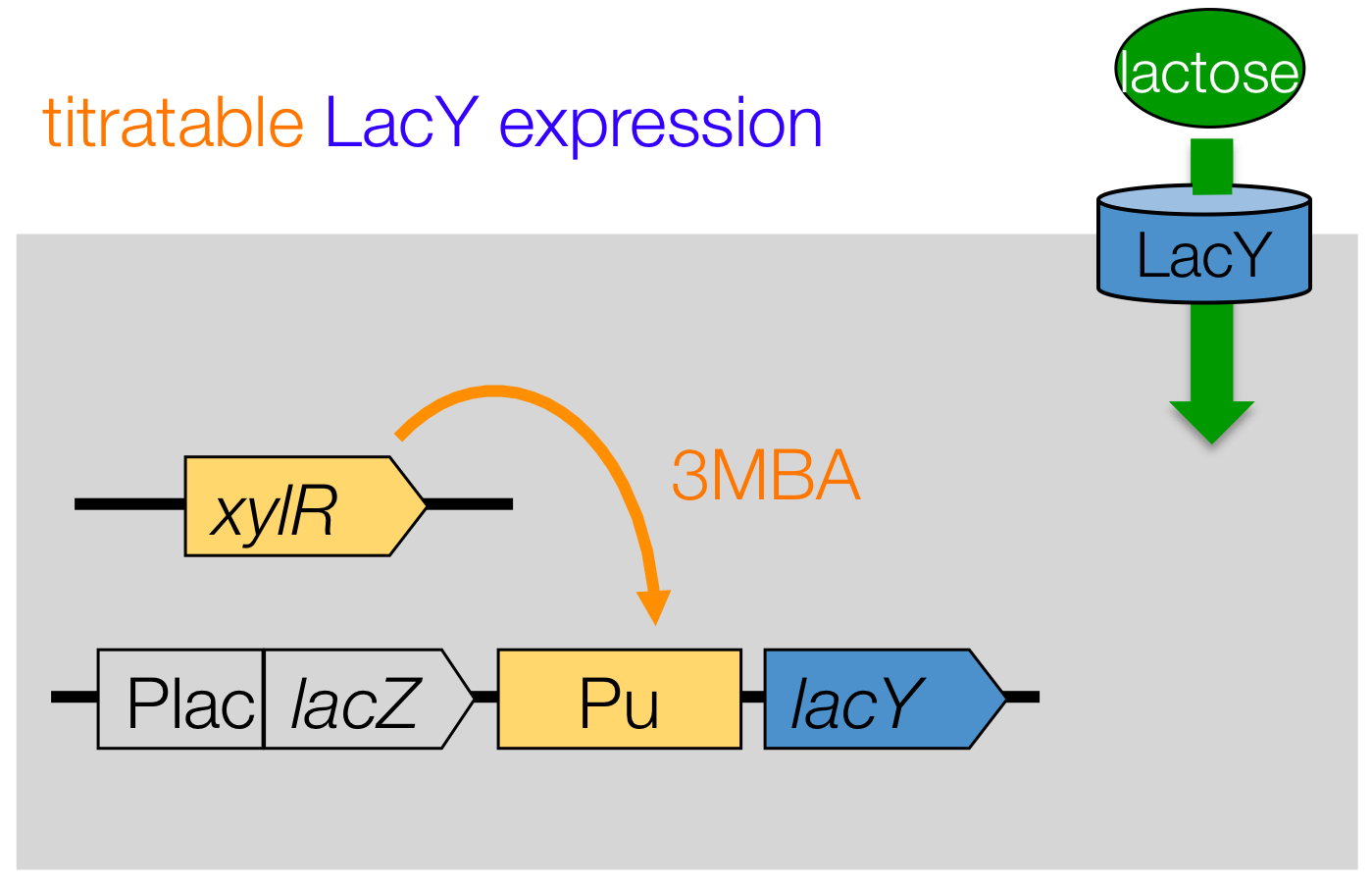
**

**Figure S1. C-limitation by titrating lactose uptake.**

LacY (or lactose permease) is the only transporter that allows *E. coli* to grow on lactose as the sole carbon source. We therefore sought to control lactose uptake by titrating the expression of LacY using the strain NQ381 (Yo*u et* al, 2013). The strain was constructed by inserting a titratable P*u* promoter from *Pseudomonas putida* between the *lacZ* stop codon and *lacY* start codon. The expression of the P*u* promoter is activated by the regulator XylR upon induction by 3-methylbenzyl alcohol (3MBA). Strain NQ381 was grown in lactose minimal medium, supplemented with 1 mM IPTG and various levels of 3MBA (0-500 µM) to stimulate XylR and titrate the expression of LacY.


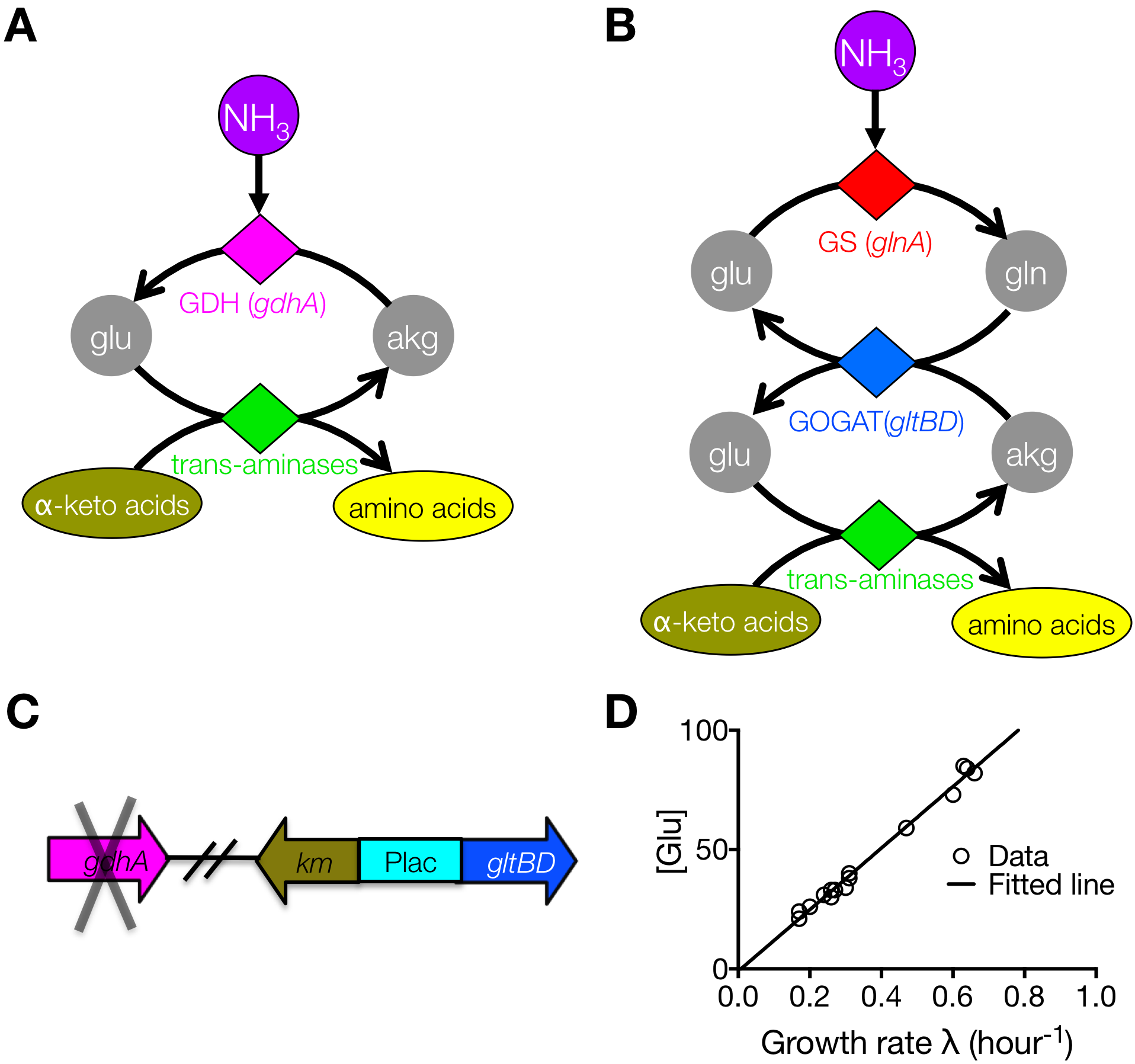


**Figure S2. A-limitation by titrating ammonia assimilation.**

To impose A-limitation, we constructed the strain NQ393 whose capacity to assimilate ammonium as the sole nitrogen source can be varied in graded manners.

A and B illustrate the two known pathways for the assimilation of ammonium in *E. coli*. In pathway A, ammonium is fixed onto alpha-ketoglutarate (akg) via the enzyme glutamate dehydrogenase (GDH, purple diamond, encoded by *gdhA*) to form glutamate (glu), which subsequently trans-aminates (green diamond) one of many alpha-keto acids (light blue oval) to form amino acids (yellow oval), regenerating akg in the process. In pathway B, the overall process is the same except that GDH is replaced by two enzymes, glutamine synthetase (GS, red diamond, encoded by *glnA*) and glutamate synthase (GOGAT, blue diamond, encoded by the *gltBD* operon). In this pathway, ammonium is first assimilated into glutamine (gln) and then passed on to glu. Note that among the biosynthetic pathways of the 20 amino acids, only the tryptophan pathway does not involve transamination reaction.

C In strain NQ393 the gene *gdhA* is deleted and the promoter of *gltBD* is replaced by the P*lac* promoter, so that pathway A is broken and ammonium assimilation must proceed by pathway B. See Supplementary Text S1 for details of strain construction. Strain NQ393 was grown on glucose minimal medium, supplemented with various concentrations of IPTG (30-100 µM) to titrate the expression of GOGAT. A strain of similar purpose but with disabled pathway B (by deleting *gltB*) and titratable promoter of *gdhA* was used in (Yo*u et* al, 2013).

D The intracellular glu pool concentration ([Glu]) increases linearly with the growth rate. The data are for a strain that has the native *lac* promoter replaced with the *glnA* promoter but otherwise is identical to NQ393. It suggests that the growth reduction of this strain and also of NQ393 is due to limitation in glu, which presumably directly affects amino acid synthesis via trans-amination (A). The method for measuring the glu pool was described in Okano et al. 2010 (Okan*o et* al, 2010).


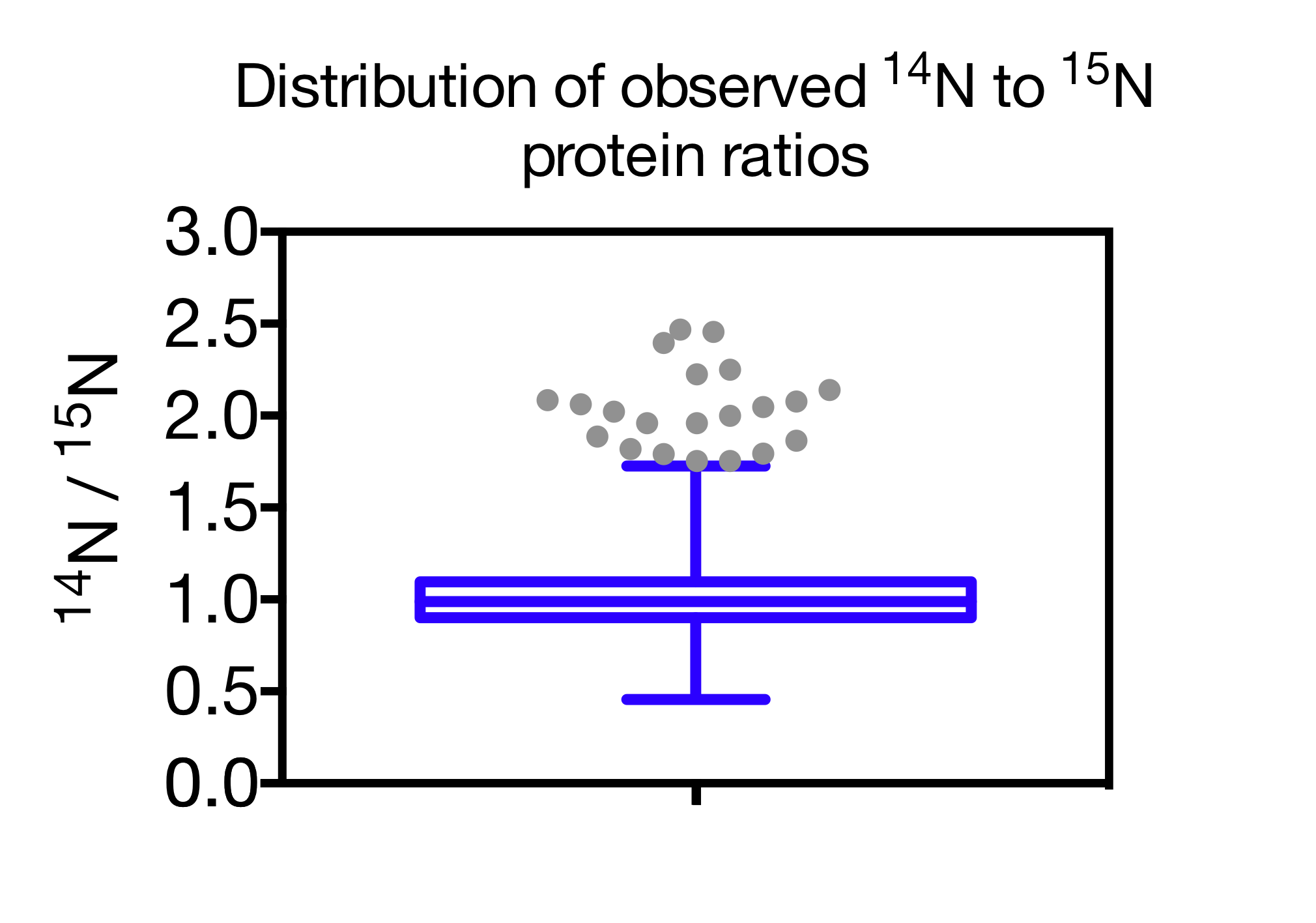


**Figure S3. The overall precision of the method of quantifying relative protein expression levels with mass spectrometry.**

To measure the overall precision of the relative protein quantification method using mass spectrometry, we focus on the control sample which consists of 1:1 mixture of ^15^N and ^14^N samples, where each protein has an expected ^15^N to ^14^N ratio of 1. Above is the distribution of the observed ^14^N over ^15^N ratios for 638 proteins in the control sample. The box includes data points between first quartile (0.906) and the third quartile (1.114), with the line in the center of the box representing the median value (0.998). The upper and low hinges represent the maximum and minimum data points, excluding 21 outliers. The outliers are shown in gray points and are defined as points that are at least 3×(3^rd^ quartile – 1^st^ quartile) away from either the 1^st^ quartile or the 3^rd^ quartile. The standard deviation for all the data points except the outliers is calculated to be 0.179, or about 18%, which is taken as the precision of the method.


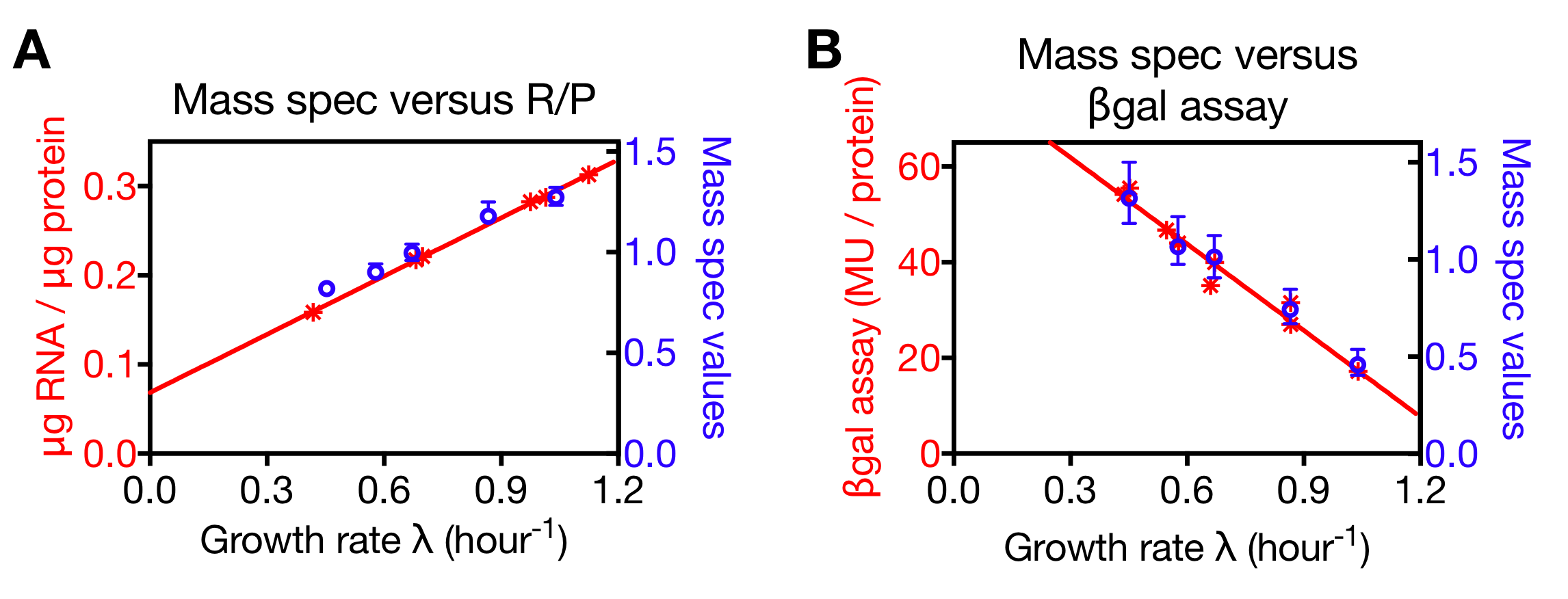


**Figure S4: Comparison of the relative mass spectrometry method to traditional biochemical methods.**

A Comparison between mass spectrometry data and the measurements of the total RNA to total protein ratio (R/P). The ratio between total RNA and total protein is well established as a good proxy (with a constant converting factor) for ribosome content, and has a linear relation with growth rate for nutrient-limited growth (Schaechte*r et* al, 1958; Maaloe, 1979; Scot*t et* al, 2010). The red dots are R/P data for cells grown on various carbon sources. The blue circles are the relative change of ribosomal proteins under C-limitation as detected by mass spectrometry. The mass spectrometry values for the ribosomal proteins were taken as the medians of the 52 ribosomal proteins detected. The error associated with each value was taken as the quartiles.

B Comparison between mass spectrometry data (in blue) and the β-galactosidase assay data (in red), both under C-limitation (lactose-limited growth; Fig S1).


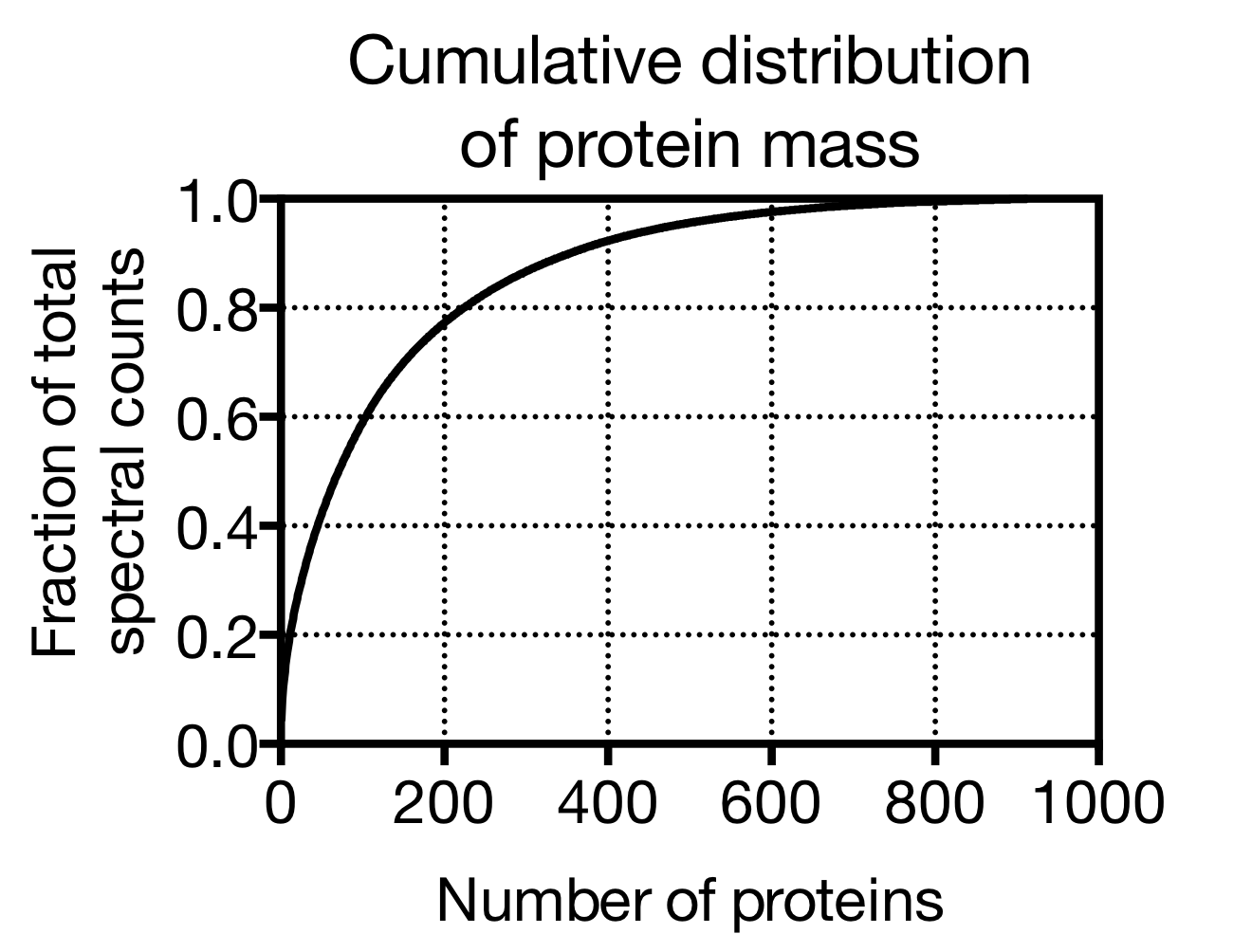


**Figure S5. Estimation of coverage of total protein mass by mass spectrometry.**

To estimate the fraction of total protein mass covered by mass spectrometry, we rely on two pieces of information: 1) the highly non-uniform distribution of individual protein mass as given by the method of spectral counting (shown above); and 2) the absolute protein quantitation results from the 2D gel study by Pedersen et al. (Pederse*n et* al, 1978). The plot shows the cumulative distribution of protein mass detected in the standard condition (i.e., WT cells growing in glucose minimal medium), with the proteins ranked from high to low according to their masses as measured by spectral counts. The 2D gel study found that in glucose minimal medium the most abundant 190 proteins account for about 60% of the total protein mass. Recent 2D gel absolute protein quantitation study (private communication with Scott, et al.) found a similar number for the same medium, with the top 190 proteins accounting for about 64% of the total protein mass. Here the plot shows that top 190 proteins comprise 76% of the total spectral counts. Therefore, the total proteome mass detected by the mass spectrometry is estimated to be between 60%/76%=79% and 64%/76%=84%. We thus take 80% as the estimated value for the coverage of total protein mass by mass spectrometry.

Recently, using the method of ribosome profiling, Li et al. (L*i et* al, 2014) was able to estimate the absolute protein abundance for *E. coli* strain MG1655 under three different growth conditions, glucose minimal medium, rich defined medium, and rich defined medium lacking methionine. Based on their data, the 1053 proteins we focus on occupy about 83% of total protein mass for their strain in all three conditions. However, as the strain we use, NCM3722, grows 30-50% faster than MG1655 in minimal medium, we did not attempt a more detailed quantitative comparison.


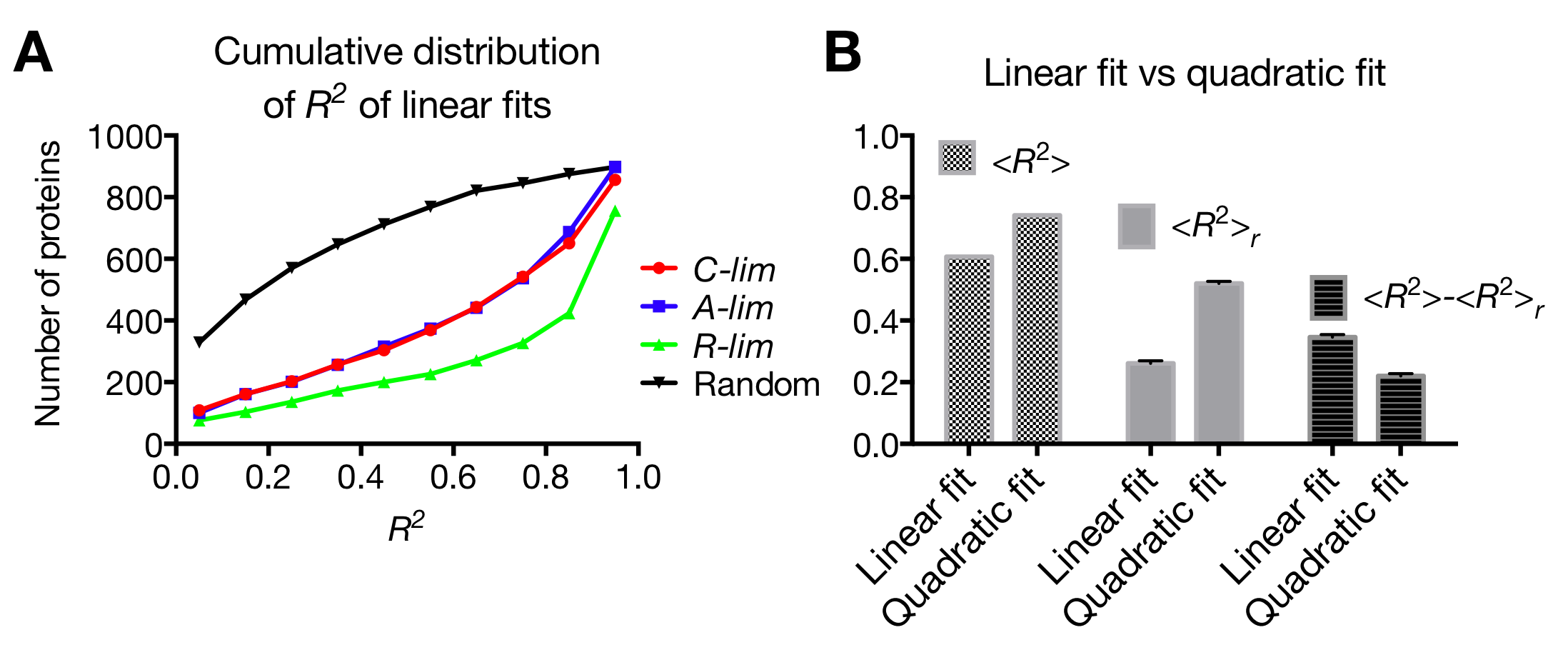


**Figure S6. Linearity of the growth-rate dependence of protein expression.**

A Cumulative distribution of R squared values (*R*^2^) of linear fits. For each of the three limitation data sets, a line was fit for each protein and its *R*^2^ value was calculated (See Table S2 for the parameters of fits). The red symbols and line show the cumulative distribution of *R*^2^ for C-limitation, while the blue and green data are for A- and R- limitation, respectively. The black symbols and line are for the A-limitation data with the expression values for each protein randomly permuted.

B For the A-limitation data set, both linear fit and quadratic fit were carried out for each protein, and an average of *R*^2^ values (denoated as <*R*^2^>) were calculated for all proteins in both cases. The first pair of bars compares the <*R*^2^> of the two fits, indicating not surprisingly that with respect to the null fit the quadratic fit performs better. This, however, does not mean that the quadratic fit is better than the linear fit, because the quadratic fit also performs better for random data (which was generated by randomly permutation of the expression data for each protein in the A-limitation data set), with larger value of <*R*^2^>_r_, as indicated by the second pair of bars. With respect to the performance for random data, the linear fit describes the A-limitation data better, i.e., it has a larger value for <*R*^2^>-<*R*^2^>_r_, as indicated by the third pair of bars.


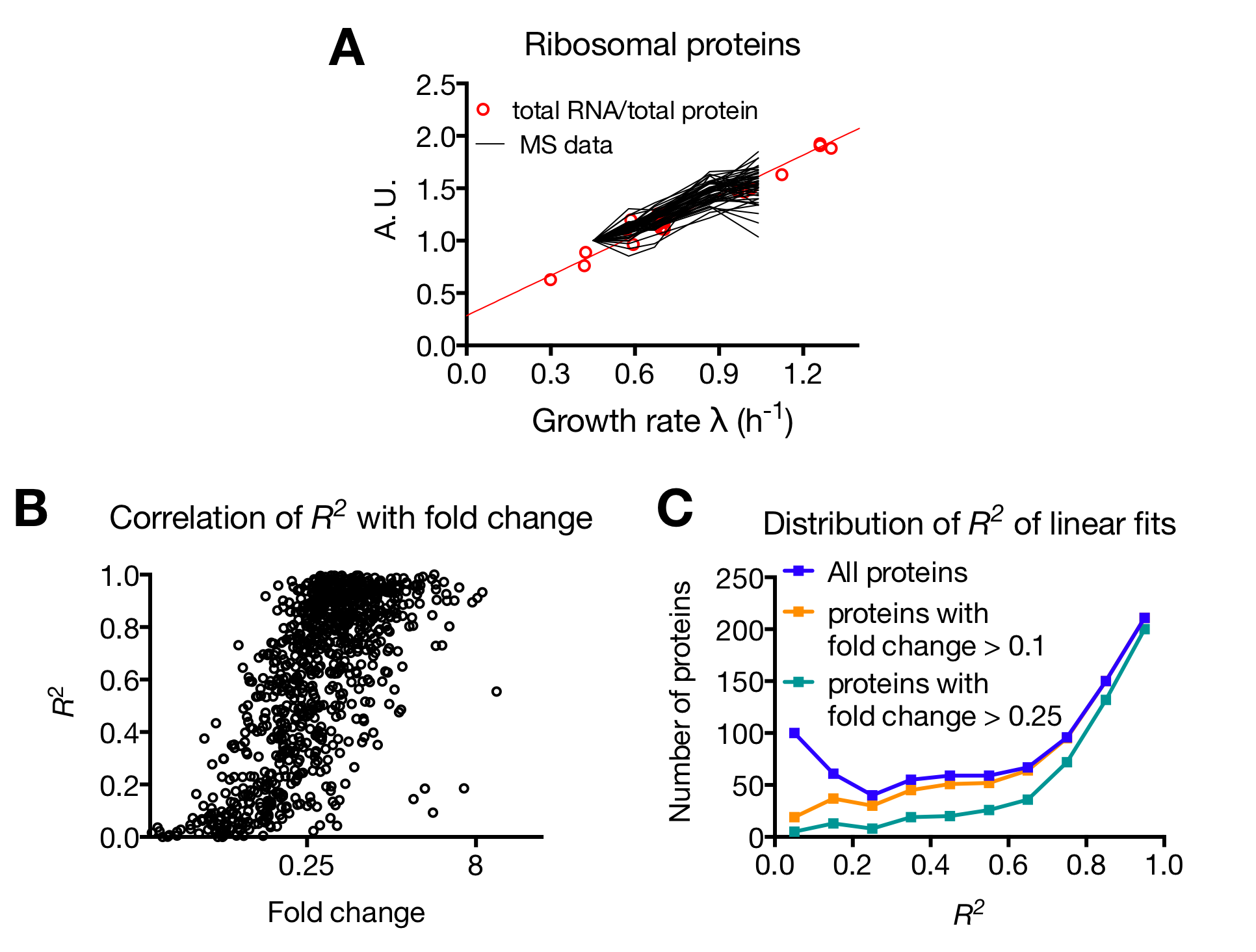


**Figure S7. Two causes for low values of** *R*^2^ **of some linear fits of the growth-rate dependence of protein expression.**

A The relative protein expression for 52 ribosomal proteins by mass spectrometry compared to ribosome abundance obtained from the total RNA over total protein measurements. The much larger spread of the ribosomal protein data by mass spectrometry suggests limited precision of the method for individual proteins.

B For the A-limitation data set, each protein’s *R*^2^ value of linear fit is plotted against the protein’s fold change. The fold change is in log-scale. There is a positive correlation between the two variables, with small values of *R*^2^ corresponding to small values of fold changes.

C For the A-limitation data set, the distribution of *R*^2^ values of linear fits to all the proteins is shown as the blue symbols and line. Note that there is a small peak of number of proteins at the zero end of the *R*^2^ value. While the distribution for only proteins with fold change greater than 0.1 is plotted (red symbols and line), the peak disappears, indicating that small values of *R*^2^ mostly correspond to small values of fold change, consistent with panel (B). This is more vividly shown with the distribution for proteins with fold change greater than 0.25 (green symbols and line), where the higher cutoff value of fold change filters out mostly proteins with small values of *R*^2^.


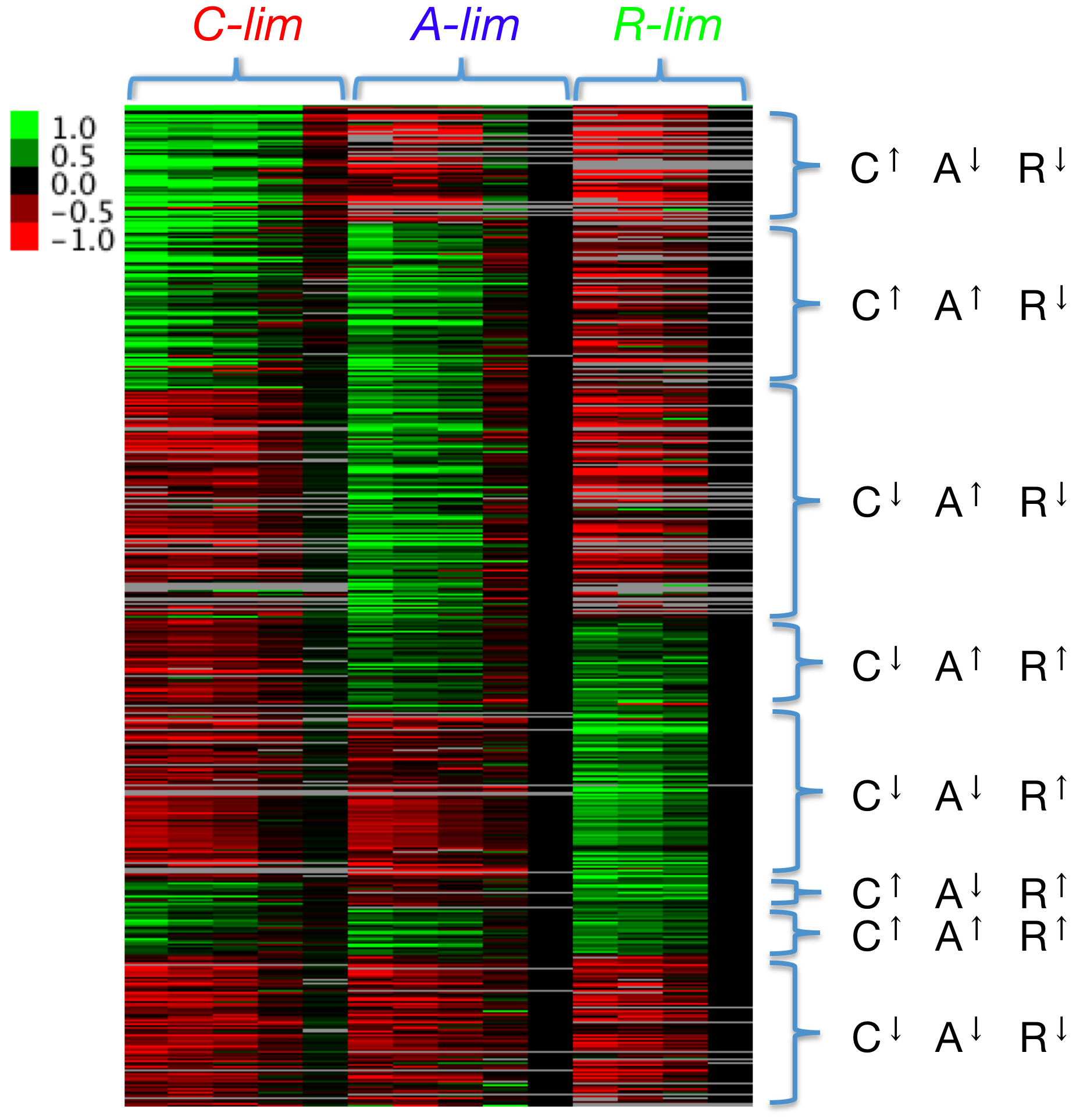


**Figure S8. Grouping proteins into 8 groups.**

In the expression matrix, the first five columns are for C-limitation, the next five columns for A-limitation, and the last four columns for R-limitation. Within each limitation, the growth rate increases from left to right. Red color indicates negative values, green color indicates positive values, and black indicates zero values. Gray indicates missing entries. The right side of the expression matrix shows the 8 groups. From top to bottom, the groups are C↑A↓R↓, C↑A↑R↓, C↓A↑R↓, C↓A↑R↑, C↓A↓R↑, C↑A↓R↑, C↑A↑R↑, and C↓A↓R↓, where the upward arrow denotes expression values going up (specific response) as growth rate goes down in a limitation and down arrow means the opposite (general response). If a protein is missing (i.e., having no values) under a limitation, we treated its response as general response.


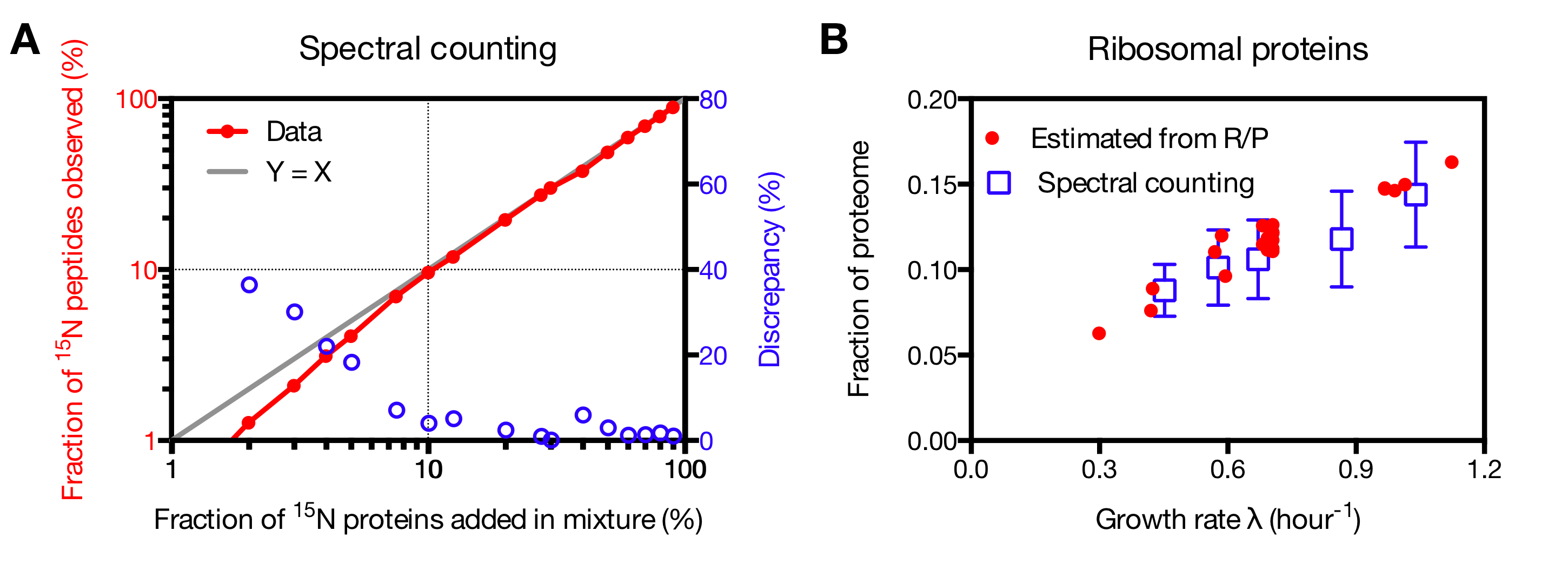


**Figure S9. Absolute protein quantitation with spectral counting.**

A Spectral counting data from the whole cell series. ^15^N-labeled cell sample was mixed with unlabeled cell sample at different proportions. The estimated fractions of the ^15^N-labeled proteins based on spectral counting are plotted against the real fractions (red symbols and line). Discrepancy between the estimated value from spectral counting and the expected value is defined as the absolute value of the difference between the two values divided by the expected value (blue symbols). The discrepancy quickly goes down as the fraction goes up, with around 20% for 5% of expected fraction and less than 10% for 7.5% of expected fraction.

B Comparison of spectral counting data with the R/P data and ribosomal profiling data (L*i et* al, 2014) for the proteome fraction of ribosomal proteins. The ribosomal protein fraction for various *E. coli* strains follows similar linear relation with growth rate when growth is limited by nutrients (Scot*t et* al, 2010). The red dots were estimated from R/P measurements (Supplemental Materials and Methods) of NCM3722 growing on various carbon and nitrogen sources, with the formula: fraction of proteome = 0.52*R/P (See Eq [S1] in (Scot*t et* al, 2010)). The blue squares are the spectral counting data of the C-limitation series, with error bars indicating the standard deviations from triplicate mass spectrometry runs. The green triangle data were obtained by calculating the mass fraction of the ribosomal proteins using the absolute protein abundance estimated by Li et al (L*i et* al, 2014). From slow to fast growth, the three triangles correspond to MG1655 strain growing on glucose minimal medium, rich defined medium without methionine, and rich defined medium.


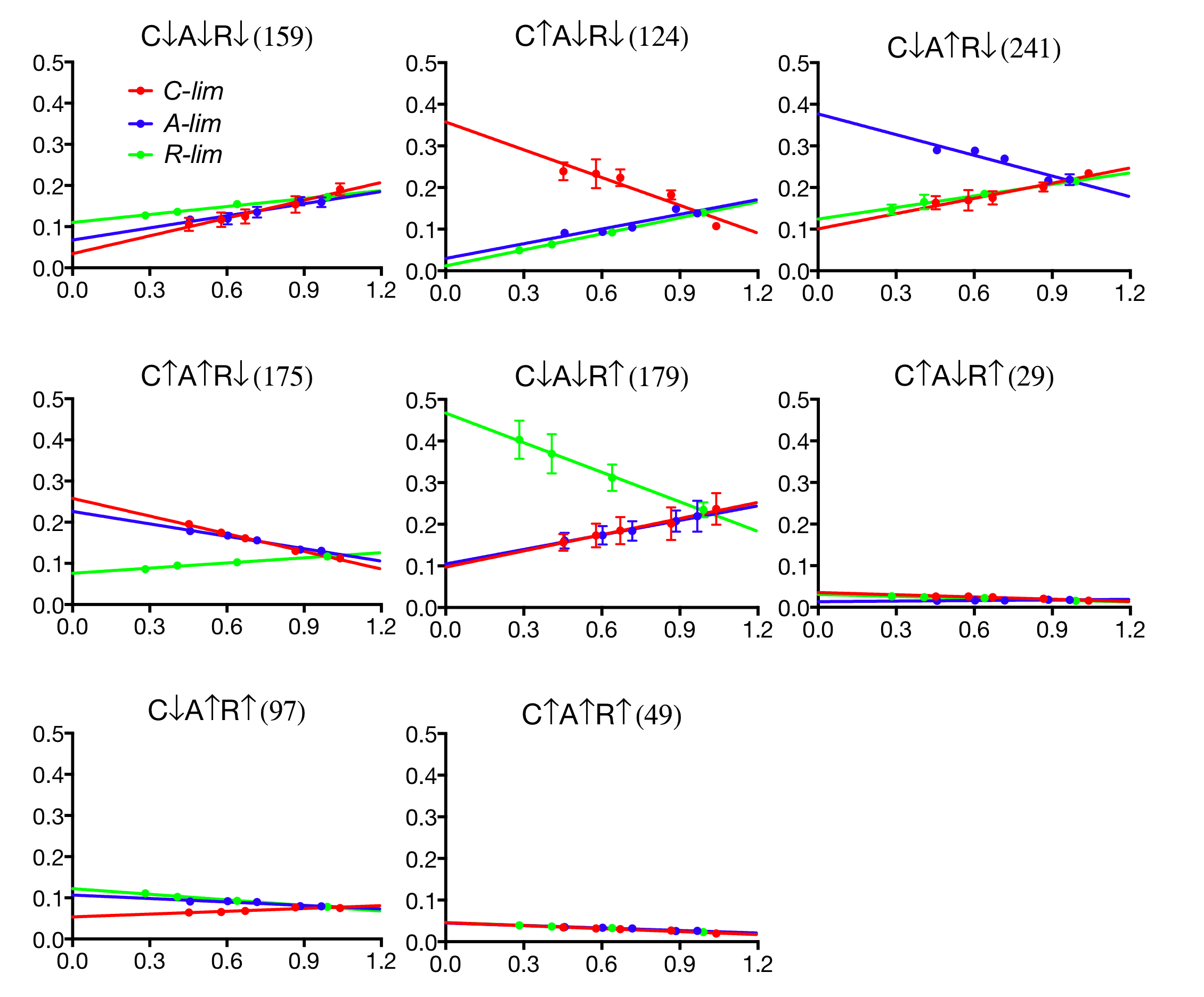


**Figure S10. Coarse-grained results for the 8 protein groups.**

The Y-axis of each of the plots is fraction of proteome and the X-axis is the growth rate (in units of per hour). The red symbols and lines are for C-limitation, blue for A-limitation, and green for R-limitation. The lines are the best linear fits to the data represented by symbols of the same colors. The title of a group indicates the types of response the group has to the three limitations, with an upward arrow (↑) for a line with negative slope and downward arrow (↓) for a line with positive slope. The number in the title indicates the number of proteins in the group.

The variation of the abundance for the triplicate runs is much larger for the C↓A↓R↑ group (or R-sector in Fig 3) than other sectors. This reflects the coarse-graining method we used for estimating the absolute abundance for proteome sectors. The method assumes a diverse representation of proteins with broad distributions of efficiencies in various steps of the experimental flow. The R-sector includes most of the r-proteins which together form one complex, ribosome. Similar behaviors of this large group of proteins in terms of noise could cause the observed large variation for the R-sector.


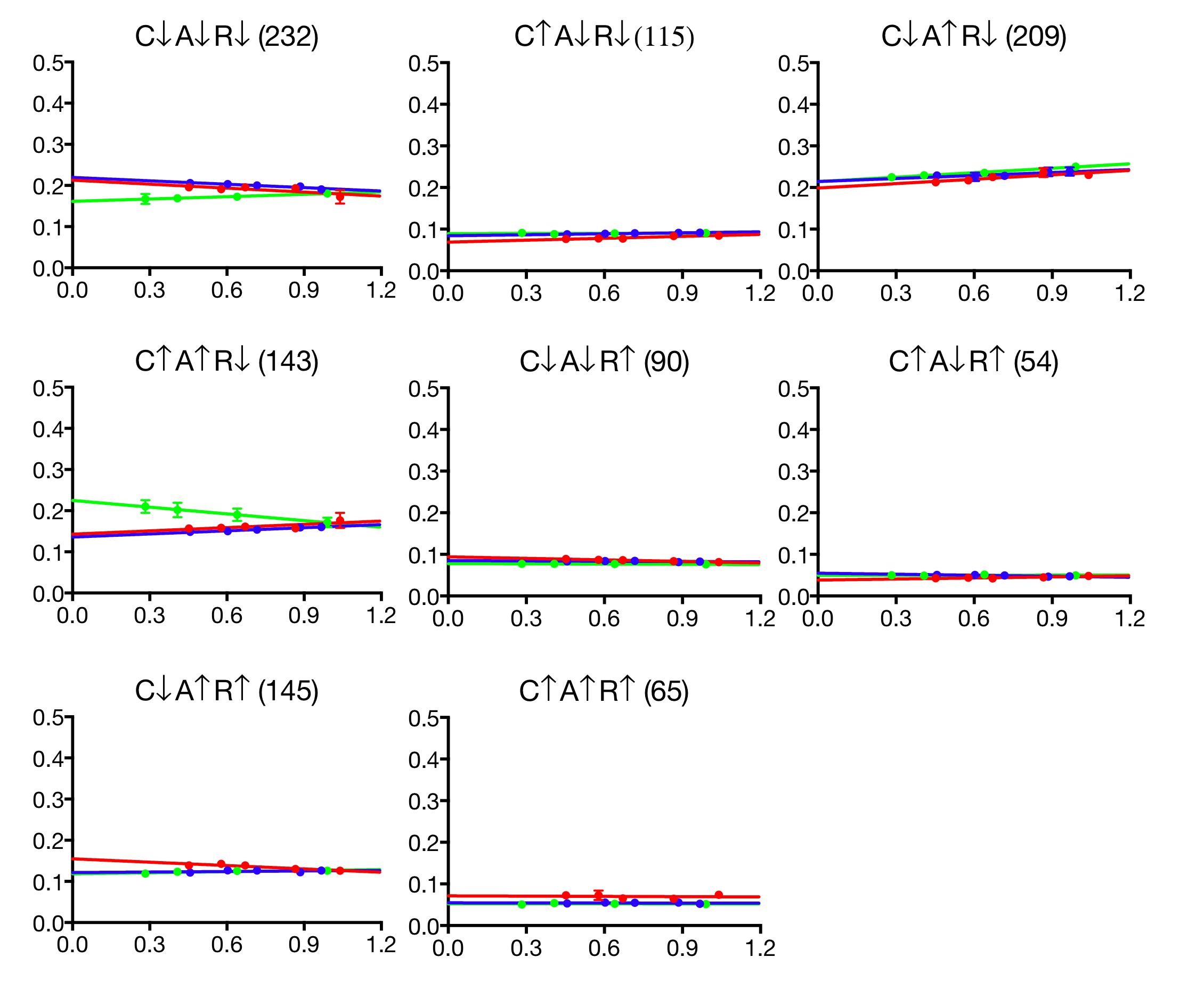


**Figure S11. Coarse-grained results for the randomly grouped groups.**

As described in the text, each relative protein data set is represented as an *N ×M* expression matrix, with *N* being the number of proteins and *M* the number of growth conditions corresponding to different degrees of growth limitation. Here we randomly shuffled the protein rows before grouping the proteins into 9 groups, in the same way as the group is carried out for the un-shuffled data sets. Coarse-graining was also carried out in the same way as for the original data. See the figure legend of Fig S10 for description of the plots.


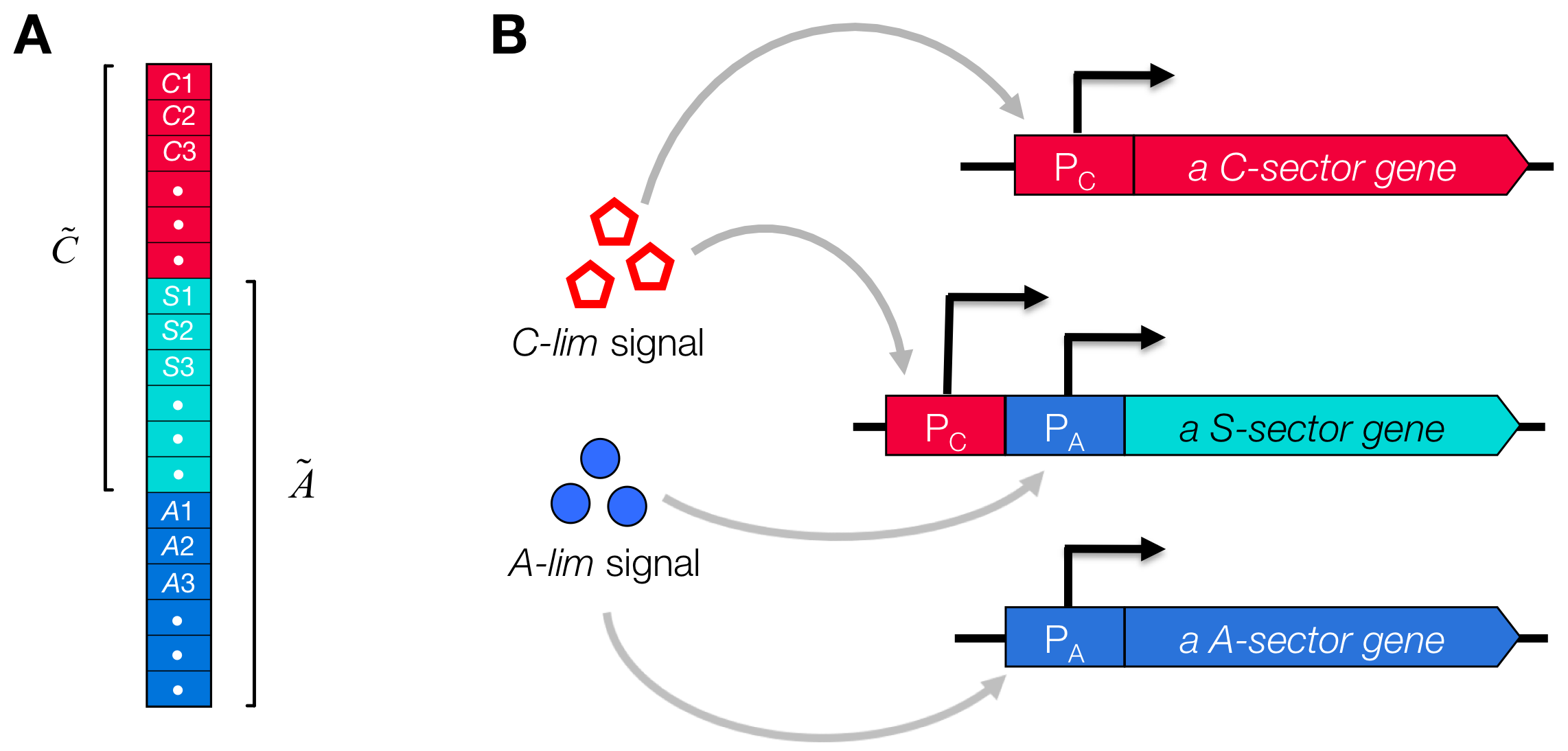


**Figure S12. S-sector proteins respond to both C- and A- limitations.**

A Illustration of two hypothetical lists of proteins, and , responding to only C-limitation and only A-limitation, respectively. While the C- and A- sector proteins belong only to the and respectively, the “multi-purpose” S-sector proteins belong to both lists.

B An illustrative mechanism generating the expression pattern of an S-sector protein: the corresponding gene is expressed by the activation of either the promoter P_c_ which responds to signals for C- limitation or the promoter P_A_ which responds to signals for A- limitation.

In the following, we derive the general and specific responses of the C-, A- and S- sectors. The growth-rate dependent components of and are denoted as and , respectively. Similar to the R- and U- sectors (Eqs [4-5]), we have for proteins

, [i]

and

, [ii]

for proteins, with *ν_C_* and *ν_A_* being the respective rate constants. We assume that a constant fraction (*f_C_*) of belongs to , the growth-rate dependent component of the S-sector. Similarly, we assume that a constant fraction (*f_A_*) of also belongs to . We then have

. [iii]

The remaining parts of and are respectively ∆*φ_C_* and ∆*φ_A_*, i.e.,

, [iv]

and

. [v]

Eqs. [iii], [iv], and [v] describe the general responses of the S-, C-, and A- sectors. To derive the specific responses of the sectors, we use the constraint given by Eq. [8] in the text. For example, under C-limitation where only *ν_C_* is changed, A-, R-, and U- sectors still follow the general responses. Using Eqs. [4-5], [v], and [8], we have

. [vi]

Using Eqs. [iii] and [iv], we have

. [vii]

Solving Eqs. [vi] and [vii] for and gives

, [viii]

where $\kappa_{C}^{-1}\equiv\nu_{A}^{-1}+\nu_{R}^{-1}+\nu_{U}^{-1}$.

Similarly, under A-limitation, we obtain

, [ix]

with $\kappa_{A}^{-1}\equiv\nu_{C}^{-1}+\nu_{R}^{-1}+\nu_{U}^{-1}$.

Eqs. [viii] and [ix] describe the specific responses of the three sectors. Inspired by the similar specific responses of the S-sector to both C- and A- limitations (see the two upward lines in Fig 3E), we simply used $f=f_{A}=f_{B}$ in Eqs. [viii] and [ix], yielding simpler equations for specific responses of the C-, A-, and S-sector (Eqs. [S8-10] of Table S6). Similarly, Eqs. [S3-5] of Table S6 are the result of applying this simplification to Eqs. [iii], [iv], and [v]. This simplification still allows good quantitative description of the data (Fig 5; Table S7).

# Supplementary Tables

| **Growth limitations** | **Medium** | **Strains, inducers/antibiotic amounts, and doubling times** | | | | | |
| --- | --- | --- | --- | --- | --- | --- | --- |
| **C-limitation** | Lactose minimal medium | **Titratable LacY**  **NQ381** (*attB*::P_Llac-O1_-*xylR*, *lacY*::*km-Pu-lacY* ) | | | | | **WT**  **NCM3722** |
|  |  | 3MBA (uM) | 0 | 25 | 50 | 500 |  |
|  |  | Dbl (min) | 92 | 72 | 62 | 48 | 40 |
| **A-limitation** | Glucose minimal medium | **Titratable GOGAT**  **NQ393** (*attB::Sp-lacIQ-tetR*, ∆*lacY*, *∆gdhA,*  P*_Llac-O1_-gltBD*) | | | | | **WT**  **NCM3722** |
|  |  | IPTG (uM) | 30 | 40 | 50 | 100 |  |
|  |  | Dbl (min) | 91 | 69 | 58 | 47 | 43 |
| **R-limitation** | Glucose minimal medium | **WT**  **NCM3722** (wild type) | | | | |  |
|  |  | Chloramphenicol (uM) | 8 | 4 | 2 | 0 |  |
|  |  | Dbl (min) | 147 | 102 | 65 | 42 |  |

**Table S1. Strains and growth conditions.**

Three strains were used in this study: the wild type NCM3722, NQ381, and NQ393. The latter two strains are based on NCM3722. The C-limitation was carried out by titrating the lactose uptake for the strain NQ381 growing on lactose minimal medium. Four growth rates were obtained for four different 3MBA levels. The fifth growth condition in the C-limitation series was WT NCM3722 growing on lactose minimal medium. The lactose minimal media were prepared with 1 mM IPTG.

NQ393 was used for the A-limitation, with four growth rates corresponding to four different IPTG levels in the glucose minimal medium. Similarly, WT NCM3722 growing on glucose minimal medium was the fifth growth condition in the A-limitation series.

WT NCM3722 was used for the R-limitation, with four growth rates corresponding to four chloramphenicol levels in the glucose minimal medium.

The fastest growth condition in both the A- and R- limitation series is the condition of WT cells growing on glucose minimal medium. We refer to this growth condition as the “glucose standard condition”, from which cell growth was A-limited or R-limited. Although C-limitation was carried out on lactose minimal medium, the growth conditions in the C-limitation series can still be regarded as C-limited growth states relative to the glucose standard condition, because glucose and lactose are just different carbon sources.

**Table S2. Relative protein expression data, parameters of linear fits, and membership in proteome sectors. See Excel file Supplementary Table S2 for table content.**

**Table S3. Proteome fractions for the 6 sectors under the three limitations.**

Proteome fraction data for the triplicate runs (the 4^th^-6^th^ columns) and their means (the 7^th^ column) are listed for each sector under each limitation. The means and the corresponding standard deviations are shown in Fig 3, with same color scheme for each of the three growth limitations. See Materials and Methods for how the proteome fraction data were obtained. See Excel file Supplementary Table S3 for table content.

|  | *C-lim ($l=C$)* | *A-lim ($l=A$)* | *R-lim ($l=R$)* |  |
| --- | --- | --- | --- | --- |
| C-sector ($\sigma=C$) | 0.35±0.01 | 0.04±0.01 | 0.01±0.01 | 0.14±0.00 |
| A-sector ($\sigma=A$) | 0.11±0.01 | 0.37±0.01 | 0.12±0.01 | 0.22±0.00 |
| R-sector ($\sigma=R$) | 0.10±0.01 | 0.10±0.01 | 0.47±0.01 | 0.23±0.00 |
| U-sector ($\sigma=U$) | 0.04±0.01 | 0.06±0.01 | 0.11±0.01 | 0.17±0.00 |
| S-sector ($\sigma=S$) | 0.25±0.01 | 0.24±0.01 | 0.07±0.01 | 0.12±0.00 |
| O-sector ($\sigma=O$) | 0.13±0.01 | 0.17±0.01 | 0.20±0.01 | 0.12±0.00 |
| *R*^2^ of the fit | 0.99 | | | |

**Table S4. Parameters describing the linear growth-rate dependence of the 6 proteome sectors under the three growth limitations.**

For a sector *σ*, 4 parameters are required to describe the responses to the three growth limitations, with 3 for the Y-intercepts () and 1 for the proteome fraction at the glucose standard condition (), i.e., . The fitted lines are shown in Fig 3. The *R^2^* of the fit measures the quality the overall fit (i.e., the 6×3=18 lines) with respect to the mean proteome fraction data (the last column in Table S3). This value of *R^2^* is also useful for later comparison with the quality of fit by the flux model (Table S7). See Materials and Methods for the definition of *R^2^*.

**Table S5. Lists of genes associated with each of the GO terms identified by the abundance-based GO analysis. See Excel file Supplementary Table S5 for table content.**

| **Sector** | ***C-lim*** | ***A-lim*** | ***R-lim*** |
| --- | --- | --- | --- |
| **C** | [S8a] $\kappa_{C}^{-1}=\nu_{A}^{-1}+\nu_{R}^{-1}+\nu_{U}^{-1}$ [S8b] | [S3] | [S3] |
| **A** | [S4] | [S9a] $\kappa_{A}^{-1}=\nu_{C}^{-1}+\nu_{R}^{-1}+\nu_{U}^{-1}$ [S9b] | [S4] |
| **R** | [S1] | [S1] | [S7a] $\kappa_{R}^{-1}=\nu_{C}^{-1}+\nu_{A}^{-1}+\nu_{U}^{-1}$ [S7b] |
| **U** | [S2] | [S2] | [S2] |
| **S** | [S10a] $\kappa_{S}^{-1}=\nu_{R}^{-1}+\nu_{U}^{-1}$ [S10b] | [S10a] $\kappa_{S}^{-1}=\nu_{R}^{-1}+\nu_{U}^{-1}$ [S10b] | [S5] |
| **O** | [S6] | [S6] | [S6] |

**Table S6. Flux model equations describing responses of the six sectors to the three growth limitations.**

The table lists equations describing all 18 responses, growth-rate ($\lambda$) dependences of proteome fractions of the 6 sectors () under the 3 growth limitations. As developed in the text, the equations are the results of the proteome-based flux model. These equations contain 16 parameters, 6 growth-rate independent components of proteome fractions (), 4 effective rate constants describing the slopes of general responses ($\nu_{C}$, $\nu_{A}$, $\nu_{R}$, and $\nu_{U}$), 2 global parameters ( and $f$), and 4 parameters describing the slopes of specific responses (*κ_C_*, *κ_A_*, *κ_S_*, and *κ_R_*). The last four parameters can be expressed as functions of rest of the parameters (Eqs. [S8b], [S9b], [S10b], and [S7b]), reducing the number of free parameters to 12. Due to the definition , the number of free parameters is further reduced to 11. For a given condition, Eqs. [4-8] yield an expression of growth rate *λ* as a function of the effective rate constants and *φ*_max_, i.e.,

, [S11]

which further eliminates one parameter if the growth rate of the condition is given.

| Parameters | | Determined values |
| --- | --- | --- |
| *6 growth-rate independent components* | *φ_C_,*_0_ | 0.06±0.01 |
|  | *φ_A_,*_0_ | 0.14±0.01 |
|  | *φ_R_,*_0_ | 0.09±0.01 |
|  | *φ_U_,*_0_ | 0.09±0.01 |
|  | *φ_S_,*_0_ | 0.07±0.01 |
|  | *φ_O_,*_0_ | 0.14±0.00 |
| *4 effective rate constants* | $\nu_{C}^{-1}$ | 0.11±0.02  0.30±0.04 (glycerol) |
|  | $\nu_{A}^{-1}$ | 0.10±0.02 |
|  | $\nu_{R}^{-1}$ | 0.14±0.02 |
|  | $\nu_{U}^{-1}$ | 0.07±0.02 |
|  | *f* | 0.32±0.03 |
|  | *φ*_max_ | 0.41±0.02 |
| *R*^2^ of the fit |  | 0.95 |

**Table S7. Parameters of the flux model.**

List here are 12 parameters, including 6 growth-rate independent components of the sectors, 4 effective rate constants, the constant *f*, and *φ*_max_. Only 10 of them are free parameters due to two relations among the parameters. The first one is the definition . The second relation is Eq. [S11] given that the growth rate *λ* is known for a condition.

For the glucose standard condition, $\lambda=\lambda^{*}$ (corresponding to a doubling time of 42 min). The parameter values were determined by fitting the 10-parameter flux model (Table S6) to the proteome responses data with respect to the glucose standard condition (Table S3). The results of the fit are shown as lines in Fig 5. The quality of the fit is measured by the value of *R^2^*. See Materials and methods for its definition.

For the glycerol standard condition, all parameters except *ν_C_* are expected to have these same values. The new *ν_C_* value (indicated in the table with “glycerol” next to it) was determined by Eq. [S11], using the growth rate $\lambda^{\dagger}$ (corresponding to a doubling time of 61 min) of the glycerol standard condition and parameter values from this table. This new value of *ν_C_*, together with the values of other parameters listed in this table, are used for the model (Table S6) to give the thick (both solid and dashed) lines in Fig 6.

| **Growth limitations** | **Medium** | **Strains, inducers/antibiotic amounts, and doubling times** | | | | | |
| --- | --- | --- | --- | --- | --- | --- | --- |
| **Glycerol C-limitation** | Glycerol minimal medium | **NQ399** (*attB*::P_Llac-O1_-*xylR*, *km-Pu-glpFK* ) | | | | | **NCM3722** (*wild type*) |
|  |  | 3MBA (uM) | 25 | 100 | 500 |  |  |
|  |  | Dbl (min) | 147 | 99 | 74 |  | 69 |
| **Glycerol A-limitation** | Glycerol minimal medium | **NQ393** (*attB::Sp-lacIQ-tetR*, ∆*lacY*, *∆gdhA,*  P*_Llac-O1_-gltBD*) | | | | |  |
|  |  | IPTG (uM) | 20 | 30 | 40 | 75 |  |
|  |  | Dbl (min) | 149 | 94 | 73 | 61 |  |
| **Protein overexpression** | glucose minimal medium | **NQ1389** (*Ptet-tetR on pZA31; Ptetstab-lacZ on pZE1*) | | | | | |
|  |  | cTc (ng/ml) | 12.5 | 10 | 5 | 2.5 | 0 |
|  |  | Dbl (min) | 95 | 79 | 58 | 51 | 47 |

**Table S8. Strains and growth conditions for the C- and A- limitations in the glycerol minimal medium, and for the growth limitation by protein overexpression.**

The glycerol C-limitation was carried out by titrating the glycerol uptake for the strain NQ399 (Yo*u et* al, 2013) growing on glycerol minimal medium. Three growth rates were obtained for three different 3MBA levels. The fourth growth condition in the glycerol C-limitation series was NCM3722 growing on glycerol minimal medium. The four glycerol C-limitation conditions all contained 1 mM IPTG. Strain NQ393 was used for the glycerol A-limitation, with the four growth rates corresponding to four different IPTG levels in the glycerol minimal medium. Strain NQ1389 was used for the growth limitation by protein overexpression, with five growth rates corresponding to five different chloro-tetracycline (cTc) levels in the glucose minimal medium.

|  | Doubling time (min) | C-sector | A-sector | R-sector | U-sector | S-sector | O-sector |
| --- | --- | --- | --- | --- | --- | --- | --- |
| *Gly C-lim* | 147 | 0.234 | 0.167 | 0.131 | 0.104 | 0.211 | 0.126 |
|  | 99 | 0.243 | 0.165 | 0.144 | 0.112 | 0.188 | 0.126 |
|  | 74 | 0.233 | 0.179 | 0.155 | 0.122 | 0.173 | 0.122 |
|  | 69 | 0.224 | 0.180 | 0.160 | 0.126 | 0.172 | 0.122 |
| *Gly A-lim* | 149 | 0.124 | 0.266 | 0.125 | 0.086 | 0.218 | 0.142 |
|  | 94 | 0.154 | 0.237 | 0.141 | 0.107 | 0.206 | 0.129 |
|  | 73 | 0.172 | 0.209 | 0.148 | 0.122 | 0.206 | 0.122 |
|  | 61 | 0.192 | 0.183 | 0.170 | 0.129 | 0.184 | 0.121 |
| *Protein overexpression* | 95 | 0.090 | 0.121 | 0.202 | 0.091 | 0.072 | 0.077 |
|  | 79 | 0.103 | 0.136 | 0.201 | 0.091 | 0.081 | 0.081 |
|  | 58 | 0.127 | 0.158 | 0.211 | 0.114 | 0.084 | 0.088 |
|  | 51 | 0.126 | 0.162 | 0.241 | 0.131 | 0.095 | 0.096 |
|  | 47 | 0.135 | 0.181 | 0.247 | 0.142 | 0.113 | 0.099 |

**Table S9. Proteome fraction data for the 6 sectors under the two growth limitations in glycerol medium, and the growth limitation by protein overexpression.**

Proteome fraction data for the 6 proteome sectors are listed for each of the two growth limitations in glycerol medium, and for the protein overexpression growth limitation. See Materials and Methods for how the proteome fraction data were obtained.

# Supplementary Text S1

**Supplemental Materials and Methods**

**Growth of bacterial culture**

**MOPS base medium:** All growth media used in this study were based on the MOPS-buffered minimal medium used by Cayley et al. (Cayle*y et* al, 1989) with slight modifications. The base medium contains 40 mM MOPS and 4 mM tricine (adjusted to pH 7.4 with KOH), 0.1 M NaCl, 10 mM NH_4_Cl, 1.32 mM KH_2_PO_4_, 0.523 mM MgCl_2_, 0.276 Na_2_SO_4_, 0.1 mM FeSO_4_, and the trace micronutrients described in Neidhardt et al. (Neidhard*t et* al, 1974). For ^15^N-labeled media, ^15^NH_4_Cl was used in place of ^14^NH_4_Cl.

**Growth measurements:** All batch culture growth was performed in a 37°C water bath shaker shaking at 250 rpm. The culture volume was at most 10 ml in 25 mm × 150 mm test tubes. Each growth experiment was carried out in three steps: “seed culture” in LB broth, “pre-culture” and “experimental culture” in identical minimal medium. For seed culture, one colony from fresh LB agar plate was inoculated into liquid LB and cultured at 37°C with shaking. After 4-5 hrs, cells were centrifuged and washed once with desired minimal medium. Cells were then diluted into the minimal medium and cultured in 37°C water bath shaker overnight (pre-culture). The overnight pre-culture was allowed to grow for at least 3 doublings. Cells from the overnight pre-culture was then diluted to OD600 = 0.005-0.025 in identical pre-warmed minimal medium, and cultured in 37°C water bath shaker (experimental culture). 200 μl cell culture was collected in a Starna Sub-Micro Cuvette (Starna Cells, Atascadero, CA) for OD600 measurement using a Thermal GENESYSTM 20 Spectrophotometer around every half doubling of growth. About 5-7 OD600 data points within the range of ~0.05 and ~0.5 (Above OD600=~0.6 the spectrophotometer was determined to be slightly nonlinear.) were used for calculating growth rate.

**Strain construction**

The strains used in this study are derived from *Escherichia coli* K12 strain NCM3722 (Soupen*e et* al, 2003; Lyon*s et* al, 2011) and summarized in Table S1 and Table S8.

**Construction of titratable *lacY* (NQ381) and titratable *glpFK* (NQ399) strains:** DNA fragment containing the *Pu* promoter (- 1 bp to -178 bp relative to the transcriptional start site) was amplified by PCR from a *Pu* promoter containing plasmid pEZ9, then inserted into the *Sal*I and *Bam*HI sites of plasmid pKD13, producing plasmid pKDPu. Using this plasmid as a template, the region containing the *km* gene and *P*u promoter was PCR amplified and integrated into the chromosome of *E. coli* strain NQ351 between the *lacZ* and *lacY* (from *lacZ* stop codon to *lacY* start codon), and in front of *glpF* (-1 bp to -252 bp relative to the translational start point of *glpF*) respectively, by using the λ Red system (Datsenko & Wanner, 2000). Because the activation of P*u* promoter needs the XylR protein, we constructed a strain NQ386 in which a synthetic *lac* promoter P_Llac-O1_ (Lutz & Bujard, 1997)(a promoter that is repressed by LacI but does not need Crp-cAMP for activation) driving *xylR* (*xylR* gene was cloned from pEZ6 (de Lorenz*o et* al, 1991)) was inserted at the *attB* site. The *km*-P*u-lacY* and km-Pu-glpFK constructs in NQ351 were transferred into strain NQ386 containing P_Llac-O1_-*xylR* by P1 transduction, resulting in strains NQ381 and NQ399, respectively.

**Construction of titratable *GOGAT* strain (NQ393):** Using the λ Red system (Datsenko & Wanner, 2000), we replaced the promoter (+123 bp to -176 bp) of *gltBDF* operon by the synthetic *lac* promoter P*_Llac-O1_* (Lutz & Bujard, 1997) (a promoter that is repressed by LacI but does not need Crp-cAMP for activation) together with selection maker *km* gene. The resulting *Km-P_Llac-O1_-gltBDF* construct was transferred to strain NCM3722 by P1 transduction (Thomaso*n et* al, 2007). The *km* gene was then eliminated by using plasmid pCP20 (Cherepanov & Wackernagel, 1995). A *sp*-*lacI*Q-*tetR* cassette providing constitutive expression of *lacI* to tightly repress P*_Llac-O1_* activity was inserted at the *attB* site by P1 transduction. Lactose permease encoded by *lacY* can concentrate intracellular IPTG and will narrow the titration range, we inactivated *lacY* by P1 transduction using strain JW0334-1 from CGSC (*E. coli* Genetic Stock Center, Yale University) as *ΔlacY* donor following by *Km* gene elimination. The *gdhA* gene was knocked out by P1 transduction using strain JW1750-2 from CGSC as *ΔgdhA* donor following by *Km* gene elimination to obtain the final strain NQ393.

**Construction of *lacZ* overexpression strain (NQ1389):** The *lacZ* structural gene was amplified from *E. coli* MG1655 with upstream and downstream primers including the digestion sites *Xho*I and *BamH*I respectively. The PCR products were gel purified, digested with *Xho*I and *BamH*I, then inserted into the same sites immediately downstream of P_L_*_tet-_*_O1_ in the pZE11 plasmid(Lutz & Bujard, 1997), yielding pZE11-*lacZ*. To improve the stability of P_L_*_tet-_*_O1_ with respect to homologous recombination, we later replaced the promoter sequence with the following modified promoter sequence (the underlined bases are changed as compared to the original sequence of P_L_*_tet-_*_O1_), which we refer to as the P_tetstab_ promoter:

CTCGAGTCCCTATCAGTGATAGCTCTTGACAGATCTATCAATGATAGAGATACTGAGCACATATGCAGCAGGACGCACTGACCGAATTCATTAAAGAGGAGAAAGGTACC

To construct this promoter, we first synthesized a single DNA fragment

CTCTTGACAGATCTATCAATGATAGAGATACTGAGCACATATGCAGCAGGACGCACTGAC

that served as template for PCR amplification of P_tetstab_ using primers ptetstab-F and ptetstab-R (see the primer table below for the sequences). The products were purified, digested with *Xho*I and *Kpn*I and substituted for P_L_*_tet-_*_O1_ in pZE11*-lacZ*. This yielded the plasmid pZE11 P*_tetstab_*-*lacZ*. We then transformed this plasmid into NCM3722 in combination with the auto-regulated TetR plasmid pZA31 P_L_*_tet-_*_O1_-*tetR*(Klump*p et* al, 2009), creating strain NQ1389 with a stable, titratable system capable of high levels of LacZ expression.

| **Primer** | **Plasmid/Construct** | **Use/ Digestion Sites** | **Sequence** |
| --- | --- | --- | --- |
| ptetstab-F | pZE1 P*tetstab*-*lacZ* | Forward amplification P*tetstab*, *XhoI* | ACACTCGAGTCCCTATCAGTGATAGCTCTTGACAGATCTATCAATG |
| ptetstab-R | pZE1 P*tetstab*-*lacZ* | Reverse amplification P*tetstab*, *BamHI* | TGTGGTACCTTTCTCCTCTTTAATGAATTCGGTCAGTGCGTCCTGCTGCATATG |

**Total protein and total RNA Measurements, and β-Galactosidase Assay**

**Total protein quantitation:** The Biuret method was used for total protein quantitation (Herbert et al. 1971). Briefly, 1.8 ml of cell culture at around OD_600_=0.5 during the exponential phase was collected by centrifugation. The cell pellet was washed with water and re-suspended in 0.2 ml water and fast frozen on dry ice. The cell pellet was then thawed in water bath at RT. 0.1 ml 3M NaOH was added to the cell pellet and samples were incubated at 100°C heat block for 5 min to hydrolyze proteins. Samples were then cooled in water bath at RT for 5 min. The biuret reactions are carried out by adding 0.1 ml 1.6% CuSO_4_ to above samples with thorough mixing at RT for 5 min. Samples were then centrifuged and the absorbance at 555 nm was measured by a spectrophotometer. Same biuret reaction was also applied to a series of BSA standards to get a standard curve. Protein amounts in the above samples were determined by the BSA standard curve.

**Total RNA quantitation:** The RNA quantitation method is based on the method used by Benthin et al. (Benthin et al. 1991) with modifications. Briefly, 1.5 ml of cell culture at around OD_600_=0.5 during the exponential phase was collected by centrifugation and the cell pellet was fast frozen on dry ice. The cell pellet was thawed and washed twice with 0.6 ml cold 0.1 M HClO_4_, then digested with 0.3 ml 0.3 M KOH for 60 min at 37°C with constant shaking. The cell extracts were then neutralized with 0.1 ml 3 M HClO_4_ and centrifuged at 13,000 rpm for 5 min. The supernatant was collected and the precipitate was washed twice with 0.55 ml 0.5 M HClO_4_. A final volume of 1.5 ml of supernatant was then centrifuged and the supernatant was measured for its absorbance at 260 nm on a Bio-Rad spectrophotometer. The RNA concentration (μg/ml/ OD_600_) was given by OD_260_ x 31/OD_600_, where we have used the converting factor of 31 between the OD_260_ and RNA concentration. The converting factor of 31 is based on the molar extinction coefficient is 10.5 mmole^-1^cm^-1^ and the average molecular weight of an *E. coli* RNA nucleotide residue is 324.

**β-Galactosidase Assay:** Samples (0.2 ml cell culture) were collected, fast frozen on dry ice and stored at -80°C prior to β-Galactosidase assay. Four samples were collected for each culture during exponential growth (for OD_600_ = 0.1~0.5). For each sample collected, β-Galactosidase activity was measured at 37°C by the traditional Miller method (Miller, 1972). The activities obtained (in unit of U/ml=OD_420_/min/ml) were plotted against the respective OD_600_, and the resulting slope from linear regression is taken to be the “LacZ expression level” (in unit of U/ml OD_600_, or “Miller Unit”).

# Supplementary Text S2

**Probabilistic binary classification of proteins**

**Introduction**

In the main text, we have classified the proteins into one of the 8 groups assuming a binary response of each protein (i.e., either ‘up’ or ‘down’) under a given mode of growth limitation. This “clear-cut” deterministic classification can, however, be an oversimplification because proteins with small change under a growth limitation can be misclassified due to the precision limitation of the method. To examine the effect of possible misclassification, here in this note we classify proteins using a probabilistic binary classification by calculating the probability that a protein belongs to one of the 8 groups. We then obtain the coarse-grained proteome fractions for the resulting groups and apply the same model presented in the main text to describe the results.

**Calculation of the probability that a protein is classified to a group**

To determine the probability that a protein $i$ belongs to a particular group, we first need to calculate the probability that the protein $i$ goes up under each of the growth limitations ($p_{i,l}$), with $p_{i,C}$ for C-limitation, $p_{i,A}$for A-limitation, and $p_{i,R}$for R-limitation. For example, for the group g=C^↑^A^↓^R^↓^ where proteins go up under C-limitation but down under A- and R- limitations, the probability ($P_{g,i}$) that the protein $i$ belongs to the group is given by$P_{g,i}=p_{i,C}(1-p_{i,A})(1-p_{i,R})$. For every protein, a total number of 8 values can be calculated, corresponding to the probabilities that it belongs to each of the 8 groups.

To calculate $p_{i,l}$, we determine the slope $s_{i,l}$ and its standard error $\delta_{i,l}$ by doing a linear fit to the protein expression data versus the growth rate for the protein $i$ under the limitation $l$, with the error in the growth rate as 0.05 and the error in the protein expression data given by the third quartile minus the first quartile divided by 2 (see the “relative protein quantification section in the Materials and Methods for definitions of quartiles.) Assuming a Gaussian distribution for the slope with the mean as $s_{i,l}$ and width as $\delta_{i,l}$, i.e., $f(x,s,\delta)=\frac{1}{\delta\sqrt{2\pi}}e^{-\frac{(x-s)^{2}}{2\delta^{2}}}$, we have$p_{i,l}=\int_{-\infty}^{0} f(x,s_{i,l},\delta_{i,l})dx$, which is the probability that the slope is negative. For proteins that were not detected under a growth limitation, we assign a value of 0 to $p_{i,l}$, assuming that the poor detection is due to decreased protein expression.

**Probabilistic coarse-graining of proteome fractions for the groups**

Given that the proteome fraction for a protein $i$ is based on the spectral counting data, the coarse-grained proteome fraction for the group $g$ is simply . The results are shown in Fig T2-1.


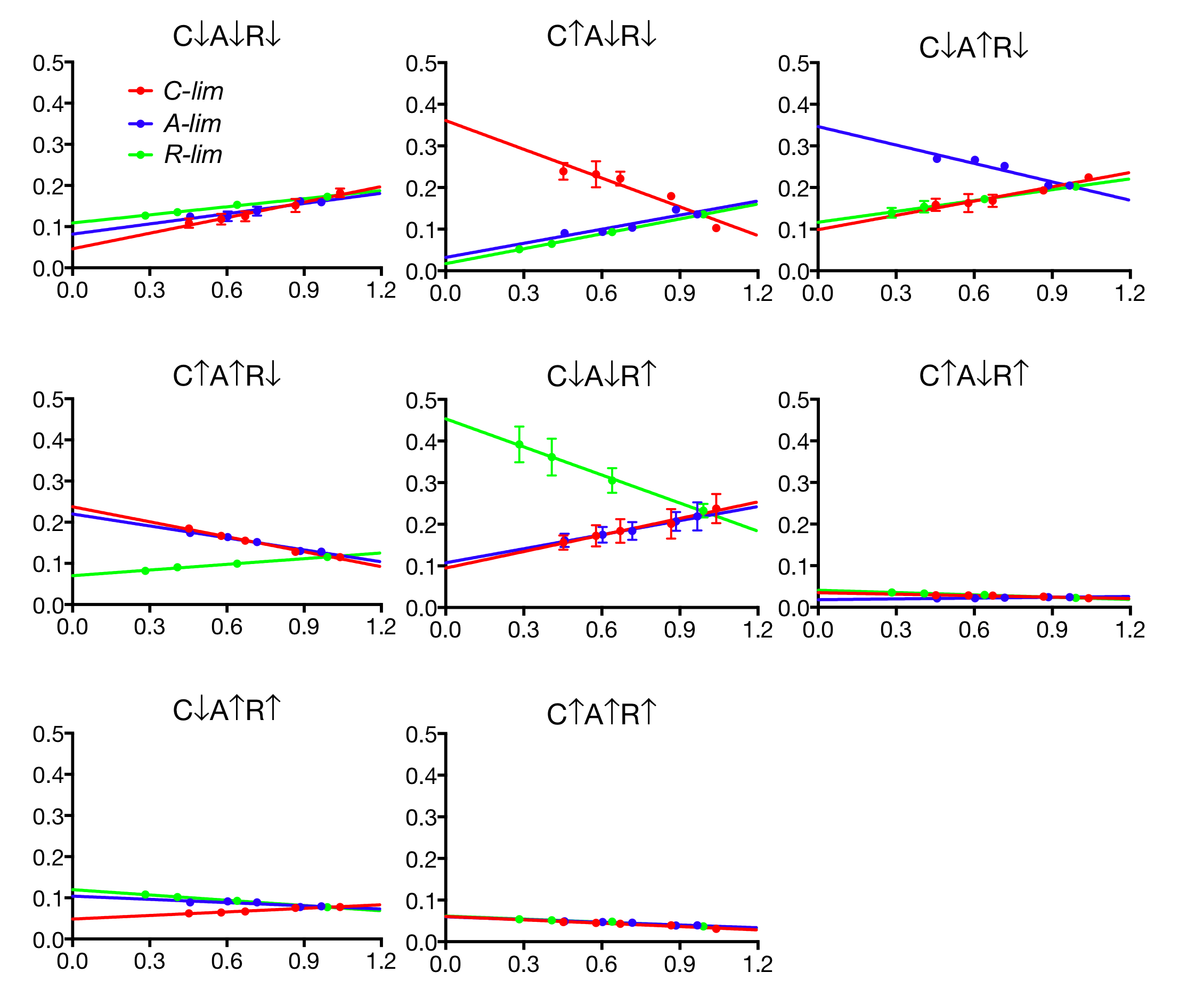


Figure T2-1. Coarse-grained proteome fractions for the 8 groups.

**Comparison with the deterministic binary classification**

To see how our results are affected by this probabilistic approach, we follow the similar procedure in the main text, by first obtaining 6 sectors (with the three small groups, C^↑^A^↓^R^↑^, C^↓^A^↑^R^↑^, and C^↑^A^↑^R^↑^ lumped together into the O-sector), and then fitting the 10-parameter model to the 6 sectors (Fig T2-2).


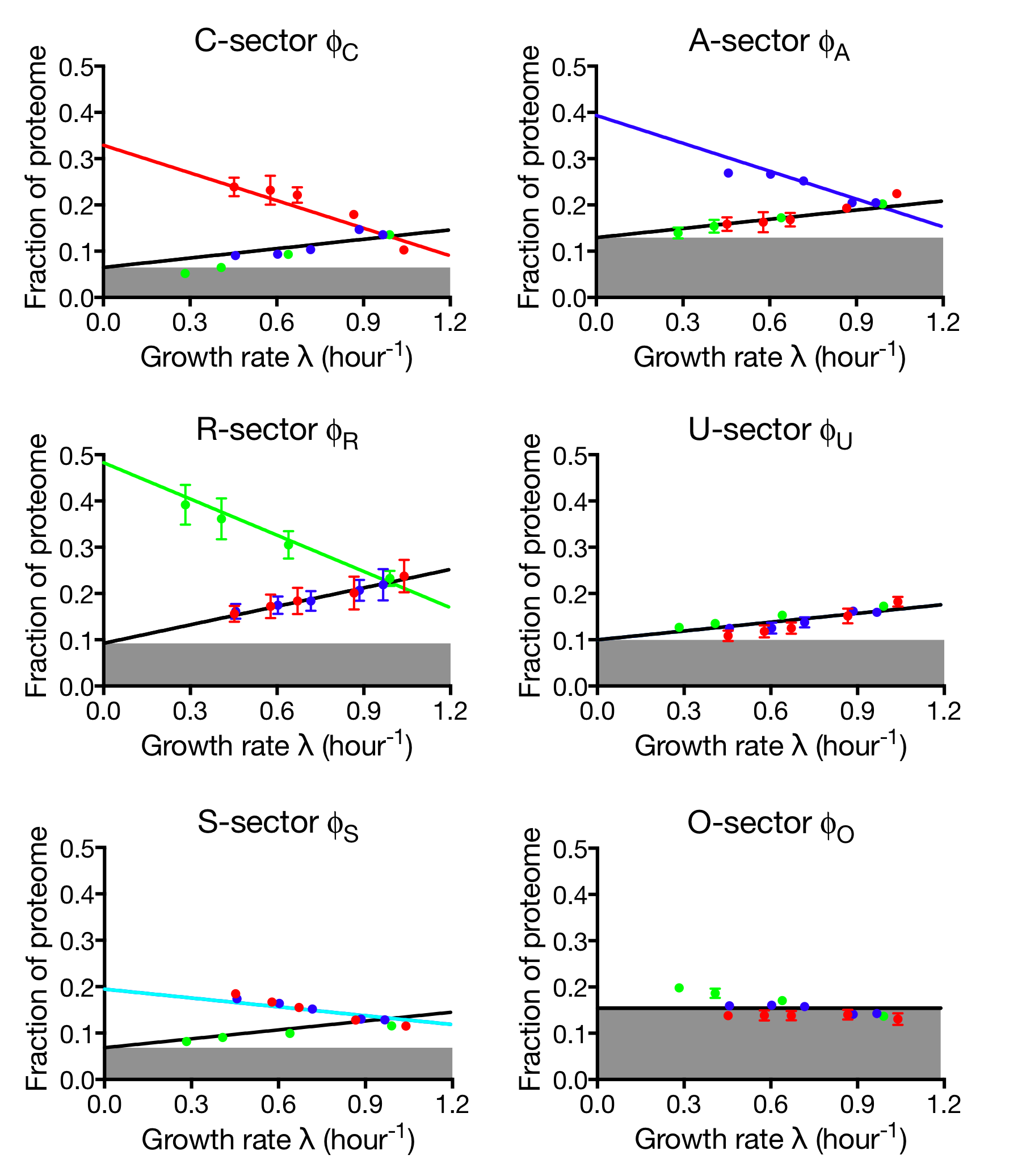


Figure T2-2. Model description of the coarse-grained proteome fractions from the probabilistic binary classification.

A comparison between the resulting model parameters is shown in Fig T2-3, with the parameters only slightly changed in the probabilistic classification. The result demonstrates that given the noise level of our data, the deterministic binary classification is a reasonable simplification.


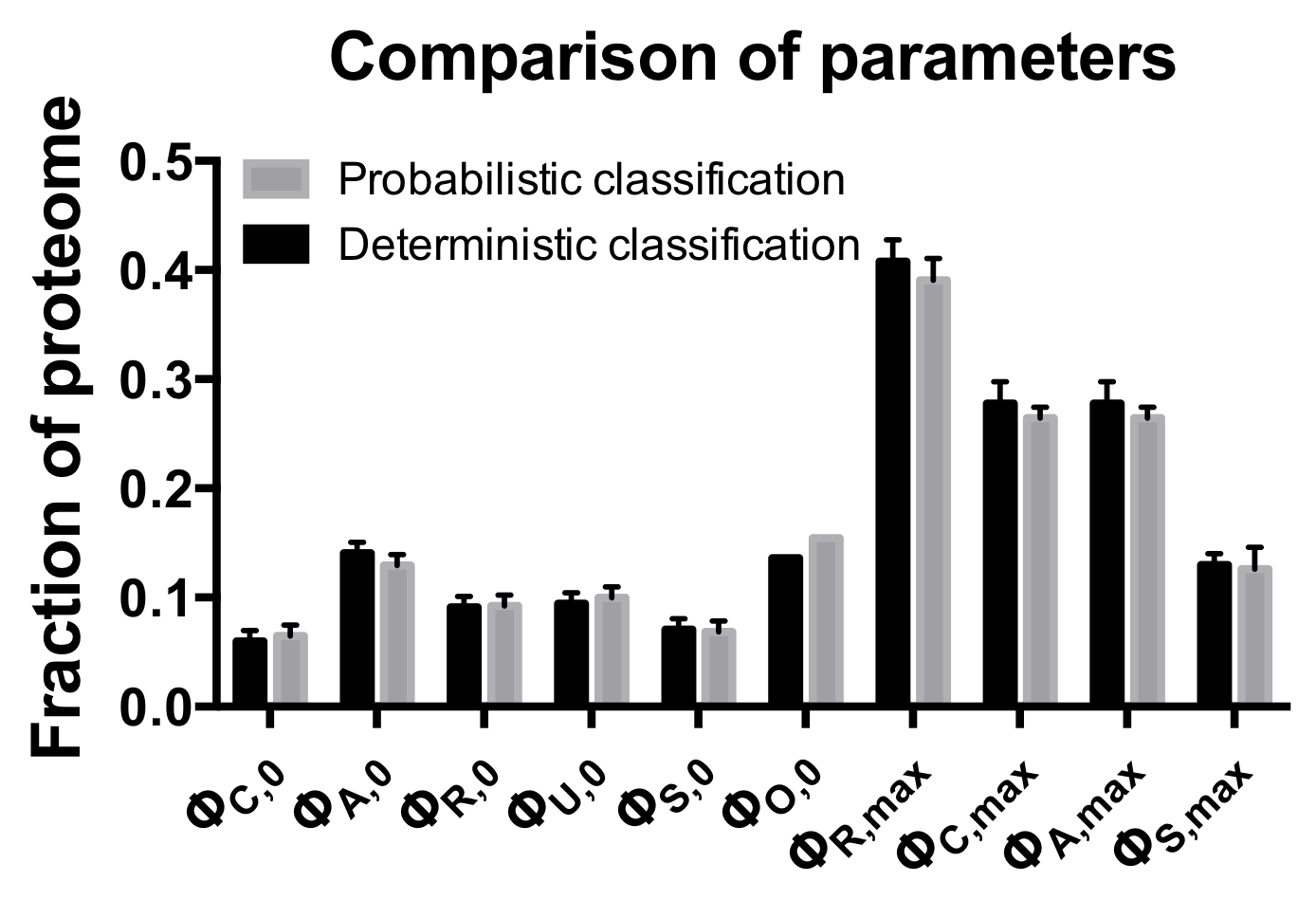


Figure T2-3. Comparison of parameters between the deterministic binary classification and the probabilistic classification.

# Supplementary Text S3

**An abundance-based functional analysis of sectors**

**Introduction**

We want to identify the biological functions of proteins in each proteome sector. The standard analysis of Gene Ontology (GO) terms [ref] identifies a list of terms that are enriched for a given set of genes compared to a background list of genes, e.g., the genome. Biological functions for the set of genes are then inferred from the identified GO terms. This approach is not well suited for our purpose due to the fact that individual proteins have vastly different abundance in the proteome, e.g., the elongation factor Tu comprises ~20% of the R-sector, but may be lost as a single gene among ~200 in the group. To take this fact into consideration, i.e., to answer the question what are the functions of the vast majority of proteins (by mass) doing in a sector, we formulated an abundance-weighted GO analysis. The analysis aims to identify for each sector a list of non-redundant GO terms that best reflect the functions of the sector, in terms of the amount of protein invested.

Our strategy is to first filter out pathological GO terms that are not meaningful, e.g., “cellular process” which is associated with 83% of the proteins; see Table T3-1. Then we perform an abundance-weighted enrichment of GO terms for each sector. After removing trivial redundancies (e.g., the terms “taxis” and “chemotaxis” are both enriched in C-sector, and only the most specific terms is kept), we perform a procedure to remove overlapping GO terms: We enumerate all possible combinations of *k* GO terms and calculate the overlap in protein abundance covered by these terms. This overlap quickly explodes as *k* increases beyond the order of 4~5 terms (see Fig T3-2), yielding a set of GO terms that accounts for most the proteins found in a sector by abundance. With this procedure, for each proteome sector we have reached a small number of lists of GO terms that can represent the protein functions of the sector.

**Data files**

**Gene ontology and gene association files**. The gene ontology file and the gene association file of *E. coli* were downloaded from the Gene Ontology project website ([http://geneontology.org](http://geneontology.org/)). The “data-version” of the ontology file is “2013-07-17” and the “date” is “16:07:2013 13:38”. The gene association file has a “submission date” of “6/5/2013” and a “GOC validation date” of “6/14/2013”. The ontology file contains information for the hierarchical relations between GO terms. In the gene association file, a gene is associated with GO terms that lie at the bottom of the hierarchy. The two files together provide full correspondence between genes and GO terms. For our purpose of identifying biological functions of proteome sectors, we consider only the “biological process” GO terms.

**Abundance data set.** We use the spectral counting data for the glucose standard condition, which was obtained by merging the spectral counting data of the triplicate runs of the R-limitation sample with no chloramphenicol. As it becomes clear later, we found that it is convenient to introduce the mathematical concept of set for representing spectra. We denote all the spectra in the data set as members in a set *S_0_*, so that the number of members in the set (or *S_0_*) is the spectral counts (e.g., the number of spectra) in the data set. Note that a spectrum in the data set is a recording event of a peptide by the mass spectrometer. The same peptide can occur multiple times and each occurrence is counted as one spectrum.

**Filtering out GO terms**

For each of the sectors, before we focus on lists of GO terms, we first filter out GO terms that are not justified for inclusion in the representing list. For this purpose, we define the following two quantities for a GO term, “fraction of sector”, and “fraction of proteome”.

**Fraction of sector of a GO term**. For a proteome sector *i*, the fraction of sector of a GO term *t* (*ω_i,t_*) is defined as,

$\omega_{i,t}\equiv\frac{\left|S_{t}\cap S_{i}\right|}{\left|S_{i}\right|}$,

where *S_i_* is a set of spectra that belong to a proteome sector *i*, and *S_t_* is a set of spectra that are associated with the GO term *t* (through the association between the GO term and its corresponding proteins in the data set). *ω_i,t_* represents how much of the abundance of the sector *i* the GO term *t* can account for. It is clear that for our purpose of identifying a representing list, we want to filter out GO terms with small values of *ω_i,t_*.

**Fraction of proteome of a GO term.** Similarly, for the whole proteome, the fraction of proteome of the GO term *t* (Ω*_t_*) is defined as:

$\Omega_{t}\equiv\frac{\left|S_{t}\right|}{\left|S_{0}\right|}$.

We want to filter out GO terms with large values of Ω*_t_*, because those terms would be too general (or too prevalent) to represent a particular sector.

**Three filters.** To decide the cutoff values for the above two quantities, we make a scatter plot of *ω_i,t_* versus Ω*_t_* for all GO terms and for all sectors (Fig T3-1).


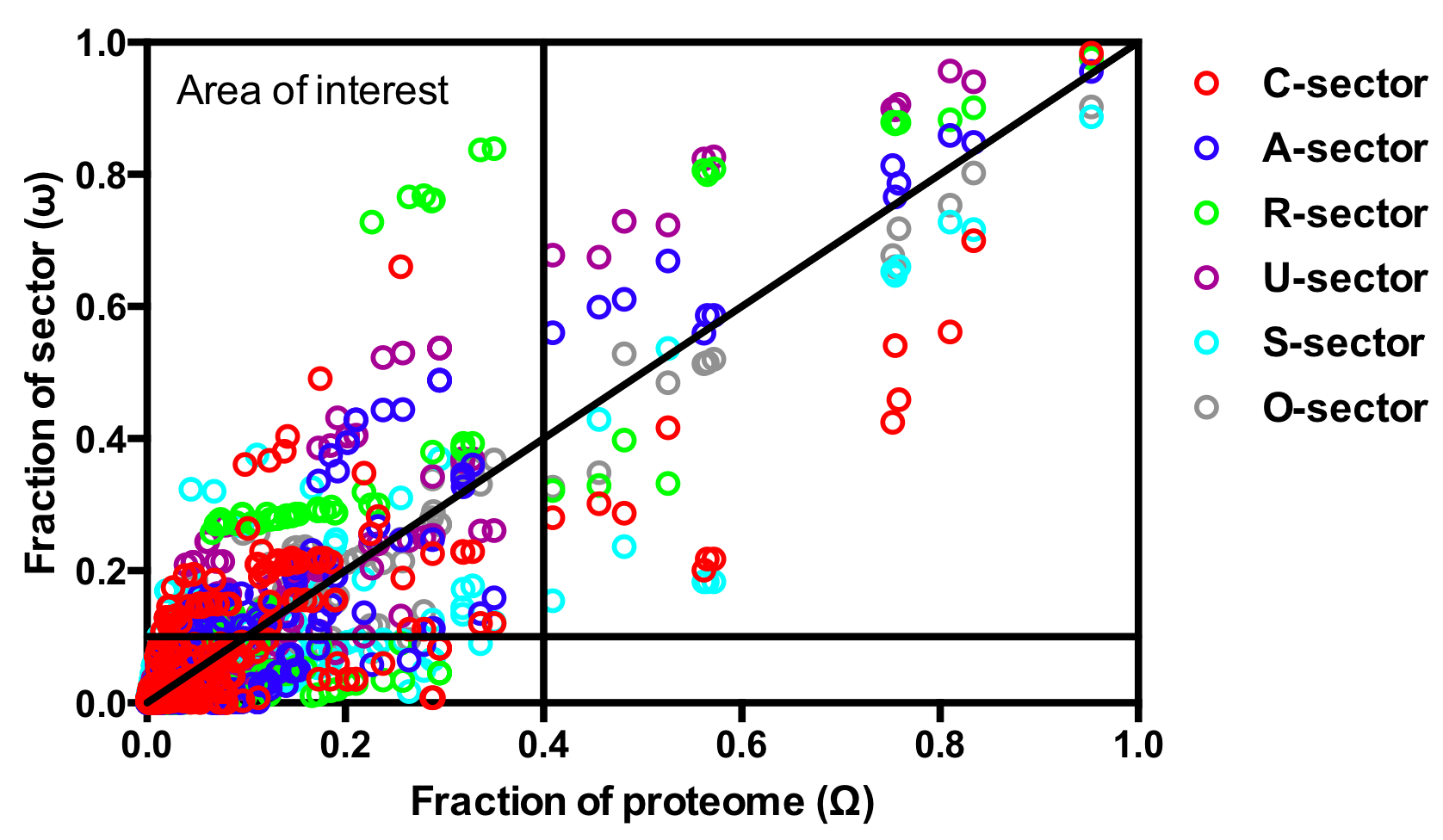


Figure T3-1. Scatter plot of fraction of sector versus fraction of proteome for all GO terms.

As mentioned above, we are interested in the data points that are in the top left corner of the plot (indicated as “area of interest” in Fig T3-1), because they represent GO terms that account for a large fraction of a sector and account for a small fraction of proteome. To define the area of interest mathematically we used 1) *ω_i,t_* > 0.1, and 2) Ω*_t_* < 0.4, as indicated by the horizontal and vertical black lines in Fig T3-1. The first criterion filters out GO terms that are small for a sector, i.e., accounting for less than 10% of the sector. The second criterion removes GO terms that are too general, accounting for more than 40% of the total proteome (see the list in Table T3-1). The numerical values for the criteria are chosen in such a way that they are not strict, i.e., filtering out only terms that are clearly not justified to represent the biological functions of a sector. We also introduce a third criterion: 3) *ω_i,t_* > Ω*_t_*, as indicated by the third black line in Fig T3-1. This criterion is a measure of “enrichment”, i.e., terms above the line are more enriched in a sector, compared to their distribution in the whole proteome.

| **Fraction of proteome** | **GO name** | **GO ID** |
| --- | --- | --- |
| 0.95 | biological_process | GO:0008150 |
| 0.83 | cellular process | GO:0009987 |
| 0.81 | metabolic process | GO:0008152 |
| 0.76 | cellular metabolic process | GO:0044237 |
| 0.75 | primary metabolic process | GO:0044238 |
| 0.75 | organic substance metabolic process | GO:0071704 |
| 0.57 | biosynthetic process | GO:0009058 |
| 0.57 | organic substance biosynthetic process | GO:1901576 |
| 0.56 | cellular biosynthetic process | GO:0044249 |
| 0.53 | single-organism metabolic process | GO:0044710 |
| 0.48 | nitrogen compound metabolic process | GO:0006807 |
| 0.46 | small molecule metabolic process | GO:0044281 |
| 0.41 | organonitrogen compound metabolic process | GO:1901564 |

Table T3-1. List of GO terms that are general.

The three filters reduce the number of GO terms from 1584 to less than 100 for each of the sectors (See the row of “After the three filters” in Table T3-2).

| **Sector** | | **C** | **A** | **R** | **U** | **S** | **O** |
| --- | --- | --- | --- | --- | --- | --- | --- |
| **Number of remaining GO terms** | **After the three filters** | 73 | 55 | 82 | 80 | 39 | 35 |
|  | **After the 4^th^ filter** | 40 | 47 | 46 | 59 | 31 | 32 |

Table T3-2. Number of remaining GO terms after subjecting all terms to filters.

**The fourth filter.** Next focusing on the remaining GO terms for a sector *i*, we continue filtering out a term *t*_1_ if there is another term *t*_2_ for which *t*_1_ is a parent term and has $\omega_{i,t_{1}}=\omega_{i,t_{2}}$. *t*_1_ is a parent term to *t*_2_ if *t*_2_ has a relation of “is_a” with *t*_1_ according to the gene ontology file. This fourth filter is reasonable because *t*_2_ is more “specific” than *t*_1_ while both account for the same fraction of sector. Table T3-3 lists for the C-sector some examples of those terms that are filtered out in this way. For example, the GO term “taxis” is filtered out because the term “taxis” is a parent term to the term “chemotaxis” which accounts for the same fraction of the C-sector. This fourth filter was applied to all of the sectors and further reduced the number of remaining GO terms (See the row of “After the 4^th^ filter” in Table T3-2). We denote the remaining GO terms for a sector *i* as set *T_i_*.

|  | **Terms filtered out** | | **Corresponding "specific" terms** | |
| --- | --- | --- | --- | --- |
| **Fraction of sector** | **GO name** | **GO ID** | **GO name** | **GO ID** |
| 0.21 | biological regulation | GO:0065007 | regulation of biological process | GO:0050789 |
| 0.17 | nucleoside metabolic process | GO:0009116 | purine nucleoside metabolic process | GO:0042278 |
| 0.17 | glycosyl compound metabolic process | GO:1901657 | nucleoside metabolic process | GO:0009116 |
| 0.17 | purine-containing compound metabolic process | GO:0072521 | purine nucleoside metabolic process | GO:0042278 |
| 0.16 | purine nucleotide metabolic process | GO:0006163 | purine ribonucleotide metabolic process | GO:0009150 |
| 0.16 | ribonucleoside metabolic process | GO:0009119 | purine ribonucleoside metabolic process | GO:0046128 |
| 0.16 | nucleoside triphosphate metabolic process | GO:0009141 | purine nucleoside triphosphate metabolic process | GO:0009144 |
| 0.16 | nucleoside triphosphate metabolic process | GO:0009141 | ribonucleoside triphosphate metabolic process | GO:0009199 |
| 0.16 | purine nucleoside triphosphate metabolic process | GO:0009144 | purine ribonucleoside triphosphate metabolic process | GO:0009205 |
| 0.16 | ribonucleotide metabolic process | GO:0009259 | purine ribonucleotide metabolic process | GO:0009150 |
| 0.16 | ribonucleoside triphosphate metabolic process | GO:0009199 | purine ribonucleoside triphosphate metabolic process | GO:0009205 |
| 0.16 | ribose phosphate metabolic process | GO:0019693 | ribonucleotide metabolic process | GO:0009259 |
| 0.13 | taxis | GO:0042330 | chemotaxis | GO:0006935 |

Table T3-3. Examples of GO term pairs that are identified by the fourth filter.

**Filtering out lists of GO terms**

Our task now is to identify from *T_i_* a list of GO terms *l* (denoted as set *T_l_*) that best represents a sector. Similar to the procedure carried out for filtering out GO terms, we subject lists of GO terms to three filters before we are left with a small number of lists. The three filters involve defining three measures for a list of GO terms, degree of overlapping between GO terms in the list, fraction of sector of the list, and gene coverage of the list.

**Degree of overlapping between GO terms in a list.** Note that *T_l_* ⊆ *T_i_* and thus *T_l_* (denoted as *k*) can be any integer from 1 to *n*, where *n* ≡ *T_i_*. It is clear that *k* should not be too big for the representing list or else the GO terms in the list will overlap with one another. Two GO terms *t*_1_ and *t*_2_ overlap with each other in a sector *i* if . To quantify the extent of term overlap, we introduce a measure called the “degree of overlapping” (O*_i,l_*) for a list *l* and a sector *i*. To calculate O*_i,l_*, we first calculate for a GO term *t* in the list *T_l_* (i.e., *t* ∈*T_l_*) the following quantity:

,

where *j* ∈*T_l_* and *k* ≡ *T_l_*). We then take the maximal value of O*_i,l,t_* for *t* ∈*T_l_* to be the value of O*_i,l_*. According to this definition, O*_i,l_* = 0 for lists with single terms and for lists with non-overlapping terms. At the other extreme where one term of a list accounts for a subset of spectra that another term in the same list accounts for, the degree of overlapping is at its maximum, or O*_i,l_* = 1. Note that as *k* increases, the values of O of the lists tend to get bigger. Fig T3-2 shows for the C-sector the minimal value of O*_C,l_* for all *l*‘s plotted against the size of lists *k*. For *k* < 5, there exist lists with zero overlapping, or O*_C,l_* = 0, while for larger *k* the minimal value of O*_C,l_* quickly goes up. By choosing a cutoff value for O, we can decide a maximum value for *k*. For example, a rather large (or loose) cutoff value of 0.3 already allows us to only consider lists with *k* < 5 for *O_C,l_*, *k* < 7 for *O_A,l_*, *k* < 4 for *O_R,l_*, *k* < 6 for *O_U,l_*, *k* < 6 for *O_S,l_*, and *k* < 5 for *O_O,l_*.


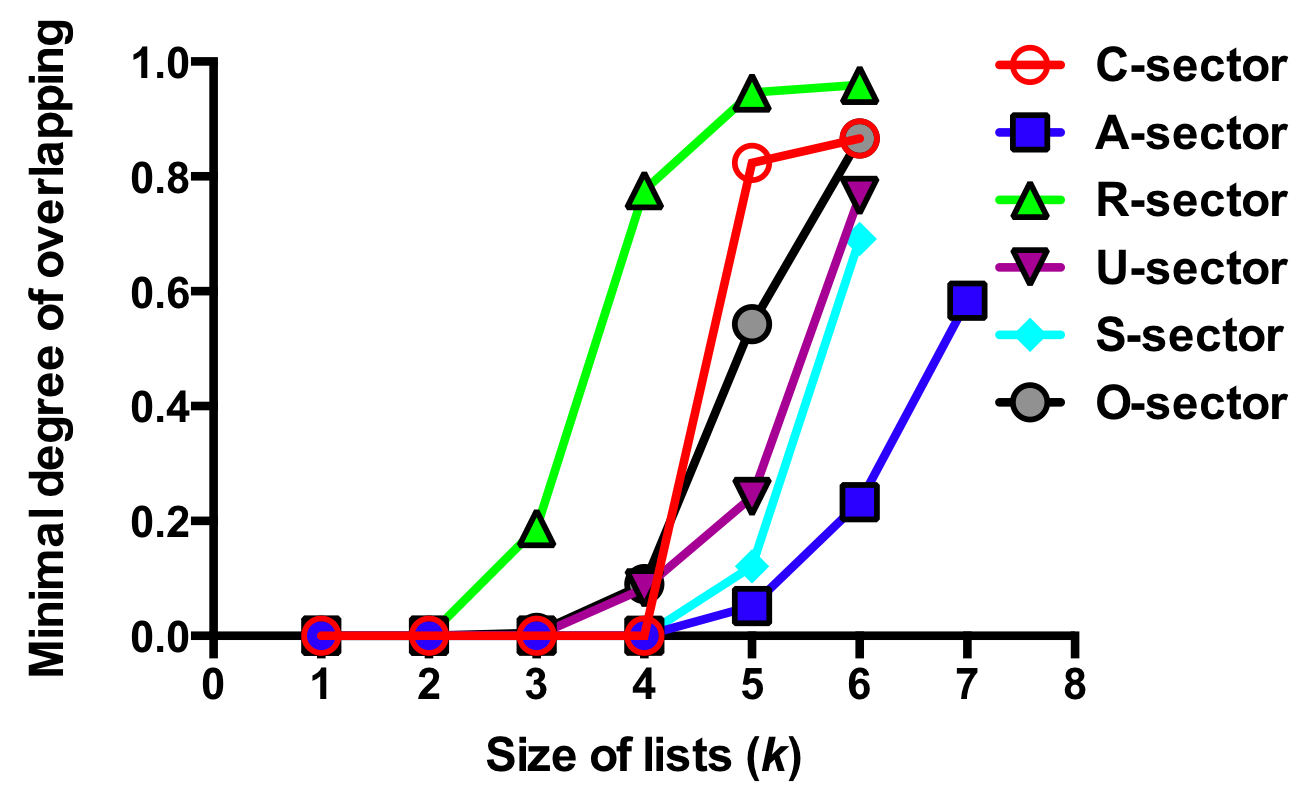


Figure T3-2. The minimal degree of overlapping as a function of the list size for each of the sectors.

To decide the cutoff value for the degree of overlapping, we inspect the histograms of lists with degree of overlapping less than 0.3 (Fig T3-3). We choose a cutoff value of 0.05, which leaves us with many lists to consider, ranging from hundreds to thousands.


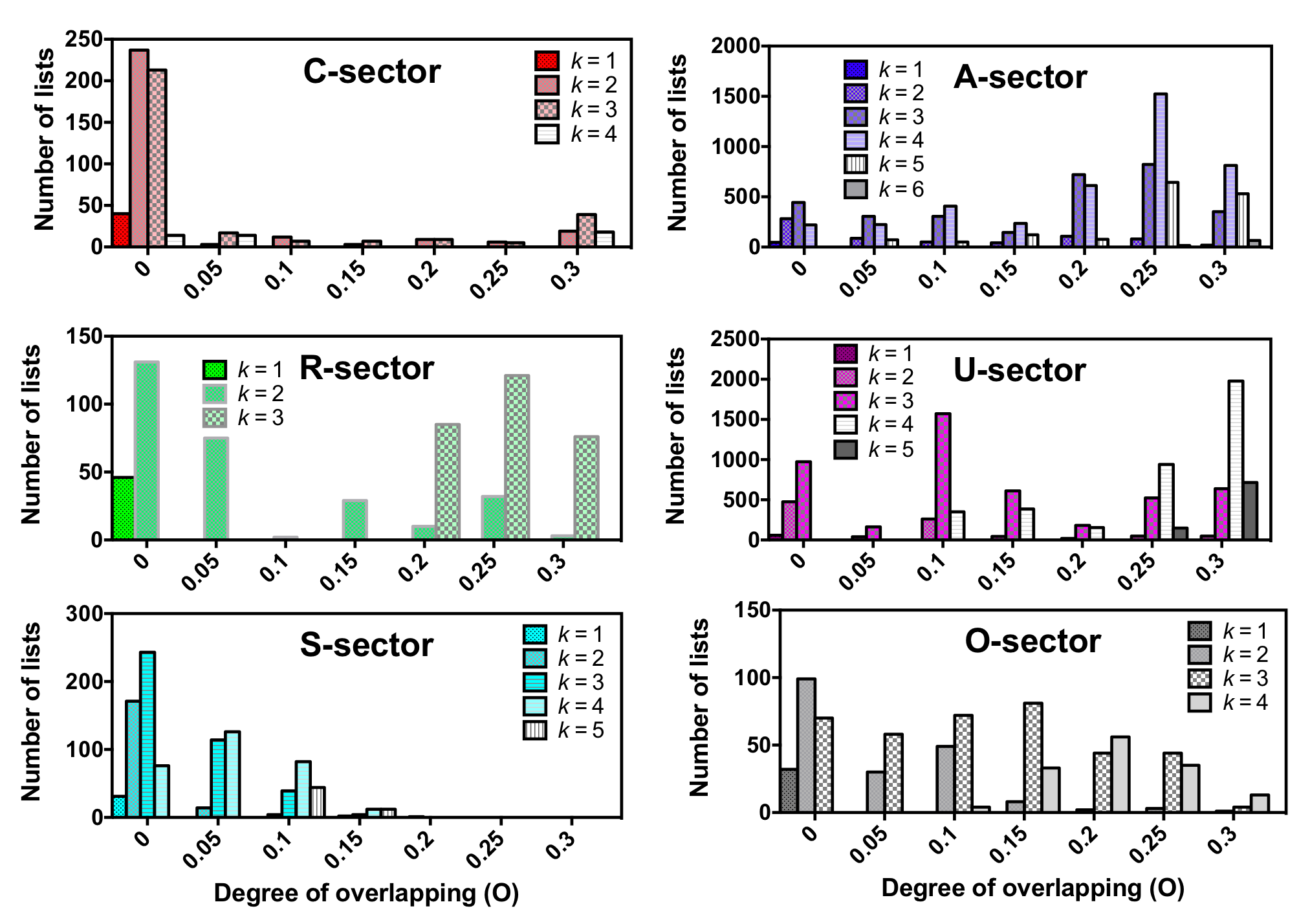


Figure T3-3. Histograms of the degree of overlapping for GO lists.

**Fraction of sector of a list of GO terms.** The measure of “fraction of sector” can be extended to a list of GO terms (*ω_i,l_*),

,

where *j* ∈ *T_l_* and *k* ≡ *T_l_*).

The representing list should have a large value of *ω*. We choose *ω_C,l_* > 0.6, i.e., the list *l* has to account for at least 60% of the C-sector.

In summary, by requiring O*_C,l_* > 0.05 and *ω_C,l_* > 0.6, we reached 78 lists for the C-sector, from which we continue identifying the representing one for the sector. We carried out the same procedure to the other sectors (Fig T3-3) and Table T3-4 shows the number of lists that satisfy the respective cutoff values for O and *ω*.

| **Sector** | **C** | **A** | **R** | **U** | **S** | **O** |
| --- | --- | --- | --- | --- | --- | --- |
| **Cutoff value for** O | 0.05 | 0.05 | 0.05 | 0.05 | 0.05 | 0.05 |
| **Cutoff value for** *ω* | 0.6 | 0.6 | 0.6 | 0.6 | 0.6 | 0.6 |
| **Number of lists** | 78 | 140 | 7 | 123 | 87 | 16 |

Table T3-4. Cutoff values for the degree of overlapping and fraction of sector of lists of GO term lists, and the remaining number of lists after applying the two filters.

**Gene coverage of a list of GO terms.** There is another attribute for a list GO terms and it is what we refer to as “gene coverage”. A GO term *t* is associated with a number of genes in the genome, which is denoted as the set *G_t_*. The set of genes included in a proteome sector *i* is denoted as set *G_i_*. The “gene coverage” *χ_i,t_* for the GO term in the proteome sector is defined as follows,

$\chi_{i,t}\equiv\frac{\left|G_{t}\cap G_{i}\right|}{\left|G_{t}\right|}$.

The definition can be generalized to a list of GO terms and is denoted as *χ_i,l_* for the list *l* and for the sector *i*,

,

where *j* ∈*T_l_* and *k* ≡ *T_l_*).

Fig T3-4 shows the histogram of *χ* for each of the sectors, with the total number of lists in each histogram given in Table T3-4.

**
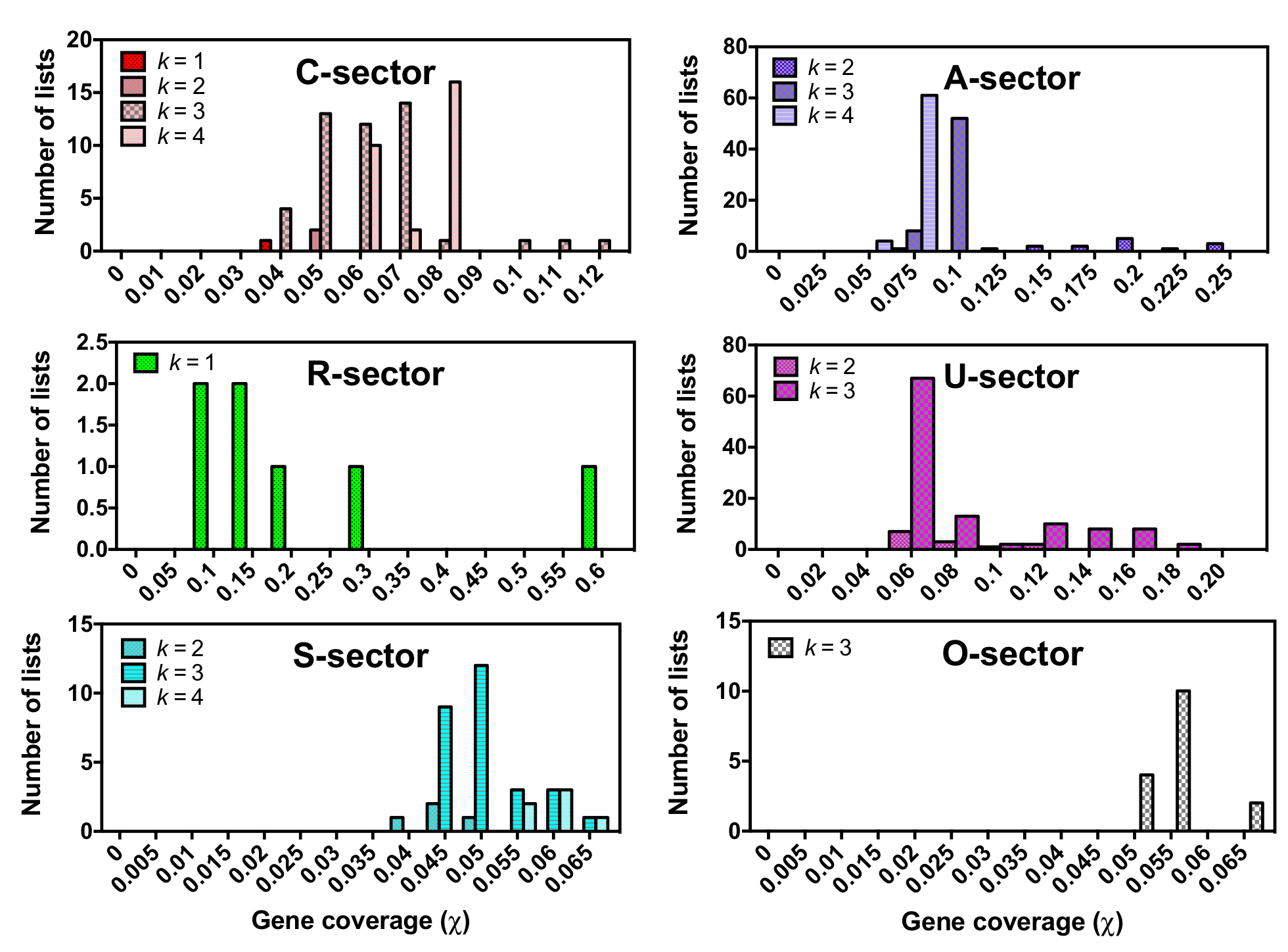
**

Figure T3-4. Histograms of the gene coverage for GO lists.

**Top lists of GO terms for each sector**

In Fig T3-4, the histograms for the C-, A-, R-, and U- sectors have “long tails”, which means that the lists on the right side of the distribution are clearly better than the other lists. Table T3-5 shows a few lists with top *χ* values for each of the four sectors. The representing list (highlighted in light orange color in Table T3-5) for each sector is then picked from these small number of lists.

|  | ***ω*** | **O** | ***χ*** | **GO name** | | | ***ω* of individual GO terms** | | |
| --- | --- | --- | --- | --- | --- | --- | --- | --- | --- |
| **C** | 0.74 | 0.002 | 0.12 | tricarboxylic acid cycle | ion transport | locomotion | 0.19 | 0.36 | 0.19 |
|  | 0.66 | 0.003 | 0.11 | tricarboxylic acid cycle | ion transport | chemotaxis | 0.19 | 0.36 | 0.12 |
|  | 0.67 | 0.003 | 0.10 | tricarboxylic acid cycle | ion transport | response to external stimulus | 0.19 | 0.36 | 0.12 |
| **A** | 0.60 | 0.000 | 0.26 | glucose catabolic process | cellular amino acid metabolic process |  | 0.16 | 0.44 |  |
|  | 0.60 | 0.000 | 0.25 | glucose metabolic process | cellular amino acid metabolic process |  | 0.16 | 0.44 |  |
|  | 0.60 | 0.000 | 0.25 | cellular amino acid metabolic process | hexose catabolic process |  | 0.44 | 0.16 |  |
|  | 0.60 | 0.000 | 0.23 | cellular amino acid metabolic process | hexose metabolic process |  | 0.44 | 0.16 |  |
|  | 0.60 | 0.000 | 0.21 | cellular amino acid metabolic process | carbohydrate catabolic process |  | 0.44 | 0.16 |  |
|  | 0.60 | 0.000 | 0.20 | glucose catabolic process | organonitrogen compound biosynthetic process |  | 0.16 | 0.44 |  |
|  | 0.60 | 0.000 | 0.20 | glucose metabolic process | organonitrogen compound biosynthetic process |  | 0.16 | 0.44 |  |
|  | 0.61 | 0.000 | 0.20 | hexose catabolic process | organonitrogen compound biosynthetic process |  | 0.16 | 0.44 |  |
|  | 0.61 | 0.000 | 0.19 | hexose metabolic process | organonitrogen compound biosynthetic process |  | 0.16 | 0.44 |  |
|  | 0.61 | 0.000 | 0.18 | carbohydrate catabolic process | organonitrogen compound biosynthetic process |  | 0.16 | 0.44 |  |
|  | 0.61 | 0.000 | 0.17 | cellular amino acid metabolic process | single-organism carbohydrate metabolic process |  | 0.44 | 0.16 |  |
|  | 0.61 | 0.000 | 0.15 | carbohydrate metabolic process | cellular amino acid metabolic process |  | 0.16 | 0.44 |  |
|  | 0.61 | 0.009 | 0.15 | single-organism carbohydrate metabolic process | organonitrogen compound biosynthetic process |  | 0.16 | 0.44 |  |
|  | 0.61 | 0.009 | 0.13 | carbohydrate metabolic process | organonitrogen compound biosynthetic process |  | 0.16 | 0.44 |  |
| **R** | 0.73 | 0.000 | 0.59 | translation |  |  |  |  |  |
|  | 0.61 | 0.003 | 0.17 | regulation of translation | purine ribonucleotide biosynthetic process | alpha-amino acid biosynthetic process | 0.11 | 0.12 | 0.39 |
|  | 0.62 | 0.002 | 0.17 | purine ribonucleotide biosynthetic process | posttranscriptional regulation of gene expression | alpha-amino acid biosynthetic process | 0.12 | 0.11 | 0.39 |
|  | 0.62 | 0.003 | 0.17 | regulation of translation | purine-containing compound biosynthetic process | alpha-amino acid biosynthetic process | 0.11 | 0.13 | 0.39 |
|  | 0.62 | 0.002 | 0.17 | posttranscriptional regulation of gene expression | purine-containing compound biosynthetic process | alpha-amino acid biosynthetic process | 0.11 | 0.13 | 0.39 |
|  | 0.62 | 0.003 | 0.16 | regulation of translation | cellular amino acid biosynthetic process | purine ribonucleotide biosynthetic process | 0.11 | 0.39 | 0.12 |
|  | 0.62 | 0.002 | 0.15 | cellular amino acid biosynthetic process | purine ribonucleotide biosynthetic process | posttranscriptional regulation of gene expression | 0.39 | 0.12 | 0.11 |
|  | 0.60 | 0.003 | 0.15 | regulation of translation | cellular amino acid biosynthetic process | purine ribonucleoside monophosphate biosynthetic process | 0.11 | 0.39 | 0.11 |
|  | 0.62 | 0.003 | 0.15 | regulation of translation | cellular amino acid biosynthetic process | purine-containing compound biosynthetic process | 0.11 | 0.39 | 0.13 |
|  | 0.60 | 0.002 | 0.15 | cellular amino acid biosynthetic process | purine ribonucleoside monophosphate biosynthetic process | posttranscriptional regulation of gene expression | 0.39 | 0.11 | 0.11 |
|  | 0.62 | 0.002 | 0.15 | cellular amino acid biosynthetic process | posttranscriptional regulation of gene expression | purine-containing compound biosynthetic process | 0.39 | 0.11 | 0.13 |

Table T3-5. Lists of GO terms for the C-, A-, R-, and U- sectors as reached by the searching procedure.

The histograms for the S-sector (Fig T3-4) only shows a short tail. We list in Table T3-6 all the lists with *χ_S,l_* > 0.05. The representing list is again highlighted in light orange color.

| ***ω*** | **O** | ***χ*** | **GO name** | | | | ***ω* of individual GO terms** | | | |
| --- | --- | --- | --- | --- | --- | --- | --- | --- | --- | --- |
| 0.63 | 0.021 | 0.06 | response to stress | carboxylic acid metabolic process | organic substance transport |  | 0.13 | 0.37 | 0.13 |  |
| 0.60 | 0.042 | 0.06 | glucose metabolic process | response to stress | monocarboxylic acid metabolic process | single-organism transport | 0.14 | 0.13 | 0.18 | 0.15 |
| 0.64 | 0.036 | 0.06 | carbohydrate metabolic process | response to stress | organic substance transport |  | 0.38 | 0.13 | 0.13 |  |
| 0.61 | 0.045 | 0.06 | response to stress | electron transport chain | organic substance transport | organic substance catabolic process | 0.13 | 0.12 | 0.13 | 0.24 |
| 0.60 | 0.042 | 0.06 | monosaccharide metabolic process | response to stress | monocarboxylic acid metabolic process | single-organism transport | 0.14 | 0.13 | 0.18 | 0.15 |
| 0.61 | 0.000 | 0.06 | carboxylic acid metabolic process | cellular response to stimulus | organic substance transport |  | 0.37 | 0.11 | 0.13 |  |
| 0.65 | 0.042 | 0.06 | response to stress | carboxylic acid metabolic process | single-organism transport |  | 0.13 | 0.37 | 0.15 |  |
| 0.61 | 0.042 | 0.06 | response to stress | monocarboxylic acid metabolic process | dicarboxylic acid metabolic process | single-organism transport | 0.13 | 0.18 | 0.15 | 0.15 |
| 0.61 | 0.048 | 0.06 | response to stress | monocarboxylic acid metabolic process | single-organism carbohydrate metabolic process | single-organism transport | 0.13 | 0.18 | 0.15 | 0.15 |
| 0.60 | 0.042 | 0.06 | glyoxylate cycle | response to stress | dicarboxylic acid metabolic process | single-organism transport | 0.17 | 0.13 | 0.15 | 0.15 |
| 0.61 | 0.018 | 0.06 | carbohydrate metabolic process | cellular response to stimulus | organic substance transport |  | 0.38 | 0.11 | 0.13 |  |
| 0.63 | 0.025 | 0.05 | carboxylic acid metabolic process | single-organism transport | cellular response to stimulus |  | 0.37 | 0.15 | 0.11 |  |
| 0.60 | 0.042 | 0.05 | response to stress | cellular carbohydrate metabolic process | single-organism transport |  | 0.13 | 0.32 | 0.15 |  |
| 0.63 | 0.044 | 0.05 | carbohydrate metabolic process | single-organism transport | cellular response to stimulus |  | 0.38 | 0.15 | 0.11 |  |
| 0.63 | 0.006 | 0.05 | glucose metabolic process | monocarboxylic acid metabolic process | single-organism process |  | 0.14 | 0.18 | 0.31 |  |

Table T3-6. Lists of GO terms for the S-sector reached by the searching procedure.

The histogram for the O-sector (Fig T3-4) shows that all lists have similar values of *χ*. For the O-sector, we list all of them in Table T3-7. Again, the representing list is highlighted in light orange color.

| ***ω*** | **O** | ***χ*** | **GO name** | | | ***ω* of individual GO terms** | | |
| --- | --- | --- | --- | --- | --- | --- | --- | --- |
| 0.60 | 0.006 | 0.06 | macromolecule biosynthetic process | carboxylic acid biosynthetic process | organic substance transport | 0.29 | 0.21 | 0.10 |
| 0.61 | 0.005 | 0.06 | macromolecule biosynthetic process | small molecule biosynthetic process | organic substance transport | 0.29 | 0.22 | 0.10 |
| 0.64 | 0.006 | 0.06 | macromolecule biosynthetic process | single-organism transport | carboxylic acid biosynthetic process | 0.29 | 0.13 | 0.21 |
| 0.67 | 0.006 | 0.06 | transport | macromolecule biosynthetic process | carboxylic acid biosynthetic process | 0.17 | 0.29 | 0.21 |
| 0.64 | 0.005 | 0.06 | macromolecule biosynthetic process | small molecule biosynthetic process | single-organism transport | 0.29 | 0.22 | 0.13 |
| 0.68 | 0.005 | 0.06 | transport | macromolecule biosynthetic process | small molecule biosynthetic process | 0.17 | 0.29 | 0.22 |
| 0.60 | 0.009 | 0.06 | RNA metabolic process | single-organism transport | carboxylic acid biosynthetic process | 0.26 | 0.13 | 0.21 |
| 0.64 | 0.009 | 0.06 | transport | RNA metabolic process | carboxylic acid biosynthetic process | 0.17 | 0.26 | 0.21 |
| 0.61 | 0.009 | 0.06 | RNA metabolic process | small molecule biosynthetic process | single-organism transport | 0.26 | 0.22 | 0.13 |
| 0.64 | 0.009 | 0.06 | transport | RNA metabolic process | small molecule biosynthetic process | 0.17 | 0.26 | 0.22 |
| 0.71 | 0.009 | 0.05 | macromolecule metabolic process | single-organism transport | carboxylic acid biosynthetic process | 0.37 | 0.13 | 0.21 |
| 0.72 | 0.009 | 0.05 | macromolecule metabolic process | small molecule biosynthetic process | single-organism transport | 0.37 | 0.22 | 0.13 |
| 0.64 | 0.009 | 0.05 | transport | carboxylic acid biosynthetic process | nucleic acid metabolic process | 0.17 | 0.21 | 0.26 |
| 0.64 | 0.009 | 0.05 | transport | small molecule biosynthetic process | nucleic acid metabolic process | 0.17 | 0.22 | 0.26 |
| 0.60 | 0.009 | 0.05 | single-organism transport | carboxylic acid biosynthetic process | nucleic acid metabolic process | 0.13 | 0.21 | 0.26 |
| 0.61 | 0.009 | 0.05 | small molecule biosynthetic process | single-organism transport | nucleic acid metabolic process | 0.22 | 0.13 | 0.26 |

Table T3-7. Lists of the GO terms for the O-sector by the searching procedure.

**Lists of genes for the representing list of each sector**

See Table S5 (in a separate Excel file) for lists of genes for the representing GO term lists.

The Matlab code for implementing the procedure is available as Supplementary Code.

# Supplementary Text S4

**Microarray studies in *S. cerevisiae***

A number of studies over the last decade have carefully measured the growth rate dependence of mRNA transcript levels, proteins, and metabolites in Baker's yeast under various nutrient limiting conditions in chemostat, e.g., (Airold*i et* al, 2009; Lev*y et* al, 2007; Braue*r et* al, 2008; Regenber*g et* al, 2006; Castrill*o et* al, 2007). Given their complementary focus, we feel it is important to discuss these early works. We point out, for reasons fully explained in Supplementary Text 5, that changes in the abundance of any given mRNA should not be taken as a straight measure of the abundance of the corresponding protein. With that caveat in mind, we now compare the general conclusions reached by the various studies.

A common finding between all the studies is a positive correlation between ribosomal proteins and the growth rate *λ*. These results are unsurprising, and likely reflect the obligatory relationship between ribosome levels and growth rate outside of ribosome limiting conditions (e.g. chloramphenicol) which were not probed in these studies. Notably, Levy *et al* report a general decrease in ribosomal protein mRNA synthesis rates as the cell nears the end of exponential growth and runs out of nutrients.

Castrillo *et al* and Regenberg *et al* report divergent behavior between functional gene classes as growth rate is varied by nutrient limitation. Focusing on the carbon limitation condition (set by chemostat control of glucose), both studies report groups of genes (by mRNA in Regenberg *et al*, by protein in Castrillo *et al*) that increase with growth rate, i.e. that are down-regulated by carbon limitation. Additionally, Castrillo *et al* report a large cluster of enzymes that correlate negatively with growth rate, i.e. that are specifically up-regulated with increasing carbon limitation. This class consists largely of proteins employed in cellular carbohydrate metabolism, cellular macromolecule catabolism, transport, and response to stress. This finding is in good agreement with our C and S sectors which exhibit a similar general trajectory under carbon limitation, and are dominated by similar descriptive terms in our GO analysis (ion transport, tricarboxylic acid cycle, carbohydrate metabolic process, and response to stress).

Upon casual inspection, the protein measurements in Castrillo *et al* appear to contradict the findings of Regenberg *et al*, who report only one cluster (Cluster 13) that increases upon carbon limitation, and thirteen that increase or have no clear trend. However, the authors note that a number of ORFs were found to decrease linearly with growth rate and that the entire dataset was normalized such that a small subset (42) of these ORFs would exhibit growth rate independent behavior. With this information in hand, Clusters 8 through 10 (which exhibit no strong relationship to *λ*) likely decrease with growth rate. Inspecting the dominant GO terms for these Clusters, we find transport, carboxylic acid metabolism, main pathways of carbohydrate metabolism, and energy pathways. Moreover, the most dominant GO term in Cluster 13 is reported as autophagy, a classic stress response. Thus, upon correcting for the normalization, we find that clusters in Regenberg *et al* downregulated by carbon limitation largely correspond to those reported in Castrillo *et al*, as well as to our C and S sectors.

Stressing the strong case for skepticism in equating trends at the transcript and protein levels, as discussed in (Klump*p et* al, 2009), the studies tend to reinforce one another in the carbon limitation case. It would be valuable to look more deeply at the response for carbon, and nitrogen limitation reported in *S. cerevisiae* and *E. coli*, as well as for other limitations (e.g. ribosome slowing, sulfur, phosphate).

Airoldi *et al* focus on the inference problem of predicting growth rate from relative gene expression levels, i.e. the backwards problem of our study. For simplicity, they exclude genes that have non-uniform correlation with *λ* across differing nutrient limitations (in our study the R, U, and S sectors harbor such genes, when the ribosome slowing limitation is excluded). They find that a linear model can accurately predict cellular growth rate from the measurement of a small set of reporter genes. This comports with our finding that the majority of proteins change linearly with *λ* in a characteristic fashion.

Finally, Brauer *et al* study the growth rate dependence of the Yeast transcriptome across six major nutrient limitations. We focus here on the glucose and ammonia limitations. The authors find that ~60% of the variance can be explained by 3 “eigengenes”: two that decrease upon every limitation, and another that increases upon every limitation. Focusing on the nutrient limitations common with our study, the eigengenes of the first case would encompass the behavior of the R and U sectors, while the second case would describe our S sector. Strikingly, there does not appear to be a major eigengene with opposite behavior in the glucose, and nitrogen limiting conditions as we find with the prominent C and A sectors in *E. coli*. As with the other studies, Brauer *et al* report the positive correlation between ribosomal genes and *λ*.

# Supplementary Text S5

**Proteome fraction as a useful quantitative measure**

**Proteome fraction vs. copy number**

In this work, we measure the proteome fraction of each gene, given by $M_{i}/M_{T}$, where $M_{i}$is the mass of enzyme *i* within the cell, and $M_{T}$ is the total mass of protein within the cell. We suggest that this is a profitable measure of protein abundance.
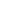


In particular, the proteome fraction of a protein is directly proportional to its concentration. To see this, we begin by pointing out that the total mass of protein in a cell scales directly with the cell volume, i.e. $M_{T}\sim V$. This is known from the facts that (i) protein mass is the dominant component of a cell’s dry mass, and (ii) the mass:water ratio of a cell is growth-rate independent as determined by buoyant density (Nanninga & Woldringh, 1985). Clearly, the mass $M_{i}$of a protein is proportional to its copy number $N_{i}$, and so, $M_{i}/M_{T}\propto N_{i}/V=c_{i}$. In other words, the proteome fraction of an enzyme is directly proportional to concentration of the enzyme ($c_{i}$) in the cell.

In addition to its usefulness, the relative change in proteome fraction is easy to measure using mass spectrometry. To measure the change in proteome fraction for a given protein (and thus its change in concentration) across a series of conditions, all one must do is mix together equal amounts of experimental and reference proteome. To see this, suppose we have an amount $M^{r}$of reference proteome, and $M^{e}$of experimental proteome. In the reference, protein *i* makes up some fraction $f_{i}^{r}$of the proteome by mass, and the fraction $f_{i}^{e}$in the experimental sample. If we combine the samples, and measure the relative level of protein *i* in either condition, we obtain $M_{i}^{e}/M_{i}^{r}=f_{i}^{e}M^{e}/f_{i}^{r}M^{r}$. Therefore, as long as we mix equal amounts of total protein, we have $M_{i}^{e}/M_{i}^{r}=f_{i}^{e}/f_{i}^{r}$.

We note that proteome fraction answers the call issued recently by Milo for accurate quantitative measures of protein copy number per cell volume (Milo, 2013).

**Proteome fraction crucial for coarse graining**

Historically, most gene expression studies have focused on measuring mRNA transcript levels because i.) the ability to quantify mRNA levels en masse was developed before a convenient proteomic equivalent, and ii.) mRNA transcript abundance was long taken as a good proxy for its corresponding enzyme product. However, when cells are grown at the limit of translational capacity i.e. when each ribosome occupies an mRNA (as in our limiting conditions), mRNA transcripts are in competition for a small number of free ribosomes, and initiation is strongly influenced by the sequence of the ribosome binding site, and mRNA secondary structure (Kudl*a et* al, 2009). Hence, relative mRNA levels need not reflect relative protein levels. Indeed, global correlation studies have found low to moderate correlation between mRNA and protein levels (Maie*r et* al, 2009). Concretely, the probability of translation initiation is a product of mRNA concentration, and a sequence dependent term characterizing the interaction efficiency between the mRNA and the ribosome, $p_{i}\propto mRNA_{i}\times\beta(s_{i})$. For this reason, we would not have been able to conduct our coarse-graining analysis on the basis of mRNA quantification (e.g. deep sequencing, microarray).

This stands in contrast to techniques such as spectral counting, and ribosome profiling which measure protein abundance. By definition, the proteome fraction of a given enzyme reflects the fraction of proteome resources that the cell devotes to its production. Therefore, questions of expression burdens, and bottlenecks may be addressed quantitatively using this measure.

**Noise comparison (ribosome example)**

Here we show that mass spectrometry affords superior accuracy in measurement to mRNA microarrays. Although microarrays afford wider coverage, they are prone to well known sources of noise, including sequence specific mRNA:dye interactions, variations in reverse transcription efficiencies, etc. that limit the interpretation of observed changes. Thus, even if one were to assume perfect correspondence between actual mRNA copy numbers and protein level (specious, see above), on the basis of measurement quality, mass spectrometry should be used for quantitative studies when possible.

To compare the quantitative nature of both measurements, we compared the relative concentration of ribosomal proteins as a function of growth rate in *S. cerevisiae* as measured with microarray and in *E. coli* using MS. We focus on the measurements of Brauer et al. (Braue*r et* al, 2008), which we take as an exemplary microarray experiment.

Though we cannot expect identical behavior in both organisms, it is reasonable to expect that ribosomal genes exhibit similar levels of cohesion given the stoichiometry of ribosome composition in both organisms. Below in Fig T4-1 we display the relative level of each ribosomal protein (colored curves), along with the median behavior for (black points) in each organism as λ is changed by carbon limitation. It is clear that the signal to noise ratio is much higher for the *E. coli* measurement than for the *S. cerevisiae*. This difference cannot easily be explained by differences between the organisms as ribosomal protein expression in *S. cerevisiae* is tightly regulated (Tana*y et* al, 2005; Bosi*o et* al, 2014). It is more likely that this reflects a difference in measurement technique.


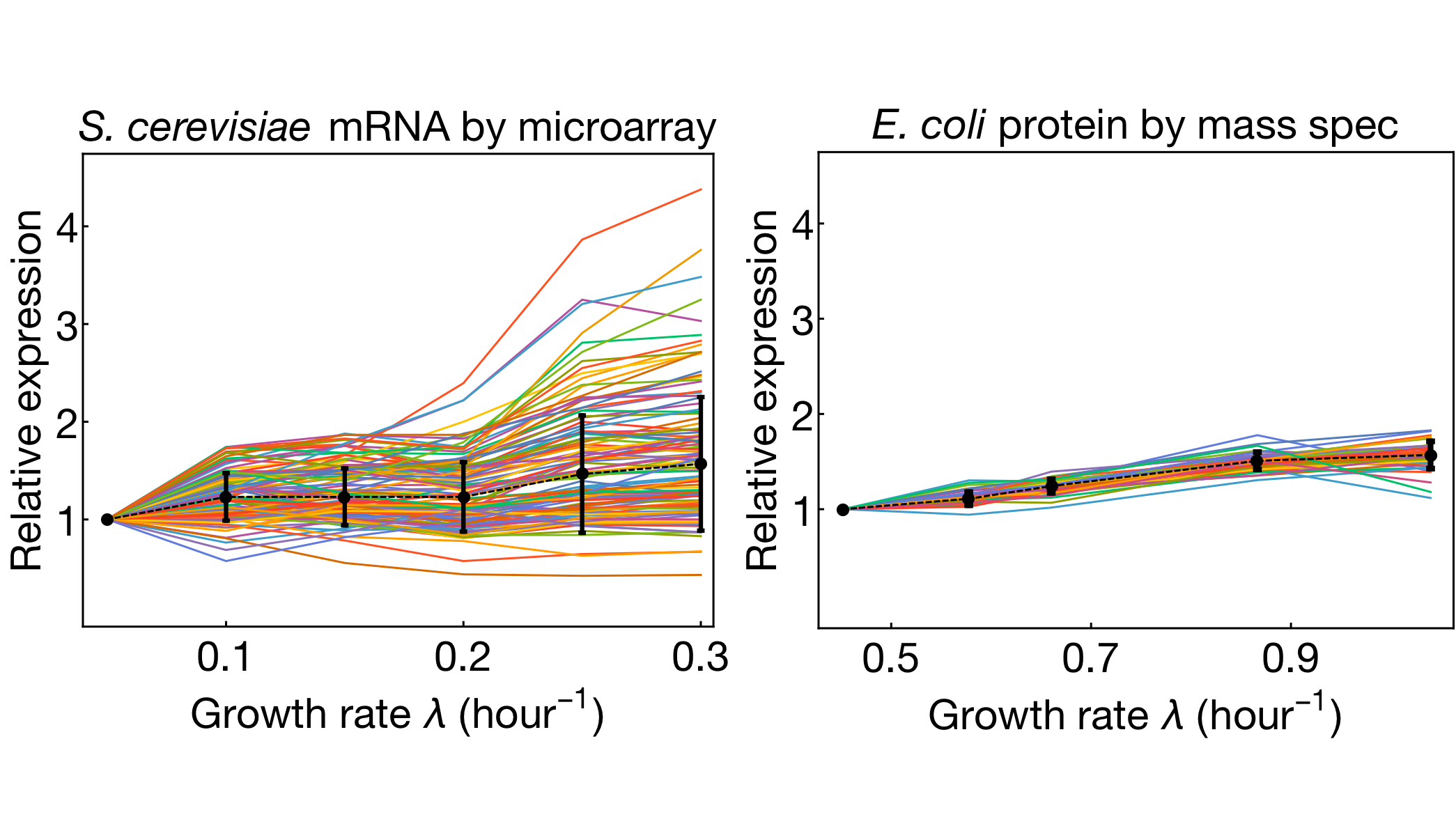


Figure T4-1. Measurements of relative mRNA abundance in *S. cerevisiae* (left plot) and relative protein abundance in *E. coli* (right plot). Growth rate in *E. coli* is set by titrating the level of lactose importer, while in *S. cerevisiae* it is set by the chemostat influx rate with glucose as the limiting nutrient.

# Supplementary Dataset S1

**A zip folder including plots for individual proteins under the three growth limitations.**

# Supplementary Code

**A zip folder including annotated Matlab Scripts for implementing the Gene Ontology enrichment analysis in Supplementary Text S3.**

# References

Airoldi EM, Huttenhower C, Gresham D, Lu C, Caudy AA, Dunham MJ, Broach JR, Botstein D & Troyanskaya OG (2009) Predicting Cellular Growth from Gene Expression Signatures. *PLoS Comput Biol* **5:** e1000257

Bosio MC, Negri R & Dieci G (2014) Promoter architectures in the yeast ribosomal expression program. *Transcription* **2:** 71–77

Brauer M, Huttenhower C, Airoldi E, Rosenstein R, Matese J, Gresham D, Boer V, Troyanskaya O & Botstein D (2008) Coordination of Growth Rate, Cell Cycle, Stress Response, and Metabolic Activity in Yeast. *Molecular Biology of the Cell* **19:** 352

Castrillo JI, Zeef LA, Hoyle DC, Zhang N, Hayes A, Gardner DC, Cornell MJ, Petty J, Hakes L, Wardleworth L, Rash B, Brown M, Dunn WB, Broadhurst D, O'Donoghue K, Hester SS, Dunkley TP, Hart SR, Swainston N, Li P, et al (2007) Growth control of the eukaryote cell: a systems biology study in yeast. *J Biol* **6:** 4

Cayley S, Record MT & Lewis BA (1989) Accumulation of 3-(N-morpholino)propanesulfonate by osmotically stressed Escherichia coli K-12. *Journal of Bacteriology* **171:** 3597–3602

Cherepanov PP & Wackernagel W (1995) Gene disruption in Escherichia coli: TcR and KmR cassettes with the option of Flp-catalyzed excision of the antibiotic-resistance determinant. *Gene* **158:** 9–14

Datsenko KA & Wanner BL (2000) One-step inactivation of chromosomal genes in Escherichia coli K-12 using PCR products. *Proceedings of the National Academy of Sciences* **97:** 6640–6645

de Lorenzo V, Herrero M, Metzke M & Timmis KN (1991) An upstream XylR- and IHF-induced nucleoprotein complex regulates the sigma 54-dependent Pu promoter of TOL plasmid. *EMBO J* **10:** 1159–1167

Klumpp S, Zhang Z & Hwa T (2009) Growth Rate-Dependent Global Effects on Gene Expression in Bacteria. *Cell* **139:** 1366–1375

Kudla G, Murray AW, Tollervey D & Plotkin JB (2009) Coding-sequence determinants of gene expression in Escherichia coli. *Science* **324:** 255–258

Levy S, Ihmels J, Carmi M, Weinberger A, Friedlander G & Barkai N (2007) Strategy of Transcription Regulation in the Budding Yeast. *PLoS ONE* **2:** e250

Li G-W, Burkhardt D, Gross C & Weissman JS (2014) Quantifying Absolute Protein Synthesis Rates Reveals Principles Underlying Allocation of Cellular Resources. *Cell* **157:** 624–635

Lutz R & Bujard H (1997) Independent and tight regulation of transcriptional units in Escherichia coli via the LacR/O, the TetR/O and AraC/I1-I2 regulatory elements. *Nucleic Acids Research* **25:** 1203–1210

Lyons E, Freeling M, Kustu S, Inwood W & Idnurm A (2011) Using Genomic Sequencing for Classical Genetics in E. coli K12. *PLoS ONE* **6:** 12585–12592

Maaloe O (1979) Regulation of the protein synthesizing machinery - ribosomes, tRNA, factors, and so on. *Biological Regulation and Development Volume 1 - Gene expression***:** 1–56

Maier T, Güell M & Serrano L (2009) Correlation of mRNA and protein in complex biological samples. *FEBS Lett* **583:** 3966–3973

Miller JH (1972) Experiments in Molecular Genetics Cold Spring Harbor Laboratory Press

Milo R (2013) What is the total number of protein molecules per cell volume? A call to rethink some published values. *Bioessays* **35:** 1050–1055

Nanninga N & Woldringh CL (1985) Cell growth, genome duplication, and cell division. In pp 1–31.

Neidhardt FC, Bloch PL & Smith DF (1974) Culture medium for enterobacteria. *Journal of Bacteriology* **119:** 736–747

Okano H, Hwa T, Lenz P & Yan D (2010) Reversible Adenylylation of Glutamine Synthetase Is Dynamically Counterbalanced during Steady-State Growth of Escherichia coli. *J Mol Biol* **404:** 522–536

Pedersen S, Bloch P, Reeh S & Neidhardt F (1978) Patterns of protein synthesis in E. coli: a catalog of the amount of 140 individual proteins at different growth rates. *Cell* **14:** 179–190

Regenberg B, Grotkjaer T, Winther O, Fausbøll A, Akesson M, Bro C, Hansen LK, Brunak S & Nielsen J (2006) Growth-rate regulated genes have profound impact on interpretation of transcriptome profiling in Saccharomyces cerevisiae. *Genome Biol* **7:** R107

Schaechter M, Maaloe O & Kjeldgaard N (1958) Dependency on medium and temperature of cell size and chemical composition during balanced growth of Salmonella typhimurium. *Microbiology* **19:** 592

Scott M, Gunderson CW, Mateescu EM, Zhang Z & Hwa T (2010) Interdependence of cell growth and gene expression: origins and consequences. *Science* **330:** 1099–1102

Soupene E, van Heeswijk WC, Plumbridge J, Stewart V, Bertenthal D, Lee H, Prasad G, Paliy O, Charernnoppakul P & Kustu S (2003) Physiological studies of Escherichia coli strain MG1655: growth defects and apparent cross-regulation of gene expression. *Journal of Bacteriology* **185:** 5611–5626

Tanay A, Regev A & Shamir R (2005) Conservation and evolvability in regulatory networks: The evolution of ribosomal regulation in yeast. *Proceedings of the National Academy of Sciences* **102:** 7203–7208

Thomason LC, Costantino N & Court DL (2007) E. coli Genome Manipulation by P1 Transduction. In *Current protocols in molecular biology*, Ausubel FM Brent R Kingston RE Moore DD Seidman JG Smith JA & Struhl K (eds) Hoboken, NJ, USA: John Wiley & Sons, Inc.

You C, Okano H, Hui S, Zhang Z, Kim M, Gunderson CW, Wang Y-P, Lenz P, Yan D & Hwa T (2013) Coordination of bacterial proteome with metabolism by cyclic AMP signalling. *Nature* **500:** 301–306
