# Supplementary material for: Quantitative proteomic analysis reveals a simple strategy of global resource allocation in bacteria
Source: Mol Syst Biol. 2015 Feb 12;11(2):784. doi: 10.15252/msb.20145697 (PMC4358657; doi:10.15252/msb.20145697)
Supplement: Supplementary file 5 [file msb0011-0784-sd5.zip › Supplementary Dataset S1/Alim/all_Alim_plots.html]

publishmassacre 

Published with MATLAB® 7.9
