# Supplementary figures and images for: Quantitative proteomic analysis reveals a simple strategy of global resource allocation in bacteria (part 2 of 2)
Source: Mol Syst Biol. 2015 Feb 12;11(2):784. doi: 10.15252/msb.20145697 (PMC4358657; doi:10.15252/msb.20145697)

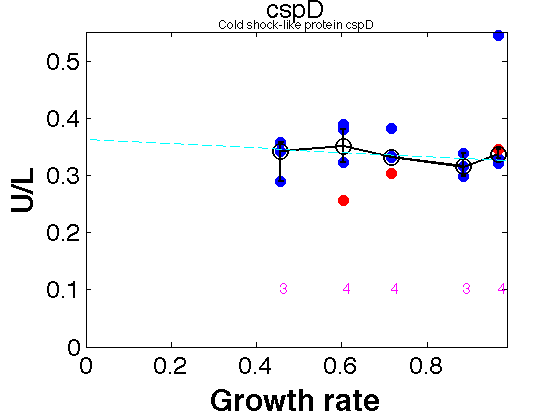

Supplement: Supplementary file 5 [file msb0011-0784-sd5.zip › Supplementary Dataset S1/Alim/cspD.png]

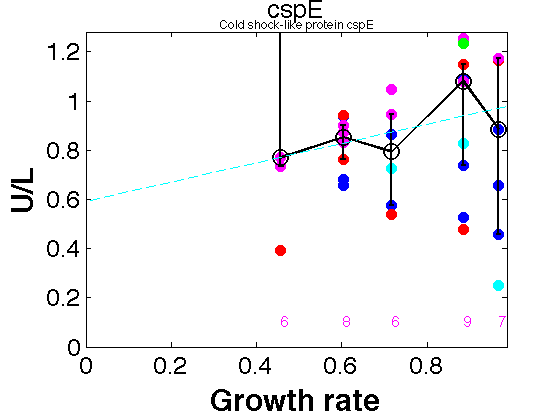

Supplement: Supplementary file 5 [file msb0011-0784-sd5.zip › Supplementary Dataset S1/Alim/cspE.png]

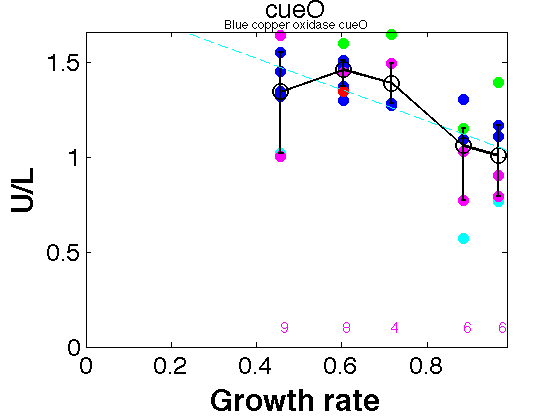

Supplement: Supplementary file 5 [file msb0011-0784-sd5.zip › Supplementary Dataset S1/Alim/cueO.png]

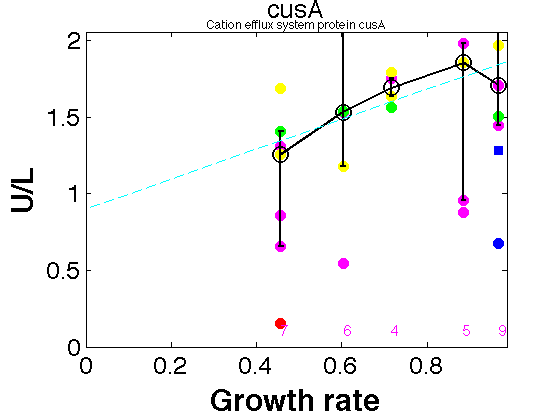

Supplement: Supplementary file 5 [file msb0011-0784-sd5.zip › Supplementary Dataset S1/Alim/cusA.png]

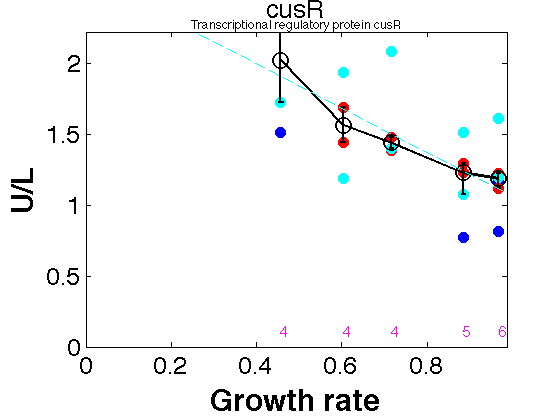

Supplement: Supplementary file 5 [file msb0011-0784-sd5.zip › Supplementary Dataset S1/Alim/cusR.png]

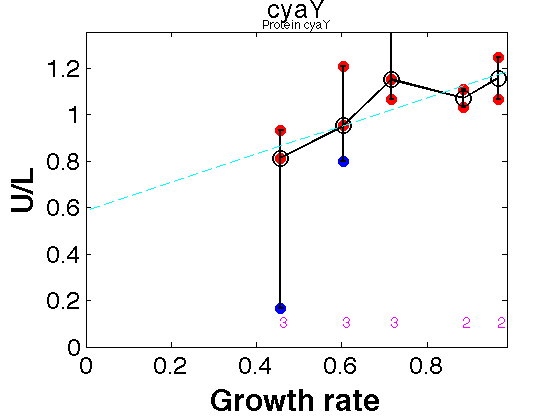

Supplement: Supplementary file 5 [file msb0011-0784-sd5.zip › Supplementary Dataset S1/Alim/cyaY.png]

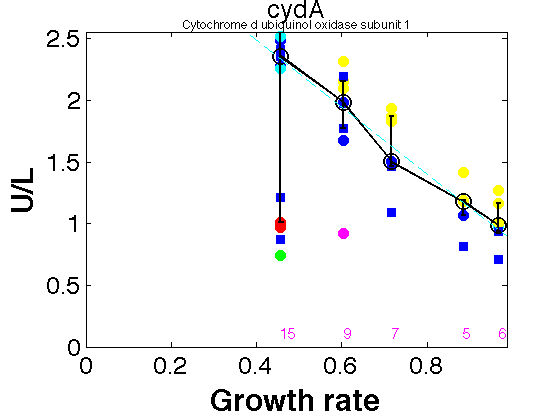

Supplement: Supplementary file 5 [file msb0011-0784-sd5.zip › Supplementary Dataset S1/Alim/cydA.png]

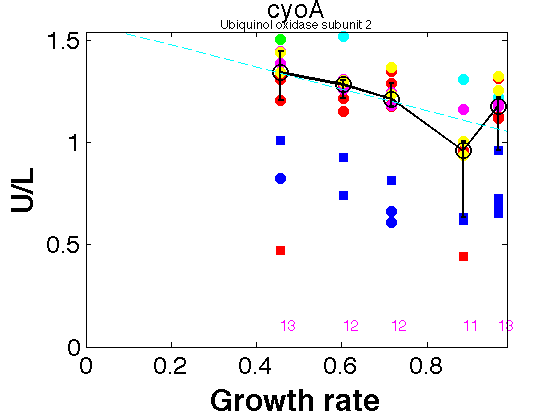

Supplement: Supplementary file 5 [file msb0011-0784-sd5.zip › Supplementary Dataset S1/Alim/cyoA.png]

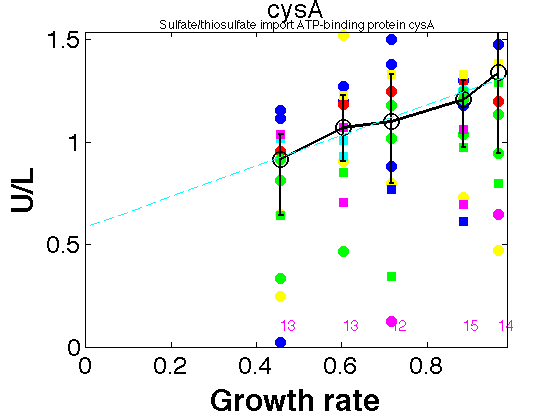

Supplement: Supplementary file 5 [file msb0011-0784-sd5.zip › Supplementary Dataset S1/Alim/cysA.png]

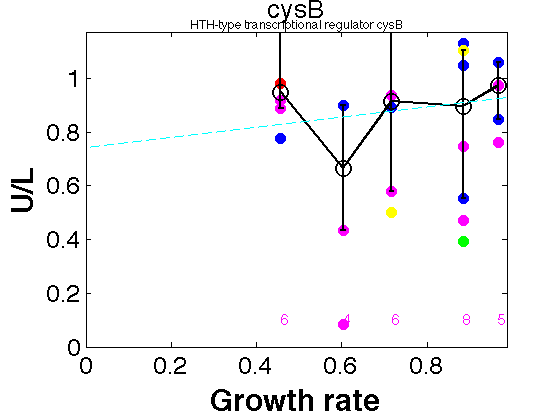

Supplement: Supplementary file 5 [file msb0011-0784-sd5.zip › Supplementary Dataset S1/Alim/cysB.png]

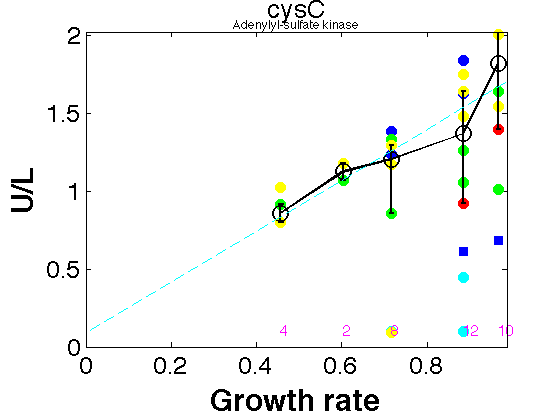

Supplement: Supplementary file 5 [file msb0011-0784-sd5.zip › Supplementary Dataset S1/Alim/cysC.png]

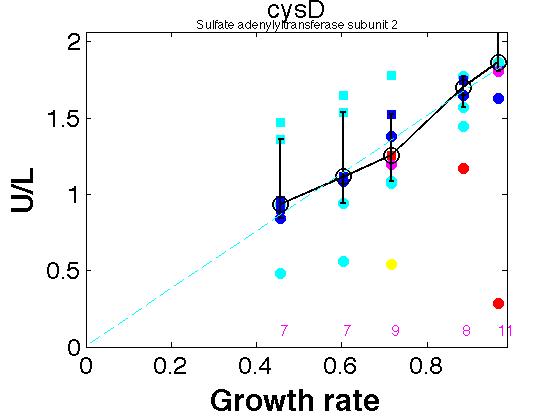

Supplement: Supplementary file 5 [file msb0011-0784-sd5.zip › Supplementary Dataset S1/Alim/cysD.png]

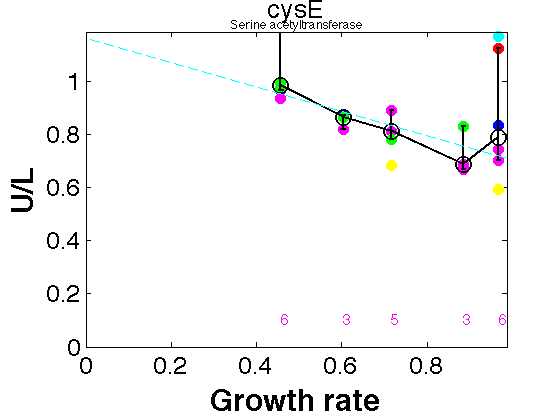

Supplement: Supplementary file 5 [file msb0011-0784-sd5.zip › Supplementary Dataset S1/Alim/cysE.png]

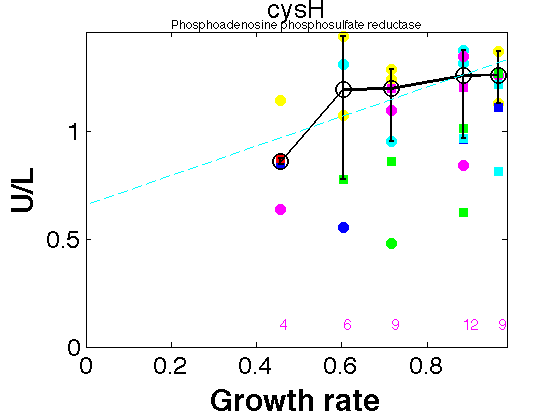

Supplement: Supplementary file 5 [file msb0011-0784-sd5.zip › Supplementary Dataset S1/Alim/cysH.png]

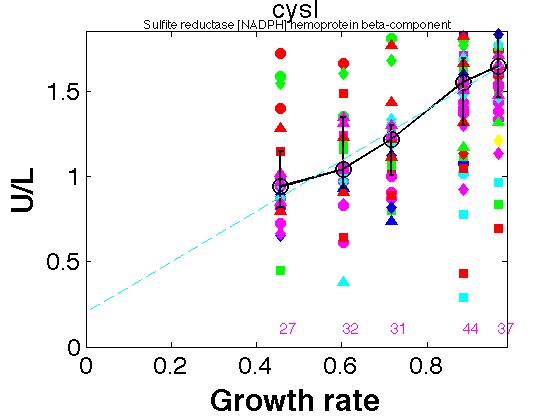

Supplement: Supplementary file 5 [file msb0011-0784-sd5.zip › Supplementary Dataset S1/Alim/cysI.png]

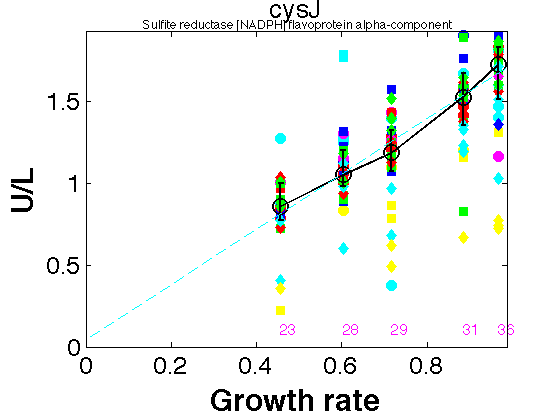

Supplement: Supplementary file 5 [file msb0011-0784-sd5.zip › Supplementary Dataset S1/Alim/cysJ.png]

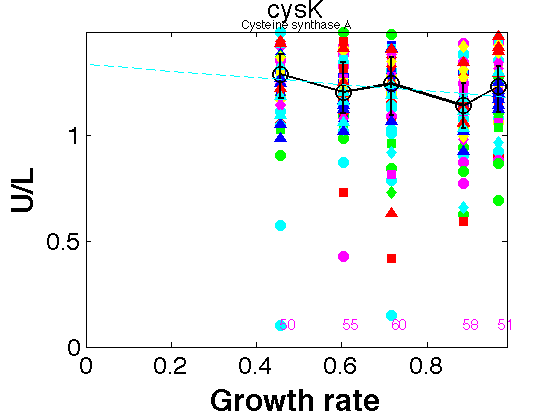

Supplement: Supplementary file 5 [file msb0011-0784-sd5.zip › Supplementary Dataset S1/Alim/cysK.png]

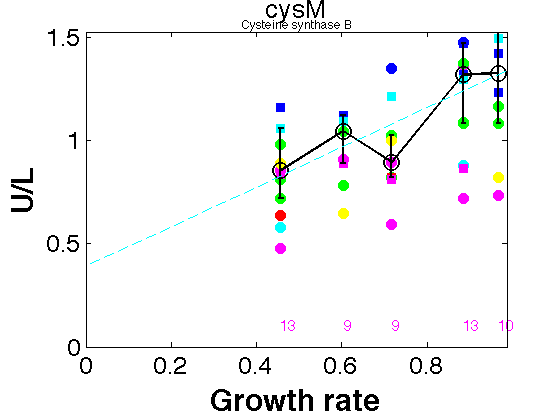

Supplement: Supplementary file 5 [file msb0011-0784-sd5.zip › Supplementary Dataset S1/Alim/cysM.png]

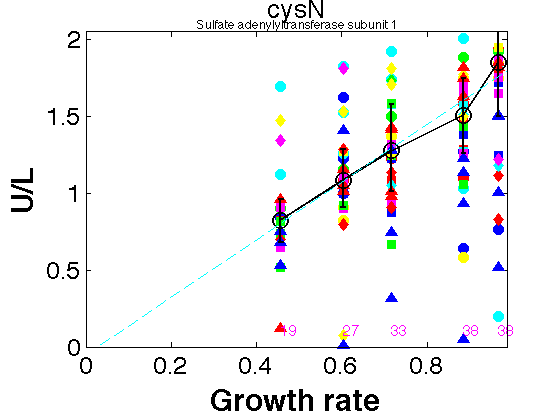

Supplement: Supplementary file 5 [file msb0011-0784-sd5.zip › Supplementary Dataset S1/Alim/cysN.png]

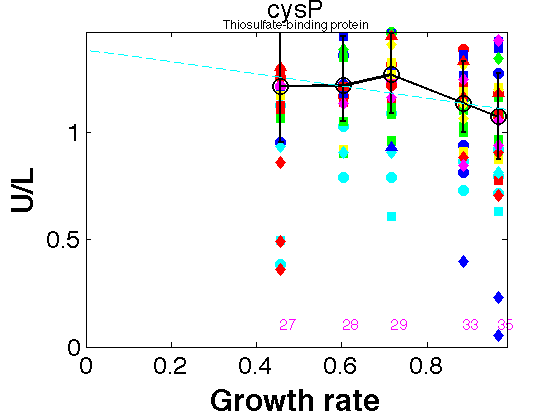

Supplement: Supplementary file 5 [file msb0011-0784-sd5.zip › Supplementary Dataset S1/Alim/cysP.png]

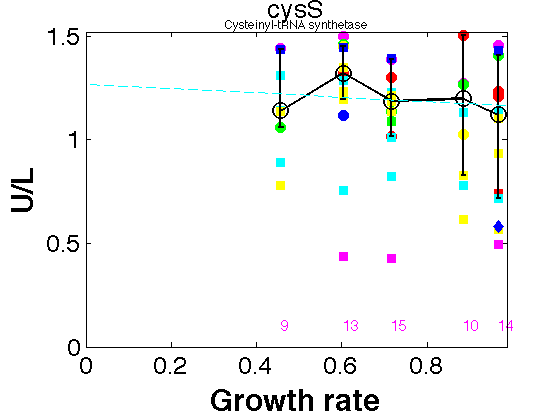

Supplement: Supplementary file 5 [file msb0011-0784-sd5.zip › Supplementary Dataset S1/Alim/cysS.png]

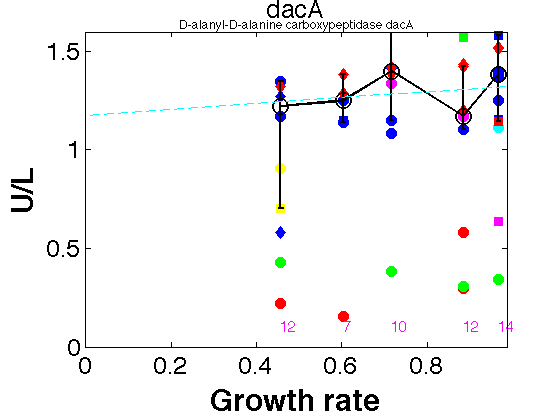

Supplement: Supplementary file 5 [file msb0011-0784-sd5.zip › Supplementary Dataset S1/Alim/dacA.png]

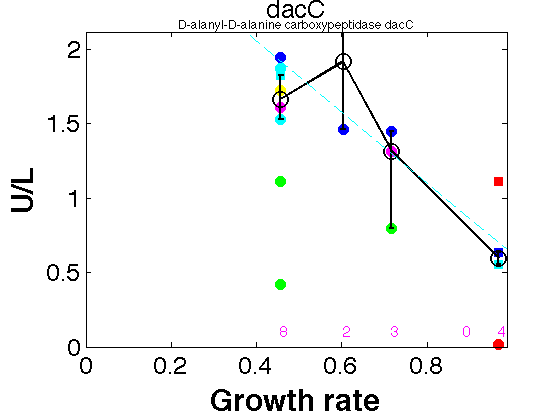

Supplement: Supplementary file 5 [file msb0011-0784-sd5.zip › Supplementary Dataset S1/Alim/dacC.png]

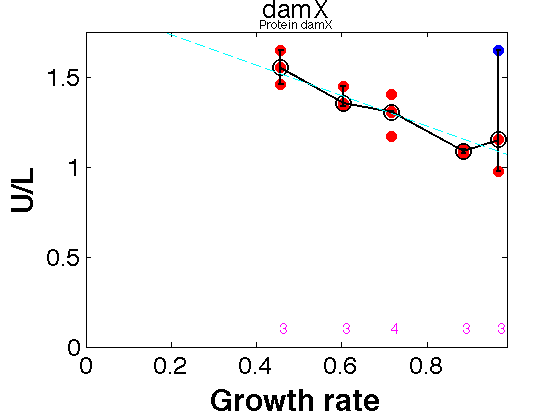

Supplement: Supplementary file 5 [file msb0011-0784-sd5.zip › Supplementary Dataset S1/Alim/damX.png]

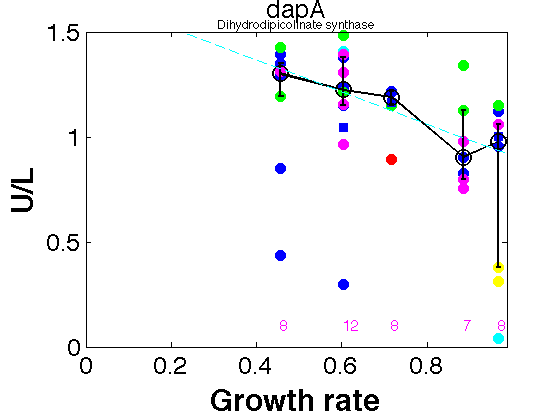

Supplement: Supplementary file 5 [file msb0011-0784-sd5.zip › Supplementary Dataset S1/Alim/dapA.png]

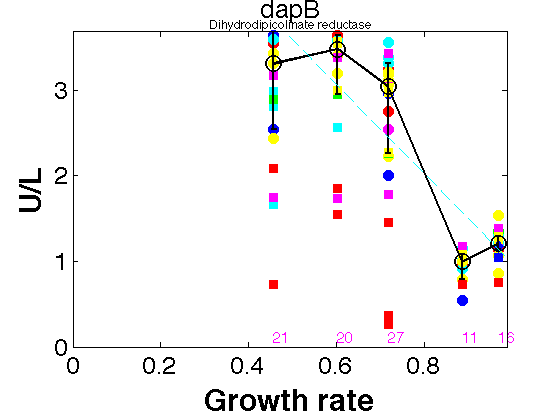

Supplement: Supplementary file 5 [file msb0011-0784-sd5.zip › Supplementary Dataset S1/Alim/dapB.png]

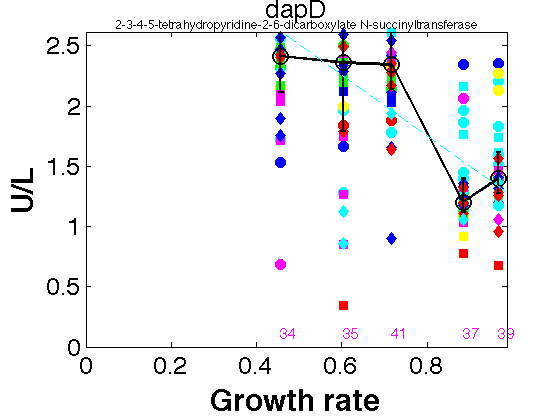

Supplement: Supplementary file 5 [file msb0011-0784-sd5.zip › Supplementary Dataset S1/Alim/dapD.png]

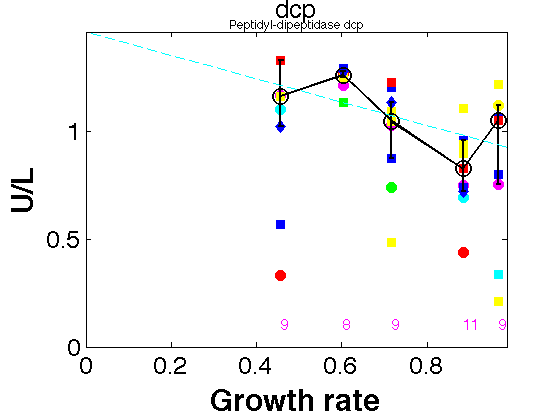

Supplement: Supplementary file 5 [file msb0011-0784-sd5.zip › Supplementary Dataset S1/Alim/dcp.png]

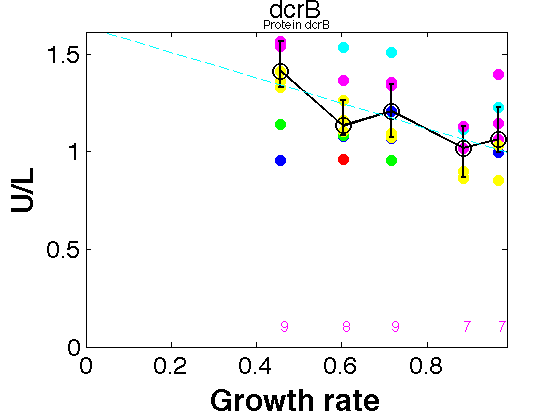

Supplement: Supplementary file 5 [file msb0011-0784-sd5.zip › Supplementary Dataset S1/Alim/dcrB.png]

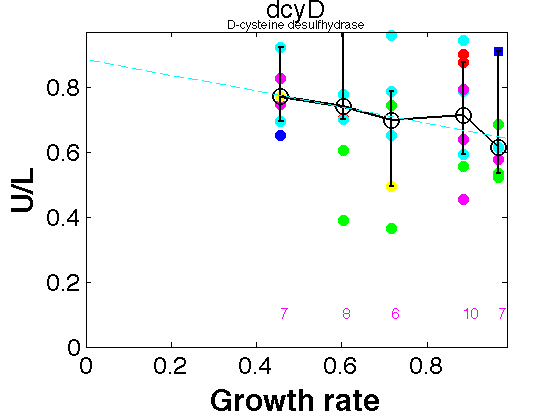

Supplement: Supplementary file 5 [file msb0011-0784-sd5.zip › Supplementary Dataset S1/Alim/dcyD.png]

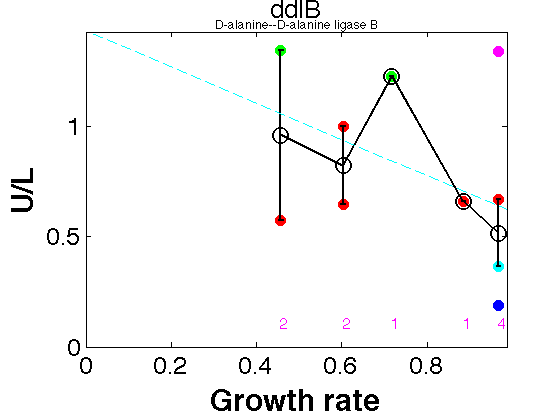

Supplement: Supplementary file 5 [file msb0011-0784-sd5.zip › Supplementary Dataset S1/Alim/ddlB.png]

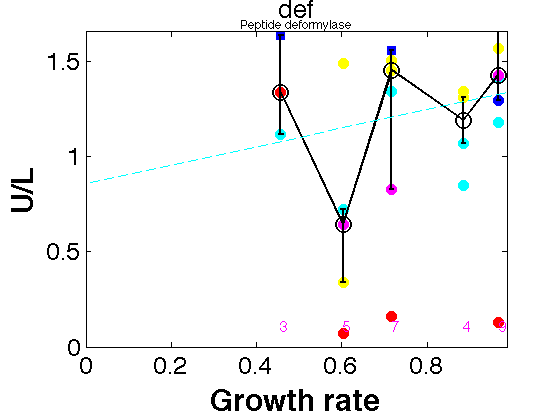

Supplement: Supplementary file 5 [file msb0011-0784-sd5.zip › Supplementary Dataset S1/Alim/def.png]

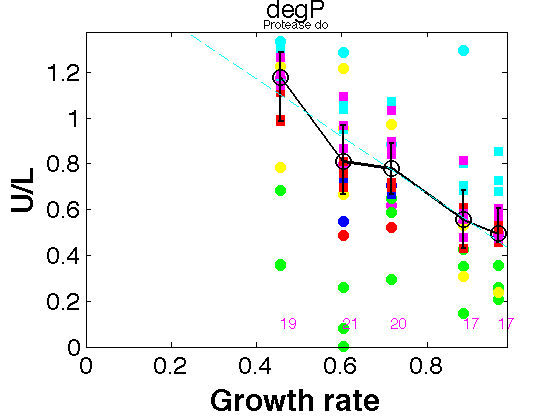

Supplement: Supplementary file 5 [file msb0011-0784-sd5.zip › Supplementary Dataset S1/Alim/degP.png]

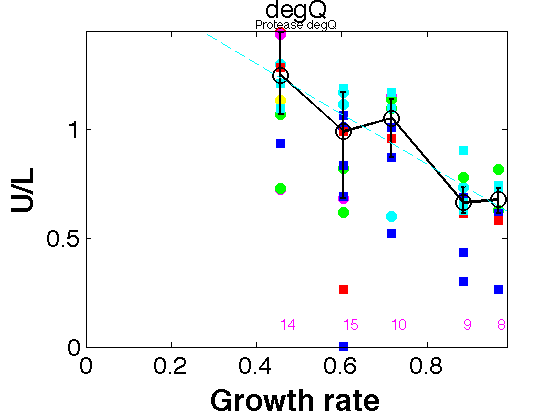

Supplement: Supplementary file 5 [file msb0011-0784-sd5.zip › Supplementary Dataset S1/Alim/degQ.png]

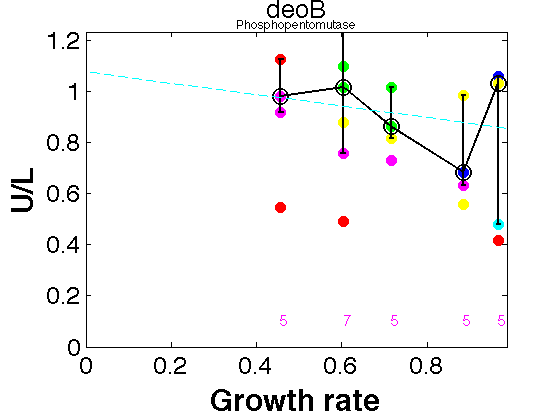

Supplement: Supplementary file 5 [file msb0011-0784-sd5.zip › Supplementary Dataset S1/Alim/deoB.png]

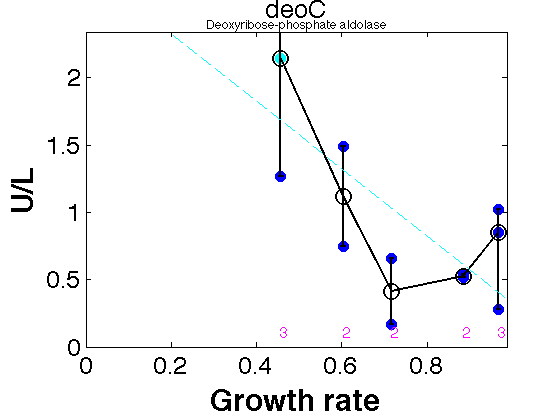

Supplement: Supplementary file 5 [file msb0011-0784-sd5.zip › Supplementary Dataset S1/Alim/deoC.png]

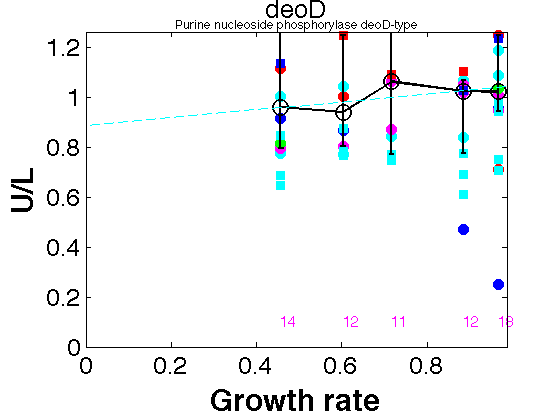

Supplement: Supplementary file 5 [file msb0011-0784-sd5.zip › Supplementary Dataset S1/Alim/deoD.png]

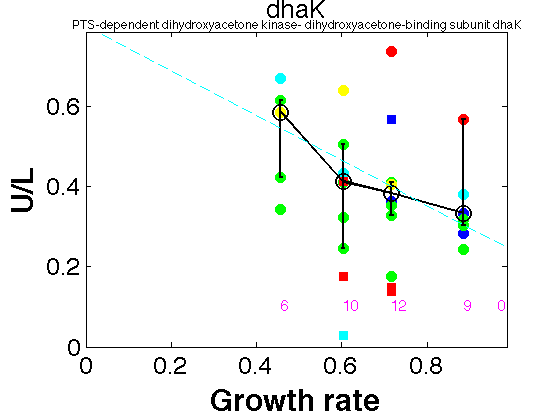

Supplement: Supplementary file 5 [file msb0011-0784-sd5.zip › Supplementary Dataset S1/Alim/dhaK.png]

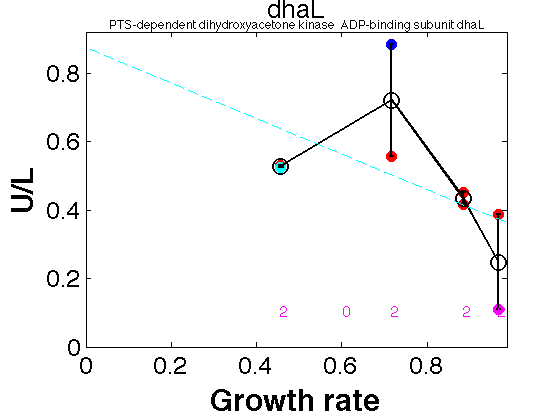

Supplement: Supplementary file 5 [file msb0011-0784-sd5.zip › Supplementary Dataset S1/Alim/dhaL.png]

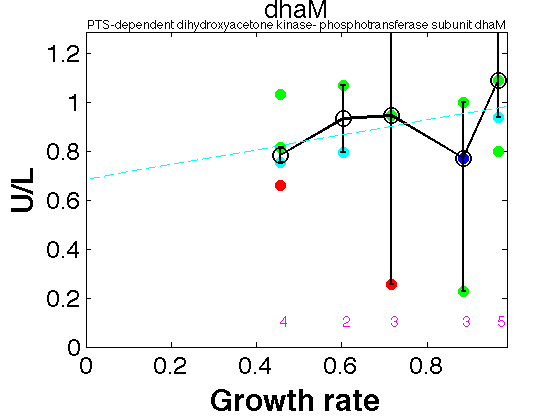

Supplement: Supplementary file 5 [file msb0011-0784-sd5.zip › Supplementary Dataset S1/Alim/dhaM.png]

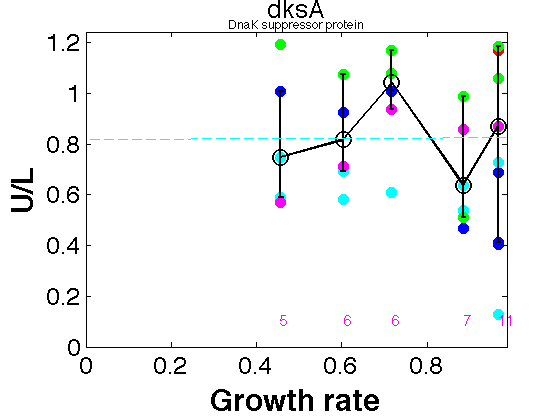

Supplement: Supplementary file 5 [file msb0011-0784-sd5.zip › Supplementary Dataset S1/Alim/dksA.png]

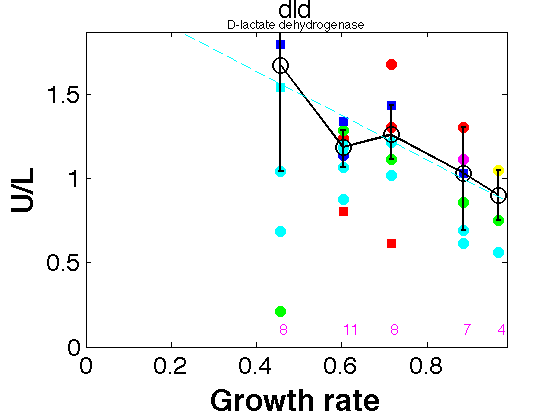

Supplement: Supplementary file 5 [file msb0011-0784-sd5.zip › Supplementary Dataset S1/Alim/dld.png]

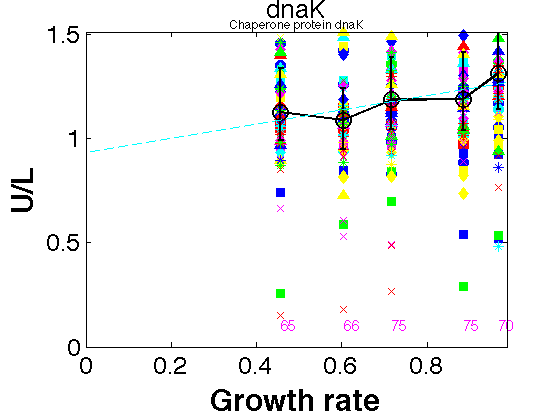

Supplement: Supplementary file 5 [file msb0011-0784-sd5.zip › Supplementary Dataset S1/Alim/dnaK.png]

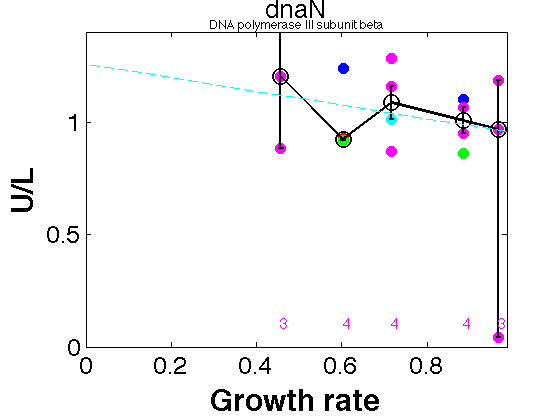

Supplement: Supplementary file 5 [file msb0011-0784-sd5.zip › Supplementary Dataset S1/Alim/dnaN.png]

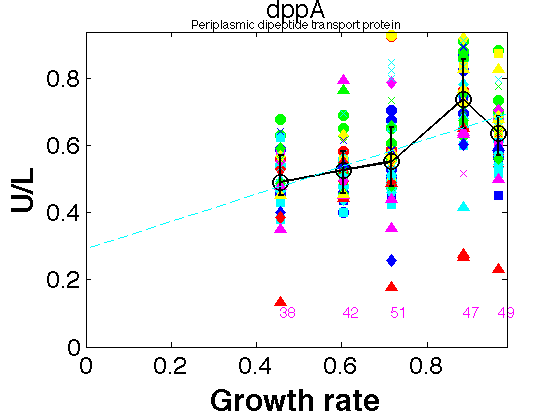

Supplement: Supplementary file 5 [file msb0011-0784-sd5.zip › Supplementary Dataset S1/Alim/dppA.png]

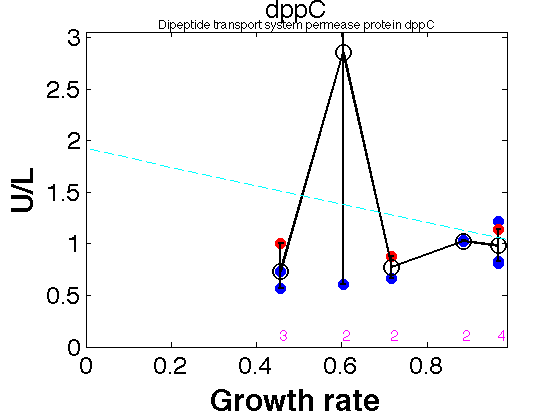

Supplement: Supplementary file 5 [file msb0011-0784-sd5.zip › Supplementary Dataset S1/Alim/dppC.png]

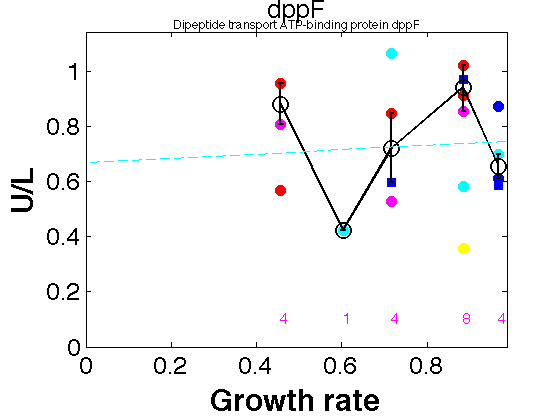

Supplement: Supplementary file 5 [file msb0011-0784-sd5.zip › Supplementary Dataset S1/Alim/dppF.png]

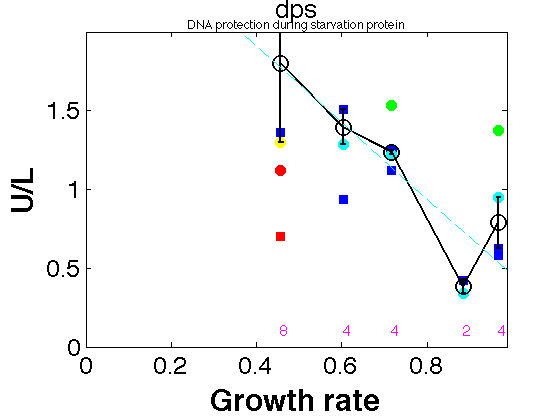

Supplement: Supplementary file 5 [file msb0011-0784-sd5.zip › Supplementary Dataset S1/Alim/dps.png]

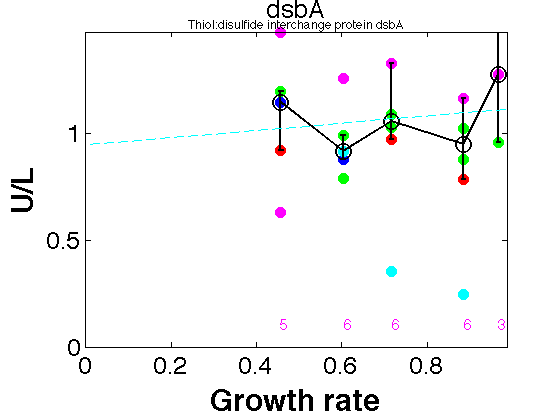

Supplement: Supplementary file 5 [file msb0011-0784-sd5.zip › Supplementary Dataset S1/Alim/dsbA.png]

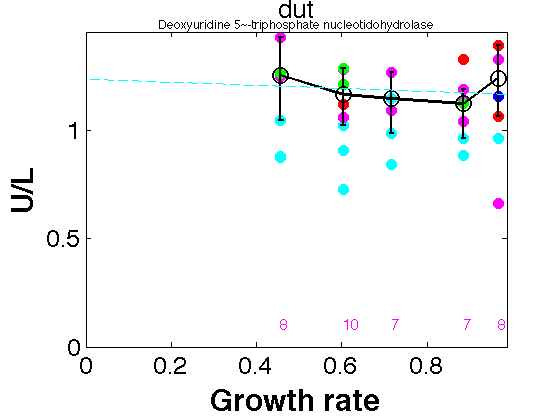

Supplement: Supplementary file 5 [file msb0011-0784-sd5.zip › Supplementary Dataset S1/Alim/dut.png]

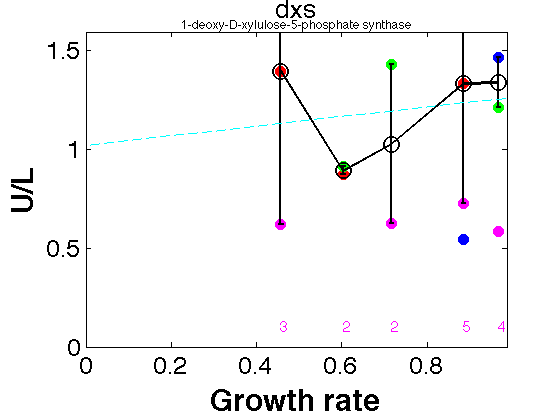

Supplement: Supplementary file 5 [file msb0011-0784-sd5.zip › Supplementary Dataset S1/Alim/dxs.png]

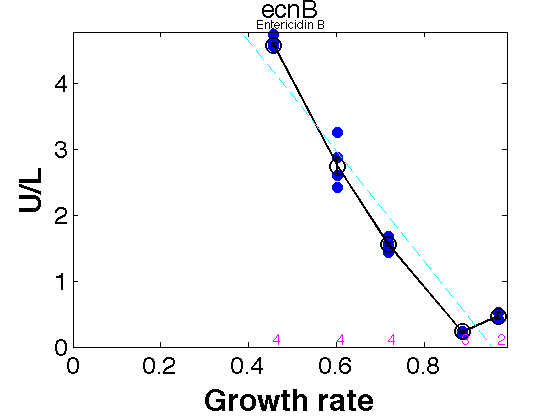

Supplement: Supplementary file 5 [file msb0011-0784-sd5.zip › Supplementary Dataset S1/Alim/ecnB.png]

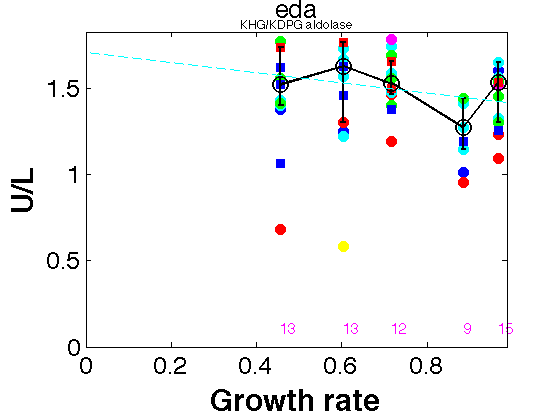

Supplement: Supplementary file 5 [file msb0011-0784-sd5.zip › Supplementary Dataset S1/Alim/eda.png]

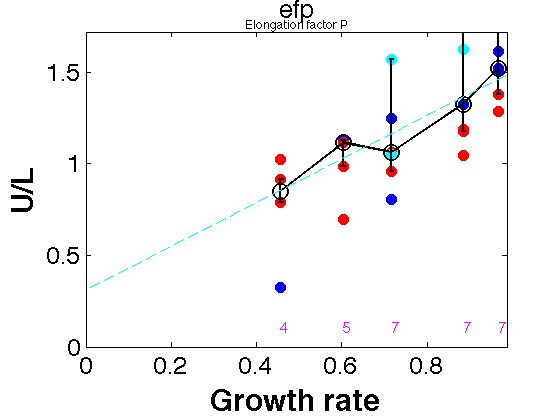

Supplement: Supplementary file 5 [file msb0011-0784-sd5.zip › Supplementary Dataset S1/Alim/efp.png]

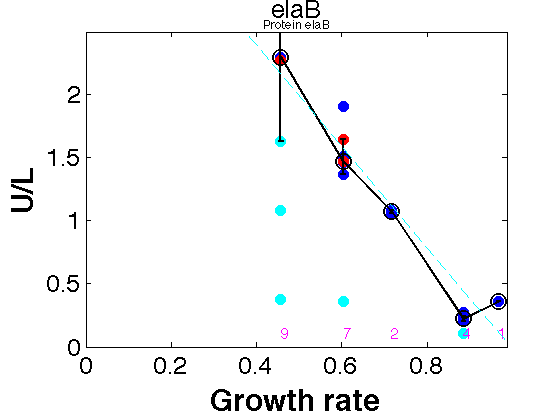

Supplement: Supplementary file 5 [file msb0011-0784-sd5.zip › Supplementary Dataset S1/Alim/elaB.png]

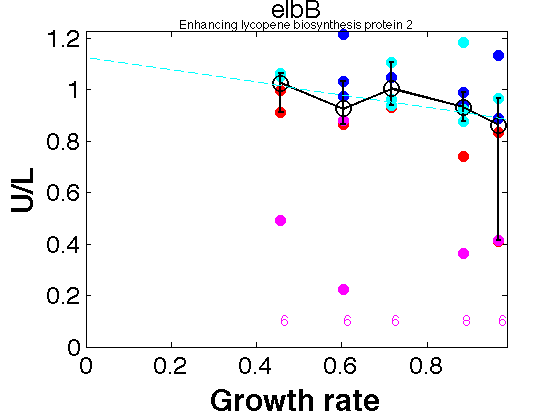

Supplement: Supplementary file 5 [file msb0011-0784-sd5.zip › Supplementary Dataset S1/Alim/elbB.png]

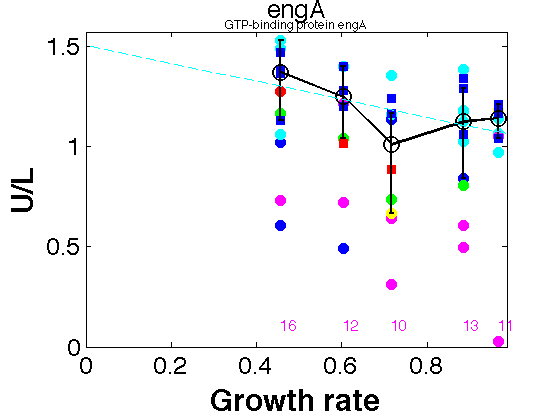

Supplement: Supplementary file 5 [file msb0011-0784-sd5.zip › Supplementary Dataset S1/Alim/engA.png]

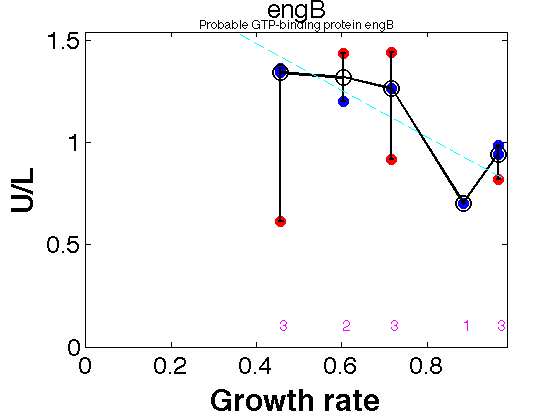

Supplement: Supplementary file 5 [file msb0011-0784-sd5.zip › Supplementary Dataset S1/Alim/engB.png]

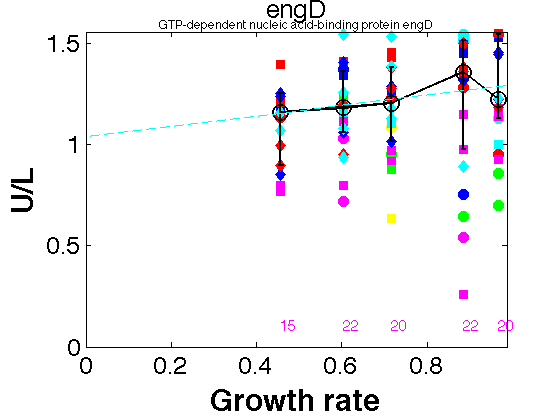

Supplement: Supplementary file 5 [file msb0011-0784-sd5.zip › Supplementary Dataset S1/Alim/engD.png]

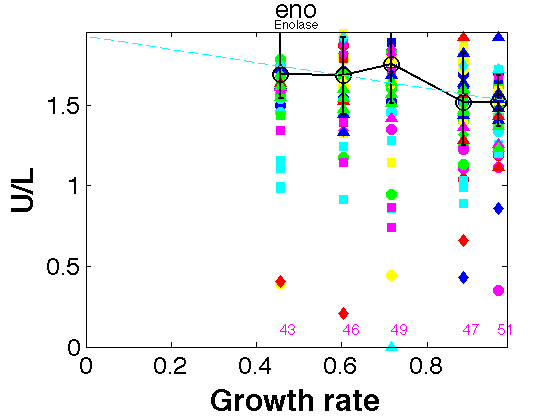

Supplement: Supplementary file 5 [file msb0011-0784-sd5.zip › Supplementary Dataset S1/Alim/eno.png]

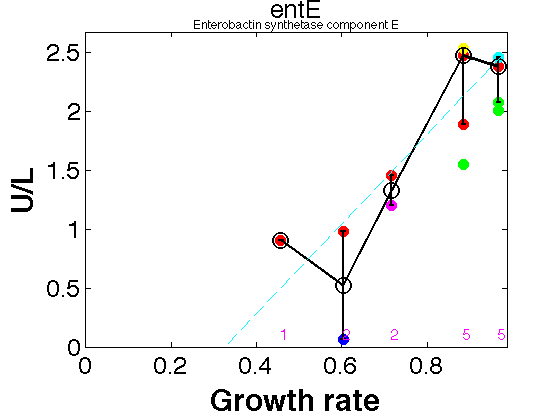

Supplement: Supplementary file 5 [file msb0011-0784-sd5.zip › Supplementary Dataset S1/Alim/entE.png]

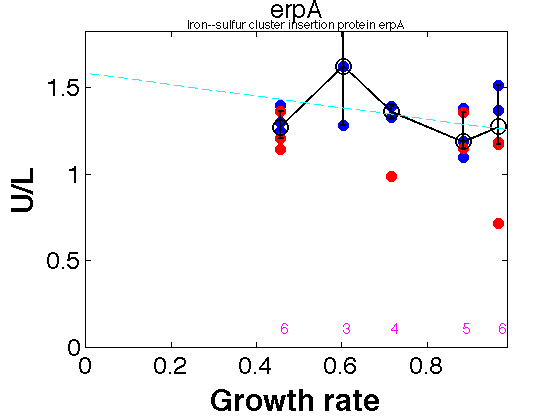

Supplement: Supplementary file 5 [file msb0011-0784-sd5.zip › Supplementary Dataset S1/Alim/erpA.png]

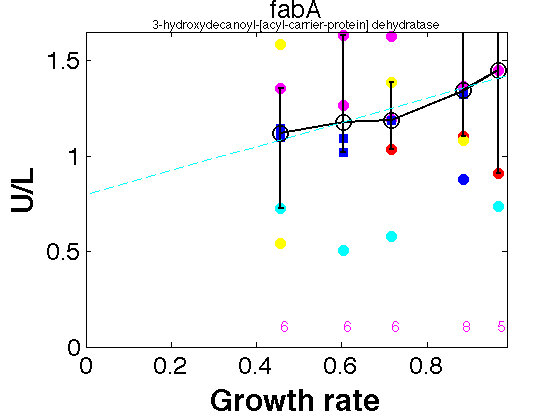

Supplement: Supplementary file 5 [file msb0011-0784-sd5.zip › Supplementary Dataset S1/Alim/fabA.png]

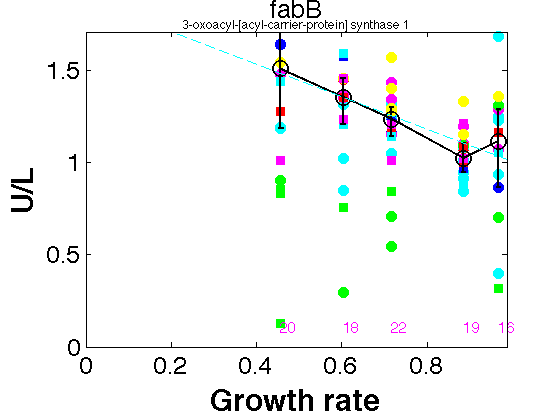

Supplement: Supplementary file 5 [file msb0011-0784-sd5.zip › Supplementary Dataset S1/Alim/fabB.png]

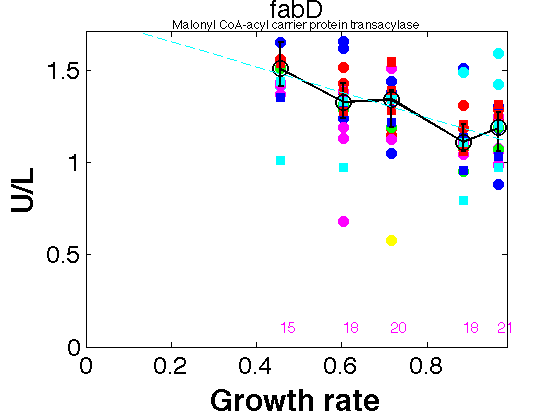

Supplement: Supplementary file 5 [file msb0011-0784-sd5.zip › Supplementary Dataset S1/Alim/fabD.png]

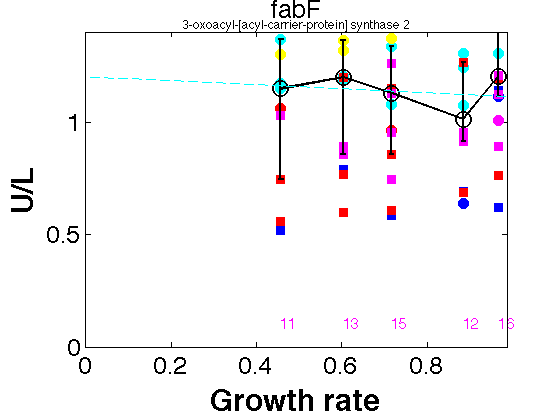

Supplement: Supplementary file 5 [file msb0011-0784-sd5.zip › Supplementary Dataset S1/Alim/fabF.png]

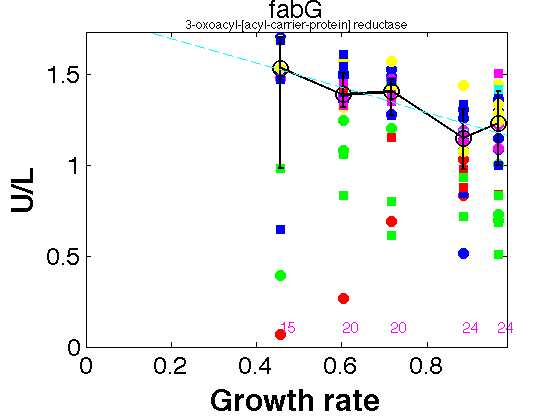

Supplement: Supplementary file 5 [file msb0011-0784-sd5.zip › Supplementary Dataset S1/Alim/fabG.png]

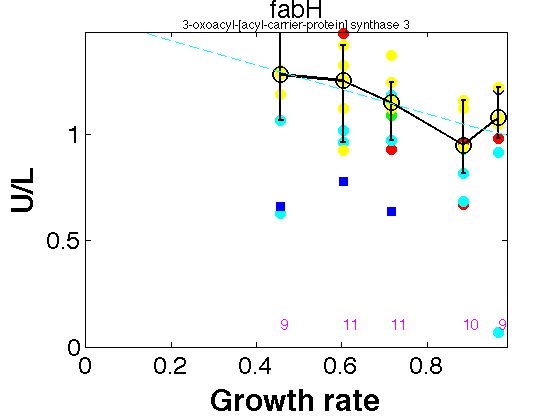

Supplement: Supplementary file 5 [file msb0011-0784-sd5.zip › Supplementary Dataset S1/Alim/fabH.png]

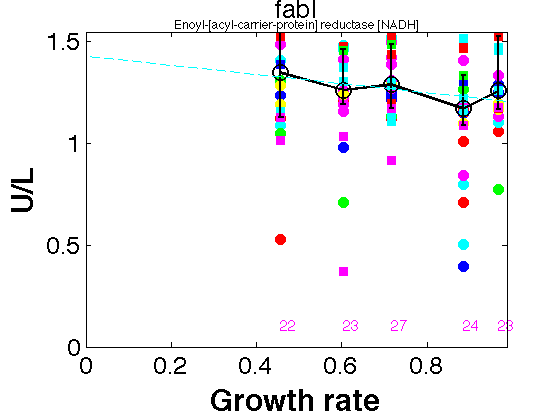

Supplement: Supplementary file 5 [file msb0011-0784-sd5.zip › Supplementary Dataset S1/Alim/fabI.png]

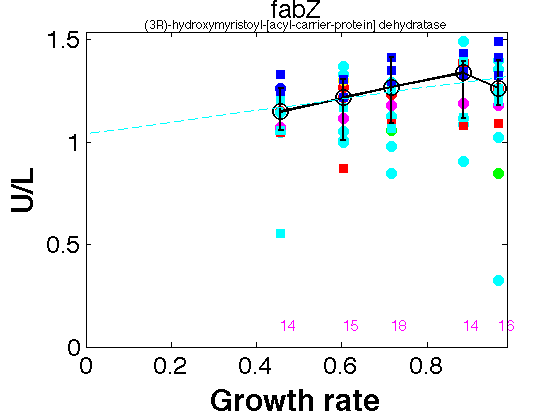

Supplement: Supplementary file 5 [file msb0011-0784-sd5.zip › Supplementary Dataset S1/Alim/fabZ.png]

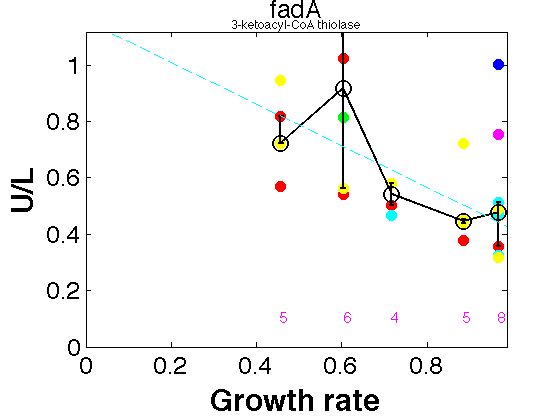

Supplement: Supplementary file 5 [file msb0011-0784-sd5.zip › Supplementary Dataset S1/Alim/fadA.png]

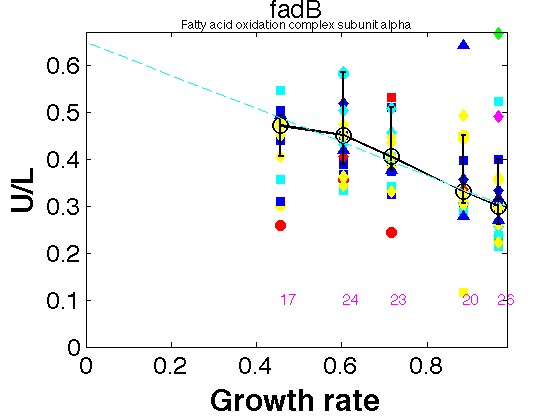

Supplement: Supplementary file 5 [file msb0011-0784-sd5.zip › Supplementary Dataset S1/Alim/fadB.png]

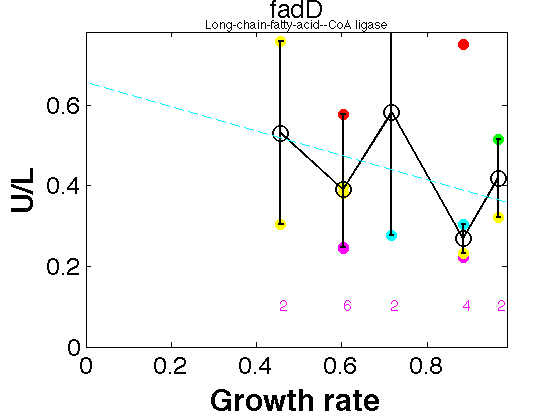

Supplement: Supplementary file 5 [file msb0011-0784-sd5.zip › Supplementary Dataset S1/Alim/fadD.png]

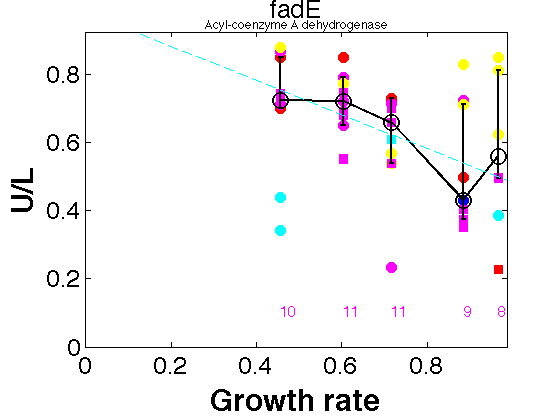

Supplement: Supplementary file 5 [file msb0011-0784-sd5.zip › Supplementary Dataset S1/Alim/fadE.png]

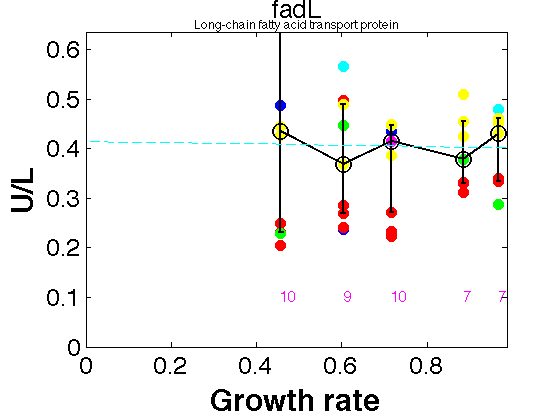

Supplement: Supplementary file 5 [file msb0011-0784-sd5.zip › Supplementary Dataset S1/Alim/fadL.png]

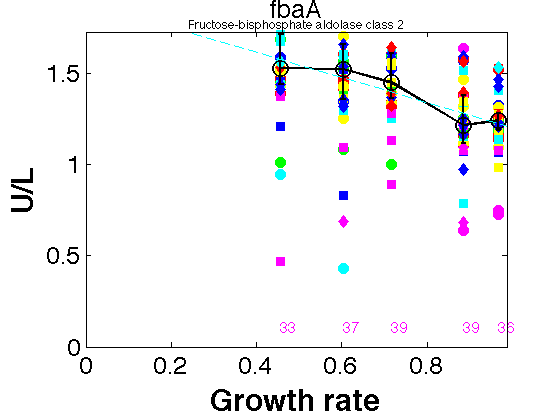

Supplement: Supplementary file 5 [file msb0011-0784-sd5.zip › Supplementary Dataset S1/Alim/fbaA.png]

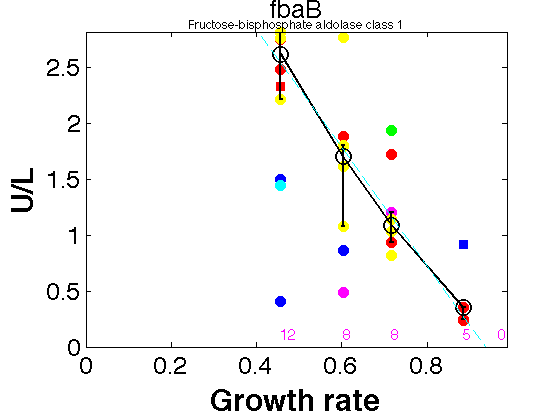

Supplement: Supplementary file 5 [file msb0011-0784-sd5.zip › Supplementary Dataset S1/Alim/fbaB.png]

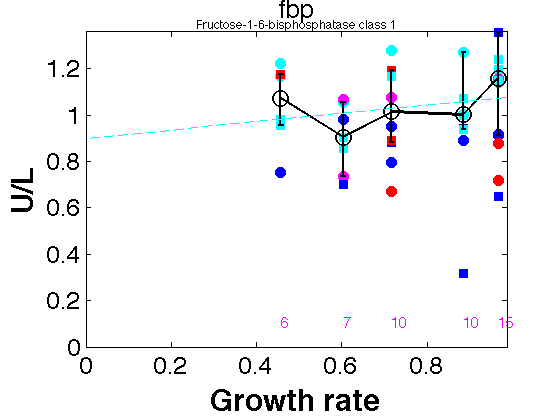

Supplement: Supplementary file 5 [file msb0011-0784-sd5.zip › Supplementary Dataset S1/Alim/fbp.png]

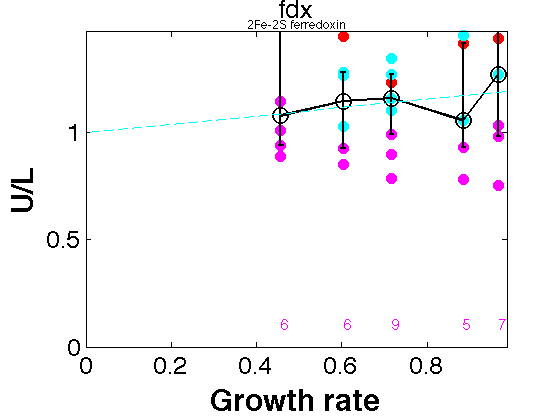

Supplement: Supplementary file 5 [file msb0011-0784-sd5.zip › Supplementary Dataset S1/Alim/fdx.png]

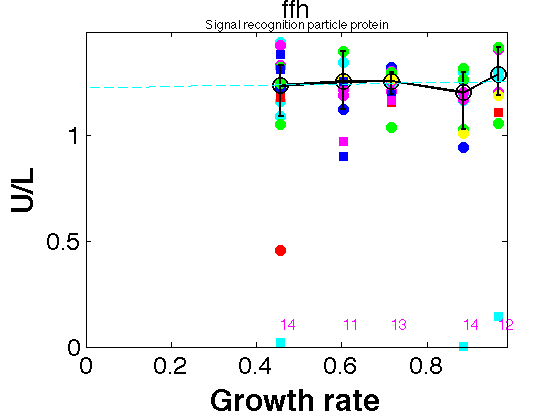

Supplement: Supplementary file 5 [file msb0011-0784-sd5.zip › Supplementary Dataset S1/Alim/ffh.png]

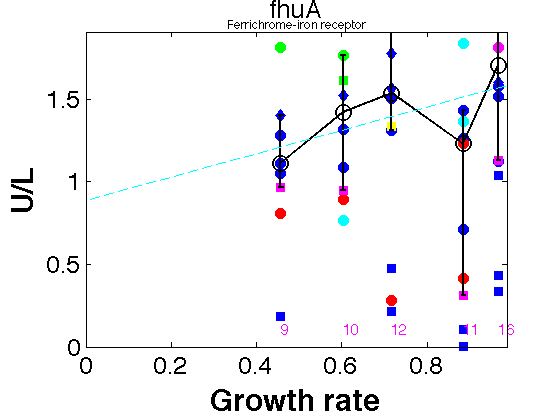

Supplement: Supplementary file 5 [file msb0011-0784-sd5.zip › Supplementary Dataset S1/Alim/fhuA.png]

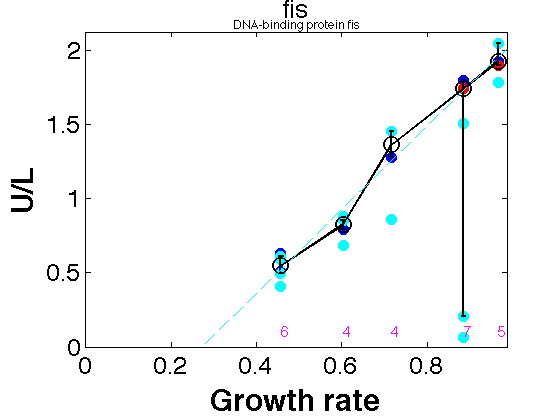

Supplement: Supplementary file 5 [file msb0011-0784-sd5.zip › Supplementary Dataset S1/Alim/fis.png]

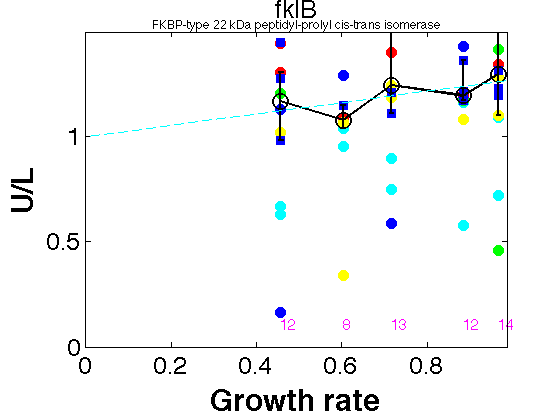

Supplement: Supplementary file 5 [file msb0011-0784-sd5.zip › Supplementary Dataset S1/Alim/fklB.png]

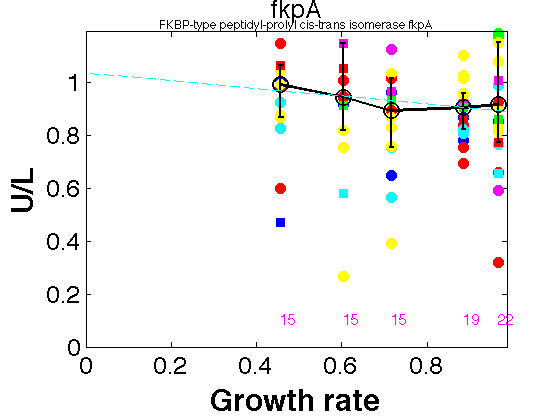

Supplement: Supplementary file 5 [file msb0011-0784-sd5.zip › Supplementary Dataset S1/Alim/fkpA.png]

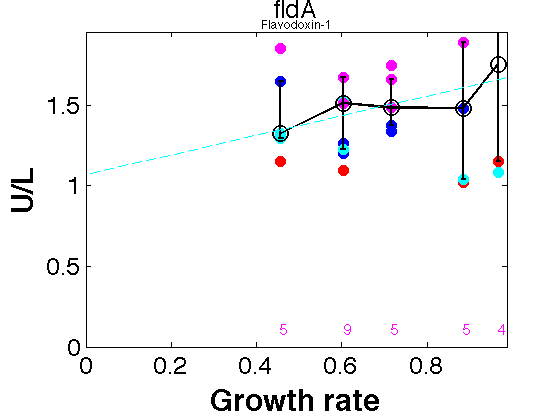

Supplement: Supplementary file 5 [file msb0011-0784-sd5.zip › Supplementary Dataset S1/Alim/fldA.png]

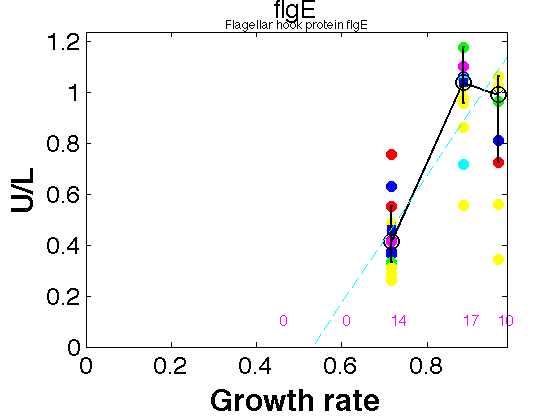

Supplement: Supplementary file 5 [file msb0011-0784-sd5.zip › Supplementary Dataset S1/Alim/flgE.png]

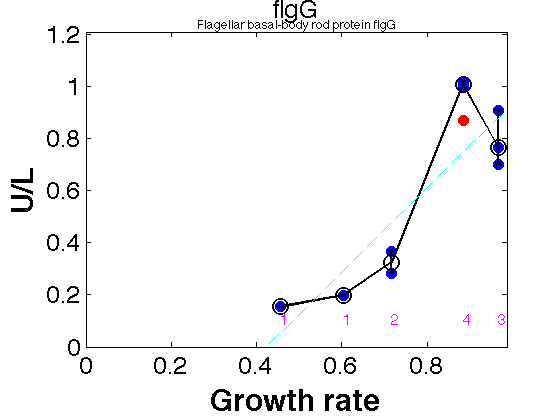

Supplement: Supplementary file 5 [file msb0011-0784-sd5.zip › Supplementary Dataset S1/Alim/flgG.png]

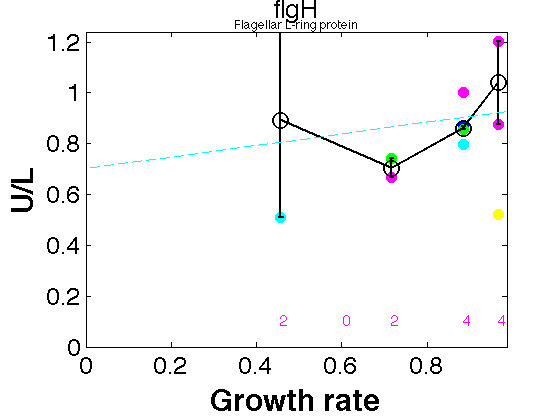

Supplement: Supplementary file 5 [file msb0011-0784-sd5.zip › Supplementary Dataset S1/Alim/flgH.png]

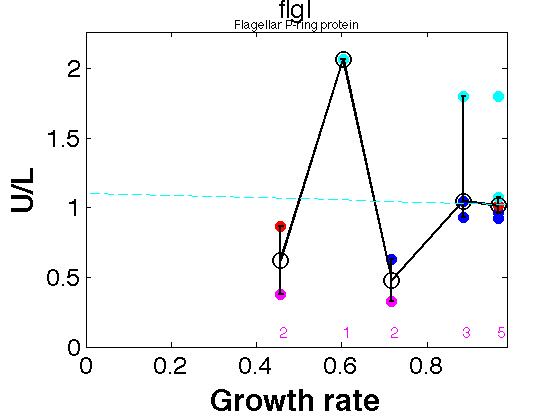

Supplement: Supplementary file 5 [file msb0011-0784-sd5.zip › Supplementary Dataset S1/Alim/flgI.png]

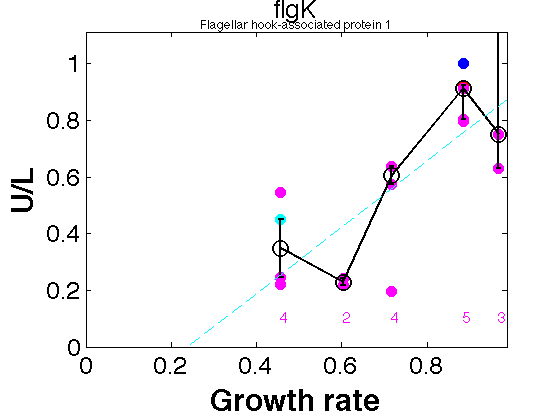

Supplement: Supplementary file 5 [file msb0011-0784-sd5.zip › Supplementary Dataset S1/Alim/flgK.png]

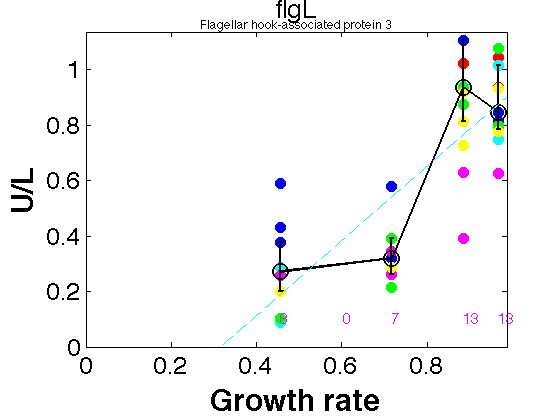

Supplement: Supplementary file 5 [file msb0011-0784-sd5.zip › Supplementary Dataset S1/Alim/flgL.png]

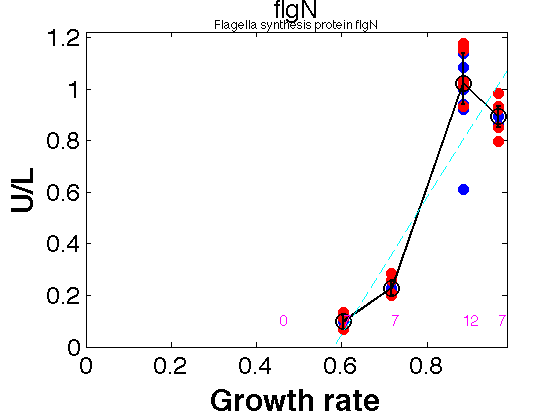

Supplement: Supplementary file 5 [file msb0011-0784-sd5.zip › Supplementary Dataset S1/Alim/flgN.png]

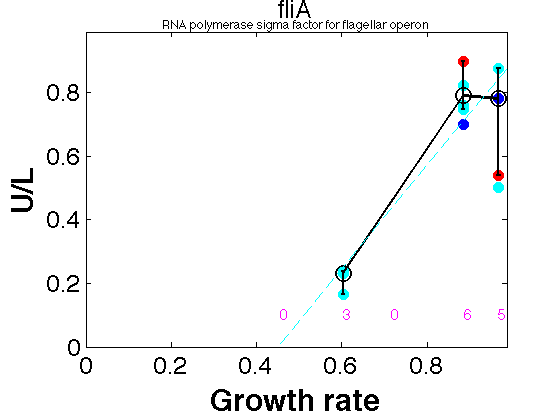

Supplement: Supplementary file 5 [file msb0011-0784-sd5.zip › Supplementary Dataset S1/Alim/fliA.png]

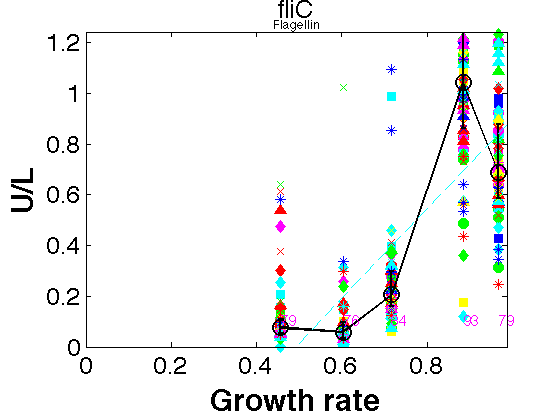

Supplement: Supplementary file 5 [file msb0011-0784-sd5.zip › Supplementary Dataset S1/Alim/fliC.png]

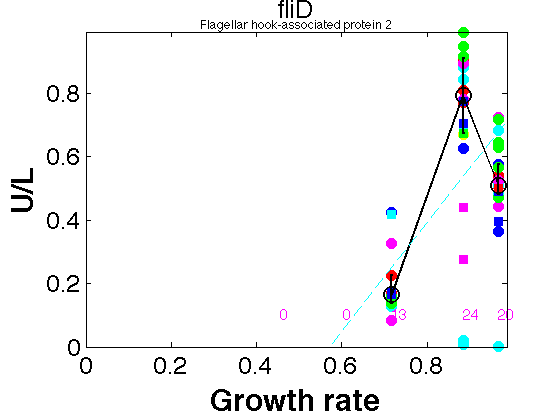

Supplement: Supplementary file 5 [file msb0011-0784-sd5.zip › Supplementary Dataset S1/Alim/fliD.png]

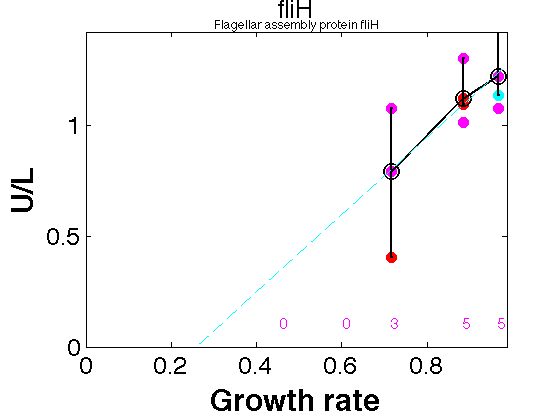

Supplement: Supplementary file 5 [file msb0011-0784-sd5.zip › Supplementary Dataset S1/Alim/fliH.png]

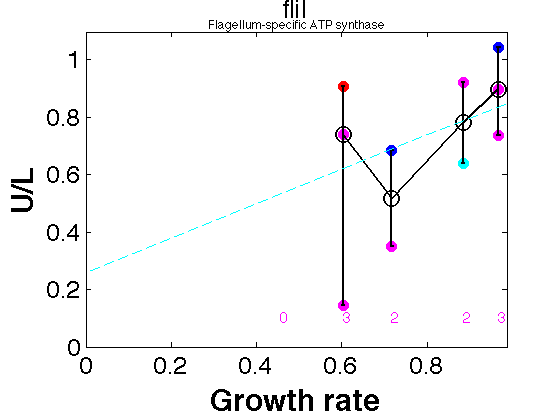

Supplement: Supplementary file 5 [file msb0011-0784-sd5.zip › Supplementary Dataset S1/Alim/fliI.png]

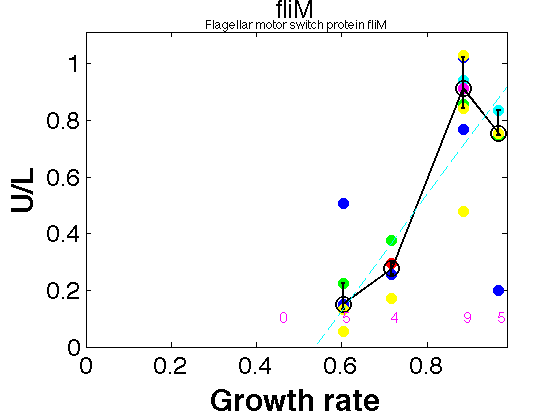

Supplement: Supplementary file 5 [file msb0011-0784-sd5.zip › Supplementary Dataset S1/Alim/fliM.png]

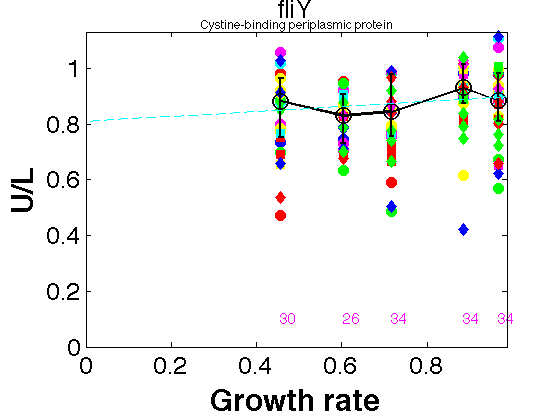

Supplement: Supplementary file 5 [file msb0011-0784-sd5.zip › Supplementary Dataset S1/Alim/fliY.png]
