# Supplementary figures and images for: Quantitative proteomic analysis reveals a simple strategy of global resource allocation in bacteria (part 1 of 2)
Source: Mol Syst Biol. 2015 Feb 12;11(2):784. doi: 10.15252/msb.20145697 (PMC4358657; doi:10.15252/msb.20145697)

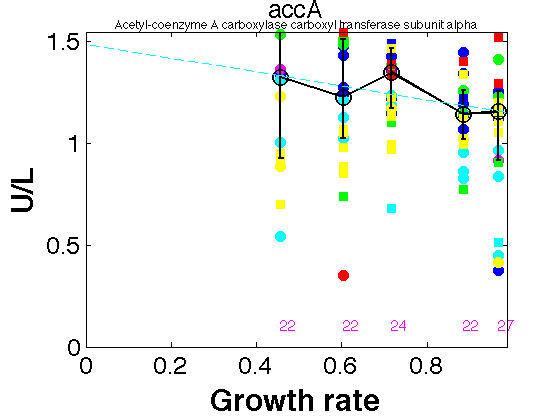

Supplement: Supplementary file 5 [file msb0011-0784-sd5.zip › Supplementary Dataset S1/Alim/accA.png]

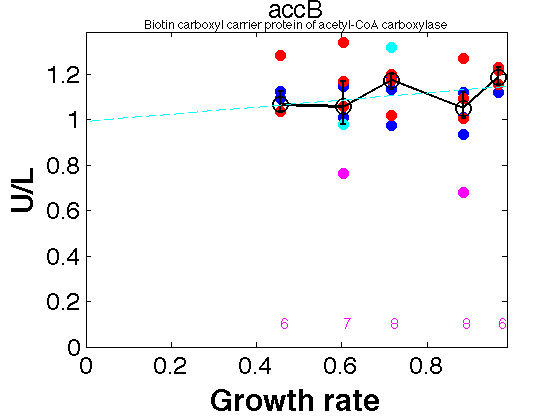

Supplement: Supplementary file 5 [file msb0011-0784-sd5.zip › Supplementary Dataset S1/Alim/accB.png]

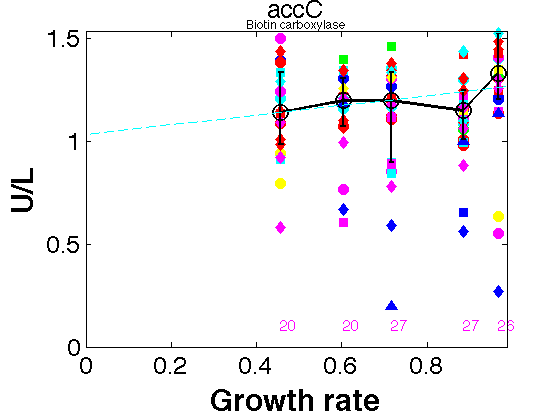

Supplement: Supplementary file 5 [file msb0011-0784-sd5.zip › Supplementary Dataset S1/Alim/accC.png]

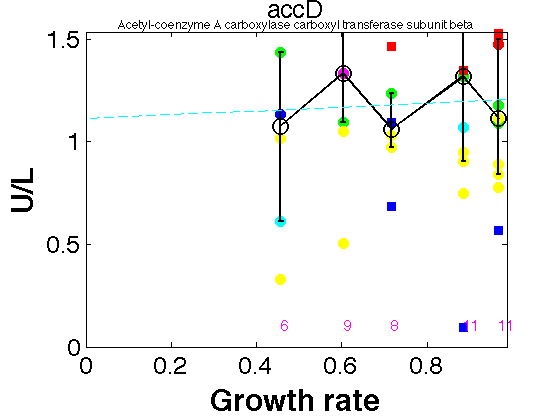

Supplement: Supplementary file 5 [file msb0011-0784-sd5.zip › Supplementary Dataset S1/Alim/accD.png]

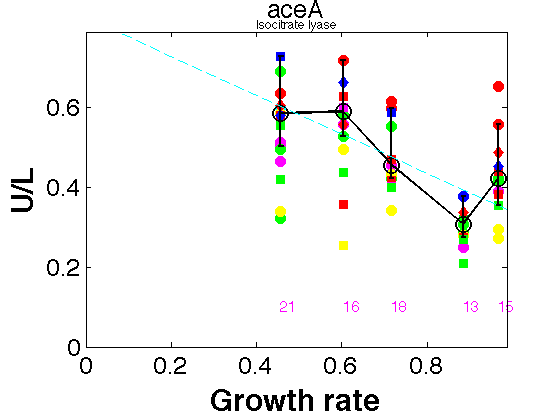

Supplement: Supplementary file 5 [file msb0011-0784-sd5.zip › Supplementary Dataset S1/Alim/aceA.png]

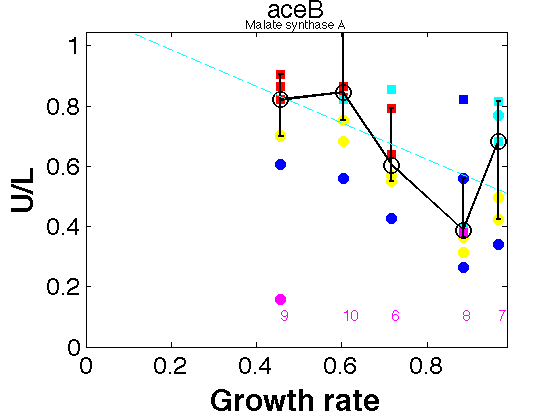

Supplement: Supplementary file 5 [file msb0011-0784-sd5.zip › Supplementary Dataset S1/Alim/aceB.png]

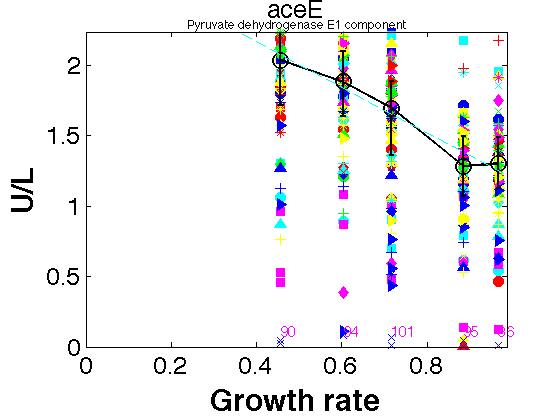

Supplement: Supplementary file 5 [file msb0011-0784-sd5.zip › Supplementary Dataset S1/Alim/aceE.png]

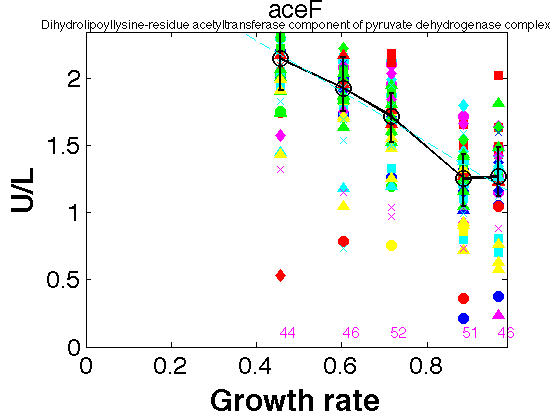

Supplement: Supplementary file 5 [file msb0011-0784-sd5.zip › Supplementary Dataset S1/Alim/aceF.png]

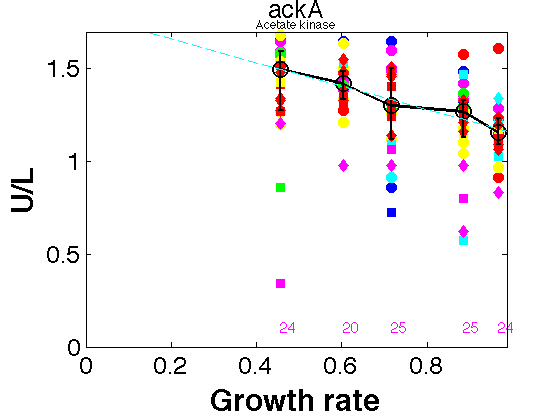

Supplement: Supplementary file 5 [file msb0011-0784-sd5.zip › Supplementary Dataset S1/Alim/ackA.png]

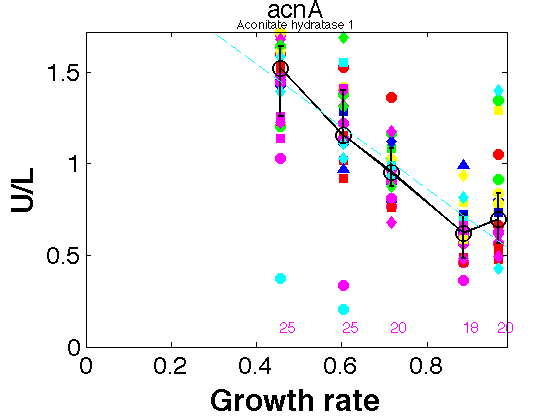

Supplement: Supplementary file 5 [file msb0011-0784-sd5.zip › Supplementary Dataset S1/Alim/acnA.png]

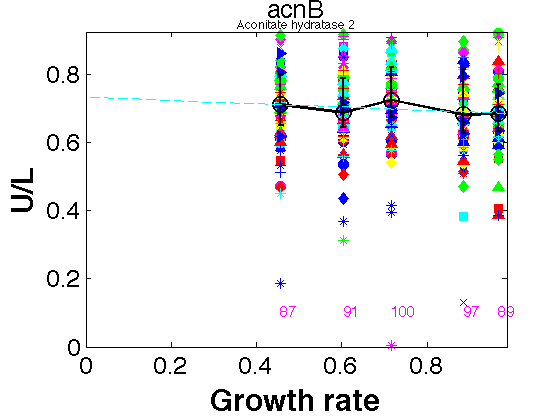

Supplement: Supplementary file 5 [file msb0011-0784-sd5.zip › Supplementary Dataset S1/Alim/acnB.png]

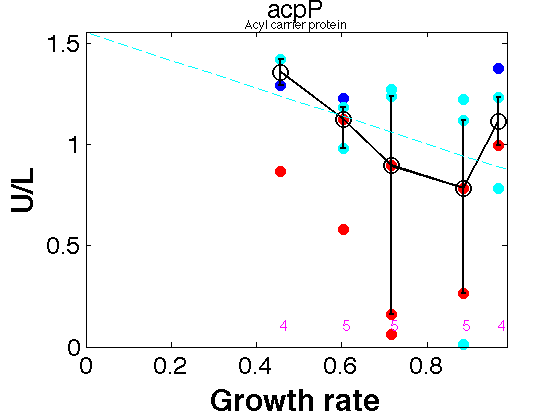

Supplement: Supplementary file 5 [file msb0011-0784-sd5.zip › Supplementary Dataset S1/Alim/acpP.png]

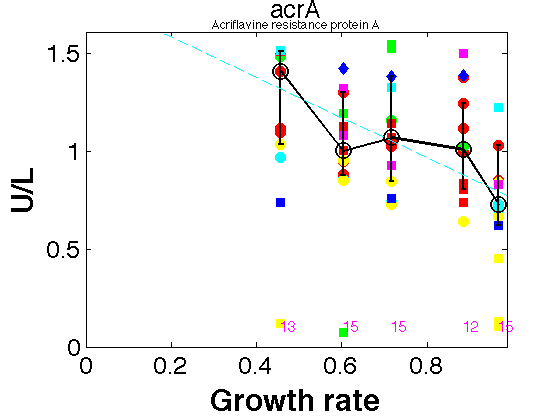

Supplement: Supplementary file 5 [file msb0011-0784-sd5.zip › Supplementary Dataset S1/Alim/acrA.png]

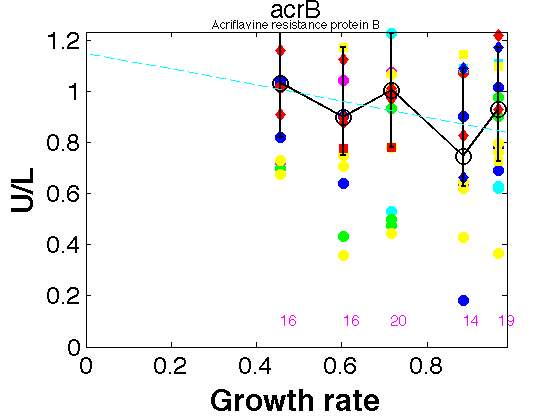

Supplement: Supplementary file 5 [file msb0011-0784-sd5.zip › Supplementary Dataset S1/Alim/acrB.png]

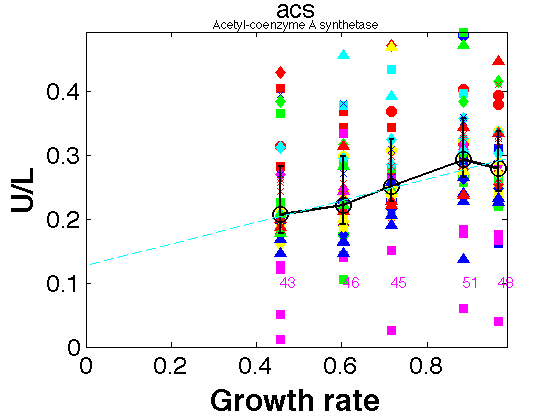

Supplement: Supplementary file 5 [file msb0011-0784-sd5.zip › Supplementary Dataset S1/Alim/acs.png]

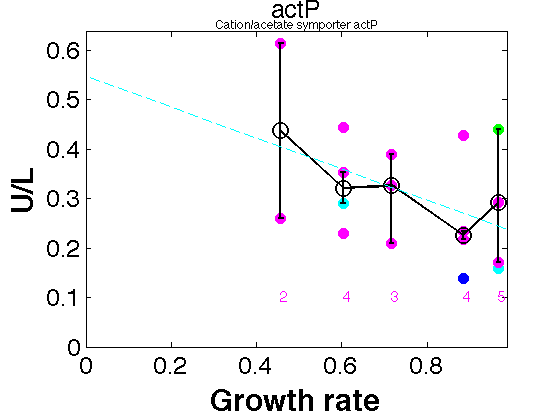

Supplement: Supplementary file 5 [file msb0011-0784-sd5.zip › Supplementary Dataset S1/Alim/actP.png]

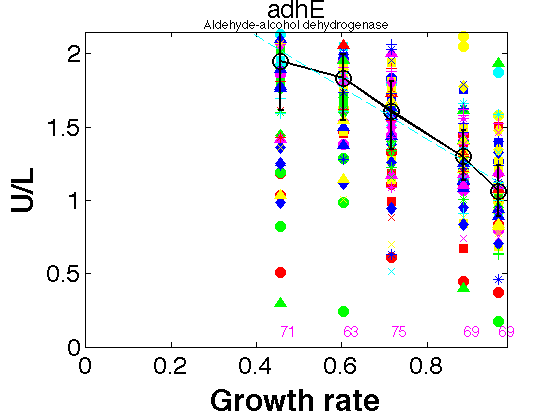

Supplement: Supplementary file 5 [file msb0011-0784-sd5.zip › Supplementary Dataset S1/Alim/adhE.png]

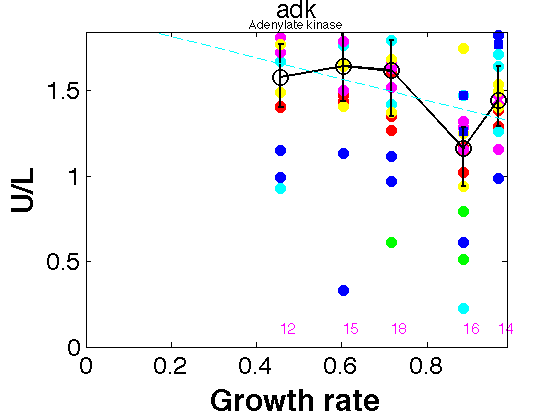

Supplement: Supplementary file 5 [file msb0011-0784-sd5.zip › Supplementary Dataset S1/Alim/adk.png]

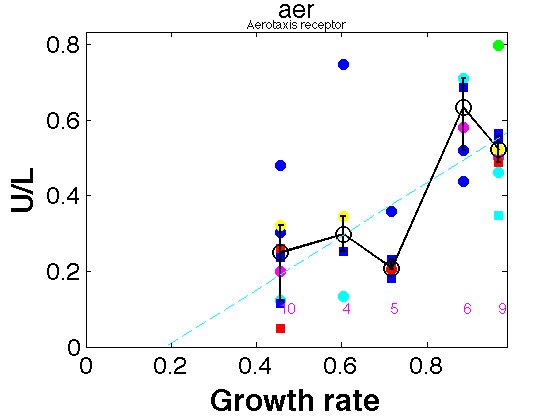

Supplement: Supplementary file 5 [file msb0011-0784-sd5.zip › Supplementary Dataset S1/Alim/aer.png]

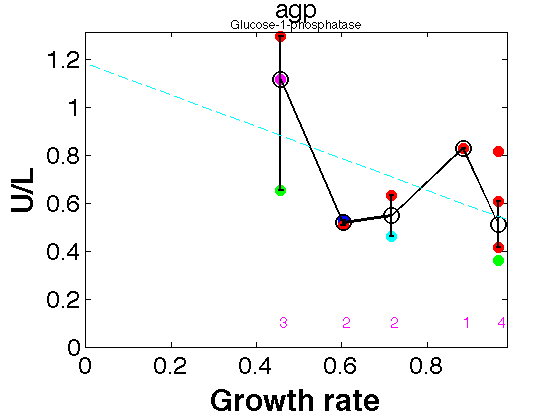

Supplement: Supplementary file 5 [file msb0011-0784-sd5.zip › Supplementary Dataset S1/Alim/agp.png]

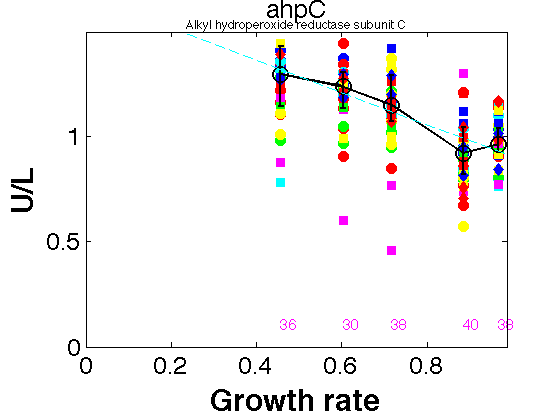

Supplement: Supplementary file 5 [file msb0011-0784-sd5.zip › Supplementary Dataset S1/Alim/ahpC.png]

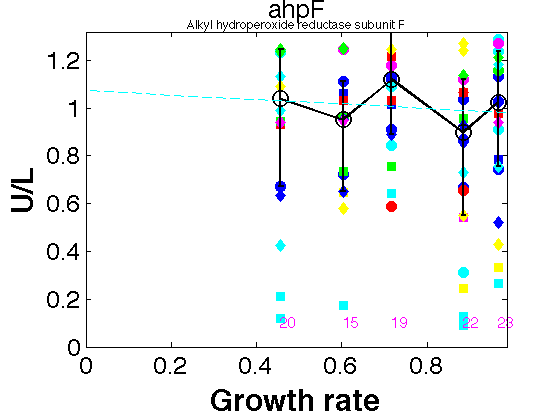

Supplement: Supplementary file 5 [file msb0011-0784-sd5.zip › Supplementary Dataset S1/Alim/ahpF.png]

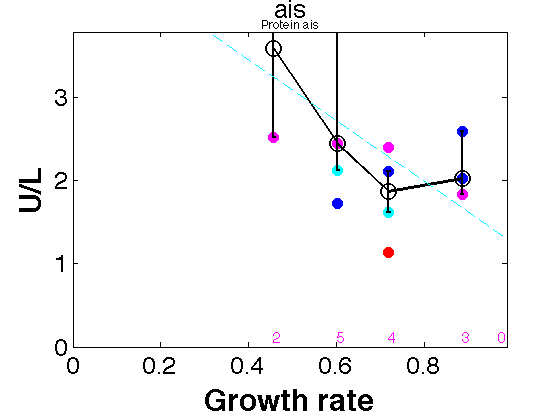

Supplement: Supplementary file 5 [file msb0011-0784-sd5.zip › Supplementary Dataset S1/Alim/ais.png]

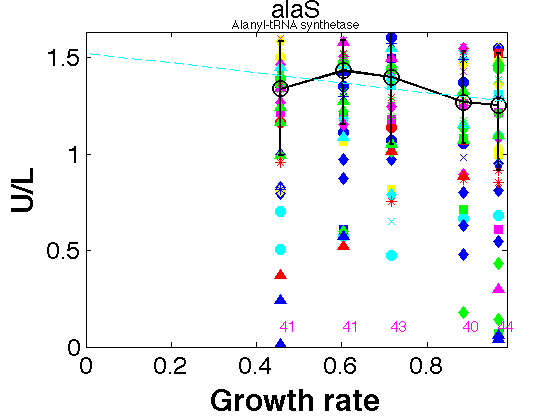

Supplement: Supplementary file 5 [file msb0011-0784-sd5.zip › Supplementary Dataset S1/Alim/alaS.png]

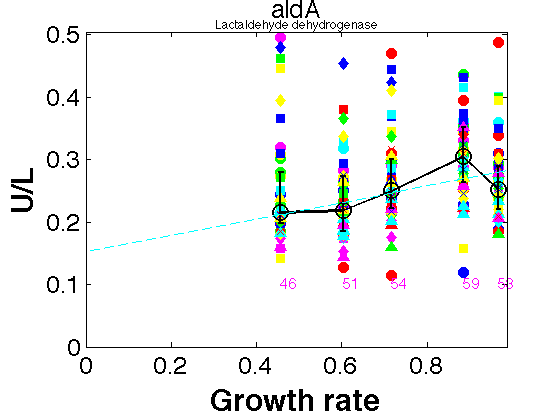

Supplement: Supplementary file 5 [file msb0011-0784-sd5.zip › Supplementary Dataset S1/Alim/aldA.png]

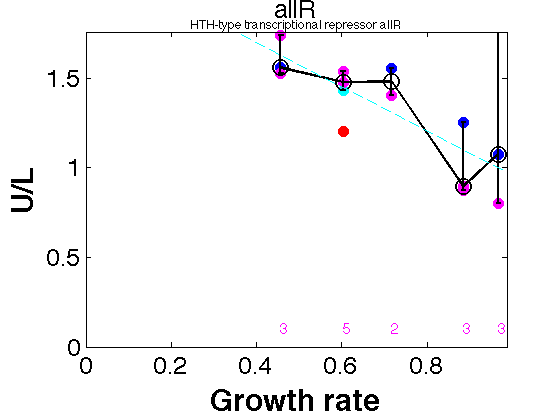

Supplement: Supplementary file 5 [file msb0011-0784-sd5.zip › Supplementary Dataset S1/Alim/allR.png]

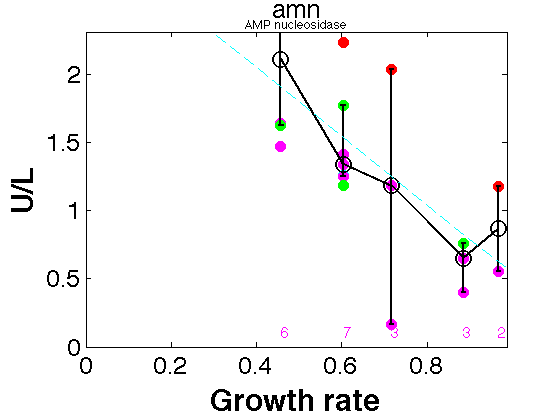

Supplement: Supplementary file 5 [file msb0011-0784-sd5.zip › Supplementary Dataset S1/Alim/amn.png]

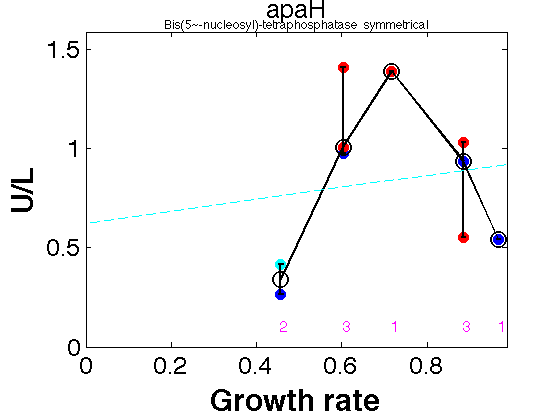

Supplement: Supplementary file 5 [file msb0011-0784-sd5.zip › Supplementary Dataset S1/Alim/apaH.png]

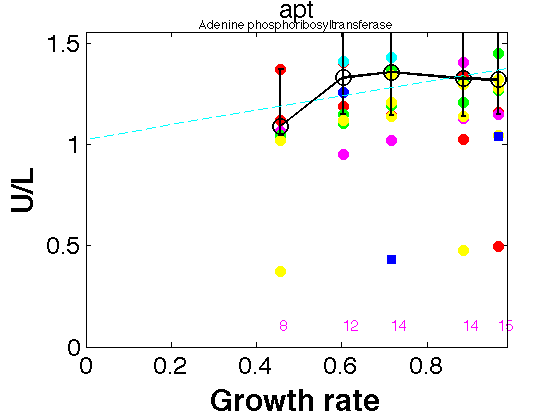

Supplement: Supplementary file 5 [file msb0011-0784-sd5.zip › Supplementary Dataset S1/Alim/apt.png]

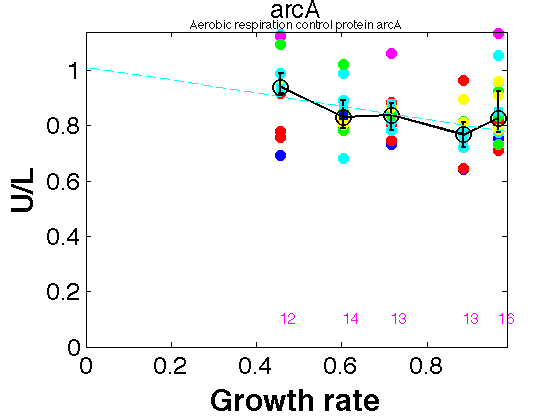

Supplement: Supplementary file 5 [file msb0011-0784-sd5.zip › Supplementary Dataset S1/Alim/arcA.png]

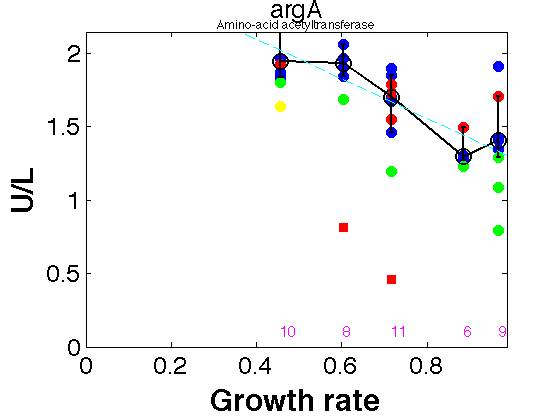

Supplement: Supplementary file 5 [file msb0011-0784-sd5.zip › Supplementary Dataset S1/Alim/argA.png]

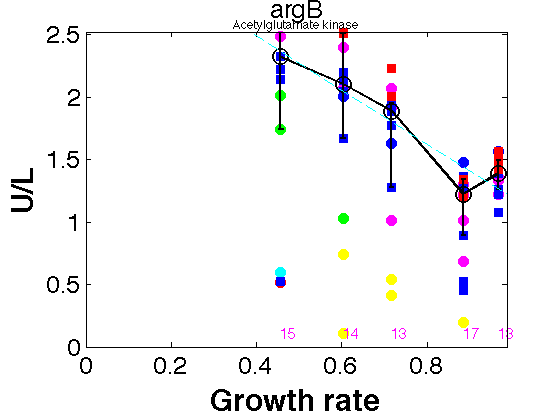

Supplement: Supplementary file 5 [file msb0011-0784-sd5.zip › Supplementary Dataset S1/Alim/argB.png]

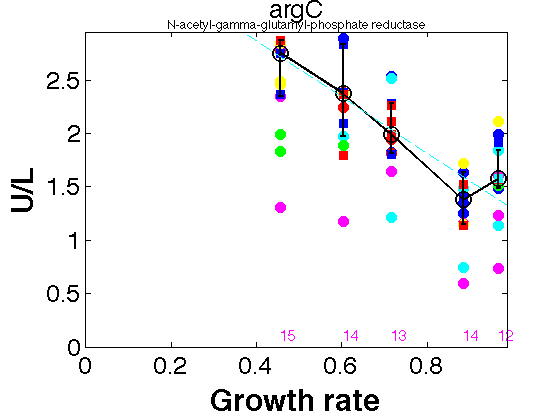

Supplement: Supplementary file 5 [file msb0011-0784-sd5.zip › Supplementary Dataset S1/Alim/argC.png]

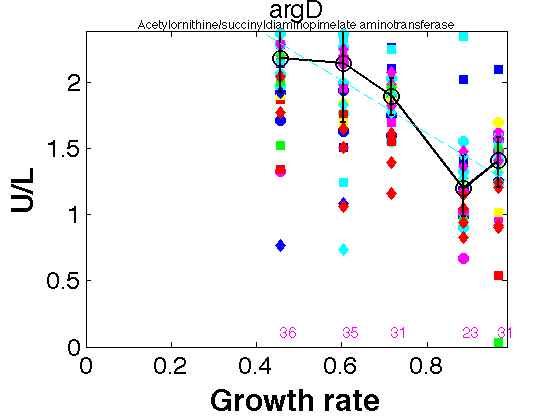

Supplement: Supplementary file 5 [file msb0011-0784-sd5.zip › Supplementary Dataset S1/Alim/argD.png]

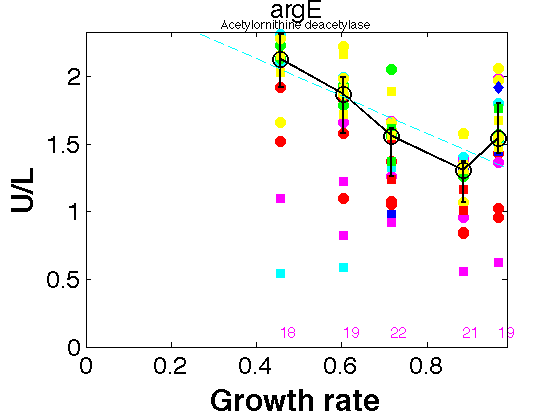

Supplement: Supplementary file 5 [file msb0011-0784-sd5.zip › Supplementary Dataset S1/Alim/argE.png]

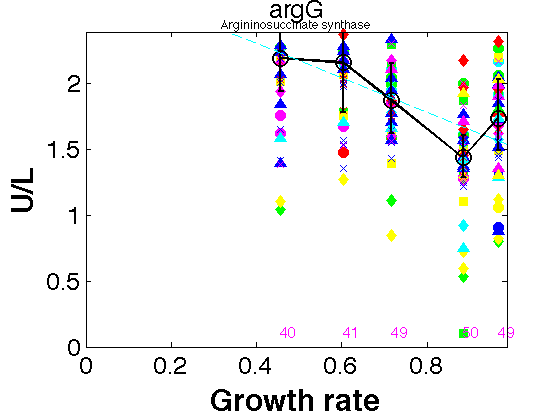

Supplement: Supplementary file 5 [file msb0011-0784-sd5.zip › Supplementary Dataset S1/Alim/argG.png]

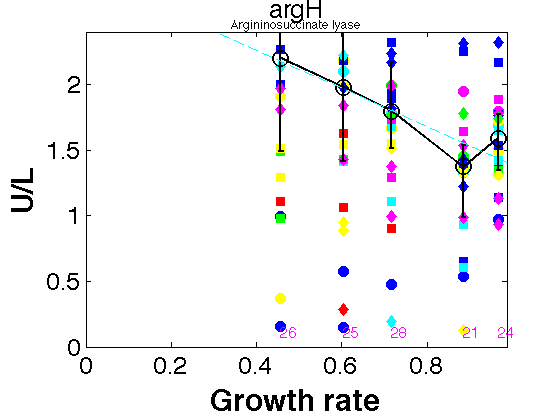

Supplement: Supplementary file 5 [file msb0011-0784-sd5.zip › Supplementary Dataset S1/Alim/argH.png]

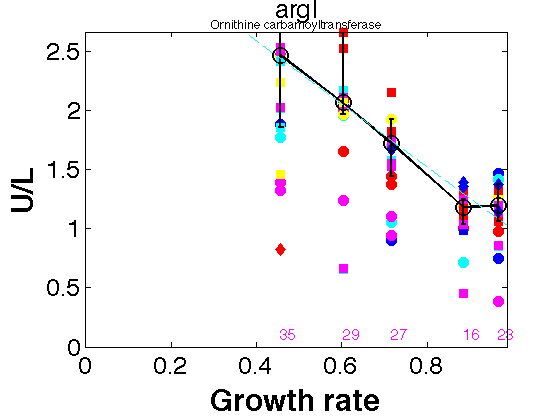

Supplement: Supplementary file 5 [file msb0011-0784-sd5.zip › Supplementary Dataset S1/Alim/argI.png]

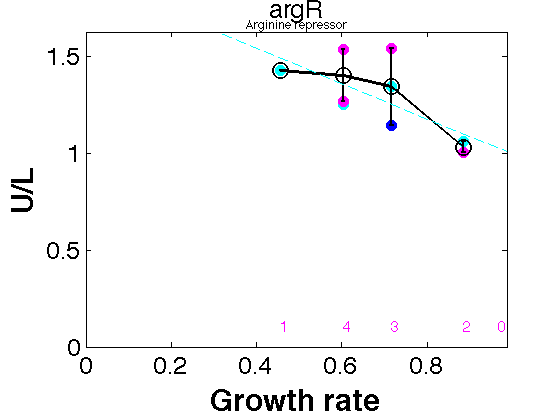

Supplement: Supplementary file 5 [file msb0011-0784-sd5.zip › Supplementary Dataset S1/Alim/argR.png]

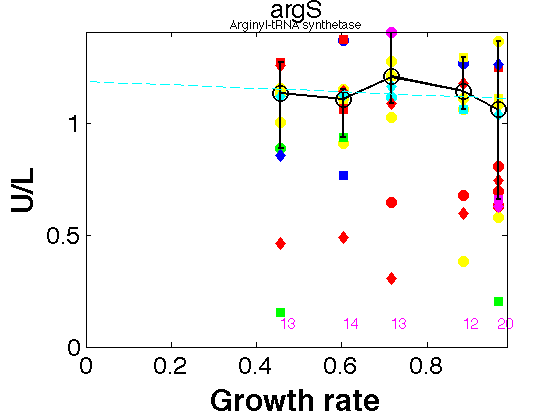

Supplement: Supplementary file 5 [file msb0011-0784-sd5.zip › Supplementary Dataset S1/Alim/argS.png]

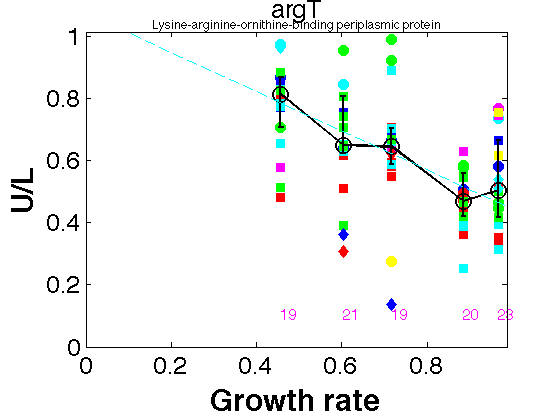

Supplement: Supplementary file 5 [file msb0011-0784-sd5.zip › Supplementary Dataset S1/Alim/argT.png]

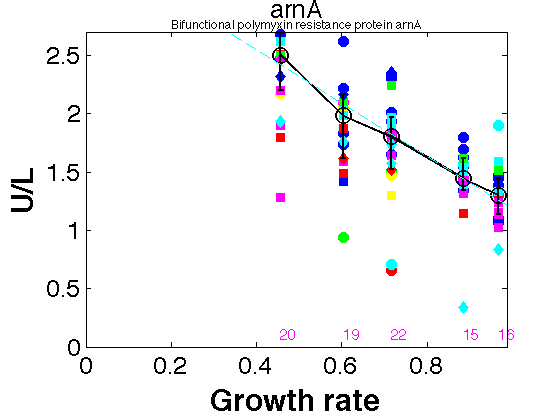

Supplement: Supplementary file 5 [file msb0011-0784-sd5.zip › Supplementary Dataset S1/Alim/arnA.png]

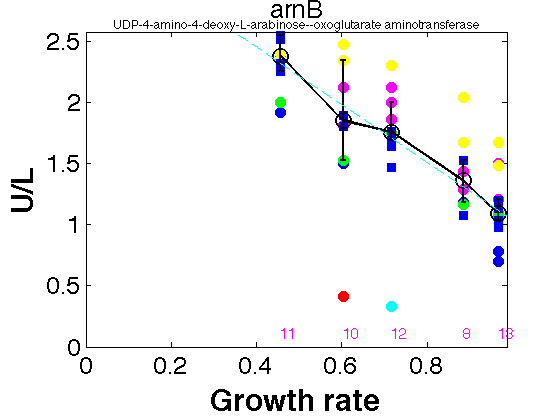

Supplement: Supplementary file 5 [file msb0011-0784-sd5.zip › Supplementary Dataset S1/Alim/arnB.png]

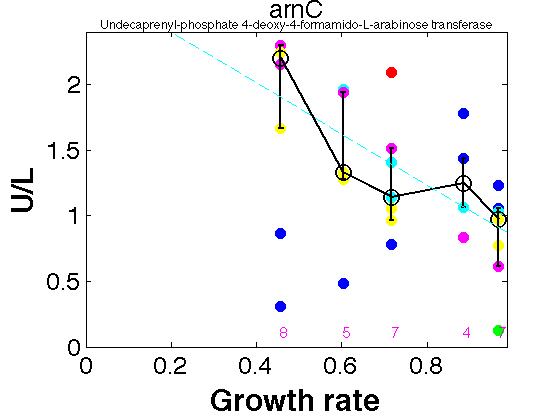

Supplement: Supplementary file 5 [file msb0011-0784-sd5.zip › Supplementary Dataset S1/Alim/arnC.png]

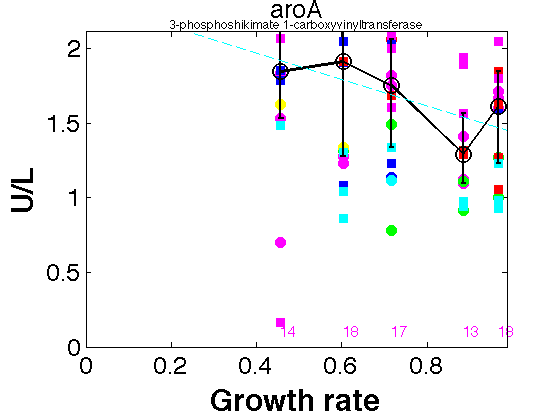

Supplement: Supplementary file 5 [file msb0011-0784-sd5.zip › Supplementary Dataset S1/Alim/aroA.png]

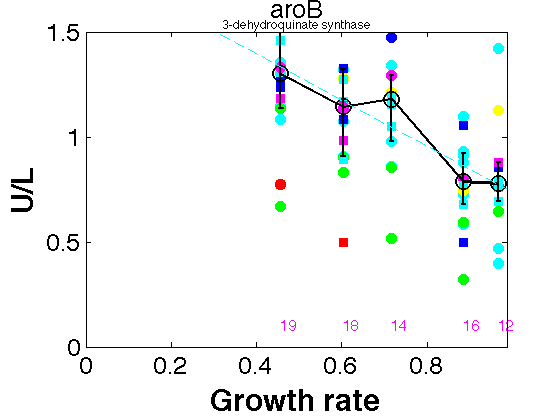

Supplement: Supplementary file 5 [file msb0011-0784-sd5.zip › Supplementary Dataset S1/Alim/aroB.png]

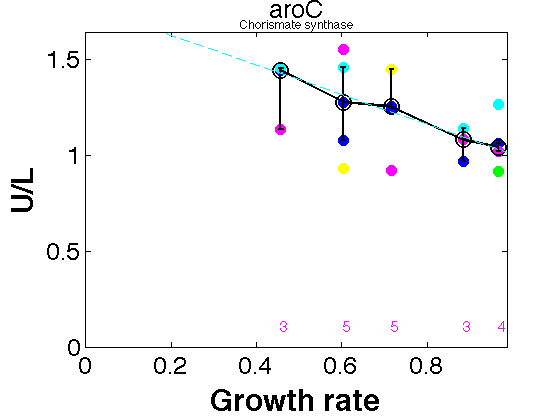

Supplement: Supplementary file 5 [file msb0011-0784-sd5.zip › Supplementary Dataset S1/Alim/aroC.png]

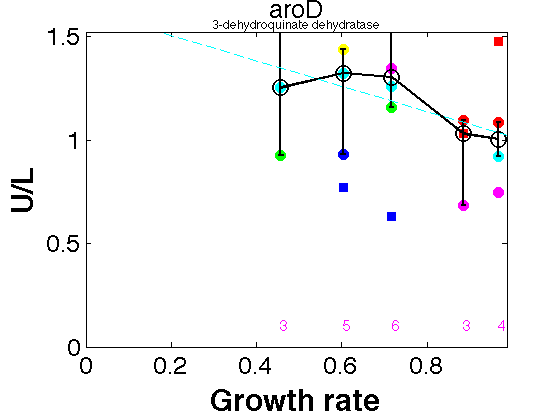

Supplement: Supplementary file 5 [file msb0011-0784-sd5.zip › Supplementary Dataset S1/Alim/aroD.png]

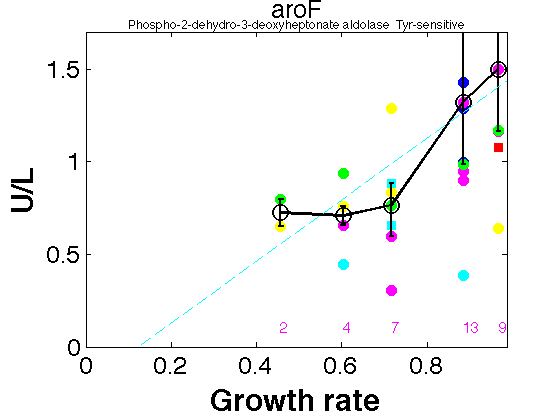

Supplement: Supplementary file 5 [file msb0011-0784-sd5.zip › Supplementary Dataset S1/Alim/aroF.png]

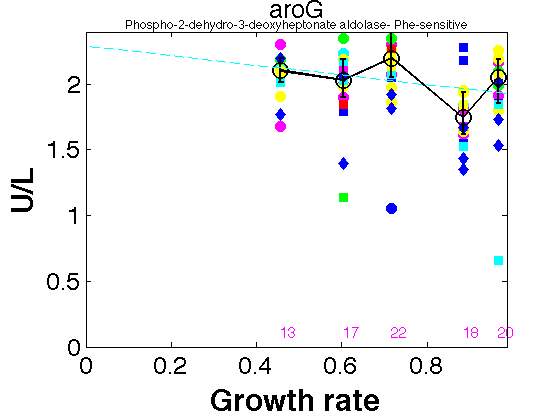

Supplement: Supplementary file 5 [file msb0011-0784-sd5.zip › Supplementary Dataset S1/Alim/aroG.png]

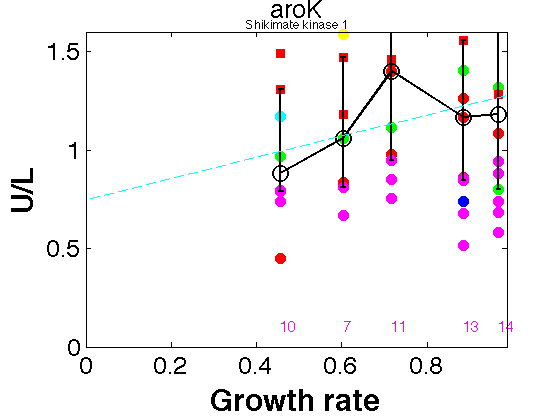

Supplement: Supplementary file 5 [file msb0011-0784-sd5.zip › Supplementary Dataset S1/Alim/aroK.png]

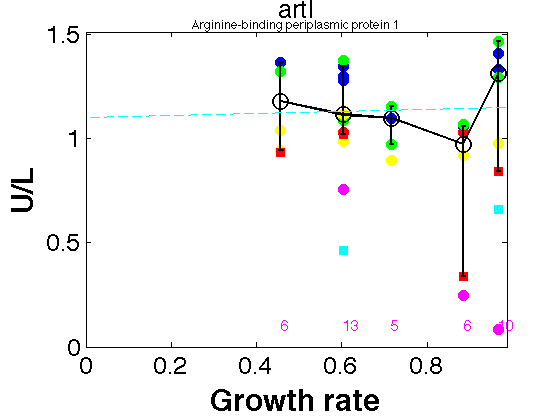

Supplement: Supplementary file 5 [file msb0011-0784-sd5.zip › Supplementary Dataset S1/Alim/artI.png]

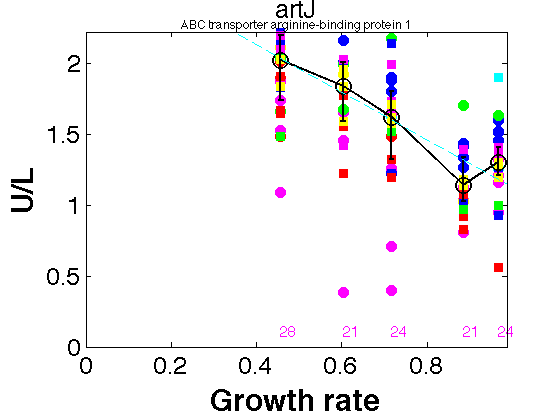

Supplement: Supplementary file 5 [file msb0011-0784-sd5.zip › Supplementary Dataset S1/Alim/artJ.png]

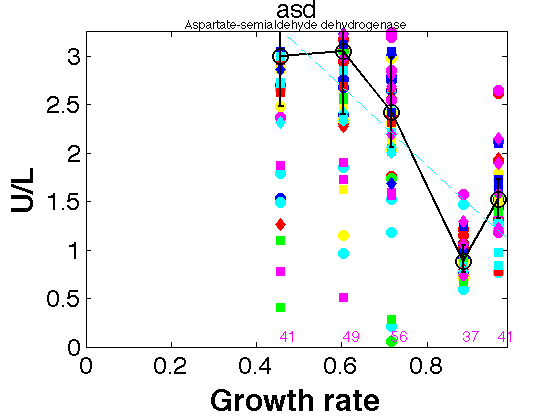

Supplement: Supplementary file 5 [file msb0011-0784-sd5.zip › Supplementary Dataset S1/Alim/asd.png]

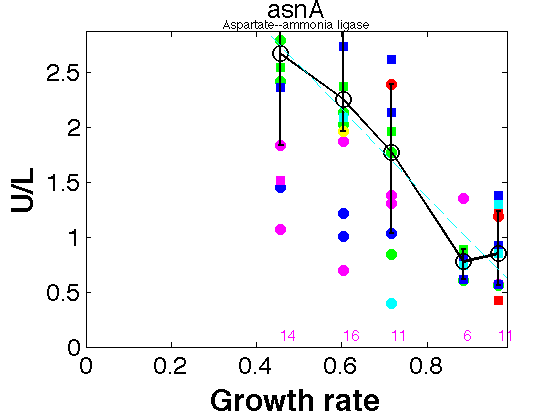

Supplement: Supplementary file 5 [file msb0011-0784-sd5.zip › Supplementary Dataset S1/Alim/asnA.png]

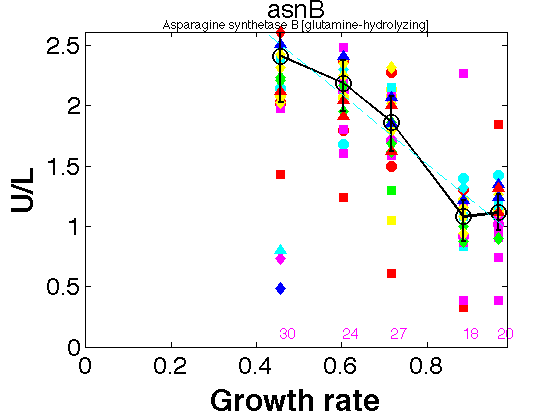

Supplement: Supplementary file 5 [file msb0011-0784-sd5.zip › Supplementary Dataset S1/Alim/asnB.png]

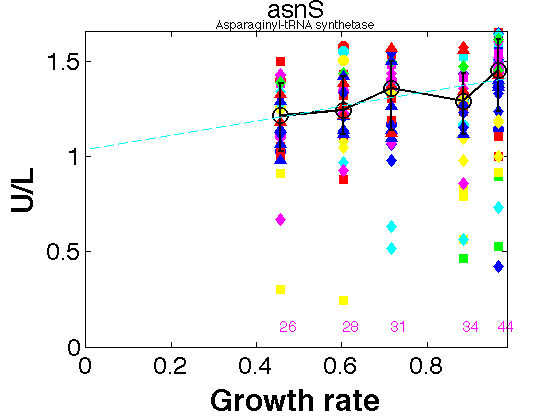

Supplement: Supplementary file 5 [file msb0011-0784-sd5.zip › Supplementary Dataset S1/Alim/asnS.png]

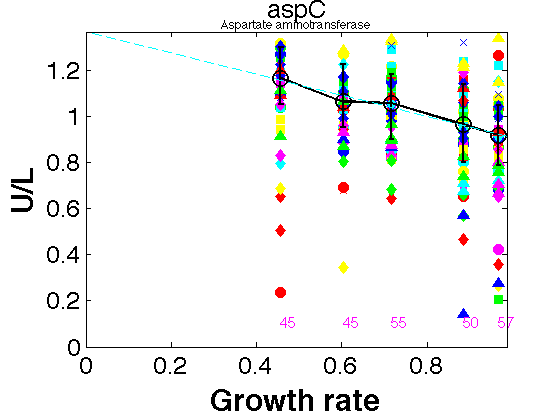

Supplement: Supplementary file 5 [file msb0011-0784-sd5.zip › Supplementary Dataset S1/Alim/aspC.png]

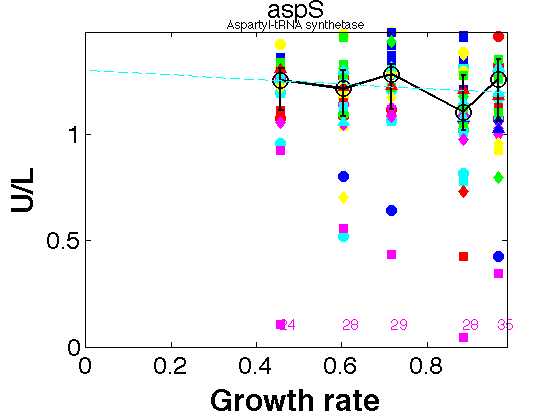

Supplement: Supplementary file 5 [file msb0011-0784-sd5.zip › Supplementary Dataset S1/Alim/aspS.png]

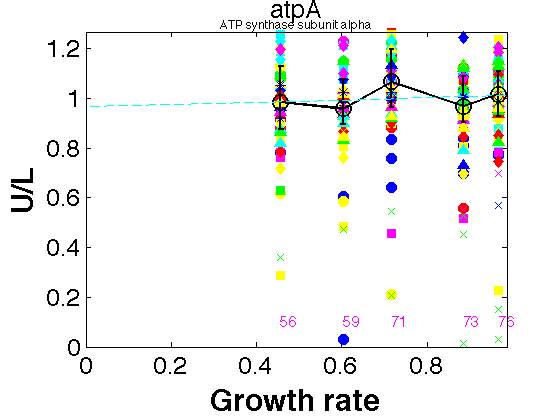

Supplement: Supplementary file 5 [file msb0011-0784-sd5.zip › Supplementary Dataset S1/Alim/atpA.png]

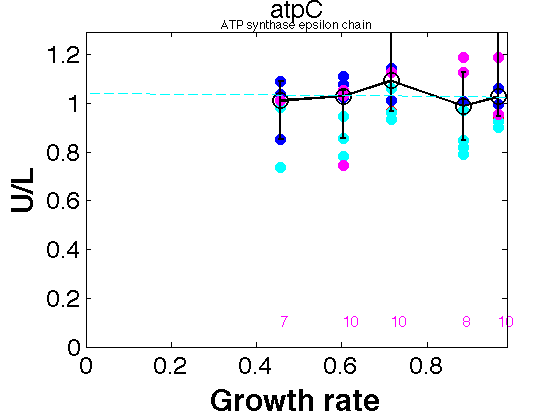

Supplement: Supplementary file 5 [file msb0011-0784-sd5.zip › Supplementary Dataset S1/Alim/atpC.png]

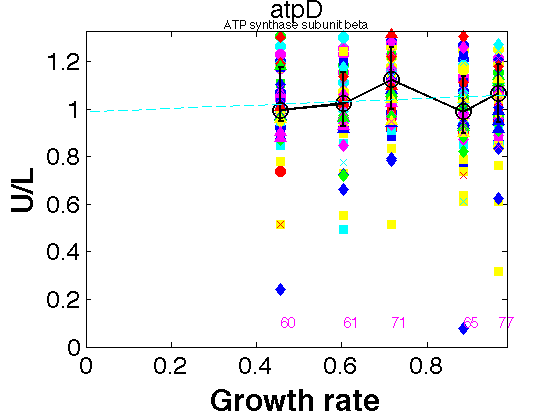

Supplement: Supplementary file 5 [file msb0011-0784-sd5.zip › Supplementary Dataset S1/Alim/atpD.png]

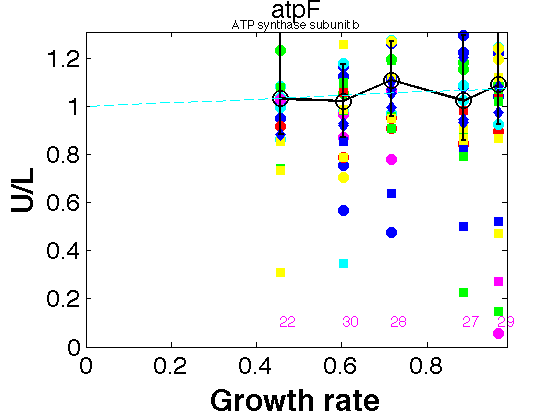

Supplement: Supplementary file 5 [file msb0011-0784-sd5.zip › Supplementary Dataset S1/Alim/atpF.png]

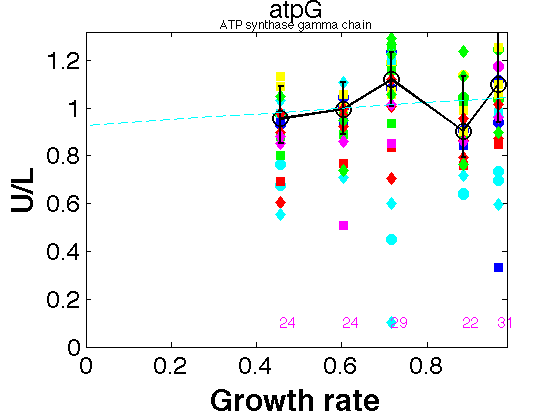

Supplement: Supplementary file 5 [file msb0011-0784-sd5.zip › Supplementary Dataset S1/Alim/atpG.png]

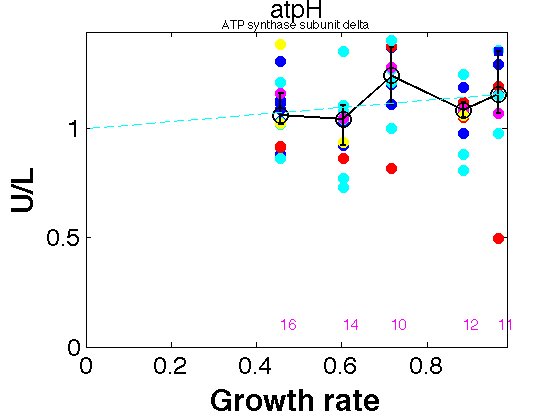

Supplement: Supplementary file 5 [file msb0011-0784-sd5.zip › Supplementary Dataset S1/Alim/atpH.png]

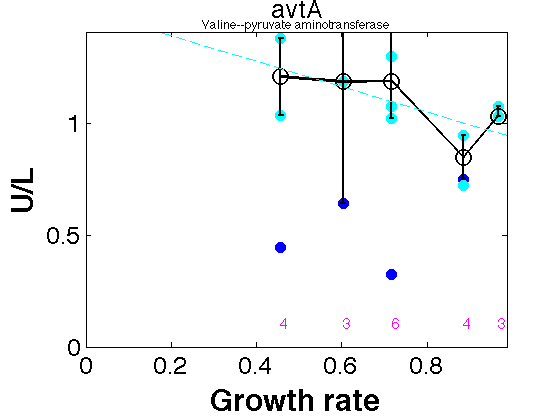

Supplement: Supplementary file 5 [file msb0011-0784-sd5.zip › Supplementary Dataset S1/Alim/avtA.png]

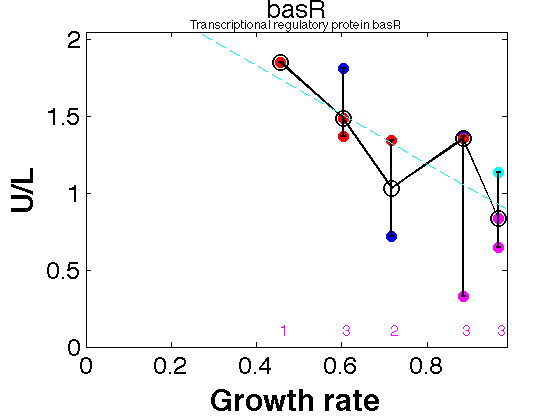

Supplement: Supplementary file 5 [file msb0011-0784-sd5.zip › Supplementary Dataset S1/Alim/basR.png]

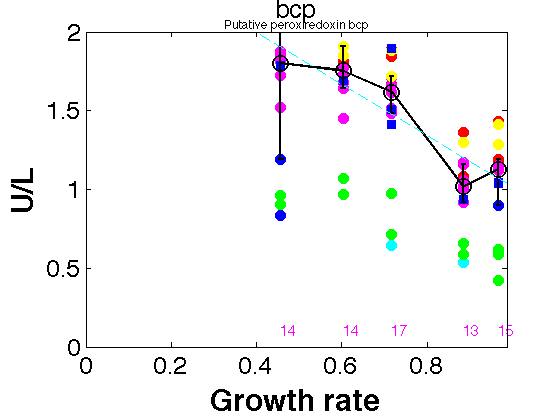

Supplement: Supplementary file 5 [file msb0011-0784-sd5.zip › Supplementary Dataset S1/Alim/bcp.png]

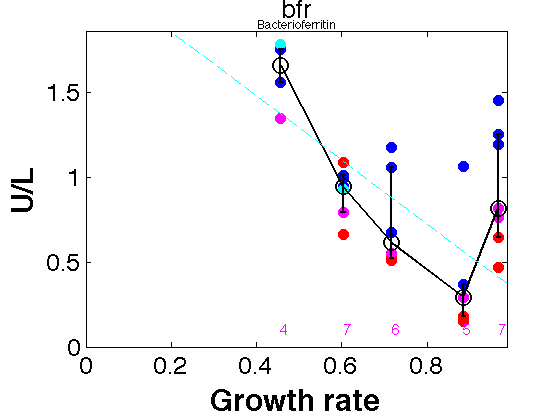

Supplement: Supplementary file 5 [file msb0011-0784-sd5.zip › Supplementary Dataset S1/Alim/bfr.png]

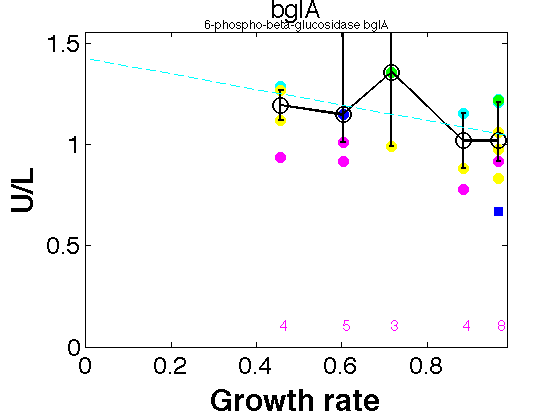

Supplement: Supplementary file 5 [file msb0011-0784-sd5.zip › Supplementary Dataset S1/Alim/bglA.png]

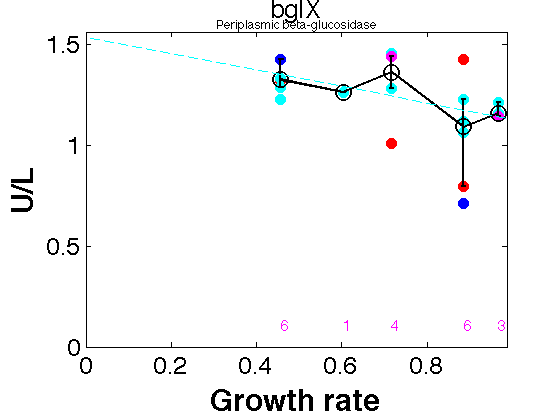

Supplement: Supplementary file 5 [file msb0011-0784-sd5.zip › Supplementary Dataset S1/Alim/bglX.png]

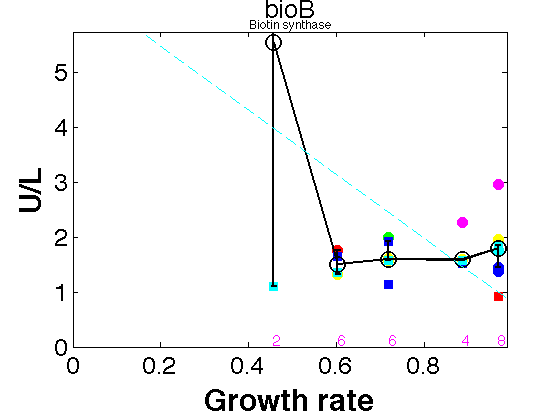

Supplement: Supplementary file 5 [file msb0011-0784-sd5.zip › Supplementary Dataset S1/Alim/bioB.png]

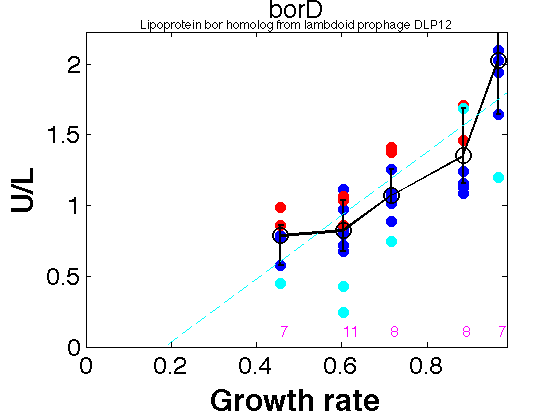

Supplement: Supplementary file 5 [file msb0011-0784-sd5.zip › Supplementary Dataset S1/Alim/borD.png]

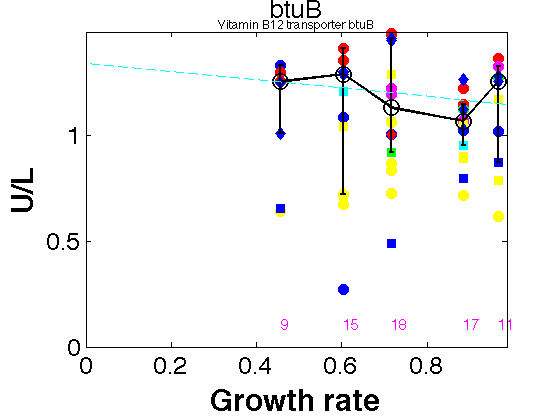

Supplement: Supplementary file 5 [file msb0011-0784-sd5.zip › Supplementary Dataset S1/Alim/btuB.png]

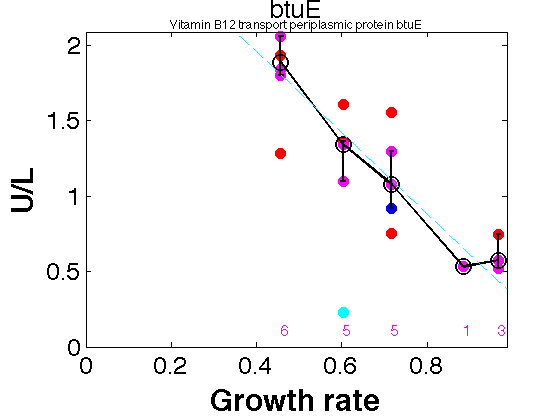

Supplement: Supplementary file 5 [file msb0011-0784-sd5.zip › Supplementary Dataset S1/Alim/btuE.png]

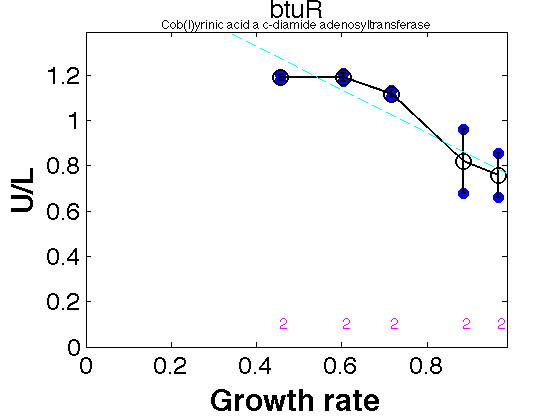

Supplement: Supplementary file 5 [file msb0011-0784-sd5.zip › Supplementary Dataset S1/Alim/btuR.png]

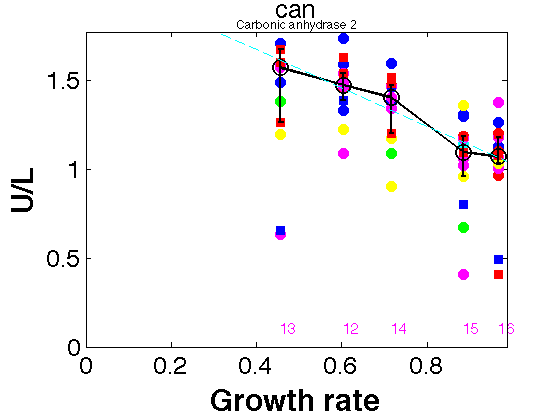

Supplement: Supplementary file 5 [file msb0011-0784-sd5.zip › Supplementary Dataset S1/Alim/can.png]

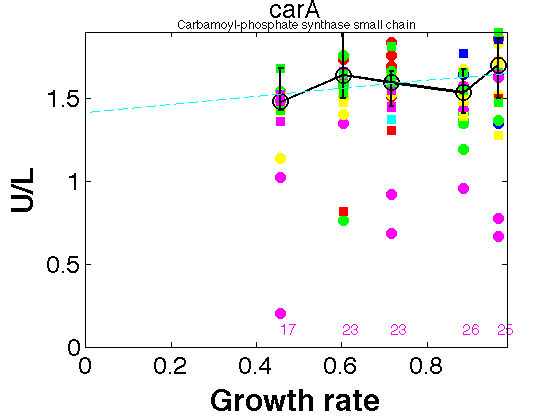

Supplement: Supplementary file 5 [file msb0011-0784-sd5.zip › Supplementary Dataset S1/Alim/carA.png]

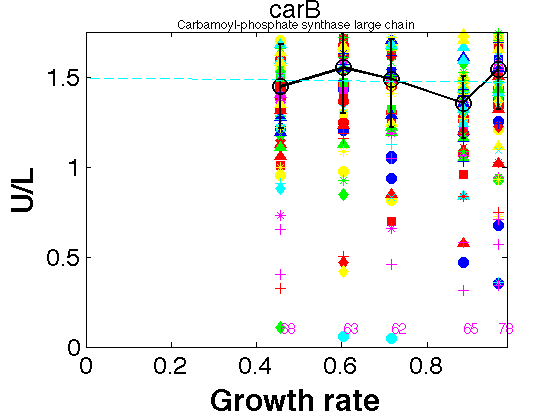

Supplement: Supplementary file 5 [file msb0011-0784-sd5.zip › Supplementary Dataset S1/Alim/carB.png]

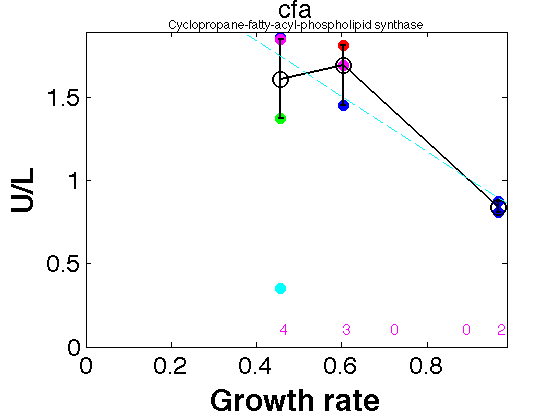

Supplement: Supplementary file 5 [file msb0011-0784-sd5.zip › Supplementary Dataset S1/Alim/cfa.png]

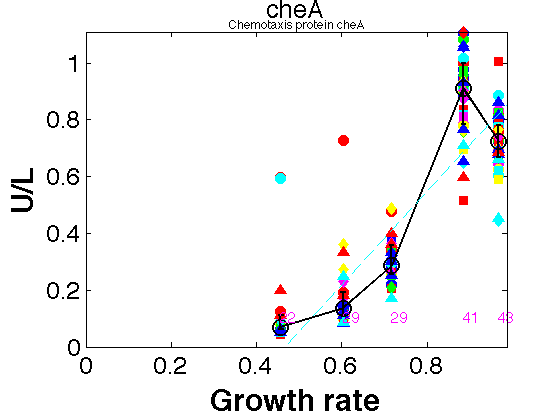

Supplement: Supplementary file 5 [file msb0011-0784-sd5.zip › Supplementary Dataset S1/Alim/cheA.png]

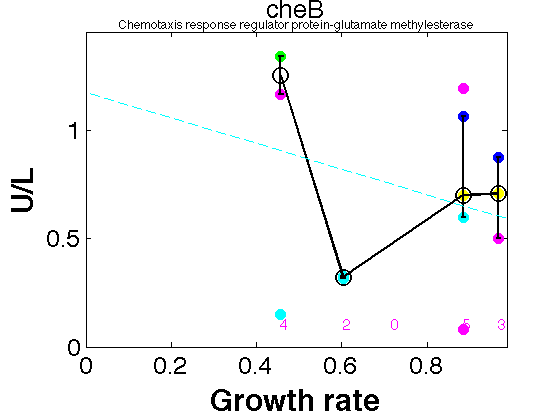

Supplement: Supplementary file 5 [file msb0011-0784-sd5.zip › Supplementary Dataset S1/Alim/cheB.png]

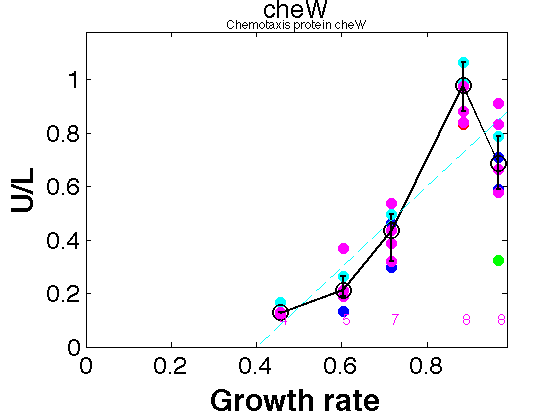

Supplement: Supplementary file 5 [file msb0011-0784-sd5.zip › Supplementary Dataset S1/Alim/cheW.png]

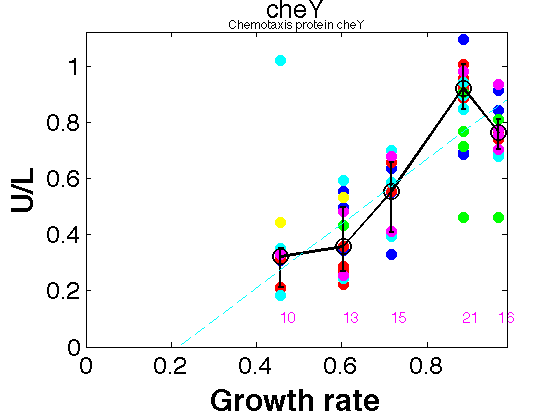

Supplement: Supplementary file 5 [file msb0011-0784-sd5.zip › Supplementary Dataset S1/Alim/cheY.png]

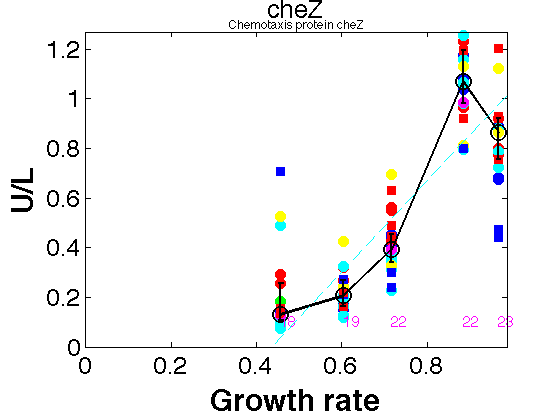

Supplement: Supplementary file 5 [file msb0011-0784-sd5.zip › Supplementary Dataset S1/Alim/cheZ.png]

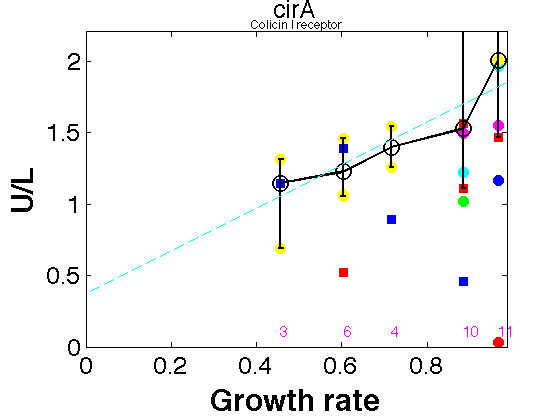

Supplement: Supplementary file 5 [file msb0011-0784-sd5.zip › Supplementary Dataset S1/Alim/cirA.png]

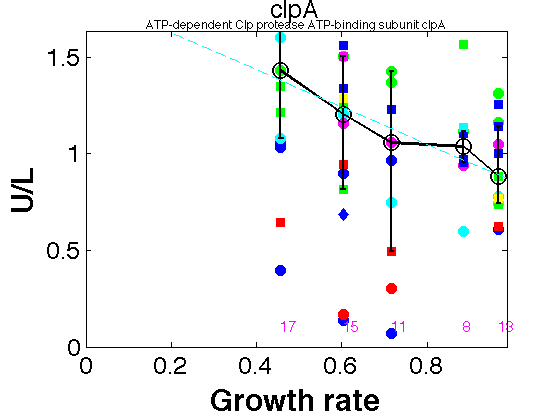

Supplement: Supplementary file 5 [file msb0011-0784-sd5.zip › Supplementary Dataset S1/Alim/clpA.png]

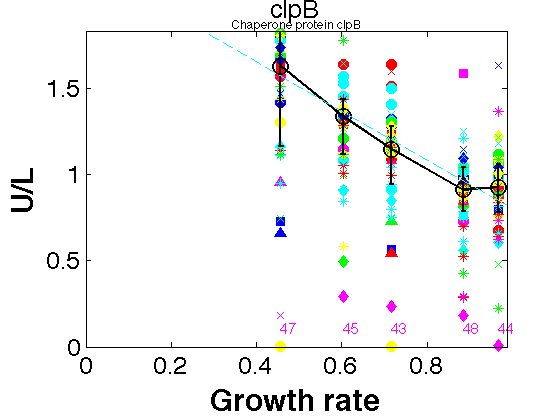

Supplement: Supplementary file 5 [file msb0011-0784-sd5.zip › Supplementary Dataset S1/Alim/clpB.png]

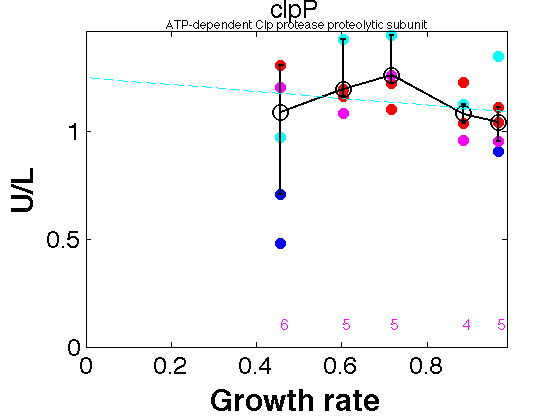

Supplement: Supplementary file 5 [file msb0011-0784-sd5.zip › Supplementary Dataset S1/Alim/clpP.png]

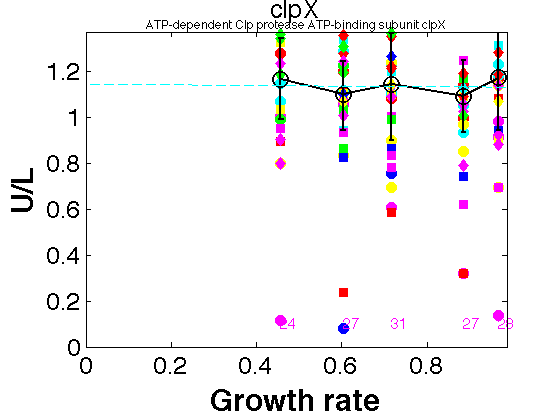

Supplement: Supplementary file 5 [file msb0011-0784-sd5.zip › Supplementary Dataset S1/Alim/clpX.png]

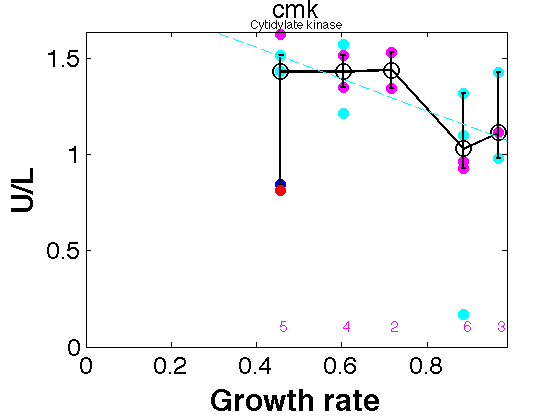

Supplement: Supplementary file 5 [file msb0011-0784-sd5.zip › Supplementary Dataset S1/Alim/cmk.png]

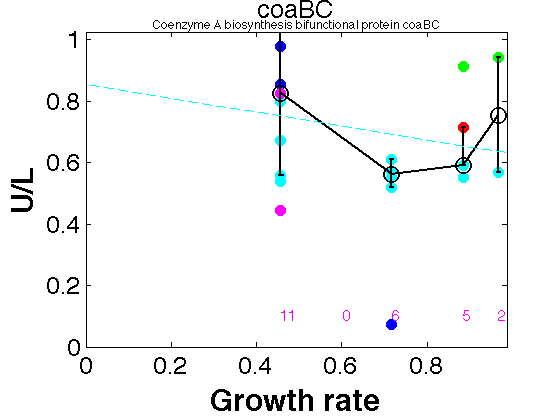

Supplement: Supplementary file 5 [file msb0011-0784-sd5.zip › Supplementary Dataset S1/Alim/coaBC.png]

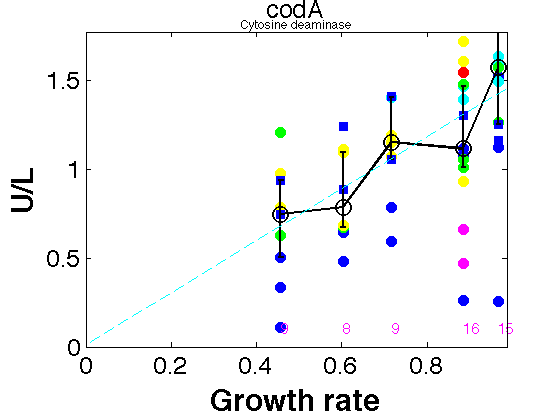

Supplement: Supplementary file 5 [file msb0011-0784-sd5.zip › Supplementary Dataset S1/Alim/codA.png]

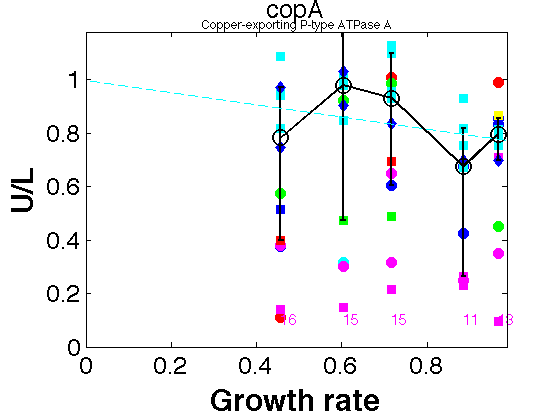

Supplement: Supplementary file 5 [file msb0011-0784-sd5.zip › Supplementary Dataset S1/Alim/copA.png]

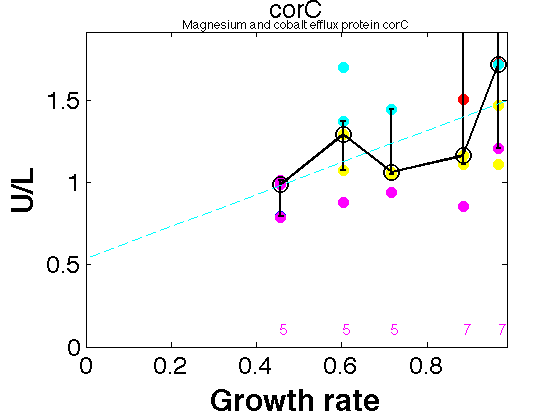

Supplement: Supplementary file 5 [file msb0011-0784-sd5.zip › Supplementary Dataset S1/Alim/corC.png]

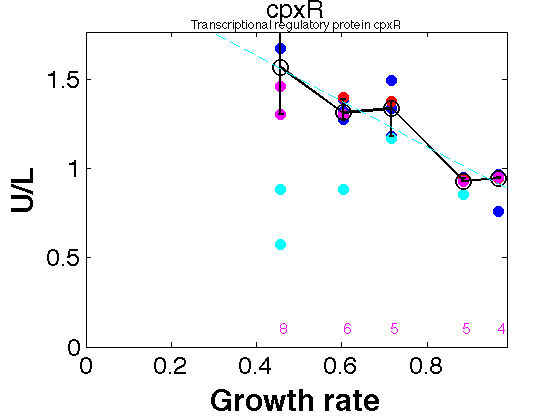

Supplement: Supplementary file 5 [file msb0011-0784-sd5.zip › Supplementary Dataset S1/Alim/cpxR.png]

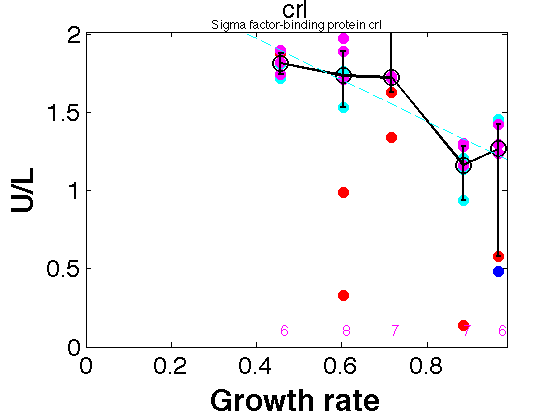

Supplement: Supplementary file 5 [file msb0011-0784-sd5.zip › Supplementary Dataset S1/Alim/crl.png]

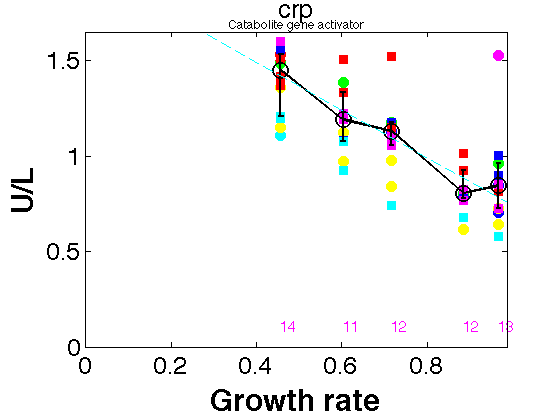

Supplement: Supplementary file 5 [file msb0011-0784-sd5.zip › Supplementary Dataset S1/Alim/crp.png]

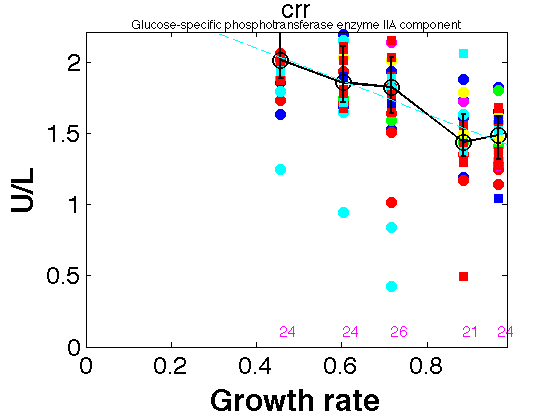

Supplement: Supplementary file 5 [file msb0011-0784-sd5.zip › Supplementary Dataset S1/Alim/crr.png]

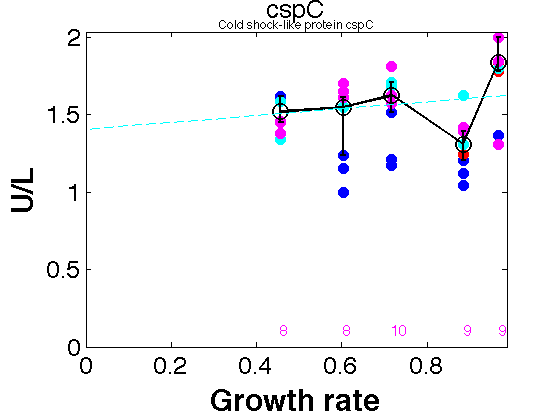

Supplement: Supplementary file 5 [file msb0011-0784-sd5.zip › Supplementary Dataset S1/Alim/cspC.png]
