# Supplementary material for: Quantitative proteomic analysis reveals a simple strategy of global resource allocation in bacteria
Source: Mol Syst Biol. 2015 Feb 12;11(2):784. doi: 10.15252/msb.20145697 (PMC4358657; doi:10.15252/msb.20145697)
Supplement: Supplementary file 7 [file msb0011-0784-sd7.pdf]

Quantitative proteomic analysis reveals a simple strategy of global resource allocation in bacteria

Sheng Hui, Joshua M. Silverman, Stephen S. Chen, David W. Erickson, Markus Basan, Jilong Wang, Terence Hwa, James R. Williamson

Corresponding author: Terence Hwa, University of California at San Diego

Review timeline:

Submission date:	18 August 2014
Editorial Decision:	29 September 2014
Revision received:	13 November 2014
Editorial Decision:	24 November 2014
Revision received:	11 December 2014
Accepted:	23 December 2014

Editor: Thomas Lemberger

Transaction Report:

(Note: With the exception of the correction of typographical or spelling errors that could be a source of ambiguity, letters and reports are not edited. The original formatting of letters and referee reports may not be reflected in this compilation.)

1st Editorial Decision 29 September 2014

Thank you again for submitting your work to Molecular Systems Biology. We have now heard back from the referees who accepted to evaluate the study. As you will see, the referees find the topic of your study of potential interest and are supportive. They raise however a series of concerns and make suggestions for modifications, which we would ask you to carefully address in a revision of the present work. The recommendations provided by the reviewers are clear in this regard and refer to the need for clarifications and some appropriate comparisons with previous works.

On a more editorial level, we would kindly ask you to deposit your proteomics dataset to an appropriate public repository (see http://msb.embopress.org/authorguide#a3.5) and include the respecitve accession number in a Data availability section at the end of the Material & Method section.

As suggested by reviewer #1 we would also ask you to submit the scripts used for the functional enrichment analysis as a zip compressed 'dataset' file (including a README file at the top level to explain the content of the archive).

Reviewer #1:

Background: Using transcriptional lacZ reporters the Hwa laboratory has previously shown linear relationships of gene transcription with growth rate upon nutrient limitation (You et al.). Genes required to cope with the stress imposed by the limitation increase linearly with reduced growth rate, while other genes decrease linearly. This intriguing linear relationship is elegantly explained by a theory of optimal resource allocation through feedback control in You et al.

Summary: The current paper by Hui et al. is an extension of this previous work, which aims to generalize this concept from a few reporters investigated previously to proteome-wide adaptations to nutrient limitations. Using mass-spectrometry, the authors measured protein levels in balanced exponential growth under limitations of carbon, nitrogen and ribosome inhibition. Each protein is then classified by the trend it shows under different nutrient limitations and proteins are grouped into sectors of the same trend. The following analysis focuses on the changes of these whole sectors upon nutrient limitation.
It is stated that, much like the transcriptional reporters of selected genes, the expression of these sectors follow a linear relation with growth rate. Using GO term analysis, it is shown that the sectors are enriched for proteins that are involved in dealing with the imposed stress that causes their upregulation. This generalizes the previous findings from the Hwa laboratory from reporters to the proteome. Finally, the data is used to fit the parameters of a coarse-grained model of metabolism and this model is used to predict proteome expression under a new conditions (using glycerol as a carbon source).

It is a significant step forward to work on the proteome as opposed to a few-genes approach. In this way, this work is an interesting extension of previous work. Notably, it seems that the theory presented is nearly identical with what was already published in a previous paper by the Hwa laboratory (You et al.)- any updates to the theory should be highlighted.

Specific points:
1. It is stated that the responses of most of the individual proteins (as plotted in Fig. S5) exhibits a linear trend. This is not apparent from the way the data is presented that this is the case. Fig S6 shows a cumulative distribution of R^2 values. It would be interesting to assess if non-linear models could explain the data better than linear models taking into account the experimental error of the experiment. i.e. are low R^2 values due to experimental noise or due to non-linear behavior of the proteins with respect to growth rate changes? It would also help to provide more plots of individual proteins, and to put all data online in a publically downloadable
2. The genes are separated into 2^3 classes based on their trend in the 3 limitations plus a class with no change in any. In principle, one may also expect genes that do not change in one condition, do so in other stresses. This would correspond to 3^3 groups. Were these cases not observed? Are there no genes with non-monotonic behavior?

3. It is unclear what defines the "top 3 groups" of proteome fractions. Based on the numbers given in Fig S9, the top three would be CdownAupRdown (209), CupAupRdown (159), I (156). What was used to rank the sectors? Not the number of proteins? The total mass? If so, in which conditions? This should be clarified in the text.

4. The bioinformatic method to use abundance-based GO term enrichment seems extraordinarily complex with extensive filtering. The method is described in great detail, and the approach is justified in the supplemental note. However, it is difficult for the reader to understand why such a complex method is used. It raises concerns on the robustness of the results to changes in cut-off values etc used in the filtering. It would be good to justify the complexity of this approach in the main text in simple terms.
There seems to be a typo in the description of the algorithm: "We then take the minimal value of Oi,l,t..." Should be maximal.
It would be important to share the bioinformatics software publicly on a server such as dryad.

5. One of the main points of the paper is that sectors scale linearly with growth rate. The data shown is most convincing for the R sector. At first sight, the data for the C-sector under C-limitation seems to be better explained by a non-linear function that flattens out at low growth rate. A similar trend is seen for C and A sector upon C and N limitation in glycerol (Fig 6.). The authors should assess whether non-linear models can explain the data better and if so discuss the discrepancy with their theory.

6. The question of whether the sector linear dependence with growth has a non-zero intercept with the y-axis is important: as cells approach zero growth, they seem to devote a fraction of some sectors towards future needs, as discussed in the text. The present theory does not seem to predict the quantitative size of this fraction. I believe that a previous optimality theory by Zaslaver et al (2009) made precise predictions for this fraction for ribosomal genes, which was compared to

genome-wide promoter activity measurements. This relevant experimental/theoretical study should be discussed more fully; the non-zero intersect property might be a way to distinguish between theories.

Overall this paper adds to our understanding of resources allocation on the proteome level in E coli. If the above points can be addressed, I strongly recommend publication.

Reviewer #2:

In the manuscript "Quantitative proteomic analysis reveals a simple strategy of global resource allocation in bacteria" by Hui et al, the authors investigate the strategy of gene expression in response to the environment. Specifically, they (1) characterized 1053 proteins of E.coli across 14 different growth conditions using mass spectrometry; (2) partitioned these 1053 proteins into 6 coarse-grained sectors (i.e. super-enzyme cluster), where the gene expression of each sector responses in distinct modes to the applied growth limitations; (3) found that the effective concentrations (i.e. mass fraction) of these 6 coarse-grained sectors correlated linearly with the growth rate, which can be characterized by a simple flux-matching model with only two global parameters.

The manuscript is very well written and the work presented in this paper is very thorough. In addition to the primary experiments listed above, the authors also comprehensively analyzed their data quality (in Fig. S3, S4, S6, S8, and Supplemental Note) and performed many control experiments (such as Fig. S10). Based on these measurement and analysis, the authors suggest a very convincing principle for global resource allocation in proteome economy of cells, which helps to understand the complicate strategy of gene regulation in response to the environment, i.e. one of the central aims of cell biology. The paper represents, in my opinion, a significant advance in the field. I strongly recommend publication in Molecule Systems Biology after the authors have addressed the following comments:

1. As mentioned in the introduction, there are many techniques for quantitative proteomic analysis. Could the authors comment a bit on the one that they chose (i.e. mass spectrometry)? What are the advantages and disadvantages? For future works, would other methods (e.g. ribosome profiling, deep sequencing, etc) bring more information to the field?
2. The main figures are not very easy to follow at first glance. It would be great if the authros could re-arrange the figures a bit. For instance, it might not be very necessary to put Fig. 2, 4, and 6 as three individual main figures, while leave Fig. S7 or S5 all in the supplementary material.
3. Table S6 is very helpful. It would be even better if the authors could also list out the meaning of different symbols somewhere (either in the main text or supplementary). For instance, is the 'effective concentration' (or 'mass fraction'); is the growth rate; etc.
4. The 'flux matching' section is a bit redundant. It might be better to move some of the details to the supplementary.
5. Introduction, paragraph 2, typo: "adjusts" -> "adjust"

Reviewer #3:

This manuscript continues the line of highly interesting and stimulating papers from the Terri Hwa lab discussing general connections between protein expression and cell growth rate. Here, a proteomic experiment was performed to define how changes in growth rate influence the genome-wide profile of protein expression. This was done by gradually imposing a limitation in one of the three central aspects of cellular activities: translation, carbon uptake and amino-acid metabolism. The authors identify groups of proteins whose expression levels correlate (positively or negatively) with growth rate, and classify them based on this growth-rate correlation in the to the different limitations. The data is summarized by a phenomenological model, which connects the protein expression with growth rate.
The most unique aspect of this paper is the model used to summarize the data. Although one can argue that this model is an over-simplified version of the reality, I find this approach, of summarizing highly complicated data in a simplified manner using only a few parameters,

refreshing and stimulating. I was in fact particularly surprised by the ability to predict basic features of how the proteome will be distributed when cells are growth in a new condition based on this simplified model.

Few comments:
Relation to previous literature:

1. The question of growth-dependent gene/protein expression was studied extensively in the budding yeast. There are several studies which generated a lot of high quality data (see e.g. - Brauer et al. (2008) Coordination of growth rate, cell cycle, stress response, and metabolic activity in yeast. Mol Biol Cell 19: 352-367; Castrillo JIet al. (2007) Growth control of the eukaryote cell: a systems biology study in yeast. J Biol 6: 4; Regenberg B, et al. (2006) Growth-rate regulated genes have profound impact on interpretation of transcriptome profiling in Saccharomyces cerevisiae. Genome Biol 7: R107; Levy S, et al. (2007) Strategy of transcription regulation in the budding yeast. PLoS One 2: e250; Airoldi et al. (2009) Predicting cellular growth from gene expression signatures. PLoS Comput Biol 5: e1000257 and perhaps others). Some of those studies performed a real through characterization including expression profiles, proteome profiles, metabolic profiles etc. None of those studies is discussed in the present MS. In fact, in one of those papers, a computational method was developed to predict growth rate from gene expression data. Other papers discussed whether the response to growth rate is direct or through a feed-forward like reading of the environment. I believe the data should not only be mentioned, but probably subject to some comparative analysis. It is really a large body of highly quantitative data of high quality that is very directly related to the present story and should therefore not be ignored.

2. The way by which limitations were imposed in the present papers were either drug, or differential expression of some enzymes. In contrast, in the previous studies mentioned above, growth rate limitations were imposed more naturally, through the use of a chemostat with a well-defined limiting factor. This difference may be fundamental to the way the cells react, since by introducing a drug or an effective mutation, the authors break the typical relationship between what the cells perceives as its environment and its actual growth potential. The fact that this could result in some differential response was discussed quite extensively; e.g. Brauer et al. show that when subjecting cells mutated in the lysine pathway to lysine limitations result in a response that is different from the typical growth-rate related response. The Botstein lab further demonstrated that 'normally' starving cells (due e.g. to running away of glucose) survive for a significantly longer time than cells which are starved due to some mutation (e.g. lack of an enzyme in the lysine pathway). Van-oudenaarden lab showed that glucose effects growth rate both through influx and through perception. Additional relevant paper from the Kishony lab treated more explicitly the case of drugs which limit translation. All in all, I believe the authors should discuss the 'un-natural' aspects of their perturbations and the possible implications.

Data presentation:
3. The authors analyzed their data by grouping together proteins with similar growth-rate correlation. I support this approach. However, the repeated claims of its originality and novelty are disturbing. I would carefully say that this grouping genes based on correlated expression is now the standard approach of analyzing high-throughput data and the first this one would do when trying to make sense of its overall behavior. This has to be changed in many places in the MS, including the results in discussion. Notation like 'super-enzymes' should be avoided in my opinion as they do not really add to the science.

4. The authors refer to 'general' growth-rate related behavior of gene groups based on two conditions only (in the third, the group behavior is changing). The fact that two different conditions result in a similar growth-rate dependent behavior is nice, but still, I would find it a bit dangerous to generalize based on two conditions. Moreover, it will not be too surprising to find that the classification of proteins into different sectors differs depending on the imposed limitation; for example, if only the C-limitation was imposed, the S and C sections would merge. Similarly, other conditions could exist that will further separate the different sectors, and will produce different classification. This doesn't invalidate the authors' approach for the analysis of the specific data, but does call for extra caution in the generalization and presentation of the reasons.

5. Much of the important information is given only in the SI. The figures remaining in the main text are a bit shallow. While I support simplicity, I believe this was over-done in this case, and in fact when reading the MS I had to continuously refer to the SI; figure 1, for example, contains no real data - once could easily add to it the information about how actual strains, characterize some of their behavior, with some controls etc.,. Figure 2 and figure 3 could easily be integrated, which would also help the reader. Figure 4 is almost identical to figure 2, and while it is nice to show that the computational curves fit, it could easily be integrated with figure this would leave at least two additional figures (or perhaps more) where the authors could show 'hard' data - to make the actual research and results more apparent, less artificial and more easy to appreciate.

Model:
6. The model provides a nice interpretation of the data, although it is not clear to me whether it goes beyond simply stating what one sees. For example, Eq. 4-5 is simply the statement expression of each sector correlates with growth rate. Eq. 9 is also the direct consequence of the pattern by which the different identified sectors change with the different limitations (that some sectors don't change while other change similarly in two of the three conditions). So it is a nice representation of the data, but I'm not sure whether it goes much beyond that.

7. As I said above, I do support the use of a simplified model to describe the data and generate biological hypothesis. Yet, the suggested biological interpretation of the model (or data representation?) raises many questions. Here are some examples:
a. What is the meaning of 'flux'? This may be clear in the case of ribosome (flux of proteins?) but is less defined in the other cases, and in particular considering the fact that each of the sector includes many proteins involved in many biological functions, metabolic pathways etc (and that, most probably although not shown, some of the proteins involved in relevant pathways are not part of the associated sectors). It is difficult to conceive that all proteins assigned e.g. to the a-section (and only them) define the flux of amino-acids, and the question is even more disturning in the case of the C-section. So that the fact that expression of the different sectors correlate with growth rate is given by the data, but the interpretation that this means that the respective protein produce some 'flux' that precisely match this growth rate is less clear and not well defined.
b. Even if one accepts (and I'm not sure I do) that there is some well-defined flux generated by each of the sectors why would there be a substantial portion of the proteins not contribution to this flux (the 'fixed' level)? Furthermore, why would this fixed level not contribution to the generation of flux remain constant in different conditions? From a biological perspective, this is something that is not easy to accept.
c. Another assumption is that the 'flux' depends on the relative fraction of the sector, rather than the total (absolute) level of enzyme. I understand the justification of this assumption in the context of the ribosome, but is this clearly generalized to other types of fluxes?

Reviewer #4:

In this work, Hui et al. have extended the previous phenomenological work from their groups.

In the current study, the authors control the growth rate of E. coli cultures using three different growth limitations: limiting carbon intake, limiting nitrogen intake, and inhibiting translation. They find that for most of quantified enzymes, individual proteins either increase or decrease in abundance as a function of growth rate where the sign of the effect (increase or decrease) depends on the particular form of limitations (carbon, nitrogen, and translation). Based on individual enzyme's response, they cluster enzymes into 9 broad categories wherein enzymes belonging to each category respond to growth limitations in a particular way (e.g. increase with carbon, decrease with nitrogen, and translation). Notably, enzymes belonging to each of the category also have specific enrichment in GO annotation terms. The authors then construct a simple flux-matching model which allows them to predict coarse-grained proteome response as a function of combinatorial growth limitations.

I generally like this work. It is simple, phenomenological, and also provides the community with a good resource on enzyme abundances as a function of various growth limitations; which may be further analyzed by other researchers. I recommend publication of this paper in MSB after certain

revisions.

1) I would like the authors to make the paper more readable by shortening it considerably. In my opinion, the introduction is longer than necessary. For example, the authors can do away with the statistical physics analogies. Also, the discussion on enrichment of GO annotations in each sector can also be considerably shortened by simply highlighting the findings in the main text and moving some of the details to supplementary section. In the section on flux matching, while it is clear how one arrives at Eq. 1 to 5, the introduction of f due to contamination of the so-called S sector is somewhat unclear. The authors may want to discuss that in some detail. Finally, almost half of the discussion section is spent in a recap of the introduction and the results. I believe that this is unnecessary. The authors can use the discussion section to briefly discuss basic principles governing cellular growth and future experimental and theoretical direction.

2) Minor point that the authors may wish to address: The authors use the classification term "upwards" for enzymes whose abundance is negatively correlated with growth rate (with a downward slope) and vice versa. I suggest that the authors reverse this somewhat confusing notation.

1st Revision - authors' response 13 November 2014

(see next page)

Reviewer #1:

Background: Using transcriptional lacZ reporters the Hwa laboratory has previously shown linear relationships of gene transcription with growth rate upon nutrient limitation (You et al.). Genes required to cope with the stress imposed by the limitation increase linearly with reduced growth rate, while other genes decrease linearly. This intriguing linear relationship is elegantly explained by a theory of optimal resource allocation through feedback control in You et al.

Summary: The current paper by Hui et al. is an extension of this previous work, which aims to generalize this concept from a few reporters investigated previously to proteome-wide adaptations to nutrient limitations. Using mass-spectrometry, the authors measured protein levels in balanced exponential growth under limitations of carbon, nitrogen and ribosome inhibition. Each protein is then classified by the trend it shows under different nutrient limitations and proteins are grouped into sectors of the same trend. The following analysis focuses on the changes of these whole sectors upon nutrient limitation.

It is stated that, much like the transcriptional reporters of selected genes, the expression of these sectors follow a linear relation with growth rate. Using GO term analysis, it is shown that the sectors are enriched for proteins that are involved in dealing with the imposed stress that causes their upregulation. This generalizes the previous findings from the Hwa laboratory from reporters to the proteome. Finally, the data is used to fit the parameters of a coarse-grained model of metabolism and this model is used to predict proteome expression under a new conditions (using glycerol as a carbon source).

It is a significant step forward to work on the proteome as opposed to a few-genes approach. In this way, this work is an interesting extension of previous work. Notably, it seems that the theory presented is nearly identical with what was already published in a previous paper by the Hwa laboratory (You et al.)- any updates to the theory should be highlighted.

We appreciate the reviewer's accurate and thorough summary of our work, and its relation with our previous work.

The reviewer suggested to highlight the updates of the current model to a previous theory. The theory the reviewer refers to is in fact buried in the Supplemental Text of You et al, which most readers we know take as an experimental study of carbon-nitrogen coordination, in particular for elucidating the source of cAMP signaling. We are grateful for this reviewer's detailed reading of that work; but unless the Editor decides otherwise, we think it is useful to have a self-contained presentation of the coarse-grained resource allocation theory in this work. We now also explicitly state the similarity and differences with respect to previous work, by adding a sentence

right after Eq. [2],

"The model is an extension of a similar model proposed in a previous work based on the growth-rate dependences of a few reporter genes (You et l. 2013) "

and another sentence right before the introduction of Eq. [7],

"Note that in a previous proteome partition model (You et al. 2013) based on measurements of a few reporter genes the hypothesized C- and A- sectors correspond respectively to the $\widetilde{C}$ - and $\widetilde{A}$ - sectors here, whereas the possibility of the S-sector was not anticipated."

We hope this adequately addresses the reviewer's concern.

Specific points:
1. It is stated that the responses of most of the individual proteins (as plotted in Fig. S5) exhibits a linear trend. This is not apparent from the way the data is presented that this is the case. Fig S6 shows a cumulative distribution of R^2 values. It would be interesting to assess if non-linear models could explain the data better than linear models taking into account the experimental error of the experiment. i.e. are low R^2 values due to experimental noise or due to non-linear behavior of the proteins with respect to growth rate changes?

This is indeed a very important issue as the linearity of the data provides the basis for the simple binary classification we used.

Our linear analysis was initially motivated by the striking linear growth-rate dependence in the expression of a number of exemplary reporters of catabolic and biosynthetic gene expression established in You et al. We were further encouraged by the prevalence of linearity in our data, as indicated by the cumulative distribution of R^2 values in the original Fig. S6 (Fig. E6A in the revised version). However, the reviewer is right on the existence of some low R^2 values of among the linear fits.

One reason could be that while our method is quite precise (with a precision of ~18% across the proteome) for an -omics method, it is still not comparable to quantitative traditional methods that focus on individual proteins or protein complexes as used in You et al. For example, the growth-rate dependence of ribosome can be determined by measuring the total RNA and total protein, which results in strikingly linear relation (Scott et al. 2010, You et al. 2013, and numerous historical studies). The behaviors of individual ribosomal proteins as measured by the mass spectrometry method show quite some variability among themselves, suggesting the imperfect linearity may result from limited precision of the method. We have added a new plot, Fig. E7A to illustrate this point.

Another reason for the existence of low R^2 values is that those cases mostly

correspond to weak growth-rate dependences, i.e., small fold changes in protein expression levels. This is shown in two new plots, Fig. E7B and E7C. The analysis suggests that the low R^2 values are mostly due to experimental noise, as small noise for a flat response can lead to a low value of R^2.

To quantify how well the linear model captures the behaviors of individual proteins as compared to a non-linear model, in the revised version, we have carried out a quadratic fit using the A-limitation data set as an example. The comparison between the linear fit quality and the quadratic fit quality is shown in the newly added plot of Fig. E6B. Though the quadratic fit obviously has a higher average value of R^2 (with one more parameter), it does not mean that it describes the data better because it also has higher value of R^2 for random data. With respect to the performance for random data, we found that the linear fit outperforms the quadratic fit significantly, supporting the usage of linear model for the data.

It would also help to provide more plots of individual proteins, and to put all data online in a publically downloadable

We now submit plots of all individual proteins as an Expanded View Dataset, and have deposited our mass spectrometry data to a public repository, and added a subsection “Data availability” at the end of the “Materials and Methods” section.

2. The genes are separated into 2^3 classes based on their trend in the 3 limitations plus a class with no change in any. In principle, one may also expect genes that do not change in one condition, do so in other stresses. This would correspond to 3^3 groups. Were these cases not observed? Are there no genes with non-monotonic behavior?

We have in fact started the analysis by classifying the proteins into 3^3 groups, using the sensitivity of the method (~25%) as the cutoff value for determining ‘no change’ protein expression. To illustrate the results we got, let us focus on the four groups (out of 27 groups) where proteins go up under R-limitation (indicated by the third letter ‘U’ in the titles of the four plots shown below) but are classified as either constant (indicated by ‘C’ in the plot titles) or down (indicated by ‘D’ in the plot titles) under the C- and A- limitations. The number in the parenthesis of a plot title is the number of proteins classified into the group. As can be seen from the plot below, proteins that exhibit downward trends upon C- and A- limitations were separated into the groups DDU, CCU, and DCU, and this details of this separation depends arbitrarily on the cutoff value imposed, i.e., how big a slope must the trend exhibit before we take it to be in the up or down group.

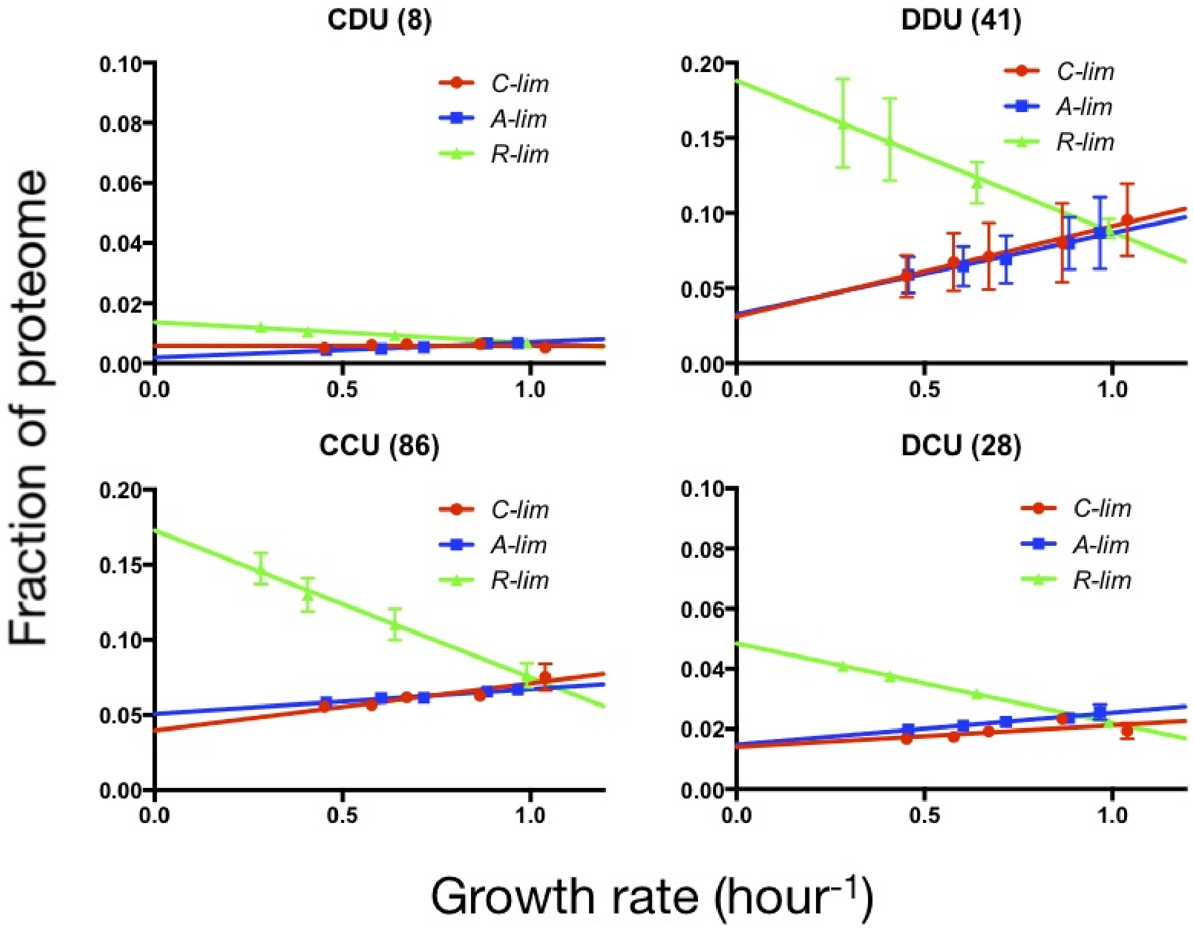

Instead of trying to understand the behavior of each of the four groups, whose sizes depend on the exact cutoff value, we decided to take the simplest classifying method, i.e., the binary classification, with which the above four groups would be mostly grouped into one group that goes up under R-limitation and goes down under the other two limitations.

To assess possible misclassification due to cases of relatively flat growth-rate dependence, in the revised version we carried out a probabilistic binary classification, which shows that the effect of misclassification is limited (Expanded View Text 2).

3. It is unclear what defines the "top 3 groups" of proteome fractions. Based on the numbers given in Fig S9, the top three would be CdownAupRdown (209), CupAupRdown (159), I (156). What was used to rank the sectors? Not the number of proteins? The total mass? If so, in which conditions? This should be clarified in the text.

We thank the reviewer for pointing out this confusing statement. In the revised version, we have added “Ranked by the extent the fraction varies (indicated by the difference between the maximal and minimal intercepts on the y-axis)” in front of the sentence of

“the top three groups…”.

4. The bioinformatic method to use abundance-based GO term enrichment seems extraordinarily complex with extensive filtering. The method is described in great detail, and the approach is justified in the supplemental note. However, it is difficult for the reader to understand why such a complex method is used. It raises concerns on the robustness of the results to changes in cut-off values etc used in the filtering. It would be good to justify the complexity of this approach in the main text in simple terms.

In response to the reviewer’s suggestion, we have now included the following sentences in the opening paragraph of the GO analysis section to briefly summarize the procedure:

“To reach such a list of GO terms, instead of calculating a single score of one measure (e.g., enrichment) for each GO term as in many published GO analyses, we have taken a multi-step procedure to search for the best representing GO terms by examining a number of different measures such as coverage and overlap. The procedure leads to only a few GO terms accounting for more than 60% of the proteome in the sector; see Expanded View Text 3. “

Also to improving the readability of the supplemental note (Expanded View Text 3 in the current version), we have streamlined it considerably, including adding a more detailed overview of the method at the beginning of the note and structuring the content by dividing it into subsections.

There seems to be a typo in the description of the algorithm: "We then take the minimal value of Oi,I,t..." Should be maximal.

We thank the reviewer for pointing out the typo. It has been corrected.

It would be important to share the bioinformatics software publicly on a server such as dryad.

The annotated matlab code for implementing the method is now submitted as an Expanded View Code.

5. One of the main points of the paper is that sectors scale linearly with growth rate. The data shown is most convincing for the R sector. At first sight, the data for the C-sector under C-limitation seems to be better explained by a non-linear function that flattens out at low growth rate. A similar trend is seen for C and A sector upon C and N limitation in glycerol (Fig 6.). The authors should assess whether non-linear models can explain the data better and if so discuss the discrepancy with their theory.

The reviewer rightly pointed out that the growth-rate dependences may not be linear. By no means did we intend to rule out nonlinearity in the data. With our current

analysis, we only argue that a linear model can already capture the data rather well (as indicated by the high R^2 of the overall linear fit), and it allows a simple understanding (i.e., flux-matching with proteome constraint) and can successfully predict sector behaviors for new growth conditions. We encourage other investigators to examine our data and we would be more than happy to see nonlinear models based on biological mechanisms that can better account for the data.

We reiterate here that our goal is not to find the function that best-fit the data, but to describe the data by a sufficiently simple model that give us some predictive power. In this regard, we cite below the comment of Reviewer 3:

“The most unique aspect of this paper is the model used to summarize the data. Although one can argue that this model is an over-simplified version of the reality, I find this approach, of summarizing highly complicated data in a simplified manner using only a few parameters, refreshing and stimulating. I was in fact particularly surprised by the ability to predict basic features of how the proteome will be distributed when cells are growth in a new condition based on this simplified model.”

We have also emphasized this point in the revision with the following sentences added at the end of the discussion section:

“We emphasize that our goal here is to describe the data by a minimal model with predictive power, we do not rule out nonlinear generalizations of the model presented here.”

6. The question of whether the sector linear dependence with growth has a non-zero intercept with the y-axis is important: as cells approach zero growth, they seem to devote a fraction of some sectors towards future needs, as discussed in the text. The present theory does not seem to predict the quantitative size of this fraction. I believe that a previous optimality theory by Zaslaver et al (2009) made precise predictions for this fraction for ribosomal genes, which was compared to genome-wide promoter activity measurements. This relevant experimental/theoretical study should be discussed more fully; the non-zero intercept property might be a way to distinguish between theories.

We thank the reviewer for bringing up the issue of the y-axis intercept which is very important. Our theory indeed does not address this issue. For the ribosomes, there have been a number of attempts rationalizing where it comes from, which is the first step towards a theory predicting its magnitude. In the paper by Scott et al, a description is given in the Supp Information on early attempts of attributing this intercept to free ribosomes or non-translating ribosomes. In a recent paper by Klumpp et al. (PNAS 2013), a y-axis intercept is seen as the remnant of a growth-rate dependence of the tRNA section, which is a substrate of the ribosomes but treated in the coarse-grained model as a part of the R-sector.

As to the model presented in the paper by Zaslaver et al. (2009), we believe an important assumption was made without justification: Eqn. (3) on page 6 of that paper states that the concentration of the substrate is proportional to the concentration of the metabolic proteins. As a microscopic constituent relation, we suggest the equation is incorrect because the substrate is not only produced by the metabolic proteins but also consumed by the ribosomal proteins at the same time (as stated in Eq. (2) of the model), and the concentration of the substrate should be determined by both the influx (production by metabolic proteins) and outflux (usage by ribosomal proteins). As an empirical relation, this relation is proposed without basis. In fact, the substrates of the ribosomes, the tRNA, shows a positive linear correlation with the growth rate (see Klumpp et al, PNAS 2013), which is the opposite of the relation proposed.

Regarding the magnitude of the R-sector offset: Zaslaver et al's model gives it in terms of a Hill coefficient of some Hill function relating the substrate of the ribosome. As no identification was made on the substrate and the nature of the Hill function, the model actually does not give a quantitative prediction on the offset, other than stating that this Hill coefficient needs to be of the order n=6 to explain the observed data. Looking at the empirical data, different strains of *E. coli* appear to have clearly offsets of different magnitude. This is not consistent with the universal offset predicted by Zaslaver et al's model.

Despite our reservation of the Zaslaver et al's model, we did cite this work along with a number of others that attempt to tackle the molecular origin of this offset. It is discussed towards the end of Discussion of the revised text.

Overall this paper adds to our understanding of resources allocation on the proteome level in E coli. If the above points can be addressed, I strongly recommend publication.

Reviewer #2:

In the manuscript "Quantitative proteomic analysis reveals a simple strategy of global resource allocation in bacteria" by Hui et al, the authors investigate the strategy of gene expression in response to the environment. Specifically, they (1) characterized 1053 proteins of E.coli across 14 different growth conditions using mass spectrometry; (2) partitioned these 1053 proteins into 6 coarse-grained sectors (i.e. super-enzyme cluster), where the gene expression of each sector responses in distinct modes to the applied growth limitations; (3) found that the effective concentrations (i.e. mass fraction) of these 6 coarse-grained sectors correlated linearly with the growth rate, which can be characterized by a simple flux-matching model with only two global parameters.

The manuscript is very well written and the work presented in this paper is very thorough. In addition to the primary experiments listed above, the authors also

comprehensively analyzed their data quality (in Fig. S3, S4, S6, S8, and Supplemental Note) and performed many control experiments (such as Fig. S10). Based on these measurement and analysis, the authors suggest a very convincing principle for global resource allocation in proteome economy of cells, which helps to understand the complicate strategy of gene regulation in response to the environment, i.e. one of the central aims of cell biology. The paper represents, in my opinion, a significant advance in the field. I strongly recommend publication in Molecule Systems Biology after the authors have addressed the following comments:

We thank the reviewer for the positive remarks.

1. As mentioned in the introduction, there are many techniques for quantitative proteomic analysis. Could the authors comment a bit on the one that they chose (i.e. mass spectrometry)? What are the advantages and disadvantages? For future works, would other methods (e.g. ribosome profiling, deep sequencing, etc) bring more information to the field?

Our focus is limited to studying the economics of protein expression, hence we needed to employ a quantitative method that directly probes protein abundances. Among the available methods are 2D gel, mass spec, and ribosome profiling. We would not employ mRNA-related methods (e.g., mRNA microarrays, RNA-seq) because the relation between mRNA abundance and protein abundance is in general not straightforward when the translational capacity of the ribosomes is limited. Information on mRNA abundances would be very useful in understanding how protein abundances become what they are; but it is beyond the scope of this study.

Among the proteomic approaches mentioned above, 2D gel requires a high-throughput method of protein identification which we have not been able to work out. Ribosome profiling is a great alternative to mass spectroscopy which was published recently (Li et al, Cell 2014), during the final preparation of this work. Based on the results of Li et al, ribosome profiling is more quantitative and has an advantage over mass spec in determining the *absolute* abundance of individual proteins. However, our impression is that ribosome profiling is much more tedious to set up. E.g., several hundred mL of each culture is needed, and must be collected very rapidly, whereas for proteomics, one only needs to collect cell lysate from 1 mL of culture.

Mass spec was chosen due to the ability to make high precision *relative* quantitations. The method we use allows relative abundances between and experimental and control sample to be determined with a precision of +/- 18% on a proteome wide scale, by analysis of MS spectra, in conjunction with protein identification by MS/MS. Only with this precision can we quantify the linear trends in the individual proteome fraction that vary over a narrow range. In order to aggregate the individual proteins into sectors, we used a form of spectral counting as a proxy for the absolute abundance. Thus, for our study where absolute abundances is needed only at the coarse grained level but over many different conditions, mass spec is a more versatile approach.

Here, we remark at length on the methods suggested by the reviewer and speculate on how they can extend our understanding. We have inserted a condensed discussion of these points in the discussion section.

Ribosome profiling has been applied to measure the distribution of mRNA involved in active translation. By making core assumptions of balanced growth, negligible protein degradation, and a sequence-independent peptide elongation rate, the fractional ribosome occupancy of a given mRNA f_i can be recast as a copy number via $f_i \to \gamma_{tln} c_R f_i / \lambda l_i$, where γ_{tln} is the peptide elongation rate, λ is the growth rate, l_i the protein length, and c_R is the (steady-state) concentration of ribosomes in the cell. Of course, ribosome profiling could be used to measure changes in gene expression level in our limitation series. Significant discrepancies between the two measurements would necessarily reflect a non-uniform γ_{tln}, i.e. sequence dependence, or non-negligible β_{deg}.

Significant degradation (e.g. during growth transitions, starvation, etc.) would enter as a major cost in the protein expression puzzle, and would be problematic for our relative quantitation technique. Similarly, ribosome profiling would chronically overestimate protein levels. However, the dual use of a pulse labeling mass spectrometry method (to measure β_{deg}), with profiling could rescue both methods to provide accurate copy numbers (which is corrected by mapping $\lambda \to \lambda + \beta_{deg}$ in the relation above) and therefore costs (fixing our model) in light of the degradation.

Under the assumptions typically applied, the ribosome profiling measurement is a proxy for protein concentration, and does not yield more general information about the broader mRNA population, i.e. significant action of a riboswitch or small RNA would be invisible. If it is true that the patterns we see in gene expression are not congruent with the relative levels of mRNA transcript, it would be a result of these kinds of phenomena. Were one to answer this question, mRNA sequencing would be an obvious choice of technique.

An urgent question raised by our results is the basis of the coordination for the A and S sectors across limitations. Coordination for the C sector is attributed to cAMP/Crp, while R sector coordination is set by intracellular ppGpp levels. While nitrogen assimilation enzymes in the A sector are known to be regulated by the PII/Ntr system, the scope of the A sector response exceeds the known reach of NtrC. Likewise, the identical response of S sector enzymes to carbon and nitrogen limitation demands explanation. Profiling the global distribution of transcription factors would help unravel the regulatory basis for the gene expression patterns we report.

2. The main figures are not very easy to follow at first glance. It would be great if the authros could re-arrange the figures a bit. For instance, it might not be very necessary to put Fig. 2, 4, and 6 as three individual main figures, while leave Fig. S7 or S5 all in

the supplementary material.

In response to the reviewer's suggestion, we have now used the original Fig. S5 (the heat map of all the protein expressions) as part of a main figure (Fig. 2 in the current version). Together with the original Fig. S3AB, which show some of the control experiment results, the current Fig. 2 now contains information on both mass spectrometry method and data. We hope that by putting the underlying mass spectrometry data upon which the original Fig. 2 is based on, it would be easier to follow the figures.

We considered the reviewer's suggestion to move some of the original Fig. 2, 4, and 6 (Fig. 3, 5, and 7 in current version) to the supplements: Fig. 4 (Fig. 5 in the revised version) summarizes the results of the model description and comprise the main conclusion of the whole work; so it has to remain as a main figure. Fig. 6 (Fig. 7 in the revision) contains both of the two glycerol test data sets, and also a new protein overexpression data set. The comparison between prediction and test is an important component of our study and we feel should also remain as a main figure. The only figure that may be moved to the supplement is therefore Fig. 2 of the original version. However, this figure is referred to 18 times in the main text. Given that Reviewer #3 has complained about the excessive reference to supplemental figures, we decided to keep this figure in the main text as well (as Fig. 3 of the revision). We are also happy to move it to the supplement if the editor favors this arrangement.

3. Table S6 is very helpful. It would be even better if the authors could also list out the meaning of different symbols somewhere (either in the main text or supplementary). For instance, φ is the 'effective concentration' (or 'mass fraction'); λ is the growth rate; etc.

In response to the reviewer's suggestion, we have expanded the caption of Table S6 (Table E6 in the revised version) to remind readers of the meaning of the symbols.

4. The 'flux matching' section is a bit redundant. It might be better to move some of the details to the supplementary.

We understand the reviewer's concern, as the presentation seems redundant due to the repetition of similar sentences and formula for the 6 sectors. We, however, feel that it is necessary to "walk through" each of the sectors, to explain how the simple model (with essentially only flux matching and proteome constraint) can account for the sector behaviors. As reviewer #3 also pointed out, a unique aspect of our study is to use a simple theoretical model to understand complex -omics data.

5. Introduction, paragraph 2, typo: "adjusts" -> "adjust"

Corrected.

Reviewer #3:

This manuscript continues the line of highly interesting and stimulating papers from the Terri Hwa lab discussing general connections between protein expression and cell growth rate. Here, a proteomic experiment was performed to define how changes in growth rate influence the genome-wide profile of protein expression. This was done by gradually imposing a limitation in one of the three central aspects of cellular activities: translation, carbon uptake and amino-acid metabolism. The authors identify groups of proteins whose expression levels correlate (positively or negatively) with growth rate, and classify them based on this growth-rate correlation in the to the different limitations. The data is summarized by a phenomenological model, which connects the protein expression with growth rate.
The most unique aspect of this paper is the model used to summarize the data. Although one can argue that this model is an over-simplified version of the reality, I find this approach, of summarizing highly complicated data in a simplified manner using only a few parameters, refreshing and stimulating. I was in fact particularly surprised by the ability to predict basic features of how the proteome will be distributed when cells are growth in a new condition based on this simplified model.

We appreciate the accurate summary of our work by the reviewer and are grateful to the reviewer's positive assessment of the manuscript.

Few comments:
Relation to previous literature:
1. The question of growth-dependent gene/protein expression was studied extensively in the budding yeast. There are several studies which generated a lot of high quality data (see e.g. - Brauer et al. (2008) Coordination of growth rate, cell cycle, stress response, and metabolic activity in yeast. Mol Biol Cell 19: 352-367; Castrillo JIet al. (2007) Growth control of the eukaryote cell: a systems biology study in yeast. J Biol 6: 4; Regenberg B, et al. (2006) Growth-rate regulated genes have profound impact on interpretation of transcriptome profiling in Saccharomyces cerevisiae. Genome Biol 7: R107; Levy S, et al. (2007) Strategy of transcription regulation in the budding yeast. PLoS One 2: e250; Airoldi et al. (2009) Predicting cellular growth from gene expression signatures. PLoS Comput Biol 5: e1000257 and perhaps others). Some of those studies performed a real thorough characterization including expression profiles, proteome profiles, metabolic profiles etc. None of those
studies is discussed in the present MS. In fact, in one of those papers, a computational method was developed to predict growth rate from gene expression data. Other papers discussed whether the response to growth rate is direct or through a feed-forward like reading of the environment. I believe the data should not only be mentioned, but probably subject to some comparative analysis. It is really a large body of highly quantitative data of high quality that is very directly related to the present story and should therefore not be ignored.

We thank the reviewer for reminding us of these important works. We agree that a

summary and comparison would be useful and now include several paragraphs in the Discussion section doing so. However, we feel that there are important distinguishing characteristics between our work and previous studies that preclude a detailed comparative data analysis (Discussion and Expanded View Text 4). In any case, we are prepared to include the following material (between the lines of asterisks) as part of a new Expanded View should the editor find this necessary.

****

Microarray studies in *S. cerevisiae*

A number of studies over the last decade have carefully measured the growth rate dependence of mRNA transcript levels, proteins, and metabolites in Baker's yeast under various nutrient limiting conditions in chemostat (e.g., Airoldi *et al* 2009, Levy *et al* 2007, Brauer *et al* 2008, Regenberg *et al*, and Castrillo *et al* 2007). Given their complementary focus, we feel it is important to discuss these early works. We point out, for reasons fully explained in Expanded View Text 4, that changes in the abundance of any given mRNA should not be taken as a straight measure of the abundance of the corresponding protein. With that caveat in mind, we now compare the general conclusions reached by the various studies.

A common finding between all the studies is a positive correlation between ribosomal proteins and the growth rate λ. These results are unsurprising, and likely reflect the obligatory relationship between ribosome levels and growth rate outside of ribosome limiting conditions (e.g. chloramphenicol) which were not probed in these studies. Notably, [Levy] report a general decrease in ribosomal protein mRNA synthesis rates as the cell nears the end of exponential growth and runs out of nutrients.

[Castrillo] and [Regenberg] report divergent behavior between functional gene classes as growth rate is varied by nutrient limitation. Focusing on the carbon limitation condition (set by chemostat control of glucose), both studies report groups of genes (by mRNA in [Regenberg], by protein in [Castrillo]) that increase with growth rate, i.e. that are down-regulated by carbon limitation. Additionally, [Castrillo] report a large cluster of enzymes that correlate negatively with growth rate, i.e. that are specifically up-regulated with increasing carbon limitation. This class consists largely of proteins employed in **cellular carbohydrate metabolism**, **cellular macromolecule catabolism**, **transport**, and **response to stress**. This finding is in good agreement with our C and S sectors which exhibit a similar general trajectory under carbon limitation, and are dominated by similar descriptive terms in our GO analysis (**ion transport**, **tricarboxylic acid cycle**, **carbohydrate metabolic process**, and **response to stress**).

Upon casual inspection, the protein measurements in [Castrillo] appear to contradict the findings of [Regenberg], who report only one cluster (Cluster 13) that increases upon carbon limitation, and thirteen that increase or have no clear trend. However, the

authors note that a number of ORFs were found to decrease linearly with growth rate and that the entire dataset was normalized such that a small subset (42) of these ORFs would exhibit growth rate independent behavior. With this information in hand, Clusters 8 through 10 (which exhibit no strong relationship to λ) likely decrease with growth rate. Inspecting the dominant GO terms for these Clusters, we find **transport**, **carboxylic acid metabolism**, **main pathways of carbohydrate metabolism**, and **energy pathways**. Moreover, the most dominant GO term in Cluster 13 is reported as **autophagy**, a classic stress response. Thus, upon correcting for the normalization, we find that clusters in [Regenberg] downregulated by carbon limitation largely correspond to those reported in [Castrillo], as well as to our C and S sectors.

Stressing the strong case for skepticism in equating trends at the transcript and protein levels (as discussed in Klumpp et al, 2009), the studies tend to reinforce one another in the carbon limitation case. It would be valuable to look more deeply at the response for carbon, and nitrogen limitation reported in *S. cerevisiae* and *E. coli*, as well as for other limitations (e.g. ribosome slowing, sulfur, phosphate).

[Airoldi] focus on the inference problem of predicting growth rate from relative gene expression levels, i.e. the backwards problem of our study. For simplicity, they exclude genes that have non-uniform correlation with λ across differing nutrient limitations (in our study the R, U, and S sectors harbor such genes, when the ribosome slowing limitation is excluded). They find that a linear model can accurately predict cellular growth rate from the measurement of a small set of reporter genes. This comports with our finding that the majority of proteins change linearly with λ in a characteristic fashion.

Finally, [Brauer] study the growth rate dependence of the Yeast transcriptome across six major nutrient limitations. We focus here on the glucose and ammonia limitations. The authors find that ~60% of the variance can be explained by 3 “eigengenes”: two that decrease upon every limitation, and another that increases upon every limitation. Focusing on the nutrient limitations common with our study, the eigengenes of the first case would encompass the behavior of the R and U sectors, while the second case would describe our S sector. Strikingly, there does not appear to be a major eigengene with opposite behavior in the glucose, and nitrogen limiting conditions as we find with the prominent C and A sectors in *E. coli*. As with the other studies, [Brauer] report the positive correlation between ribosomal genes and λ.

****

2. The way by which limitations were imposed in the present papers were either drug, or differential expression of some enzymes. In contrast, in the previous studies mentioned above, growth rate limitations were imposed more naturally, through the use of a chemostat with a well-defined limiting factor. This difference may be fundamental to the way the cells react, since by introducing a drug or an effective mutation, the authors break the typical relationship between what the cells perceives

as its environment and its actual growth potential. The fact that this could result in some differential response was discussed quite extensively; e.g. Brauer et al. show that when subjecting cells mutated in the lysine pathway to lysine limitations result in a response that is different from the typical growth-rate related response. The Botstein lab further demonstrated that 'normally' starving cells (due e.g. to running away of glucose) survive for a significantly longer time than cells
which are starved due to some mutation (e.g. lack of an enzyme in the lysine pathway). Van-oudenaarden lab showed that glucose effects growth rate both through influx and through perception. Additional relevant paper from the Kishony lab treated more explicitly the case of drugs which limit translation. All in all, I believe the authors should discuss the 'un-natural' aspects of their perturbations and the possible implications.

While the use of antibiotic drug such as chloramphenicol for inhibiting translation has been long established, we understand the reviewer's concern of "un-natural" growth limitations by using synthetic strains. In fact, the exact same concerns have been addressed in a previous study (You et al., 2013) where the "titratable uptake systems" were first introduced to probe growth-rate dependence of gene expression. In that study, catabolic gene expression as represented by the activity of the native lacZ promoter were characterized in the traditional continuous culture setup where the dilution rate was dialed in lactose- or ammonia- limited chemostat, a microfluidic setup where the concentration of lactose or ammonia was dialed, and in batch culture growth of the titratable mutants where the uptake rate of lactose or ammonia was dialed. Very similar behavior (i.e., the same linear growth rate dependence) was found among all these conditions. We therefore believe that the titratable lactose or ammonia uptake constructs we employ in this study faithfully mimic the more natural environmental limitation of carbon or nitrogen.

We have added the following sentences in the section where we introduce the growth limitations:

"Such "titratable uptake systems" have been characterized in detail and found comparable to other modes of growth limitations such as those derived from continuous culture or microfluidic devices (You *et al*, 2013). "

Data presentation:
3. The authors analyzed their data by grouping together proteins with similar growth-rate correlation. I support this approach. However, the repeated claims of its originality and novelty are disturbing. I would carefully say that this grouping genes based on correlated expression is now the standard approach of analyzing high-throughput data and the first this one would do when trying to make sense of its overall behavior. This has to be changed in many places in the MS, including the results in discussion.

We thank the reviewer for highlighting this perception of our claims. On review, we do not find "repeated claims of its originality" (in reference to the grouping together of

similarly behaved proteins). However, we understand the perception and that our intended point did not come across can only be attributed to a failure of explanation on our part. In particular, we failed to clearly distinguish grouping procedures from what we mean by "coarse graining" (which is built upon, but is not, the grouping of experimental data).

We point out that "coarse graining" has come into use recently and that we feel the current investigation is, as yet, an ambitious application of coarse graining at the global scale. Hierarchical clustering has of course, for almost two decades, been used extensively to group similar patterns together (in many fields). We hope we have no illusion regarding this rich history.

Coarse graining goes a step beyond grouping procedures. Coarse graining accepts the outcome of grouping procedures as real objects that can be modeled in their own right. It is the coarse-graining procedure which collapses hundreds of curves in a cluster (e.g,. Fig. 2C) into a few numbers (Fig. 3), which can then be modeled by coarse-grained theory that has predictive power (e.g., Fig. 7). If it weren't for coarse graining, one would be left to compare *visually* the clusters corresponding to Fig. 3 and Fig. 7, and make some qualitative statements. Indeed, it is the coarse graining procedure that we claim as novel for omic studies.

The challenge of coarse graining has to do with the nature of the data. If all one has is the list of relative abundances (as e.g., in mRNA microarray), it is not clear how coarse graining can be done in a meaningful way. In this work, coarse graining is done by directly summing up the absolute abundances of individual proteins. The coarse-grained quantity is the fraction of the proteome a cell devotes to a certain process, e.g., glycolysis or amino acid synthesis, and this quantity directly feeds into the resource allocation model we developed.

We have further explained this point at the top of the section named "Coarse-grained proteome sectors", and in Expanded View Text 4.

Notation like 'super-enzymes' should be avoided in my opinion as they do not really add to the science.

We opt to refer to these as coarse-grained enzymes, which hopefully makes it clear that we refer to collections of enzymes, carrying out roughly similar tasks, which are regulated in coordination. We have made these changes in the "Introduction", "Qualitative proteome responses to growth limitations", and "Flux matching"

4. The authors refer to 'general' growth-rate related behavior of gene groups based on two conditions only (in the third, the group behavior is changing). The fact that two different conditions result in a similar growth-rate dependent behavior is nice, but still, I would find it a bit dangerous to generalize based on two conditions. Moreover, it will not be too surprising to find that the classification of proteins into different sectors

differs depending on the imposed limitation; for example, if only the C-limitation was imposed, the S and C sections would merge. Similarly, other conditions could exist that will further separate the different sectors, and will produce different classification. This doesn't invalidate the authors' approach for the analysis of the specific data, but does call for extra caution in the generalization and presentation of the reasons.

We certainly do not mean to claim the 6 sectors are the only way to partition the proteome under all conditions. They are what can be resolved under the experimental conditions studied, i.e., C-, A-, and R- limitations. We would expect that under other forms of limitations, other relevant groups of proteins would be induced. In the revision, we have added the following sentence to incorporate this point in the second paragraph of the discussion section:

"Note that the sectors are revealed by the growth limitations studied here and we expect new sectors to emerge under other growth limitations."

But we do expect that the non-induced proteins to decrease their abundances linearly with the growth rate (the 'general response') as observed in our experiments. This is what we built in our model. The reviewer is certainly right in that the general response was proposed based on two out of the three conditions. But it was seen again in Fig. 7 in the context of our test example (C- and A- limitations involving glycerol). Indeed, the predictability of our model depends crucially on the occurrence of the general response. In the revised text, we clarify this point by emphasizing in the 'Model prediction and testing' section that the general response is a proposal based on the observed data.

To further test the generality of our results, we have added in the revised version a new set of experimental data, where we imposed a completely different mode of growth limitation, by varying the growth rate by expressing a useless protein (LacZ) which effectively controls the parameter ϕ_{max} in our model. This is the most direct test of the proposed general response, since according to our model all proteins should behave as in general response. The model prediction and experimental data are indeed found to be in good agreement; see Fig. 7. We hope this additional data further substantiate the basis of our model.

5. Much of the important information is given only in the SI. The figures remaining in the main text are a bit shallow. While I support simplicity, I believe this was over-done in this case, and in fact when reading the MS I had to continuously refer to the SI; figure 1, for example, contains no real data - once could easily add to it the information about how actual strains, characterize some of their behavior, with some controls etc.,. Figure 2 and figure 3 could easily be integrated, which would also help the reader. Figure 4 is almost identical to figure 2, and while it is nice to show that the computational curves fit, it could easily be integrated with figure this would leave at least two additional figures (or perhaps more) where the authors could show 'hard' data - to make the actual research and results more apparent, less artificial and more

easy to appreciate.

We agree with the reviewer on the problem of having some essential information in the SI. We have thus included a new section “Quantitative proteomic mass spectrometry”. The section is supported with a new main figure (as Fig. 2 in the revised version), which contains not only control experiment results (Fig. 2AB, originally Fig. S3AB) but also mass spectrometry data (Fig. 2C, originally Fig. S5).

While we are not opposed to integrating Fig. 2 and Fig. 3, we think each of them carries sufficient information to stand as independent figures. Fig. 2 is the main item we refer to when we illustrate the coarse-graining approach, a key ingredient of our work. In fact, the figure is referred to 18 times from the main text. Fig. 3 shows the results of the GO analysis, with a total number of 15 GO terms representing the sector functions. In our attempts to join the two figures we found the result to be highly condensed and we think this arrangement would not be helpful to the reader. We thus propose to keep them as separate figures (as Fig. 3 and Fig. 4 in the revised version). We welcome any suggestions that can compact without making any given figure too dense.

Fig. 4 summarizes the main result of our work, a quantitative proteome-based flux model. While the experimental data are identical to those in Fig. 2, we deliberately show both of them to illustrate the power of the model, i.e., a simple model with not only less number of parameters but also biologically meaningful parameters (instead of simple fit) is able to capture most of the coarse-grained behaviors. Again, we are open to suggestions to improve the presentation.

Model:
6. The model provides a nice interpretation of the data, although it is not clear to me whether it goes beyond simply stating what one sees. For example, Eq. 4-5 is simply the statement expression of each sector correlates with growth rate. Eq. 9 is also the direct consequence of the pattern by which the different identified sectors change with the different limitations (that some sectors don't change while other change similarly in two of the three conditions). So it is a nice representation of the data, but I'm not sure whether it goes much beyond that.

As a key component of our results, the model is much more than simply representing the data. The reason is two-fold. First, the model reduces the number of parameters considerably, as compared to simple representation. In a direct description of the data, we would need 24 parameters even if assuming linear growth-rate dependence of each sector -- with 4 parameters (the point at the glucose standard condition and three intercepts on y-axis) for each of the 6 sectors. With further simplification from the assumption of general response (i.e., a single line describing responses by multiple sectors), we would still need 16 parameters, with 1 for the O-sector and 3 (the point at the glucose standard condition and two y-intercepts) for each of the remaining 5 sectors. The model allows us to reduce the number of parameters to 10 (see Table E6).

5 of these parameters (the y-intercepts of each sector) are constants and determined once and for all for the strain studied. Then with the remaining 5 parameters specifying the proteome allocation at one standard condition (glucose min medium, could have been some other choice), the model fixes the proteome allocation in all other combinations of C-, A- and R- limitation.

Second, the model assigns physical meaning to the parameters by connecting to the metabolic processes. For example, the ν_σ's are not simply parameters describing the slopes of the general responses but instead they represent the effective enzymatic rate constants of the corresponding sectors. Biological insights gained from the model allow simple interpretation of the data (Fig. 6) and predictions for new growth conditions (Fig. 7).

7. As I said above, I do support the use of a simplified model to describe the data and generate biological hypothesis. Yet, the suggested biological interpretation of the model (or data representation?) raises many questions. Here are some examples:
a. What is the meaning of 'flux'? This may be clear in the case of ribosome (flux of proteins?) but is less defined in the other cases, and in particular considering the fact that each of the sector includes many proteins involved in many biological functions, metabolic pathways etc (and that, most probably although not shown, some of the proteins involved in relevant pathways are not part of the associated sectors). It is difficult to conceive that all proteins assigned e.g. to the a-section (and only them) define the flux of amino-acids, and the question is even more disturbing in the case of the C-section. So that the fact that expression of the different sectors correlate with growth rate is given by the data, but the interpretation that this means that the respective protein produce some 'flux' that precisely match this growth rate is less clear and not well defined.

We agree with the reviewer that our concept of flux can be better explained, in particular as we attempt to extend it from the previously elaborated C, A, and R sectors (Scott et al, 2010; You et al, 2013) to those newly introduced, U and S.

We've added an explicit definition of flux in the modeling section of the main text.

Because each enzyme carries out some kind of interconversion between molecular species in the cell, we can say that an enzyme carries out some amount of interconversion of material per unit time (in substrate molecules per unit time), which we term its flux. The flux of a collection of enzymes can be defined as the sum of the products that flow out from the terminal enzymes per unit time, multiplied by a stoichiometric factor which reflects the composition of the material.

For the collection of enzymes that we term the R sector, the flux is clearly the proteins translated by ribosomes. For the C sector, the flux comprises carbon skeletons and metabolites. For the A sector, it is largely amino acids. There are even some proteins, such as those involved in chemotaxis, that do not directly handle flux in batch culture

but are nonetheless coregulated as part of the C-sector, presumably reflecting their role in facilitating carbon flux in *E. coli*'s natural environment.

As the reviewer points out, it is unlikely that any sector can be said to carry out a single task e.g. the processing of carbon precursors, to the total exclusion of all other sectors. We point out that in our model, the C and A sectors (nominally separated to handle catabolism, and anabolism) both share some flux with the S sector. As the reviewer states, any given sector that handles these processes cannot be said to define the flux of these processes alone.

After carefully establishing the correlative patterns in the MS data, and scrutinizing the nominal set of functions involved with each sector (using the abundance-based GO analysis), we observed that some minimal set of terms could, broadly define the major functions of each sector. Only after this analysis, did we think it useful to speculate as to the possible function of each sector. We do not mean to imply that these functions are mutually exclusive, only that they explain the roughly orthogonal functions of each sector.

b. Even if one accepts (and I'm not sure I do) that there is some well-defined flux generated by each of the sectors why would there be a substantial portion of the proteins not contribution to this flux (the 'fixed' level)? Furthermore, why would this fixed level not contribution to the generation of flux remain constant in different conditions? From a biological perspective, this is something that is not easy to accept.

The current model is an extension of a similar model presented in a previous study (Scott et al. 2010), where the possible origins of the offsets of the R-sector was discussed. As described in detail in response to Q6 of Reviewer 1, quite a number of molecular mechanisms for this offset have been discussed in the literature, starting with the existence of a fraction of non-translating ribosomes. As for the metabolic sectors, the simplest mechanism could be the biophysical difficulty to tightly repress gene expression, since zero offset requires that protein synthesis be completely turned off in non-inducing conditions. There may of course be many other physiological or ecological reasons why the cell may not want to turn off gene expression completely in non-inducing conditions that have been discussed in terms of trade-offs in recent literature. While these are speculations, we hope a phenomenological model like ours can lead to the biological origins of the phenomenon. In the revision, we have included the following sentences to the discussion:

"A variety of possible mechanisms have been proposed for the R-sector offset: A favorite early model was the existence of a fraction of non-translating ribosomes; see Scott *et al* (Scott *et al*, 2010) and references there. Zaslaver *et al* (Zaslaver *et al*, 2009) obtained an offset from ad hoc optimization scheme, while Klumpp et al (Klumpp *et al*, 2013) proposed another mechanism based on the growth-rate dependence of tRNA. For the offsets of the metabolic sectors, the simplest mechanism could be the biophysical difficulty to tightly repress gene expression, since a zero offset requires

protein synthesis to be completely turned off at zero growth rate. ”

c. Another assumption is that the 'flux' depends on the relative fraction of the sector, rather than the total (absolute) level of enzyme. I understand the justification of this assumption in the context of the ribosome, but is this clearly generalized to other types of fluxes?

We thank the reviewer for asking this question, as it reflects a failure of explaining important basic concepts on our part. As the reviewer points out, we assume in our model that each sector supports a flux, and that the 'flux' is proportional to the proteome fraction (mass fraction) occupied by that sector. In fact, the proteome fraction of a protein is a stand-in for the enzyme concentration (which is what we believe the reviewer means by the “total absolute level of enzyme”):

We have explained this point in detail in the new Expanded View Text 4.

Reviewer #4:

In this work, Hui et al. have extended the previous phenomenological work from their groups.

In the current study, the authors control the growth rate of E. coli cultures using three different growth limitations: limiting carbon intake, limiting nitrogen intake, and inhibiting translation. They find that for most of quantified enzymes, individual proteins either increase or decrease in abundance as a function of growth rate where the sign of the effect (increase or decrease) depends on the particular form of limitations (carbon, nitrogen, and translation). Based on individual enzyme's response, they cluster enzymes into 9 broad categories wherein enzymes belonging to each category respond to growth limitations in a particular way (e.g. increase with carbon, decrease with nitrogen, and translation). Notably, enzymes belonging to each of the category also have specific enrichment in GO annotation terms. The authors then construct a simple flux-matching model which allows them to predict coarse-grained proteome response as a function of combinatorial growth limitations.

I generally like this work. It is simple, phenomenological, and also provides the community with a good resource on enzyme abundances as a function of various growth limitations; which may be further analyzed by other researchers. I recommend publication of this paper in MSB after certain revisions.

We are grateful to the reviewer’s overall positive assessment of the manuscript.

1) I would like the authors to make the paper more readable by shortening it considerably. In my opinion, the introduction is longer than necessary. For example, the authors can do away with the statistical physics analogies.

We agree with the reviewer and have found opportunities to trim the introductory section. On the other hand, we feel strongly that the comparison between macroscopic quantities in thermodynamics, and coarse-grained proteome responses is a useful one and opt to keep it in the text (though we have kept it to a single place, compared with the multiple appearances in the first draft).

Also, the discussion on enrichment of GO annotations in each sector can also be considerably shortened by simply highlighting the findings in the main text and moving some of the details to supplementary section.

We regret that the reviewer thinks the GO analysis section is too long. We however think that it is necessary to go through each of the GO terms for each of the sectors. In contrary to the GO enrichment analysis of most existing studies where the results are mostly only “discussed”, our results allow us to extract a coarse-grained flux network (Fig. 4B), which we used to build the quantitative model.

In the section on flux matching, while it is clear how one arrives at Eq. 1 to 5, the introduction of f due to contamination of the so-called S sector is somewhat unclear. The authors may want to discuss that in some detail.

We have reworded the part to hopefully make the meaning of f clearer.

Finally, almost half of the discussion section is spent in a recap of the introduction and the results. I believe that this is unnecessary. The authors can use the discussion section to briefly discuss basic principles governing cellular growth and future experimental and theoretical direction.

We have removed “recap” material from the discussion section.

2) Minor point that the authors may wish to address: The authors use the classification term "upwards" for enzymes whose abundance is negatively correlated with growth rate (with a downward slope) and vice versa. I suggest that the authors reverse this somewhat confusing notation.

When preparing the manuscript, we also realized the possible confusion caused by the terms “upwards” and “downward” if thinking of responses to growth rate. We still decided to use them because after consulting with biology colleagues we found that it is actually more intuitive to think of responses to stress (slower growth), instead of the lack of stress (faster growth).

2nd Editorial Decision 24 November 2014

Thank you again for submitting your work to Molecular Systems Biology. We are now globally satisfied with the modifications made and I am pleased to inform you that we will be able to accept your paper pending the following minor modifications:

- please include the discussion of the related microarray studies in the Expanded Text and refer to it briefly from the Discussion section.
- we are grateful that you deposited the proteomics data in PRIDE and provide individual visual plots for each protein. It would be great if you could actually provide the latter data as a table of numerical values so that readers can re-plot a selection of the data themselves. This would be equivalent to the source data of the heatmap shown in figure 2C and can be submitted as Source Data

2nd Revision - authors' response 11 December 2014

We are grateful to the editorial team for accepting our manuscript pending minor modifications. We have responded to each of the requests as described in detail below.

- please include the discussion of the related microarray studies in the Expanded Text and refer to it briefly from the Discussion section.

This discussion on microarray studies is now the Expanded View Text 4, and is referred to from the Discussion section.

- we are grateful that you deposited the proteomics data in PRIDE and provide individual visual plots for each protein. It would be great if you could actually provide the latter data as a table of numerical values so that readers can re-plot a selection of the data themselves. This would be equivalent to the source data of the heatmap shown in figure 2C and can be submitted as "Source Data for Figure 2C" as a dataset file (Excel, tsv or csv).

The dataset is already included in Table E2 (as TableE2.xlsx), which is also referred to from the caption of Fig 2C.

© European Molecular Biology Organization
